# An α‐Cyclopropanation of Carbonyl Derivatives by Oxidative Umpolung

**DOI:** 10.1002/anie.202007439

**Published:** 2020-08-17

**Authors:** Adriano Bauer, Giovanni Di Mauro, Jing Li, Nuno Maulide

**Affiliations:** ^1^ Institute of Organic Chemistry University of Vienna Währinger Strasse 38 1090 Vienna Austria; ^2^ Department of Chemistry Tohoku University Aoba-ku 980-8578 Sendai Japan

**Keywords:** amide activation, hypervalent iodine, non-classical carbocation, umpolung

## Abstract

The reactivity of iodine(III) reagents towards nucleophiles is often associated with umpolung and cationic mechanisms. Herein, we report a general process converting a range of ketone derivatives into α‐cyclopropanated ketones by oxidative umpolung. Mechanistic investigation and careful characterization of side products revealed that the reaction follows an unexpected pathway and suggests the intermediacy of non‐classical carbocations.

## Graphical abstract

**An impossible triangle?** Iodine(III) promotes the α‐cyclopropanation of simple ketone derivatives. Mechanistic studies reveal that an unusual intermediate might be involved.

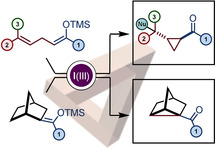

The importance of the carbonyl functionality has been identified already at the dawn of modern organic chemistry in the 19th century and remains at a cardinal point of chemical synthesis. With the advent of the umpolung approach, developed by Seebach and Corey, an important paradigm shift was provided, delivering a systematic perspective to overcome the limitations of the natural polarity of the carbonyls (Scheme 1 a–I).^1^, ^2^ The inversion of the polarity through derivatization was showcased first by dithiane chemistry, where a thioketal could be effectively used as C1 nucleophile (Scheme 1 a–II).^1^, ^3^ Some umpolung reagents such as the cyanide anion^4^, ^5^ or thiazolium based NHCs^6^ (both react as C1 nucleophiles and derive formally from formic acid) can transfer this umpolung‐reactivity through catalysis (Scheme 1 a–III).^7^ A third approach to reverse the polarity of a given functional group consist in its oxidation or reduction by an external or internal reagent (Scheme 1 a–IV).^8^ Ketones and their derivatives for instance are commonly employed in oxidative umpolung reactions. Classically promoted by toxic elements such as Hg(II),^9^, ^10^ Tl(III),^9^, ^11^ Pb(IV),^9^, ^12^, ^13^ or Se(IV) 14 more modern variants rely on Bi(V),^15^ *N*‐oxides,^16^, ^17^, ^18^, ^19^ Mn(III),^20^, ^21^ halosuccinimides,^22^ sulfoxides,^23^ and on iodine(III).^24^, ^25^, ^26^, ^27^, ^28^, ^29^

**Scheme 1:**
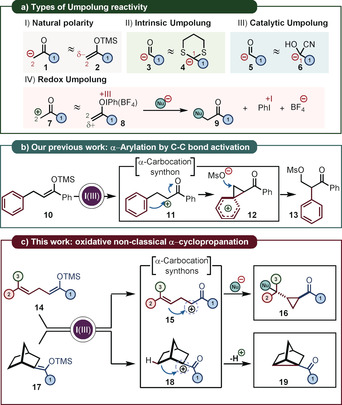
a) Natural polarity and types of umpolung. b) α‐Arylation by C−C bond activation. c) This work: oxidative α‐cyclopropanation.

The α‐functionalization of certain ketones through oxidative umpolung with iodine(III) was pioneered already in the 1960s,^30^, ^31^ has been extensively investigated in the 1980s,^32^, ^33^, ^34^, ^35^, ^36^ and gained further attention in more recent years.^37^, ^38^, ^39^, ^40^, ^41^ It is believed that the reaction involves an enolonium species (compound **8**, Scheme 1 a).^42^, ^43^ This highly electrophilic intermediate can react with a variety of different nucleophiles. Such oxidations are often promoted by acids^32^ or less frequently by bases,^44^ with enol ethers^32^, ^41^ or active methylene compounds^37^, ^38^ being commonly employed.

In many cases however, the enolonium species does not directly react with an external nucleophile. Instead, skeletal rearrangements are observed leading to stabilized carbocationic intermediates. Net 1,2‐phenyl migrations for instance which proceed *via* a phenonium intermediate^45^, ^46^ are frequently witnessed (Scheme 1 b).^47^, ^48^, ^49^ This illustrates that α‐umpolung chemistry of carbonyl compounds can be leveraged to a new avenue for carbocationic rearrangements which no longer require halide/leaving group abstractions^50^, ^51^ or protonation of carbon–carbon multiple bonds.^52^, ^53^, ^54^ Building on these developments, we herein report a new protocol for cyclopropanation by exploiting the propensity of certain enolonium species to engage in carbocationic rearrangements.

Inspired by the aforementioned reports, we started to investigate the enolonium species, which is prone to rearrangement reactions. The allylic silylenol ether **14 a** was chosen as the first substrate since homoallylic cations are known to undergo fast rearrangements.^55^, ^56^, ^57^, ^58^, ^59^, ^60^ When treated with activated iodosobenzene at low temperature, fast formation of the cyclopropane **16 aa** was observed (Scheme 2 a). The product was isolated in more than 70 % yield as a single diastereoisomer (*trans*). The only other product detected was the diastereomerically pure cyclobutane **20 aa**. The regiochemical outcome of the formation of **20 aa** was surprising, since a classical Prins‐type attack of the double bond should lead to a 1,3‐substituted cyclobutane product. This result urged us to investigate the mechanism further by isotope labeling.

**Scheme 2:**
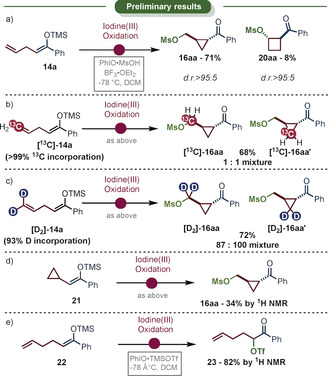
a) Preliminary result. b) ^13^C labelling study. c) Deuterium labelling study. The slight imbalance in isotopologue‐ratio can be accounted for by a secondary isotope effect. d) The *trans* cyclopropane can be synthesized similarly from a cyclopropyl silylenol ether. e) Other appended double bonds do not participate during the oxidation event.

When the substrate **14 a**, marked with a ^13^C‐label at the terminal olefin, was employed the product was observed to be a 1:1 mixture of isotopomers (Scheme 2 b). Similar results were obtained by deuterium labeling (Scheme 2 c). However, here a slight imbalance of the two isotopologues was observed, in favor of the endocyclically labeled compound **[D_2_]‐14 aa′**, which is suggestive of a secondary kinetic isotope effect.^88^

Both results are consistent with a highly fluxional intermediate and suggest the intermediacy of a non‐classical cyclopropylcarbinyl cation at least as a transient species. Two additional experiments further ascertained our assumption. The β‐cyclopropyl silylenol ether **21** led to the formation of the same product, likely by the formation of the same cationic intermediate (Scheme 2 d). Furthermore, when an additional CH_2_‐spacer was installed between the olefin and the silyl enolether (Scheme 2 e–**22**) cyclization was completely absent.^89^ Instead, a simple α‐functionalization took place showcasing that the homoallylic double bond does not participate in a general Prins‐type nucleophilic attack.

Based on our findings and previously reported experiments we propose the following mechanism for the cyclopropanation of **14 a** (Scheme 3): First the metastable enolonium species **24** (or **25** respectively) is formed which interacts with the nucleophilic alkene in close proximity. Under extrusion of PhI a non‐classical *C*_s_ symmetric cyclopropyl carbinyl cation is formed (**26**), which may be in equilibrium with two enantiomeric classical carbocations (**27**/**28**).^90^ Those can be trapped to yield the observed *trans* cyclopropane. The equilibrium is highly dynamic and thus other carbocations may contribute. However, the cyclobutonium **29**/**30** might benefit from carbonyl bridging, explaining the formation and the stereochemical outcome observed for side product **20 aa**.

**Scheme 3:**
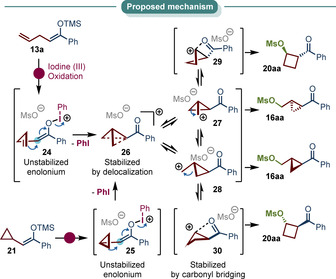
Proposed mechanism for the cyclopropanation of linear silylenol ethers.

Although homoallylic rearrangements involving non‐classical carbocations have been studied previously, they typically lead to inseparable mixtures of products and are perceived as synthetically unappealing.^55^, ^56^, ^57^, ^58^, ^59^, ^60^

As shown in Scheme 4, a broad variety of substrates could engage in this oxidative cyclopropanation in good yields and with high diastereoselectivities. A range of aromatic rings, ranging from electron‐poor to electron‐rich, was tolerated with no large effect on yield and/or selectivity. The aromatic ring itself is not required for high yields or selectivities since aliphatic ketones gave comparable results (**16 ia**–**16 ja**). Moreover, as the precursor of **16 ja** was employed as a 1:1 *E:Z* mixture (see Supporting Information for details), we believe that the geometrical purity of the starting material is inconsequential to the reaction outcome.^91^

**Scheme 4:**
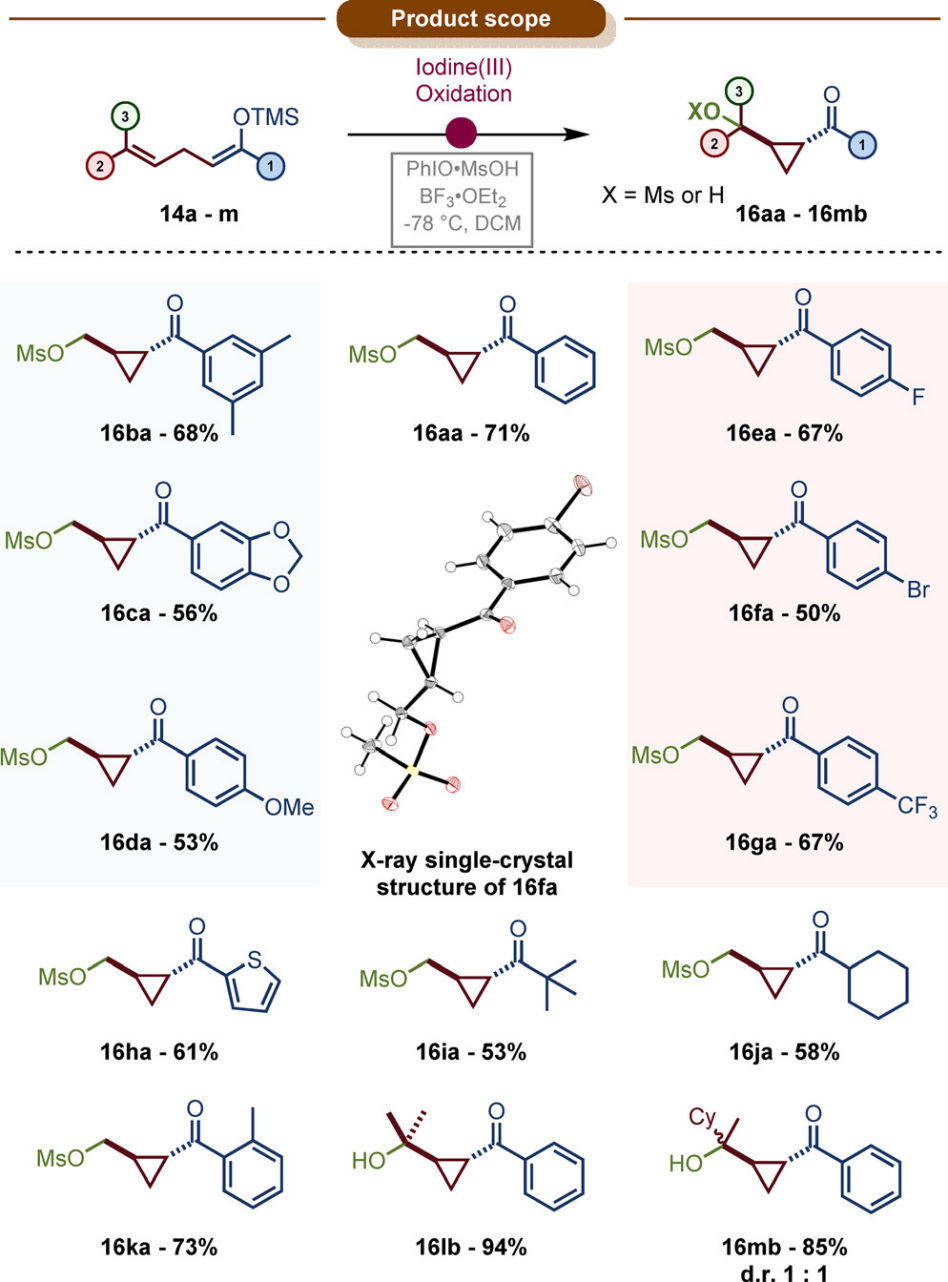
Product scope of the oxidative α‐cyclopropanation of linear ketone derivatives. All yields refer to pure, isolated material. Reactions conducted on a 0.2 mmol scale, see Supporting Information for reaction conditions.

Interestingly, trisubstituted alkenes did not give the expected mesylate product but the corresponding alcohol instead (**16 lb**, **16 mb**). Although *trans* selectivity for the cyclopropanation was excellent and yields were typically higher, the reaction showed no selectivity over a third stereogenic center in the cyclopropylcarbinyl position (**16 mb**), possibly a result of the intermediacy of a highly stabilized tertiary cyclopropylcarbinyl cation.^61^

The syntheses of cyclopropanes are often mediated by metals,^62^, ^63^, ^64^ but metal free approaches are also known^65^, ^66^ such as the Johnson–Corey–Chaykovsky reaction.^67^, ^68^ Several reviews have been published.^69^, ^70^, ^71^ Certain α‐cyclopropyl ketones can be generated from similar starting materials by epoxidizing the alkene using NBS under strongly basic conditions, followed by deprotonation of the ketone.^72^ However, those approaches suffer from reproducibility issues^73^ and are limited to the formation of the alcohol.^92^ Herein, we were able to show that the oxidative umpolung approach allows the decoration of the cyclopropylcarbonyl cation with a variety of nucleophiles in good yields and exclusive *trans* selectivities using trimethylsilyl trifluoromethanesulfonate (TMSOTf) as the activator (Scheme 5). Halides, such as chloride or iodide (**16 ad**–**16 ae**), but also oxygen‐ (**16 ah**) and sulfur‐nucleophiles (**16 ag**) were found to be competent reaction partners. We were also pleased to isolate the fluorinated product **16 ac** in very good yield and excellent diastereoselectivity.

**Scheme 5:**
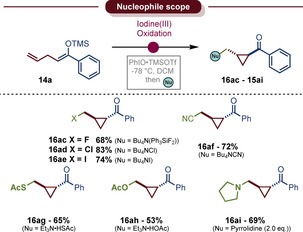
Nucleophile scope of the oxidative α‐cyclopropanation of linear ketone derivatives. All yields refer to the pure, isolated *trans* isomer, unless otherwise stated. Reactions conducted on a 0.2 mmol scale, see Supporting Information for reaction conditions.

At this stage, we were intrigued by the possibility of alternative rearrangements using other silylenol ether scaffolds. Because our experiments indicate the presence of the non‐classical cyclopropylcarbinyl carbocation, we were wondering if an enolonium species reminiscent to the 2‐norbornyl cation would lead to similar results (Scheme 6). The 2‐norbornyl cation has been part of one of the most famous (and long) debates in organic chemistry.^74^, ^75^, ^76^ When compound **17 b** was submitted to similar reaction conditions again a cyclopropanated product was observed in good yield: the nortricyclene **19 b** (Scheme 6).^77^, ^78^, ^79^, ^80^ Nortricyclene formation is often observed when non‐classical 2‐norbornyl carbocations are generated (compound **31**). This is believed to result from a γ‐elimination of the non‐classical cation,^78^, ^79^, ^80^, ^81^, ^82^ which can be interpreted as a metastable protonated cyclopropane. It is also interesting to note that this cation has not only been generated by halide abstraction of a 2‐halonorbornyl precursor but also by protonation of nortricyclene.^83^ This reaction is applicable to a broad array of different ketones. Importantly, the hypothetical side‐product **32** and the rearranged product **33** were mostly absent from the reaction mixture.

**Scheme 6:**
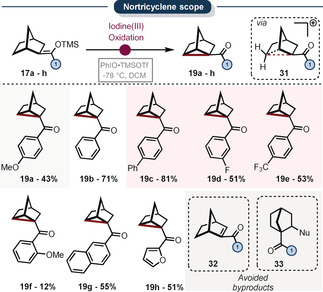
Ketone scope for the cyclopropanation *via* non‐classical 2‐norbornyl cations. All yields refer to pure, isolated material. Reactions conducted on a 0.2 mmol scale, see Supporting Information for reaction conditions.

Furthermore, we were pleased to find that 2‐norbornyl amides undergo oxidative cyclopropanation through amide umpolung chemistry using 2‐iodo pyridine, trifluoromethanesulfonic anhydride, and pyridine‐*N*‐oxide (PNO) subsequently.^84^, ^85^, ^86^, ^87^ Even though a pyridine base is used for this approach, elimination towards the norbornene derivative **36** represents only a minor reaction pathway (Scheme 7). This result showcases the aforementioned tenet of non‐classical carbocations, whereby different precursors can yield the same product.

**Scheme 7:**
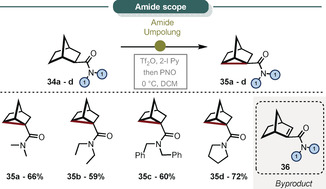
Nortricyclene synthesis by amide activation. All yields refer to pure, isolated material. Reactions conducted on a 0.2 mmol scale, see Supporting Information for reaction conditions.

In conclusion, we have demonstrated that 2‐electron oxidative umpolung is a valuable concept for the metal‐free construction of cyclopropanes. Our results strongly suggest the intermediacy of non‐classical carbocations and rely on the unusual stability of the intermediates involved.

## Conflict of interest

The authors declare no conflict of interest.

## Supporting information

As a service to our authors and readers, this journal provides supporting information supplied by the authors. Such materials are peer reviewed and may be re‐organized for online delivery, but are not copy‐edited or typeset. Technical support issues arising from supporting information (other than missing files) should be addressed to the authors.

Supplementary
