# Supplementary material for: An α‐Cyclopropanation of Carbonyl Derivatives by Oxidative Umpolung
Source: Angew Chem Int Ed Engl. 2020 Aug 17;59(41):18208–12. doi: 10.1002/anie.202007439 (PMC7589340; doi:10.1002/anie.202007439)
Supplement: Supplementary file 1 — Supplementary [file ANIE-59-18208-s001.pdf]

Angewandte Chemie
Eine Zeitschrift der Gesellschaft Deutscher Chemiker

Supporting Information

An α-Cyclopropanation of Carbonyl Derivatives by Oxidative Umpolung

*Adriano Bauer, Giovanni Di Mauro+, Jing Li+, and Nuno Maulide**

anie_202007439_sm_miscellaneous_information.pdf

Table of Contents

1) GENERAL 2
2) STARTING MATERIAL SYNTHESIS 3
Procedure A (Substrates 14a-h, 14k) 3
Procedure B (Substrate 14j) 4
Procedure C (Substrate 14i) 5
Procedure D (Substrates 14l-m) 6
Procedure E (Substrates [^{13}C]-14a, [D_2]-14a) 7
Procedure F 9
Procedure G 9
Procedure H 10
Procedure J (Substrates 17c-17g) 11
Procedure K (Substrates 17a-b, 17h) 11
3) EXPERIMENTAL SECTION 11
Procedure I (Products 16aa-mb and [D_2]-16aa/[^{13}C]-16aa) 11
Procedure II (Products 16ac-ai) 13
Procedure III (Products 19a-h) 14
Procedure IV (Products 30a-d, 31) 14
4) CHARACTERIZATION 15
5) NMR SPECTRA 42
6) BIBLIOGRAPHY 103
7) X-RAY ANALYSIS 103

1) General

Unless otherwise stated, all glassware was flame-dried before use and all reactions were performed under an atmosphere of argon. All solvents were distilled from appropriate drying agents prior to use. All reagents were used as received from commercial suppliers unless otherwise stated. Trifluoromethane sulfonic anhydride (triflic anhydride) was dried over P_2O_5 and distill under argon prior use. Iodosobenzene was prepared according to the literature.[1] Reaction progress was monitored by thin layer chromatography (TLC) performed on aluminum plates coated with silica gel F254 with 0.2 mm thickness. Chromatograms were visualized by fluorescence quenching with UV light at 254 nm or by staining using potassium permanganate. Flash column chromatography was performed using silica gel 60 (230-400 mesh, Merck and co.). Neat infrared spectra were recorded using a Perkin-Elmer Spectrum 100 FT-IR spectrometer. Wavenumbers (ν_{max}) are reported in cm^{-1}. Mass spectra were obtained using a Finnigan MAT 8200 or (70 eV) or an Agilent 5973 (70 eV) spectrometer, using electrospray ionization (ESI). All ^{1}H NMR and ^{13}C NMR spectra were recorded using a Bruker AV-400 or AV-600 spectrometer at 300K. Chemical shifts were given in parts per million (ppm, δ), referenced to the solvent peak of $CDCl_3$, defined at δ = 7.26 ppm (^{1}H-NMR) and δ = 77.16 (^{13}C-NMR). Coupling constants are quoted in Hz (J). ^{1}H NMR splitting patterns were designated as singlet (s), doublet (d), triplet (t), quartet (q) as they appeared in the spectrum. Splitting patterns that could not be interpreted or easily visualized were designated as multiplet (m) or broad (br).

2) Starting material synthesis

Procedure A (Substrates 14a-h, 14k)

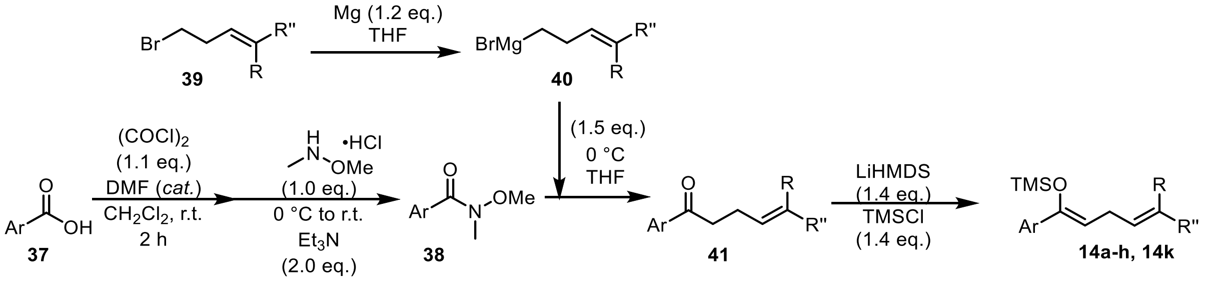

Scheme S 1

The first reaction step was carried out under air atmosphere. The benzoic acid **37** was dissolved in CH_2Cl_2 (0.3 M) and one drop of DMF was added. Then oxalyl chloride (1.1 eq.) was added quickly. After 2 h of stirring, the solvent was evaporated to yield the crude acyl chloride.

The hydrochloride salt of *N*-methoxy-*N*-methylamine (1.0 eq.) was dissolved in CH_2Cl_2 (0.3 M) and cooled to 0 °C. Et_3N (2.0 eq.) was added dropwise and stirred for 5 min at the same temperature. Then the neat acyl chloride was added dropwise to the solution. Afterwards, the flask containing the acyl chloride was rinsed with CH_2Cl_2 (5-10 mL). The solution was warmed up to room temperature and stirred for 2 h. Then the reaction was quenched with $HCl_{aq.}$ (1 M) and transferred into a separatory funnel. After washing with the aforementioned HCl solution, the organic layer was washed with saturated aqueous $NaHCO_3$, and with H_2O. After drying the organic phase over Na_2SO_4 the layer was filtered and volatiles were removed under reduced pressure. The Weinreb amide (**38**) was obtained without further purification.

Magnesium turnings (1.2 eq.) were suspended in THF (1/2 of the total volume) and a solution of 1-bromo-3-pentene (**39**) in THF (1/2 of the volume) was added slowly *via* a dropping funnel under vigorous stirring (concentration of the bromide after the addition was 0.5 M). The reaction was stirred until most of the magnesium had dissolved. A quantitative formation of the Grignard-reagent was assumed, and the solution was used as such. The previously obtained amide was dissolved in THF (0.4 M) and cooled to 0 °C. Then a THF-solution of 3-butene-1-magnesium bromide in THF (0.5 M) was added dropwise. The reaction was stirred for 3 h at 0 °C. Then the reaction was quenched with saturated aqueous NH_4Cl and extracted with Et_2O (2 x). The combined organic layers were dried over Na_2SO_4 and filtered. Evaporation of the solvent gave the crude ketone **41**, which was purified by column chromatography. (typical eluent - heptanes : EtOAc, 98 : 2 → 60 : 40 v/v%).[2]

A solution of LiHMDS in THF (0.5 M, 1.4 eq.) was cooled to 0 °C and a solution of the ketone **41** in THF (1 M, 1.0 eq.) was added dropwise. The mixture was stirred for 30 min at the same temperature. Afterwards, TMSCl (1.4 eq.) was added dropwise, the cooling bath was removed, and the reaction was stirred for another 2 h. Conversion was checked by TLC and after completion of the reaction, volatiles were removed under reduced pressure. The crude was purified by column chromatography to yield products **14a**, **14e-h** and **14k**. Most of the materials were very stable and non-polar and were eluted with pure heptanes. Electron-rich silyl enolethers were more susceptible to hydrolysis and therefore had to be filtered quickly through a silica plug to yield the desired product **14b-d** (typical eluent - heptanes : Et_2O, 96 : 4).[3]

Procedure B (Substrate 14j)

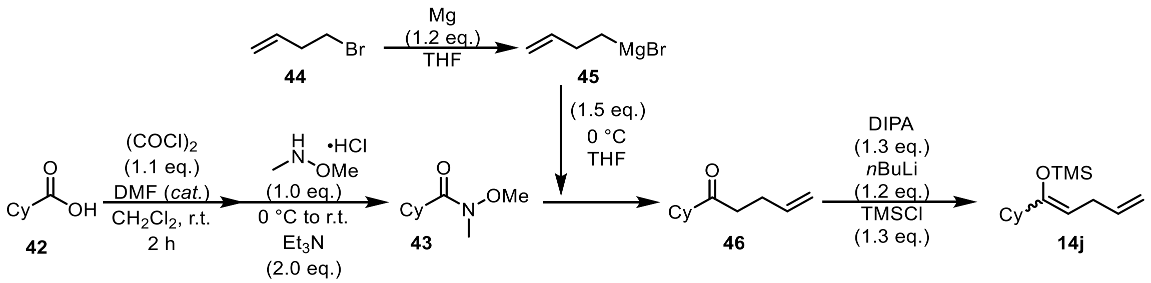

Scheme S 2

The ketone **46** was obtained by the same route described in general procedure A in 84% yield as a colorless liquid (see S*cheme S1*).

Freshly distilled di*iso*propylamine (1.3 eq., 1.3 mmol, 182 µL) was dissolved in THF (1 mL) at 0 °C and *n*BuLi in hexanes (2.5M, 1.2 eq., 1.2 mmol, 0.48 mL) was added dropwise. The mixture was stirred for 15 min at the same temperature and then cooled to -78 °C. A THF-solution of the ketone (1M, 1.0 eq., 1.0 mmol, 166 mg) was added dropwise. The mixture was warmed to room temperature over 30 min and TMSCl (1.3 eq., 1.3 mmol, 330 µL) was added. The reaction was stirred for 5 h at room temperature. The volatiles were removed under reduced pressure and the residue was dissolved in a minimal amount of $CHCl_3$. The dissolved crude was loaded on a silica plug, which was rinsed quickly with heptanes (ca. 200 mL). After evaporating the solvent, the product **14j** was obtained as a colorless liquid. (yield 235 mg, >95%).[4]

Procedure C (Substrate 14i)

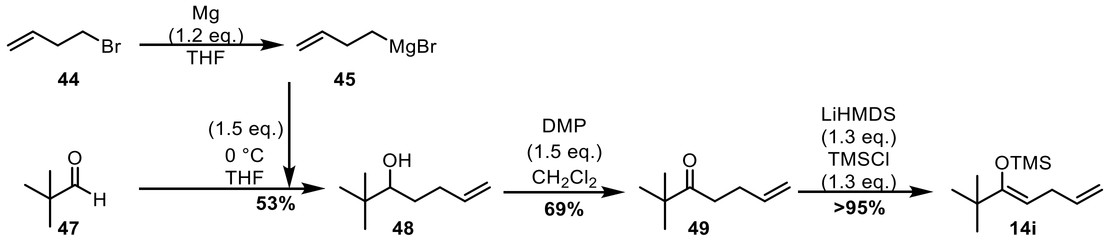

Scheme S 3

Pivalaldehyde was dissolved in THF (0.5 M, 5.0 mmol, 0.54 mL) at 0 °C and a THF-solution of 3-butene-1-magnesium bromide in THF (0.5 M, 15 mL, 1.5 eq.) was added dropwise. The reaction was stirred for 3 h at the same temperature. Afterwards, the excess Grignard reagent was quenched by the addition of saturated aqueous NH_4Cl. The aqueous layer was extracted with Et_2O (2x) and the combined organic phases were dried over Na_2SO_4 and filtered. Volatiles were removed under reduced pressure and the crude material was purified by column chromatography (eluent - heptanes : EtOAc, 90 : 10 → 50 : 50 v/v%) to yield the alcohol **48** as a yellow liquid (yield 380 mg, 53%).[5]

The alcohol (2.67 mmol, 380 mg) was dissolved in CH_2Cl_2 (0.2 M, 13.5 mL) and Dess-Martin-Periodinane was added at once (1.5 eq., 4.0 mmol, 1.70 g). The reaction was stirred 3 h at room temperature and subsequently treated with a saturated aqueous solution of $Na_2S_2O_3$. The mixture was extracted with Et_2O (3 x), dried over Na_2SO_4 and filtered. The volatiles were removed under reduced pressure and the crude was purified by column chromatography (eluent - heptanes : EtOAc, 100 : 0 → 70 : 30 v/v%) to yield the aldehyde as a yellow liquid (yield 259 mg, 69%).[6]

A solution of LiHMDS in THF (0.5 M, 1.3 eq., 2.4 mmol, 4.8 mL) was cooled to 0 °C and a solution of the ketone in THF (1 M, 1.0 eq., 1.85 mmol, 259 mg) was added dropwise. The mixture was stirred for 30 min at the same temperature. Afterwards, TMSCl (1.4 eq., 2.4 mmol, 305 µL) was added dropwise, the cooling bath was removed, and the reaction was stirred for 2 h. Volatiles were removed under reduced pressure. The crude was purified by column chromatography (eluent - heptanes : EtOAc, 100 : 0 → 70 : 30 v/v%) to yield the silyl enolether **14i** as a colorless liquid. (385 mg, >95%).[3]

Procedure D (Substrates 14l-m)

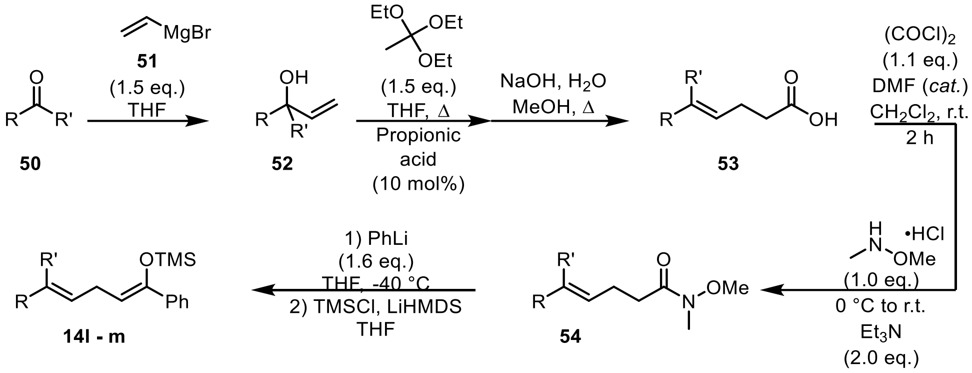

Scheme S 4

The ketone **50** (1.0 eq.) was dissolved in THF (0.5 M) at 0 °C. A THF solution of vinylmagnesium bromide **51** (1 M, 1.5 eq.) was added dropwise. The reaction was stirred at room temperature for 4 h before the mixture was quenched with saturated aqueous NH_4Cl. The aqueous layer was extracted with EtOAc and the product was purified by vacuum distillation to yield the alcohol **47**.

The alcohol **52** (1.0 eq.) was dissolved in triethyl orthoacetate (13.3 eq.) and propionic acid (10 mol%) was added as the catalyst. The reaction was heated to 140 °C for 12 h. After this time, most of the orthoacetate was distilled off using a Dean-Stark apparatus, and a solution of $NaOH_{aq.}$ (2.5M) in a H_2O-MeOH mixture was added (1:9 v/v%, equal volume of the used orthoacetate). The mixture was heated to 100 °C for 1 h. After cooling to room temperature, the mixture was diluted with $NaOH_{aq.}$ (1 M, equal volume of the used orthoacetate) and the aqueous phase was extracted with CH_2Cl_2. The aqueous layer was acidified to pH<1 by adding $HCl_{aq.}$ (4 M) and extracted again with CH_2Cl_2 (5x). The combined organic layer was dried over Na_2SO_4, filtered and volatiles were removed. The crude was purified by column chromatography (eluent - heptanes : EtOAc : AcOH, 93 : 5 : 2 → 60 : 38 : 2 v/v%) to yield the desired acid **53**.[7]

The acid **53** (1.0 eq.) was dissolved in CH_2Cl_2 (0.19 M) and two drops of DMF were added with a Pasteur pipette. Oxalyl chloride was added (1.1 eq.) and the reaction was stirred 2 h at room temperature. Thereafter, volatiles were removed to afford the crude acyl chloride. The hydrochloride salt of *N*-methoxy-*N*-methyl amine (1.1 eq.) was dissolved in CH_2Cl_2 (0.20 M) at 0 °C. Then Et_3N (2.2 eq.) was added dropwise. Then the neat acyl chloride was added dropwise, and the ice bath was removed thereafter. The reaction was stirred for 2 h before it was treated with HCl_{aq} (1 M), transferred to a separatory funnel and washed with the aqueous HCl solution. The organic phase was further washed with saturated aqueous $NaHCO_3$

and with H_2O. After drying the solution over Na_2SO_4, the organic phase was filtered and volatiles were removed to give the amide **54**, which was used without further purification.

The Weinreb amide **54** (1.0 eq.) was dissolved in THF (0.25 M) and cooled to -40 °C. A solution of phenyllithium in dibutylether (1.9M, 1.5 eq.) was added over 10 min. After stirring the mixture for further 10 min at the same temperature, MeOH was added dropwise (10 eq.) and the reaction was warmed to room temperature. Saturated aqueous NH_4Cl was added, and the aqueous phase was extracted with Et_2O (3x). The combined organic layer was dried over Na_2SO_4 and filtered. Volatiles were removed and the residue was purified by column chromatography (eluent - heptanes : EtOAc, 100 : 0 → 70 : 30 v/v) to give the desired pure ketone.[8]

A solution of LiHMDS in THF (0.5 M, 1.3 eq.) was cooled to 0 °C and a solution of the ketone in THF (1 M, 1.0 eq.) was added dropwise. The mixture was stirred for 30 min at the same temperature. Afterwards, TMSCl (1.4 eq.) was added dropwise, the cooling bath was removed and the reaction was stirred for 2 h. Volatiles were removed under reduced pressure. The crude material was purified by column chromatography (Eluent - heptanes : EtOAc, 100 : 0 → 70 : 30 v/v%) to yield the silyl enolether **7l-m**.[3]

Procedure E (Substrates [^{13}C]-14a, [D_2]-14a)

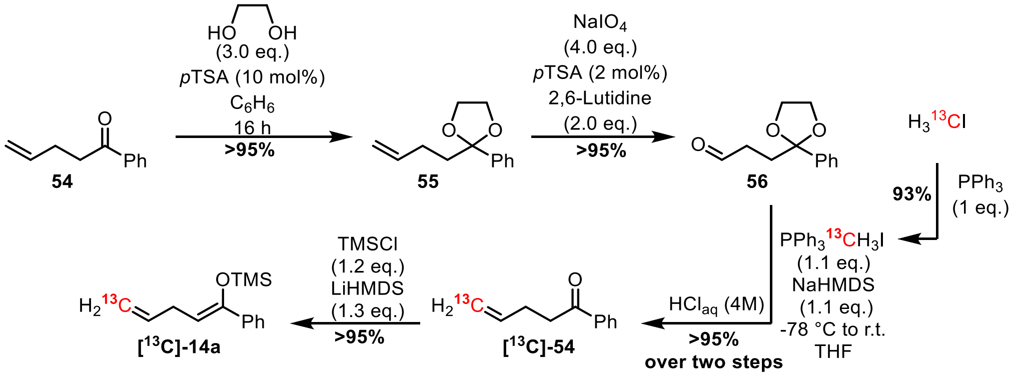

Scheme S 5

1-phenylpent-4-en-1-one **54** was synthesized according to the general procedure A.

1-phenylpent-4-en-1-one (1.0 eq., 10.7 mmol, 1.72 g) and ethylene glycol (3.0 eq., 32.2 mmol, 1.8 mL) were dissolved in benzene (60 mL). *para*-Toluenesulfonic acid monohydrate (10mol%, 1.1 mmol, 207 mg) was added to the solution, and the mixture was refluxed (oil bath temperature *ca.* 95 °C) using a Dean-stark-apparatus and a condenser. After 11 h, the reaction was cooled to room temperature and aqueous saturated $NaHCO_3$ was added. The separated aqueous layer was extracted with Et_2O (1x) and the combined organic layer was dried over Na_2SO_4 and filtered. After removing the solvent under reduced

pressure, the ketal was obtained as a colorless liquid (2.12 mg, >95% yield).[9] An analogous reaction in toluene gave a low yield and purification was troublesome.

The acetal (1.0 eq., 5.0 mmol, 1.02 g) was dissolved in a mixture of 1,4-dioxane (37.5 mL) and H_2O (12.5 mL). Lutidine (2.0 eq., 10.0 mmol, 1.16 mL), sodium periodate (4.0 eq., 20 mmol, 4.28 mg) and a solution of OsO_4 in H_2O (2mol%, 4 *w*/w%, 640 µL) were added in that order and the resulting solution was stirred until the starting material was no longer observable by TLC (3 h). The reaction was quenched with H_2O (10 mL) and aqueous saturated $Na_2S_2O_4$ solution (10 mL). $CH_2Cl_2$was added and the aqueous layer was extracted with further CH_2Cl_2 (3x). The combined organic layer was dried over Na_2SO_4, filtered and volatiles were removed under reduced pressure. The crude mixture was purified by column chromatography (eluent - heptanes : EtOAc, 100 : 0 → 50 : 50 v/v%) to yield the aldehyde **56** as a yellow liquid. (1.00 g, >95%)[10]

Triphenylphosphine (1.0 eq., 2.0 mmol, 525 mg) in THF (3 mL) was added to a solution of ^{13}C-methyl iodide in MTBE (1.0 eq., 2M, 1.0 mL, ^{13}C content >99%). The mixture was refluxed (oil bath temperature *ca.* 85°C) for 2 h and then cooled to room temperature. The crude Wittig salt was filtered and thoroughly washed with Et_2O. After drying under *vacuum*, the pure Wittig salt was obtained as a white powder (yield 749 mg, 93%).[11]

The Wittig salt (1.1 eq., 1.85 mmol, 749 mg) was dispensed in THF (11 mL) and cooled to 0 °C. A solution of NaHMDS in THF (1.1 eq., 2M, 920 µL) was added dropwise and stirred until the solution became clear. Then the mixture was cooled to -78 °C and a solution of the aldehyde **56** in THF was added (1.68M, 1.68 mmol, 347 mg). The mixture was warmed to room temperature and stirred until no aldehyde was observable by TLC. Thereafter, aqueous saturated NH_4Cl (*ca*. 3 mL) and aqueous HCl (4 M, *ca.* 10 mL) were added. The reaction was stirred for 30 min and the deprotection of the ketal was observed by TLC, before the mixture was diluted with Et_2O. Then the aqueous phase was extracted with more Et_2O (3x), the combined organic layers were dried over Na_2SO_4, filtered, and the volatiles were removed under reduced pressure. Column chromatography (eluent - heptanes : EtOAc, 100 : 0 → 60 : 40 v/v%) gave the ketone **[^{13}C]-54** as a colorless liquid (260 mg, >95%).[12]

A THF solution of LiHMDS (1.25 eq., 0.33M, 2.0 mmol) was cooled to 0 °C and a THF solution of the ketone (1.0 eq., 1.6 mmol, 260 mg) was added dropwise. The solution was stirred for 30 min, before TMSCl (1.20 eq., 1.9 mmol, 246 µL) was added. The reaction was stirred for 1 h at room temperature and quenched with aqueous saturated $NaHCO_3$. The aqueous layer was extracted with Et_2O (3x) and the combined organic layer was dried over Na_2SO_4 and filtered. Evaporation of the volatiles under reduced pressure gave the crude silyl enol ether, which was purified by column chromatography (eluent - heptanes

: EtOAc, 100 : 0 → 80 : 20 v/v%) to give the pure compound **[^{13}C]-14a** as a colorless liquid (yield 361 mg, >95%).[3]

The same procedure was used to synthesize the dideuterated compound **[D_2]-14a** with D_3Cl. However, the deprotection step of the acetal has to be done as quickly as possible (check conversion by TLC) under more dilute conditions (*ca.* 2 M), since hydrogen/deuterium exchange can take place.

Procedure F

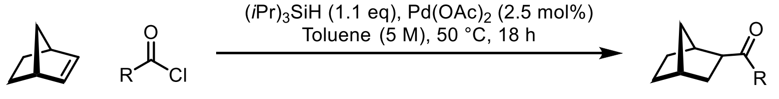

Scheme S 6

To a flame-dried Schlenk flask with a reflux condenser were added $Pd(OAc)_2$ (2.5 mol%) and norbornene (1.1 eq). The flask was evacuated and backfilled with argon 3 times, then toluene (5 M) was added to the flask. To the mixture, the acid chloride (1.0 eq) and *tri*isopropylsilane (1.1 eq) were added and the mixture was stirred at 50 °C for 18 h. After cooling to room temperature, all volatiles were removed under reduced pressure. The product was isolated by silica gel column chromatography (typical eluent - heptanes : EtOAc, 75 : 25 → 50 : 50 v/v%).[13]

Procedure G

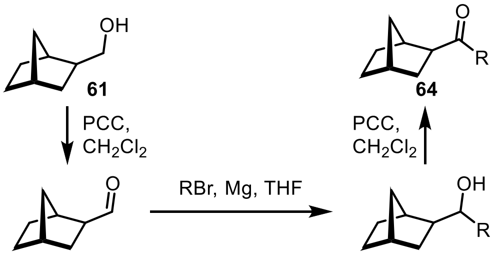

Scheme S 7

Pyridinium chlorochromate (PCC - 1.5 eq) was added to a mixture of norbornane-2-methanol (**61**, 1.0 eq), SiO_2 (20% w/w), $MgSO_4$ (20% w/w) in CH_2Cl_2 (0.1 M) and stirred for 12 h at 25 °C. The mixture was filtered over silica gel and then the volatiles were removed under reduced pressure to afford **62** in quantitative yield.

In a flame dried Schlenk, anhydrous THF (0.3 M) was added to Mg chips (3.0 eq). To the mixture, the corresponding aryl bromide (3.0 eq) was added and stirred until full consumption of the magnesium was observed. The Grignard reagent was then added to a solution of **62** in THF (0.1 M) at -78 °C and stirred for 1 h before the temperature was raised to 25 °C and the mixture stirred for an additional 1 h. The

reaction was quenched by the addition of an HCl aqueous solution (1 M). The organic layer was then diluted with EtOAc and washed with a saturated aqueous solution of $NaHCO_3$ and brine, dried over $MgSO_4$, filtered ad the volatiles removed under reduced pressure. The product **63** was isolated by column chromatography over silica gel. (typical eluent - heptanes : EtOAc, 20 : 80 → 50 : 50 v/v%).

Pyridinium chlorochromate (1.5 eq.) was added to a mixture of norbornane-2-methanol **63** (1.0 eq), SiO_2 (20% w/w), $MgSO_4$ (20% w/w) in CH_2Cl_2 (0.1 M) and stirred for 12 h at 25 °C. The mixture was filtered through silica gel and then the volatiles were removed under reduced pressure to afford **64** in quantitative yield.

Procedure H

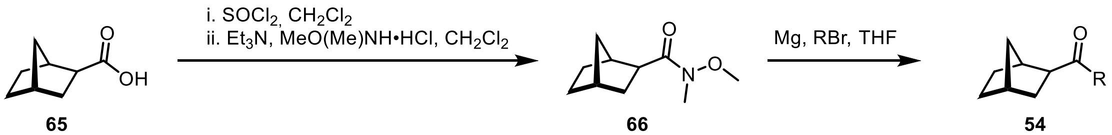

65 66 54

Scheme S 8

$SOCl_2$ (2.5 eq) was added to a solution of norbornane-2-methanol **65** (1.0 eq.), in CH_2Cl_2 (0.1 M) at 25 °C and stirred for 1 h. The volatiles were then removed under reduced pressure. The residue was dissolved in CH_2Cl_2 and slowly added to a solution of MeO(Me)NH•HCl (2.0 eq.) and Et_3N (4.0 eq.) in CH_2Cl_2 (0.3 M) at 0 °C. The solution was then stirred for 1.5 h at 25 °C. The reaction was discontinued by the addition of a saturated aqueous solution of $NaHCO_3$ and the organic phase was then washed with brine, dried over $MgSO_4$, filtered and then the volatiles removed under reduced pressure. The product **66** was isolated by column chromatography over silica gel (eluent - heptanes : EtOAc, 90 : 10 → 60 : 40 v/v%).

In a flame dried Schlenk flask, anhydrous THF (0.3 M) was added to Mg chips (3.0 eq.). To the mixture, the corresponding aryl bromide (3.0 eq.) was added and until most of the magnesium had dissolved. The Grignard reagent was then added to a solution of **66** in THF (0.1 M) at -78 °C and stirred for 1 h before the temperature was raised to 0 °C and the mixture stirred for an additional 1 h. The reaction was discontinued by the addition of an HCl aqueous solution (1 M). The organic layer was then diluted with EtOAc and washed with a saturated aqueous solution of $NaHCO_3$ and brine, dried over $MgSO_4$, filtered ad the volatiles removed under reduced pressure. The product **54** was isolated by column chromatography over silica gel (typical eluent - heptanes : EtOAc, 20 : 80 → 50 : 50 v/v%).[2]

Procedure J (Substrates 17c-17g)

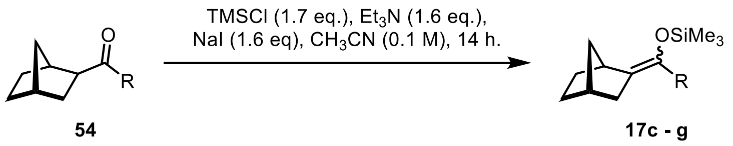

Scheme S 9

To a solution of ketone **54** (1.0 eq.) in CH_3CN (0.1 M) were added Et_3N (1.6 eq.), chlorotrimethylsilane (1.7 eq.) and sodium iodide (1.6 eq) in this order. The reaction mixture was stirred for 14 h at room temperature, then the mixture was diluted with pentane and the organic phase was washed with a saturated aqueous solution of $NaHCO_3$ and brine (100 ml), dried over anhydrous MgSO4, filtered and all the volatiles were removed under reduced pressure. The crude mixture was purified by flash column chromatography over silica gel eluent affording product **17a - h** (typical eluent - pentane : toluene, 67 : 33 → 50 : 50 v/v%).[14]

Procedure K (Substrates 17a-b, 17h)

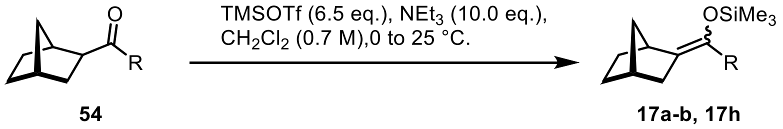

Trimethylsilyl trifluoromethanesulfonate (6.5 eq.) was added to a solution of the ketone (1.0 eq.) and Et_3N (10.0 eq.) in CH_2Cl_2 (0.7 M) at 0 °C. The mixture was stirred at 0 °C for 2.5 h and then at 24 °C for 3 h. The reaction was discontinued by addition of a saturated aqueous solution of $NaHCO_3$ solution. The resultant mixture was diluted with EtOAc to give a biphasic solution. The organic layer was collected, washed with H_2O and brine, dried over Na_2SO_4, and concentrated to crude. The product was isolated by flash column chromatography over silica gel (typical eluent - pentane : toluene, 67 : 33 → 50 : 50 v/v%).[14]

3) Experimental section

Procedure I (Products 16aa-mb and [D_2]-16aa/[^{13}C]-16aa)

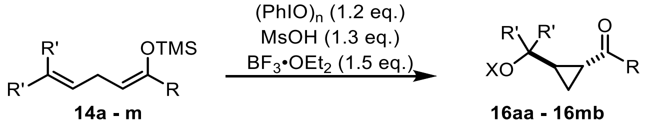

Scheme S 10

Iodosobenzene (1.2 eq., 0.24 mmol, 52.8 mg) was dispersed in CH_2Cl_2 (2 mL) and methanesulfonic acid was added (1.3 eq., 0.26 mmol, 16.9 µL), forming a clear yellow solution. The mixture was cooled to

-78 °C, resulting in a less clear solution (precipitation). BF_3•OEt_2 (1.5 eq., 0.30 mmol, 37 µL) was added and the solution became clear again with simultaneous intensifying of the yellow color. The mixture was stirred for 15 min at the same temperature before the silyl enol ether (1.0 eq., 2.0 mmol) was added quickly at once. Alternatively, the substrate can be added as a CH_2Cl_2 solution leading to the same results. The reaction changed color immediately (usually to brown, depending on the substrate) and a precipitate can be observed. After stirring for further 15 min, the flask was removed from the cooling-bath and reactive species were immediately quenched by the addition of H_2O (*ca.* 4 mL) and diluted with CH_2Cl_2 (*ca.* 4 mL). The mixture was transferred to a separatory funnel and the aqueous phase was extracted with CH_2Cl_2 (3x), and the combined organic layers were dried over Na_2SO_4 and filtered. After removal of the volatiles under reduced pressure, the crude material was purified by column chromatography (typical eluent - heptanes : EtOAc, 90 : 10 → 50 : 50 v/v%) to give the pure cyclopropane. In most cases, the product was eluted when the eluent was composed as follows: heptanes : EtOAc, 75 : 25 v/v%.

The same procedure was undertaken for the isotope labelling studies. The deuterated product afforded a 87 : 100 ratio of **[D_2]-16aa** : **[D_2]-16aa'**:

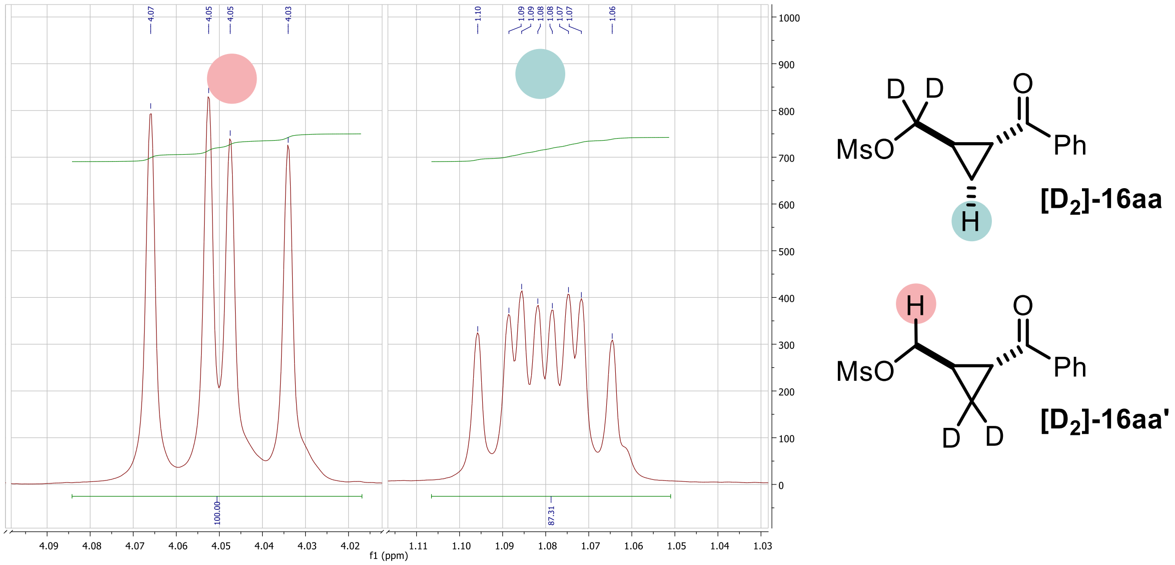

Figure S 1

The spectrum of **[13C]-16aa/[13C]-16aa** on the other hand showed only insignificant imbalances between the isomers (94 : 100):

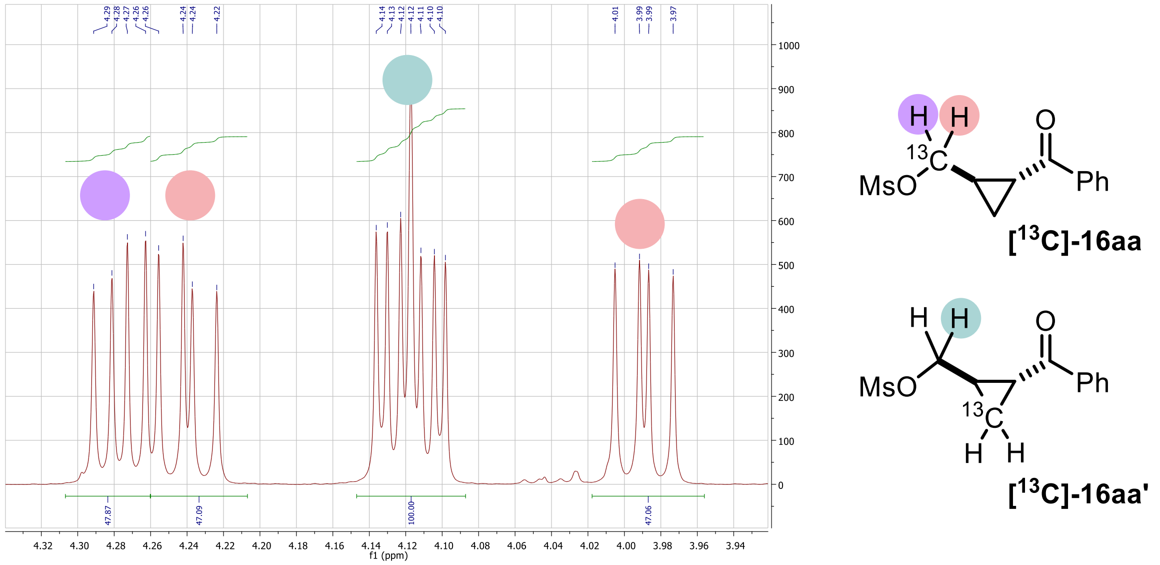

Figure S 2 – Please note: the second singal of the hydrogen marked in purple is further downfield.

Procedure II (Products 16ac-ai)

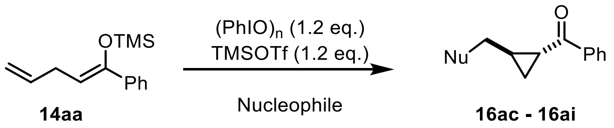

Scheme S 11

Iodosobenzene (1.2 eq., 0.24 mmol, 52.8 mg) was dispersed in CH_2Cl_2 (2 mL) and trimethylsilyl trifluoromethanesulfonate was added (1.2 eq., 0.24 mmol, 43 µL) at 0 °C, forming a clear yellow solution. The mixture was cooled to -78 °C. Then, the silyl enol ether (1.0 eq., 2.0 mmol, 46.5 mg) was added quickly at once. Alternatively, the substrate can be added as a CH_2Cl_2 solution leading to the same results. The reaction changed color immediately (usually to brown, depending on the substrate) and a precipitate can be observed. After stirring for 5 min, the nucleophile was added (see characterization section for more details) and the reaction was stirred for further 10 min at -78 °C. Then the reaction was warmed up to room temperature and stirred for an additional 10 min. Reactive species and excess of Lewis acid were quenched with H_2O (*ca.* 4 mL), the reaction mixture was diluted with CH_2Cl_2 (*ca.* 4 mL) and transferred to a separatory funnel. The aqueous phase was extracted with CH_2Cl_2 (3x), and the combined organic layer was dried over Na_2SO_4 and filtered. After removal of the volatiles under reduced pressure, the crude material was purified by column chromatography (typical eluent - heptanes : EtOAc, 90 : 10 → 50 : 50 v/v%) to give the pure cyclopropane.

Procedure III (Products 19a-h)

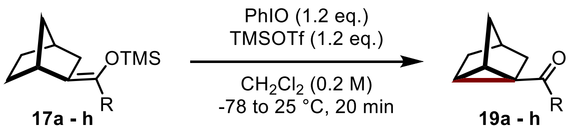

Scheme S 12

To a suspension of iodosobenzene (1.2 eq., 0.36 mmol) in CH_2Cl_2 (1.5 mL, 0.2 M) was added trimethylsilyl trifluoromethanesulfonate (1.2 eq., 0.36 mmol, 66.5 µL) at 0 °C under an argon atmosphere. Once all components had gone into solution, the mixture was cooled -78 °C and the corresponding silyl enol ether **17** (1.0 eq., 0.30 mmol) was added. The solution was stirred for 10 min at -78 °C and then 10 min at 25 °C. Reactive species and excess of Lewis acid were quenched by the addition of a saturated aqueous solution of $NaHCO_3$, diluted with EtOAc and the organic phase washed with a saturated aqueous solution of NaCl. The organic phase was then dried over anhydrous $MgSO_4$, filtered and all the volatiles removed under reduced pressure. The crude residue was purified by flash column chromatography on silica gel (typical eluent - heptanes : EtOAc, 83 : 17 → 50 : 50 v/v%) to afford the desired product **19a - h**.

Procedure IV (Products 30a-d, 31)

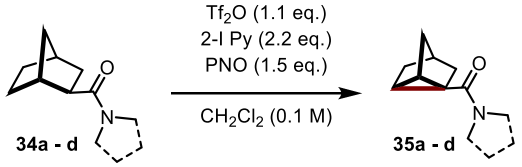

Scheme S 13

To a solution of amide **34a - d** (1.0 eq.0.25 mmol) and 2-iodopyridine (2.2 eq., 0.55 mmol) in CH_2Cl_2 (2 mL, 0.1 M) at 0 °C was added trifluoromethanesulfonic anhydride (1.1 eq., 0.27 mmol, 46.3 µL) and the resulting solution was stirred at 0 °C for 15 min. Pyridine *N*-oxide (1.5 eq., 0.37 mmol) was added in one portion and the reaction solution was stirred at 0 °C for 5 min. The solution was then allowed to warm up to ambient temperature (25 °C) over the course of 2 h, after which time a saturated aqueous solution of $NaHCO_3$ was added. The biphasic mixture was separated and the aqueous phase was extracted with CH_2Cl_2. The combined organic phases were dried over anhydrous $MgSO_4$, filtered and the filtrate was concentrated under reduced pressure. The crude residue was purified by flash column chromatography on silica gel (typical eluent - heptanes : EtOAc, 90 : 10 → 50 : 50 v/v%) to afford the desired product.

4) Characterization

(*Z*)-Trimethyl((1-phenylpenta-1,4-dien-1-yl)oxy)silane (**14a**)

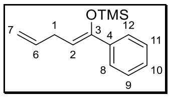

Synthesized by using the **General procedure A.** (*Z/E* > 20: 1).

Isolated yield: 1065 mg, 69% from the carboxylic acid. **^{1}H NMR** (600 MHz, $CDCl_3$) δ 7.56 – 7.45 (m, 2H, C8/C12), 7.32 (dd, *J* = 10.3, 4.7 Hz, 2H, C9/C11), 7.29 – 7.23 (m, 1H, C10), 5.91 (ddt, *J* = 16.6, 10.1, 6.1 Hz, 1H, C6), 5.28 (t, *J* = 7.3 Hz, 1H, C7), 5.13 (ddd, *J* = 17.1, 3.3, 1.6 Hz, 1H, C7), 5.07 – 4.99 (m, 1H, C2), 2.99 (td, *J* = 6.2, 1.4 Hz, 2H, C1), 0.15 (s, 9H, TMS). **^{13}C NMR** (151 MHz, $CDCl_3$) δ 149.89 (C3), 139.19 (C4), 137.33 (C6), 128.19 (C10), 127.72 (C9/11), 125.63 (C8/C12), 114.80 (C7), 108.55 (C2), 30.52 (C1), 0.70 (TMS). **HRMS** (ESI): m/z calculated for $[M+H]^+$ 233.1356: found: 233.1356. **ATR-FTIR** (cm^{-1}): 2960, 1647, 1493, 1446, 1334, 1302, 1279, 1251, 1117, 1074, 1040, 1020, 993, 948, 900, 869, 839, 754, 695, 635, 526 cm^{-1}.

(*Z*)-Trimethyl((1-phenylpenta-1,4-dien-1-yl-5-^{13}C)oxy)silane (**[^{13}C]-14a**)

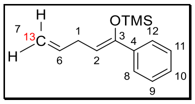

Synthesized by using the **General procedure E.** (*Z/E* > 20: 1).

Isolated yield: 361 mg, >95%. **^{1}H NMR** (600 MHz, $CDCl_3$) δ 7.56 – 7.46 (m, 2H, C8/C12), 7.32 (dd, *J* = 10.3, 4.7 Hz, 2H, C9/C11), 7.29 – 7.23 (m, 1H, C10), 5.91 (ddt, *J* = 16.6, 10.1, 6.1 Hz, 1H, C6), 5.29 – 4.97 (m, 2H, C7 ,C2), 5.03 (ddd, *J* = 157.6, 10.1, 1.3 Hz, 1H, C7), 2.99 (q, *J* = 6.5 Hz, 2H, C1), 0.15 (s, 9H, TMS). **^{13}C NMR** (151 MHz, CDC_{l3}) δ 128.20 (C10), 127.72 (C9/C11), 125.64 (C8/C12), 114.80 (C7!), 30.53 (C1), 0.70 (TMS). **HRMS** (ESI): m/z calculated for $[M+H]^+$ 234.1390: found: 234.1392.

(*Z*)-Trimethyl((1-phenylpenta-1,4-dien-1-yl-5,5-d_2)oxy)silane (**[D_2]-14a**)

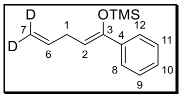

Synthesized by using the **General procedure E.** (*Z/E* > 20: 1).

Isolated yield: 363 mg, >95%. **^{1}H NMR** (400 MHz, $CDCl_3$) δ 7.51 – 7.43 (m, 2H), 7.33 – 7.27 (m, 2H), 7.26 – 7.22 (m, 1H), 5.93 – 5.81 (m, 1H), 5.26 (t, *J* = 7.3 Hz, 1H), 2.97 (t, *J* = 6.7 Hz, 2H), 0.13 (s, 9H). **^{13}C NMR** (101 MHz, $CDCl_3$) δ 149.90 (C3), 139.22 (C4), 137.13 (C6), 128.19 (C10), 127.71 (C9/C11), 125.64 (C8/C12), 108.57 (C2), 30.43 (C1), 0.70 (TMS). **HRMS** (ESI): m/z calculated for $[M+H]^+$ 235.1482: found: 235.1486.

(*Z*)-((1-(3,5-Dimethylphenyl)penta-1,4-dien-1-yl)oxy)trimethylsilane (**14d**)

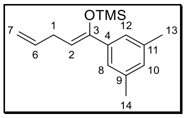

Synthesized by using the **General procedure A.** (*Z/E* > 20: 1).

Isolated yield: 496 mg, 62%. ^{1}**H NMR** (400 MHz, $CDCl_3$) δ 7.10 (s, 2H, C8/C12), 6.89 (s, 1H, C10), 5.88 (ddt, *J* = 16.3, 10.1, 6.2 Hz, 1H, C6), 5.23 (t, *J* = 6.9 Hz, 1H, C2), 5.22 – 4.96 (m, 2H, C7), 2.95 (ddt, *J* = 7.7, 6.2, 1.6 Hz, 2H, C1), 2.30 (s, 6H, C13/14), 0.14 (s, 9H, TMS). ^{13}**C NMR** (101 MHz, $CDCl_3$) δ 150.07 (C3), 139.05 (C9/C11), 137.55 (C4), 137.46 (C6), 129.36 (C10), 123.56 (C8/C12), 114.69 (C7), 108.13 (C2), 30.53 (C1), 21.48 (C13/C14), 0.73 (TMS). **HRMS** (ESI): m/z calculated for $[M+H]^+$ 261.1669: found: 261.1678. **ATR-FTIR** (cm^{-1}): 2959, 1649, 1601, 1330, 1251, 1222, 1190, 1117, 1090, 992, 962, 903, 885, 839, 753, 706, 650, 545, 534.

(*Z*)-((1-(4-Bromophenyl)penta-1,4-dien-1-yl)oxy)trimethylsilane (**14f**)

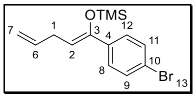

Synthesized by using the **General procedure A.** (*Z/E* > 20: 1).

Isolated yield: 602 mg, 62%. ^{1}**H NMR** (400 MHz, $CDCl_3$) δ 7.47 – 7.39 (m, 2H, C9/C11), 7.39 – 7.31 (m, 2H, C8/C12), 5.96 – 5.80 (m, 1H, C6), 5.25 (t, *J* = 7.3 Hz, 1H, C2), 5.18 – 4.96 (m, 2H, C7), 2.94 (ddt, *J* = 7.7, 6.2, 1.6 Hz, 2H, C1), 0.13 (s, 9H, TMS). ^{13}**C NMR** (101 MHz, $CDCl_3$) δ 148.95 (C3), 138.22 (C4), 136.99 (C6), 131.34 (C9/C11), 127.19 (C8/C12), 121.59 (C10), 115.01 (C7), 109.22 (C2), 30.53 (C1), 0.70 (TMS). **HRMS** (ESI): m/z calculated for $[M+H]^+$: 311.0461 found: 311.0450. **ATR-FTIR** (cm^{-1}): 3080, 2958, 1645, 1589, 1485, 1394, 1335, 1304, 1279, 1251, 1210, 1179, 1118, 1073, 1027, 1007, 993, 950, 910, 863, 840, 721.

(*Z*)-Trimethyl((1-(o-tolyl)penta-1,4-dien-1-yl)oxy)silane (**14k**)

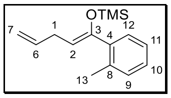

Synthesized by using the **General procedure A.** (*Z/E* = 5: 1).

Isolated yield: 328 mg, 28%. ^{1}**H NMR** (400 MHz, $CDCl_3$) δ 7.26 – 7.22 (m, 1H, C12), 7.22 – 7.12 (m, 3H, C9-11), 5.97 – 5.71 (m, 1H, C6), 5.21 – 4.97 (m, 2H, C7), 4.95 – 4.75 (m, 1H, C2), 2.96 (ddt, *J* = 7.7, 6.2, 1.6 Hz, 2H, C1), 2.37 (s, 3H, C13), -0.01 (s, 9H, TMS). ^{13}**C NMR** (101 MHz, $CDCl_3$) δ 150.50 (C3), 139.62 (C8), 137.72 (C6), 136.38 (C4), 130.32 (C9), 129.23 (C10), 127.90 (C11), 125.44 (C12), 114.40 (C7), 110.67 (C2), 30.04 (C1), 20.46 (C13), 0.38 (TMS). **HRMS** (ESI): m/z calculated for $[M+H]^+$: 247.1513 found: 247.1513. **ATR-FTIR** (cm^{-1}): 2960, 1657, 1639, 1326, 1300, 1251, 1195, 1121, 1076, 1022, 993, 952, 907, 873, 754, 728.

(*Z*)-((1-(4-Fluorophenyl)penta-1,4-dien-1-yl)oxy)trimethylsilane (**14e**)

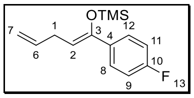

Synthesized by using the **General procedure A.** (*Z/E* > 20: 1).

Isolated yield: 485 mg, 77%. **^{1}H NMR** (400 MHz, $CDCl_3$) δ 7.50 – 7.39 (m, 2H, C8/C12), 7.05 – 6.90 (m, 2H, C9/C11), 5.88 (ddt, *J* = 16.3, 10.1, 6.2 Hz, 1H, C6), 5.18 (t, *J* = 7.3 Hz, 1H, C2), 5.16 – 4.88 (m, 2H, C7), 3.02 – 2.91 (m, 2H, C1), 0.13 (s, 9H, TMS). **^{13}C NMR** (101 MHz, $CDCl_3$) δ 162.53 (d, *J* = 247 Hz, C10), 149.05 (C3), 137.21 (C4), 135.43 (C6), 127.31 (d, *J* = 8 Hz, C8/C12), 115.06 (d, *J* = 22 Hz, C9/C11), 114.86 (C7), 108.36 (C2), 30.51 (C1), 0.68 (TMS). **HRMS** (ESI): m/z calculated for $[M+H]^+$: 251.1262 found: 251.1270. **ATR-FTIR** (cm^{-1}): 2960, 1650, 1604, 1506, 1409, 1336, 1300, 1279, 1252, 1225, 1157, 1118, 1082, 1027, 1013, 993, 951, 909, 871, 837, 753, 725, 688, 643, 578.

(*Z*)-Trimethyl((5-methyl-1-phenylhexa-1,4-dien-1-yl)oxy)silane (**14l**)

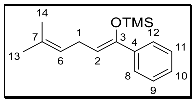

Synthesized by using the **General procedure A.** (*Z/E* > 20: 1).

Isolated yield: 411 mg, 47%. **^{1}H NMR** (400 MHz, $CDCl_3$) δ 7.50 – 7.45 (m, 2H, C8/C12), 7.32 – 7.26 (m, 2H, C9/C11), 7.26 – 7.21 (m, 1H, C10), 5.26 – 5.09 (m, 2H, C2/C6), 2.91 (t, *J* = 7.1 Hz, 2H, C1), 1.73 (d, *J* = 1.1 Hz, 3H, C13), 1.69 (s, 3H, C14), 0.15 (s, 9H, TMS). **^{13}C NMR** (101 MHz, $CDCl_3$) δ 148.81 (C3), 139.16 (C4), 131.99 (C7), 127.98 (C10), 127.35 (C9/C11), 125.38 (C8/C12), 122.78 (C6), 110.21 (C2), 25.67 (C13), 25.28 (C1), 17.80 (C14), 0.57 (TMS). **HRMS** (ESI): m/z calculated for $[M+H]^+$ 261.1669: found: 261.1673. **ATR-FTIR** (cm^{-1}): 2962, 1645, 1493, 1445, 1331, 1281, 1251, 1095, 1075, 1041, 1026, 877, 839, 752.

((5-Cyclohexyl-1-phenylhexa-1,4-dien-1-yl)oxy)trimethylsilane (**14m**)

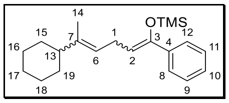

Synthesized by using the **General procedure D.** (Mixture of stereoisomers)

Isolated yield: 361 mg, 4%. **^{1}H NMR** (400 MHz, $CDCl_3$) δ 7.46 (ddd, *J* = 7.2, 3.2, 1.8 Hz, 2H, C8/C10), 7.29 (ddd, *J* = 7.5, 6.8, 1.5 Hz, 2H, C9/C11), 7.25 – 7.19 (m, 1H, C10), 5.25 – 5.09 (m, 2H, C2/C6), 2.97 – 2.84 (m, 2H, C1), 1.92 – 1.61 (m, 8H, Cy, C14), 1.52 – 1.08 (m, 6H ,Cy), 0.16 – 0.13 (m, 9H, TMS). **^{13}C NMR** (101 MHz, $CDCl_3$) δ 148.87 (C3), 141.24 (C4), 139.36 (C7), 128.13 (C10), 127.47 (C10), 125.53 (C9/C11), 125.51 (C8/C12), 122.25 (C6), 120.75 (C6'), 110.69 (C2), 110.66 (C2'), 47.45 (C13), 39.92 (C13'), 32.13 (C15/C19), 31.23 (C15/C19), 26.95 (C17), 26.85 (C16/C18), 26.60 (C16/C18), 26.46 (C16'/C18'), 25.21 (C1), 24.60 (C1'), 19.77 (C14), 14.69 (C14'), 0.71 (TMS). **HRMS** (ESI): m/z calculated for $[M+H]^+$: 329.2295 found: 329.2296.

ATR-FTIR (cm^{-1}): 2961, 2927, 2854, 2363, 2349, 2338, 2327, 1738, 1726, 1689, 1449, 1367, 1270, 1229, 1218, 774, 698, 550.

((1-Cyclohexylpenta-1,4-dien-1-yl)oxy)trimethylsilane (**14j**)

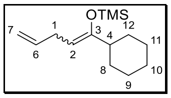

Synthesized by using the **Procedure B.** (*E/Z* ≈ 1: 1).

Isolated yield: 235 mg, 83%. **^{1}H NMR** (600 MHz, $CDCl_3$) δ 5.80 (ddtd, *J* = 16.0, 10.1, 6.0, 1.5 Hz, 1H, C6), 5.06 – 4.99 (m, 1H, C7), 4.96 – 4.92 (m, 1H, C7), 4.46 (t, *J* = 7.4 Hz, 1H, C2), 2.78 – 2.66 (m, 2H, C1), 2.73 – 2.21 (m, 2H, C1), 1.93 – 1.62 (m, 5H), 1.45 – 1.34 (m, 1H), 1.31 – 1.08 (m, 5H), 0.19 (s, 9H). **^{13}C NMR** (151 MHz, $CDCl_3$) δ 157.20 (C3), 156.05 (C3'), 138.49 (C6), 138.18 (C6'), 114.08 (C7), 113.93 (C7'), 103.14 (C2), 101.86 (C2'), 44.61 (C4), 39.23 (C4'), 31.39 (C1), 30.70 (C1'), 30.22 (C8/C12), 29.86 (C8'/C12'), 26.62 (C9/C11), 26.55 (C9'/C11'), 26.46 (C10), 26.17 (C10'), 0.85 (TMS), 0.68 (TMS'). **HRMS** (ESI): m/z calculated for $[M+H]^+$: 239.1826 found: 239.1829. **ATR-FTIR** (cm^{-1}): 2927, 2854, 1656, 1450, 1250, 1176, 1136, 1115, 1041, 1014, 992, 977, 930, 904, 838, 752, 687.

(*Z*)-((1-(4-Methoxyphenyl)penta-1,4-dien-1-yl)oxy)trimethylsilane (**14b**)

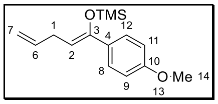

Synthesized by using the **General procedure A.** (*Z/E* = 17: 1).

Isolated yield: 504 mg, 83%. **^{1}H NMR** (400 MHz, $CDCl_3$) δ 7.45 – 7.28 (m, 2H, C8/C12), 6.97 – 6.77 (m, 2H, C9/C11), 5.88 (ddt, *J* = 16.3, 10.1, 6.2 Hz, 1H, C6), 5.18 – 4.95 (m, 3H, C2/C7), 3.81 (s, 3H, C14), 2.98 – 2.91 (m, 2H, C1), 0.13 (s, 9H, TMS). ^{13}C NMR (101 MHz, $CDCl_3$) δ 159.36 (C10), 149.64 (C3), 137.57 (C6), 131.96 (C4), 126.93 (C8/C12), 114.63 (C7), 113.55 (C9/C11), 106.92 (C2), 55.40 (C14), 30.52 (C1), 0.71 (TMS). **HRMS** (ESI): m/z calculated for $[M+H]^+$: 263.1462 found: 263.1463. **ATR-FTIR** (cm^{-1}): 2958, 16890, 1648, 1601, 1576, 1509, 1462, 1442, 1416, 1335, 1293, 1246, 1211, 1172, 1111, 1081, 1030, 1009, 994, 951, 911, 872, 835, 754, 688, 632, 588.

(*Z*)-Trimethyl((1-(4-(trifluoromethyl)phenyl)penta-1,4-dien-1-yl)oxy)silane (**14g**)

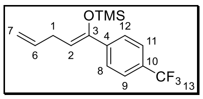

Synthesized by using the **General procedure A.** (*Z/E* > 20: 1).

Isolated yield: 574 mg, 68%. **^{1}H NMR** (400 MHz, $CDCl_3$) δ 7.62 – 7.54 (m, 4H, Ar), 5.88 (ddt, *J* = 16.3, 10.1, 6.2 Hz, 1H, C6), 5.37 (t, *J* = 7.3 Hz, 1H, C2), 5.17 – 5.01 (m, 2H, C7), 3.02 – 2.95 (m, 2H, C1), 0.14 (s, 9H,

TMS). **^{13}C NMR** (101 MHz, $CDCl_3$) δ 148.72 (C3), 142.67 (C4), 136.76 (C6), 129.64 (q, *J* = 32 Hz, C10), 125.68 (C8/C12), 125.26 (q, *J* = 4 Hz, C9/C11), 115.21 (C7), 110.80 (C2), 30.57 (C1), 0.69 (TMS). **HRMS** (ESI): m/z calculated for $[M+H]^+$: 301.1230 found: 301.1232. **ATR-FTIR** (cm^{-1}): 1646, 1617, 1410, 1322, 1282, 1253, 1165, 1123, 1109, 1067, 1014, 993, 951, 912, 869, 839, 753.

(*Z*)-Trimethyl((1-(thiophen-2-yl)penta-1,4-dien-1-yl)oxy)silane (**14h**)

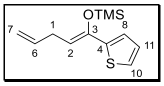

Synthesized by using the **General procedure A.** (*Z/E* = 10: 1).

Isolated yield: 430 mg, 59%. **^{1}H NMR** (400 MHz, $CDCl_3$) δ 7.13 (dd, *J* = 5.1, 1.2 Hz, 1H, C10), 7.06 (dd, *J* = 3.6, 1.2 Hz, 1H, C8), 6.94 (dd, *J* = 5.1, 3.6 Hz, 1H, C11), 5.93 – 5.81 (m, 1H, C6), 5.26 (t, *J* = 7.4 Hz, 1H, C2), 5.15 – 4.99 (m, 2H, C7), 2.93 (ddt, *J* = 7.6, 6.2, 1.6 Hz, 2H, C1), 0.22 (s, 9H, TMS). ^{13}C NMR (101 MHz, $CDCl_3$) δ 144.73 (C3), 143.53 (C4), 136.91 (C6), 127.19 (C10), 124.13 (C11), 123.32 (C8), 115.05 (C7), 107.85 (C2), 30.38 (C1), 0.70 (TMS). **HRMS** (ESI): m/z calculated for $[M+H]^+$: 239.0920 found: 239.0918. **ATR-FTIR** (cm^{-1}): 2960, 1643, 1434, 1414, 1359, 1338, 1296, 1251, 1224, 1199, 1112, 1085, 1073, 1018, 992, 911, 842, 754.

(*Z*)-((1-(Benzo[d][1,3]dioxol-5-yl)penta-1,4-dien-1-yl)oxy)trimethylsilane (**14c**)

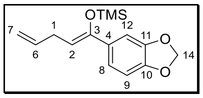

Synthesized by using the **General procedure A.** (*Z/E* = 15: 1).

Isolated yield: 529 mg, 71%.**^{1}H NMR** (400 MHz, $CDCl_3$) δ 7.00 – 6.94 (m, 2H, C12, C9), 6.76 – 6.72 (m, 1H, C8), 5.95 (s, 2H, C14), 5.87 (ddt, *J* = 16.3, 10.1, 6.1 Hz, 1H, C6), 5.15 – 4.97 (m, 3H, C2/C7), 3.03 – 2.88 (m, 2H, C1), 0.13 (s, 9H, TMS). **^{13}C NMR** (101 MHz, $CDCl_3$) δ 149.55 (C10), 147.60 (C11), 147.28 (C3), 137.40 (C6), 133.78 (C4), 119.42 (C8), 114.72 (C7), 107.96 (C12), 107.46 (C9), 106.33 (C2), 101.17 (C14), 30.51 (C1), 0.70 (TMS). **HRMS** (ESI): m/z calculated for $[M+H]^+$: 277.1254 found: 277.1254. **ATR-FTIR** (cm^{-1}): 1649, 1503, 1487, 1438, 1357, 1318, 1295, 1284, 1248, 1230, 1144, 1101, 1073, 1039, 994, 960, 938, 910, 891, 806, 753, 727, 689, 638, 571, 552.

(*Z*)-((2,2-Dimethylhepta-3,6-dien-3-yl)oxy)trimethylsilane (**14i**)

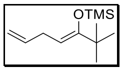

Synthesized by using the **Procedure C.** (*Z/E* > 20: 1).

Isolated yield: 385 mg, 37%.

Spectroscopic properties match with the literature.[16]

((1*S**,2*S**)-2-Benzoylcyclopropyl)methyl methanesulfonate (**16aa**)

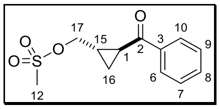 Synthesized by using the **General procedure I.**

Isolated yield: 36.2 mg, 71%. **^{1}H NMR** (600 MHz, $CDCl_3$) δ 8.05 – 7.98 (m, 2H, C6/C10), 7.63 – 7.56 (m, 1H, C8), 7.55 – 7.45 (m, 2H, C7/C9), 4.40 (dd, *J* = 11.1, 6.0 Hz, 1H, C17), 4.12 (dd, *J* = 11.1, 8.1 Hz, 1H, C17), 3.02 (s, 3H, C12), 2.84 – 2.75 (m, 1H, C1), 2.11 – 1.99 (m, 1H, C15), 1.56 (dt, *J* = 9.1, 4.7 Hz, 1H, C16), 1.19 – 1.10 (m, 1H, C16). **^{13}C NMR** (151 MHz, $CDCl_3$) δ 198.19 (C2), 137.41 (C3), 133.36 (C8), 128.79 (C6/C10), 128.30 (C7/C9), 71.77 (C17), 38.15 (C12), 23.29 (C1), 23.23 (C15), 15.89 (C16). **HRMS** (ESI): m/z calculated for $[M+Na]^+$ 277.0505 found: 277.0510. **ATR-FTIR** (cm^{-1}): 1725, 1667, 1598, 1580, 1451, 1414, 1352, 1332, 1264, 1224, 1172, 1061, 1039, 1025, 974, 948, 816, 786, 746.

((1*S**,2*S**)-2-Benzoylcyclopropyl)methyl-d$_2$ methanesulfonate (**[D$_2$]-16aa**)

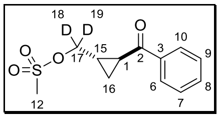 Synthesized by using the **General procedure I.**

Isolated yield: 29.8 mg, 72%. **^{1}H NMR** (600 MHz, $CDCl_3$) δ 8.06 – 7.99 (m, 2H, C6/C10), 7.59 (t, *J* = 7.4 Hz, 1H, C8), 7.49 (t, *J* = 7.8 Hz, 2H, C7/C9), 3.02 (s, 3H, C12), 2.83 – 2.77 (m, 1H, C1), 2.09 – 2.02 (m, 1H, C15), 1.58 – 1.54 (m, 1H, C16), 1.18 – 1.12 (m, 1H, C16). **^{13}C NMR** (151 MHz, $CDCl_3$) δ 198.22 (C2), 137.43 (C3), 133.37 (C8), 128.81 (C6/C10), 128.31 (C7/C9), 38.77 – 37.66 (m, C1), 23.79 – 22.82 (m, C15), 15.88 (C16). **HRMS** (ESI): m/z calculated for $[M+Na]^+$ 279.0631 found: 279.0632. **ATR-FTIR** (cm^{-1}): 1739, 1668, 1598, 1451, 1410, 1354, 1335, 1225, 1174, 1041, 1018, 973, 943, 828, 703, 527.

((1*S**,2*S**)-2-Benzoylcyclopropyl-3,3-d$_2$)methyl methanesulfonate (**[D$_2$]-16aa'**)

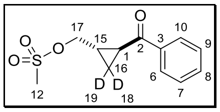

^{1}H NMR (600 MHz, $CDCl_3$) δ 8.06 – 7.99 (m, 2H, C6/C10), 7.59 (t, *J* = 7.4 Hz, 1H, C8), 7.49 (t, *J* = 7.8 Hz, 2H, C7/C9), 4.41 (dd, *J* = 11.1, 6.0 Hz, 1H, C8), 4.12 (dd, *J* = 10.8, 7.8 Hz, 1H, C17), 3.02 (s, 3H, C12), 2.83 – 2.77 (m, 1H, C1), 2.09 – 2.02 (m, 1H, C15). **^{13}C NMR** (151 MHz, $CDCl_3$) δ 198.22 (C2), 137.43 (C3), 133.37 (C8), 128.81 (C6/C10), 128.31 (C7/C9), 71.72 (C17), 38.77 – 37.66 (m, C1), 23.79 – 22.82 (m, C15).

((1*S**,2*S**)-2-Benzoylcyclopropyl)methyl-^{13}C methanesulfonate (**[^{13}C]-16aa**)

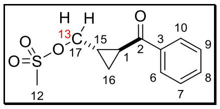

Synthesized by using the **General procedure I.**

Isolated yield: 34.8 mg, 68%. **^{1}H NMR** (600 MHz, $CDCl_3$) δ 8.06 – 8.00 (m, 2H, C6/C10), 7.62 – 7.55 (m, 1H, C8), 7.51 – 7.46 (m, 2H, C7/C9), 4.40 (ddd, *J* = 150.9, 11.1, 6.0 Hz, 1H, C17), 4.11 (ddd, *J* = 150.3, 11.1, 8.1 Hz, 1H, C17), 3.02 (s, 3H, C12), 2.83 – 2.77 (m, 1H, C1), 2.10 – 2.03 (m, 1H, C15), 1.59 – 1.54 (m, 1H, C16), 1.17 – 1.11 (m, 1H, C16). **^{13}C NMR** (151 MHz, $CDCl_3$) δ 198.22 (C2), 137.42 (C3), 133.37 (C8), 128.80 (C6/C10), 128.31 (C7/C9), 71.76 (C17!), 38.17 (C12), 34.12 (C1), 29.8 (C15), 15.91 (C16). **HRMS** (ESI): m/z calculated for $[M+Na]^+$ 278.0539 found: 278.0540. **ATR-FTIR** (cm^{-1}): 1666, 1597, 1580, 1451, 1412, 1349, 1329, 1263, 1222, 1170, 1107, 1057, 1037, 1023, 972, 931, 911, 809, 785, 757.

((1*S**,2*S**)-2-Benzoylcyclopropyl-3-^{13}C)methyl methanesulfonate (**[^{13}C]-16aa’**)

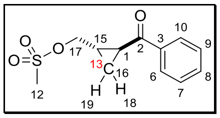

^{1}H NMR (600 MHz, $CDCl_3$) δ 8.06 – 8.00 (m, 2H, C6/C10), 7.62 – 7.55 (m, 1H, C8), 7.51 – 7.46 (m, 2H, C7/C9), 4.41 (ddd, *J* = 11.1, 6.0, 1.5 Hz, 1H, C17), 4.12 (ddd, *J* = 11.4, 8.1, 3.6 Hz, 1H, C17), 3.02 (s, 3H, C12), 2.83 – 2.77 (m, 1H, C1), 2.10 – 2.03 (m, 1H, C15), 1.56 (d, *J* = 168.5, Hz, 1H, C16), 1.15 (d, J = 163.8, Hz, 1H, C16). **^{13}C NMR** (151 MHz, $CDCl_3$) δ 198.22 (C2), 137.42 (C3), 133.37 (C8), 128.80 (C6/C10), 128.31 (C7/C9), 71.76 (C17), 38.17 (C12), 34.12 (C1), 29.8 (C15), 15.91 (C16!).

((1*S**,2*S**)-2-(3,5-Dimethylbenzoyl)cyclopropyl)methyl methanesulfonate (**16ba**)

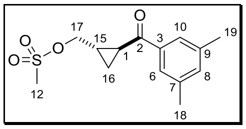

Synthesized by using the **General procedure I.**

Isolated yield: 38.1 mg, 68%.**^{1}H NMR** (600 MHz, $CDCl_3$) δ 7.62 (s, 2H, C6/C10), 7.21 (s, 1H, C8), 4.39 (dd, *J* = 11.0, 6.1 Hz, 1H, C17), 4.12 (dd, *J* = 11.0, 8.0 Hz, 1H, C17), 3.02 (s, 3H, C12), 2.79 – 2.73 (m, 1H, C1), 2.38 (s, 6H, C18/C19), 2.09 – 2.00 (m, 1H, C15), 1.58 – 1.50 (m, 1H, C16), 1.16 – 1.10 (m, 1H, C16). **^{13}C NMR** (151 MHz, $CDCl_3$) δ 198.52 (C2), 138.45 (C7/9), 137.55 (C3), 134.99 (C8), 126.11 (C6/C10), 71.92 (C17), 38.17 (C12), 23.41 (C1), 23.00 (C15), 21.36 (C18/C19), 15.81 (C16). **HRMS** (ESI): m/z calculated for $[M+Na]^+$ 305.0818 found: 305.0824. **ATR-FTIR** (cm^{-1}): 1666, 1604, 1450, 1415, 1351, 1330, 1292, 1207, 1172, 1077, 1047, 972, 930, 864, 840, 816, 738, 711, 678.

((1*S**,2*S**)-2-(Benzo[*d*][1,3]dioxole-5-carbonyl)cyclopropyl)methyl methanesulfonate (**16ca**)

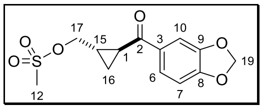

Synthesized by using the **General procedure I.**

Isolated yield: 33.3 mg, 56%.^{1}**H NMR** (400 MHz, $CDCl_3$) δ 7.68 – 7.63 (m, 1H, C6), 7.47 (d, *J* = 1.7 Hz, 1H, C10), 6.88 (d, *J* = 8.2 Hz, 1H, C7), 6.04 (s, 2H, C19), 4.40 (dd, *J* = 11.1, 6.0 Hz, 1H, C17), 4.10 (dd, *J* = 11.1, 8.1 Hz, 1H, C17), 3.02 (s, 3H, C12), 2.72 – 2.64 (m, 1H, C1), 2.06 – 1.95 (m, 1H, C15), 1.52 (ddd, *J* = 9.0, 4.9, 4.4 Hz, 1H, C16), 1.10 (ddd, *J* = 8.4, 6.1, 4.3 Hz, 1H, C16). ^{13}**C NMR** (101 MHz, $CDCl_3$) δ 196.04 (C2), 152.10 (C8), 148.41 (C9), 132.30 (C3), 124.72 (C6), 108.10 (C7/C10), 102.05 (C19), 71.86 (C17), 38.21 (C12), 23.11 (C1), 22.94 (C15), 15.56 (C1). **HRMS** (ESI) m/z calculated for $[M+Na]^+$ 321.0403 found 321.0412. **ATR-FTIR** (cm^{-1}): 1658, 1603, 1504, 1490, 1440, 1415, 1349, 1246, 1170, 1140, 1113, 1099, 1032, 973, 923, 883, 807, 762, 732, 647, 574.

((1*S**,2*S**)-2-(4-Methoxybenzoyl)cyclopropyl)methyl methanesulfonate (**16da**)

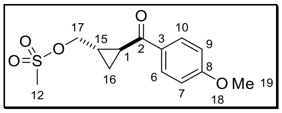

Synthesized by using the **General procedure I.**

Isolated yield: 30.1 mg, 53%.^{1}**H NMR** (400 MHz, $CDCl_3$) δ 8.08 – 7.90 (m, 2H, C6/C10), 7.00 – 6.92 (m, 2H, C7/C9), 4.39 (dd, *J* = 11.1, 6.0 Hz, 1H, C17), 4.11 (dd, *J* = 11.1, 8.1 Hz, 1H, C17), 3.87 (s, 3H, C19), 3.01 (s, 3H, C12), 2.80 – 2.70 (m, 1H, C1), 2.07 – 1.97 (m, 1H, C15), 1.57 – 1.50 (m, 1H, C16), 1.14 – 1.04 (m, 1H, C16). ^{13}**C NMR** (101 MHz, $CDCl_3$) δ 196.46 (C2), 163.82 (C8), 130.60 (C6/C10), 130.47 (C3), 113.97 (C7), 113.86 (C9), 71.99 (C17), 55.63 (C19), 38.20 (C12), 22.91 (C1), 22.81 (C15), 15.47 (C16). **HRMS** (ESI) m/z calculated for $[M+Na]^+$ 307.0611 found 307.0611. **ATR-FTIR** (cm^{-1}): 1658, 1598, 1574, 1511, 1461, 1422, 1351, 1331, 1257, 1227, 1167, 1120, 1061, 1025, 793, 943, 913, 840, 809, 750, 692, 667, 632, 565, 526.

((1*S**,2*S**)-2-(4-Fluorobenzoyl)cyclopropyl)methyl methanesulfonate (**16ea**)

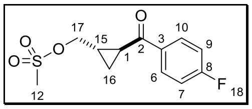

Synthesized by using the **General procedure I.**

Isolated yield: 35.6 mg, 67%. ^{1}**H NMR** (600 MHz, $CDCl_3$) δ 8.10 – 7.99 (m, 2H, C6/C10), 7.21 – 7.11 (m, 2H, C7/C9), 4.42 (dd, *J* = 11.1, 5.8 Hz, 1H, C17), 4.09 (dd, *J* = 11.1, 8.2 Hz, 1H, C17), 3.02 (s, 3H, C12), 2.79 – 2.71 (m, 1H, C1), 2.10 – 1.97 (m, 1H, C15), 1.56 (dt, *J* = 9.1, 4.7 Hz, 1H, C16), 1.14 (ddd, *J* = 20.0, 11.1, 7.8

Hz, 1H, C16). **^{13}C NMR** (151 MHz, $CDCl_3$) δ 196.62 (C2), 166.00 (d, *J* = 255 Hz, C8), 133.84 (d, *J* = 2.9 Hz, C3), 130.97 (d, *J* = 9 Hz, C6/C10), 115.90 (d, *J* = 22 Hz, C7/C9), 71.68 (C17), 38.15 (C12), 23.38 (C1), 23.26 (C15), 15.83 (C16). **HRMS** (ESI): m/z calculated for $[M+Na]^+$ 295.0411 found: 295.0416. **ATR-FTIR** (cm^{-1}): 1667, 1597, 1507, 1417, 1350, 1329, 1265, 1221, 1171, 1157, 1111, 1061, 1036, 1013, 973, 943, 915, 845, 808, 758, 739.

((1*S**,2*S**)-2-(4-Bromobenzoyl)cyclopropyl)methyl methanesulfonate (**16fa**)

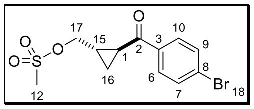

Synthesized by using the **General procedure I.**

Isolated yield: 33.5 mg, 50%. **^{1}H NMR** (600 MHz, $CDCl_3$) δ 7.89 (d, *J* = 8.5 Hz, 2H, C6/C10), 7.63 (d, *J* = 8.5 Hz, 2H, C7/C9), 4.42 (dd, *J* = 11.2, 5.8 Hz, 1H, C17), 4.09 (dd, *J* = 11.2, 8.2 Hz, 1H, C17), 3.02 (s, 3H, C12), 2.79 – 2.71 (m, 1H, C1), 2.17 – 2.00 (m, 1H, C15), 1.57 (dt, *J* = 9.0, 4.7 Hz, 1H, C16), 1.19 – 1.10 (m, 1H, C16). **^{13}C NMR** (151 MHz, $CDCl_3$) δ 197.21 (C2), 136.12 (C3), 132.11 (C7/C9), 129.85 (C6/C10), 128.59 (C8), 71.58 (C17), 38.17 (C12), 23.60 (C1), 23.33 (C15), 16.00 (C16). **HRMS** (ESI): m/z calculated for $[M+Na]^+$ 354.9610 found: 354.9610. **ATR-FTIR** (cm^{-1}): 1669, 1585, 1415, 1397, 1352, 1267, 1221, 1173, 1070, 1038, 1009, 974, 948, 837, 812, 735.

((1*S**,2*S**)-2-(4-(Trifluoromethyl)benzoyl)cyclopropyl)methyl methanesulfonate (**16ga**)

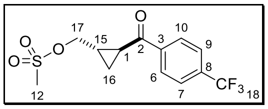

Synthesized by using the **General procedure I.**

Isolated yield: 43.3 mg, 67%.**^{1}H NMR** (600 MHz, $CDCl_3$) δ 8.12 (d, *J* = 8.1 Hz, 2H, C6/10), 7.75 (d, *J* = 8.2 Hz, 2H, C7/9), 4.46 (ddd, *J* = 16.9, 9.7, 4.8 Hz, 1H, C17), 4.09 (dd, *J* = 11.2, 8.3 Hz, 1H, C17), 3.02 (s, 3H, C12), 2.80 (dt, *J* = 8.7, 4.5 Hz, 1H, C1), 2.16 – 2.01 (m, 1H, C15), 1.66 – 1.50 (m, 1H, C16), 1.27 – 1.17 (m, 1H, C16). **^{13}C NMR** (151 MHz, $CDCl_3$) δ 197.45 (C2), 140.08 (C3), 134.56 (q, J = 33 Hz, C8), 128.66 (C6/C10), 125.84 (q, *J* = 4 Hz, C7/9), 123.70 (q, *J* = 273 Hz, C18), 71.46 (C17), 38.10 (12), 24.01 (C1), 23.73 (C15), 16.24 (C16). **HRMS** (ESI) m/z calculated for $[M+Na]^+$ 345.0379 found 345.0379. **ATR-FTIR** (cm^{-1}): 1675, 1513, 1415, 1353, 1319, 1268, 1222, 1167, 1124, 1065, 1040, 1015, 973, 946, 914, 851, 833, 811, 734, 698, 674, 649, 592.

((1*S**,2*S**)-2-(Thiophene-2-carbonyl)cyclopropyl)methyl methanesulfonate (**16ha**)

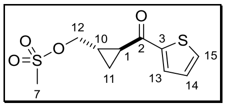 Synthesized by using the **General procedure I.**

Isolated yield: 31.5 mg, 61%.^{1}**H NMR** (400 MHz, $CDCl_3$) δ 7.87 (dd, *J* = 3.8, 1.1 Hz, 1H, C13), 7.66 (td, *J* = 4.6, 1.2 Hz, 1H, C15), 7.17 (dd, *J* = 4.9, 3.8 Hz, 1H, C14), 4.38 (dd, *J* = 11.1, 6.0 Hz, 1H, C12), 4.10 (dd, *J* = 11.1, 8.0 Hz, 1H, C12), 3.02 (s, 3H, C7), 2.68 – 2.60 (m, 1H, C1), 2.12 – 1.99 (m, 1H, C10), 1.57 (dt, *J* = 9.1, 4.7 Hz, 1H, C11), 1.18 – 1.08 (m, 1H, C11). ^{13}**C NMR** (151 MHz, $CDCl_3$) δ 190.50 (C2), 144.46 (C3), 134.17 (C13), 132.43 (C15), 128.48 (C14), 71.58 (C12), 38.18 (C7), 24.16 (C1), 22.95 (C10), 15.50 (C11). **HRMS** (ESI) m/z calculated for $[M+Na]^+$ 283.0069 found 283.0070. **ATR-FTIR** (cm^{-1}): 1643, 1518, 1446, 1417, 1347, 1328, 1262, 1238, 1227, 1169, 1082, 1065, 1018, 971, 939, 895, 844, 808, 721.

((1*S**,2*S**)-2-Pivaloylcyclopropyl)methyl methanesulfonate (**16ia**)

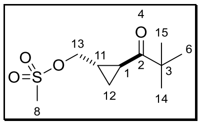 Synthesized by using the **General procedure I.**

Isolated yield: 22.1 mg, 53%.^{1}**H NMR** (400 MHz, $CDCl_3$) δ 4.31 (dd, J = 11.0, 6.1 Hz, 1H, C13), 3.98 (dd, J = 11.0, 8.2 Hz, 1H, C13), 3.01 (s, 3H, C8), 2.28 – 2.18 (m, 1H, C1), 1.83 – 1.68 (m, 1H, C11), 1.35 – 1.26 (m, 1H, C12), 1.20 (s, 9H, C6/C14/C15), 1.00 – 0.87 (m, 1H, C12). ^{13}**C NMR** (101 MHz, $CDCl_3$) δ 213.13 (C2), 71.92 (C13), 38.06 (C8), 26.27 (C6/C14/C15), 22.65 (C1), 21.95 (C11), 15.35 (C12). **HRMS** (ESI) m/z calculated for $[M+Na]^+$ 227.1048 found 227.1045. **ATR-FTIR** (cm^{-1}): 2970, 1690, 1479, 1415, 1396, 1353, 1201, 1174, 1092, 1044, 945, 951, 929, 812, 784.

((1*S**,2*S**)-2-(Cyclohexanecarbonyl)cyclopropyl)methyl methanesulfonate (**16ja**)

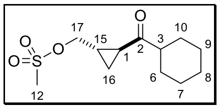 Synthesized by using the **General procedure I.**

Isolated yield: 30.0 mg, 58%.^{1}**H NMR** (400 MHz, $CDCl_3$) δ 4.27 (dd, *J* = 11.0, 6.2 Hz, 1H, C17), 3.99 (dd, *J* = 11.0, 8.0 Hz, 1H, C17), 3.01 (s, 3H, C12), 2.56 – 2.44 (m, 1H, C1), 2.12 – 2.04 (m, 1H, C3), 1.95 – 1.87 (m, 2H, C6/C10), 1.84 – 1.73 (m, 3H, C6/C10/C16), 1.71 – 1.63 (m, 1H, C15), 1.43 – 1.17 (m, 6H, Cy), 0.93 (ddd, *J* = 8.4, 6.0, 4.3 Hz, 1H, C16). ^{13}**C NMR** (101 MHz, $CDCl_3$) δ 211.37 (C2), 71.87 (C17), 51.72 (C3), 38.07 (C12), 28.40 (C6), 28.39 (C10), 26.00 (C8), 25.71 (C7/9), 24.93 (C1), 22.30 (C15), 15.14 (C16). **HRMS** (ESI) m/z

calculated for $[M+Na]^+$ 283.0975 found 283.0980. **ATR-FTIR** (cm^{-1}): 3062, 3005, 2958, 1665, 1597, 1580, 1449, 1396, 1333, 1309, 1225, 1174, 1070, 1017, 913, 867, 799, 775, 753, 701, 671, 664, 654, 573, 546.

((1*S**,2*S**)-2-(2-Methylbenzoyl)cyclopropyl)methyl methanesulfonate (**16ka**)

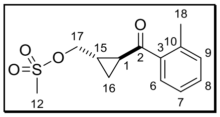

Synthesized by using the **General procedure I.**

Isolated yield: 38.9 mg, 73%.**^{1}H NMR** (400 MHz, $CDCl_3$) δ 7.76 (dd, *J* = 7.7, 1.2 Hz, 1H, C6), 7.42 – 7.36 (m, 1H, C8), 7.32 – 7.23 (m, 2H, C7/C9), 4.36 (dd, *J* = 11.0, 6.2 Hz, 1H, C17), 4.10 (dd, *J* = 11.0, 7.9 Hz, 1H, C17), 3.02 (s, 3H, C12), 2.59 – 2.51 (m, 1H, C1), 2.48 (s, 3H, C18), 2.12 – 1.99 (m, 1H, C15), 1.55 (ddd, *J* = 9.0, 4.9, 4.4 Hz, 1H, C16), 1.13 (ddd, *J* = 8.4, 6.1, 4.3 Hz, 1H, C16). **^{13}C NMR** (151 MHz, $CDCl_3$) δ 202.03 (C2), 138.69 (C10), 137.59 (C3), 131.83 (C8), 131.50 (C9), 128.78 (C6), 125.91 (C7), 71.76 (C17), 38.09 (C12), 26.46 (C18), 23.33 (C1), 21.01 (C15), 16.20 (C16). **HRMS** (ESI) m/z calculated for $[M+Na]^+$ 291.0662 found 291.0660. **ATR-FTIR** (cm^{-1}): 1669, 1456, 1413, 1350, 1288, 1260, 1219, 1171, 1060, 1031, 972, 943, 917, 810, 731, 649, 563, 527.

((1*S**,2*S**)-2-(2-Hydroxypropan-2-yl)cyclopropyl)(phenyl)methanone (**16lb**)

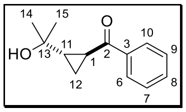

Synthesized by using the **General procedure I.**

Isolated yield: 38.5 mg, 94%.**^{1}H NMR** (600 MHz, $CDCl_3$) δ 8.05 – 8.02 (m, 2H, C6/C10), 7.58 – 7.52 (m, 1H, C8), 7.46 (dt, *J* = 7.5, 4.2 Hz, 2H, C7/C9), 2.79 (dt, *J* = 8.3, 4.6 Hz, 1H, C1), 1.77 (ddd, *J* = 9.1, 6.8, 4.4 Hz, 1H, C11), 1.41 – 1.36 (m, 1H, C12), 1.32 (s, 3H, C14/C15), 1.31 (s, 4H, C14/C15/OH), 1.20 (ddd, *J* = 8.2, 6.8, 3.6 Hz, 1H, C12). **^{13}C NMR** (151 MHz, $CDCl_3$) δ 200.35 (C2), 137.95 (C3), 132.90 (C8), 128.63 (C6/C10), 128.21 (C7/C9), 68.82 (C13), 36.92 (C1), 29.90 (C14/C15), 29.61 (C14/C15), 21.14 (C11), 14.54 (C12). **HRMS** (ESI) m/z calculated for $[M+Na]^+$ 227.1048 found 227.1045. **ATR-FTIR** (cm^{-1}): 3429, 2970, 1719, 1655, 1597, 1580, 1450, 1398, 1371, 1331, 1221, 1180, 1158, 1115, 1072, 1052, 1037, 1012, 1002, 954, 913, 873, 839, 794, 756, 729, 713, 693, 652.

((1*S**,2*S**)-2-(1-Cyclohexyl-1-hydroxyethyl)cyclopropyl)(phenyl)methanone (**16mb**)

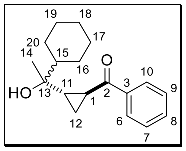

Synthesized by using the **General procedure I.**

Isolated yield: 46.5 mg, 85% (d.r. = 1 : 1).^{1}**H NMR** (400 MHz, $CDCl_3$) δ 8.08 – 7.94 (m, 2H, C6/C10), 7.59 – 7.53 (m, 1H, C8), 7.50 – 7.43 (m, 2H, C7/C9), 2.88 – 2.72 (m, 1H, C1), 1.92 – 1.59 (m, 6H, C11/Cy), 1.46 – 1.33 (m, 2H, Cy), 1.29 – 1.18 (m, 6H, C14, Cy), 1.16 – 0.96 (m, 4H, C12/Cy). ^{13}**C NMR** (101 MHz, $CDCl_3$) δ 200.81 (C2), 200.46 (C2'), 138.09 (C3), 132.86 (C8), 132.85 (C8'), 128.63 (C6/C10), 128.24 (C7/C9), 128.20 (C7'/C9'), 72.69 (C13), 72.41 (C13'), 49.51 (C15), 49.47 (C15'), 34.50 (C1), 33.95 (C1'), 27.80 (Cy), 27.64 (Cy), 27.61 (Cy), 27.55 (Cy), 26.80 (Cy), 26.78 (Cy), 26.77 (Cy), 26.76 (Cy), 26.60 (Cy), 26.56 (Cy), 25.68 (C14), 24.57 (C14'), 21.19 (C12), 20.37 (C12'), 15.44, 14.34. **HRMS** (ESI) m/z calculated for $[M+Na]^+$ 295.1669 found 295.1670. **ATR-FTIR** (cm^{-1}): 3462, 2926, 2852, 1653, 1598, 1580, 1450, 1397, 1374, 1332, 1308, 1221, 1179, 1160, 1114, 1073, 1053, 1034, 1026, 1008, 935, 915, 893, 872, 857, 843, 731, 702, 671, 655, 546.

((1*S**,2*S**)-2-(Fluoromethyl)cyclopropyl)(phenyl)methanone (**16ac**)

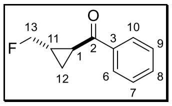

Synthesized by using the **General procedure II.** Nucleophile: $Bu_4NPh_3SiF_2$ (1.5. eq.).

Isolated yield: 24.3 mg, 68%.^{1}**H NMR** (600 MHz, $CDCl_3$) δ 8.03 – 8.00 (m, 2H, C6/C10), 7.60 – 7.55 (m, 1H, C8), 7.52 – 7.45 (m, 2H, C7/C9), 4.61 (ddd, *J* = 48.4, 9.9, 5.5 Hz, 1H, C13), 4.27 (ddd, *J* = 47.6, 9.9, 7.4 Hz, 1H, C13), 2.77 – 2.71 (m, 1H, C1), 2.07 – 1.99 (m, 1H, C11), 1.57 – 1.52 (m, 1H, C12), 1.13 – 1.07 (m, 1H, C12). ^{13}**C NMR** (151 MHz, $CDCl_3$) δ 198.78 (C2), 137.75 (C3), 133.18 (C8), 128.74 (C6/C10), 128.27 (C7/C9), 85.18 (d, *J* = 169 Hz, C13), 24.50 (d, *J* = 24 Hz, C11), 22.61 (d, *J* = 5 Hz, C11), 14.83 (d, *J* = 7.7 Hz, C12). **HRMS** (ESI) m/z calculated for $[M+Na]^+$ 201.0692 found 201.0682. **ATR-FTIR** (cm^{-1}): 1666, 1597, 1580, 1451, 1416, 1370, 1330, 1311, 1256, 1221, 1178, 1074, 1058, 1037, 1023, 976, 917, 892, 872, 849, 811, 784, 754, 700, 753, 618, 582.

((1*S**,2*S**)-2-(Chloromethyl)cyclopropyl)(phenyl)methanone (**16ad**)

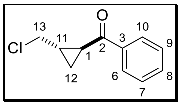

Synthesized by using the **General procedure II.** Nucleophile: Bu_4NCl (1.5. eq.).

Isolated yield: 32.1 mg, 83%.^{1}**H NMR** (400 MHz, $CDCl_3$) δ 8.05 – 8.00 (m, 2H, C6/C10), 7.61 – 7.55 (m, 1H, C8), 7.51 – 7.45 (m, 2H, C7/C9), 3.75 (dd, *J* = 11.4, 6.1 Hz, 1H, C13), 3.48 (dd, *J* = 11.4, 7.8 Hz, 1H, C13), 2.76 – 2.68 (m, 1H, C1), 2.09 – 1.98 (m, 1H, C11), 1.67 – 1.57 (m, 1H, C12), 1.17 – 1.09 (m, 1H, C12) ^{13}**C NMR** (101 MHz, $CDCl_3$) δ 198.53 (C2), 137.74 (C3), 133.15 (C8), 128.72 (C6/C10), 128.28 (C7/9), 47.48 (C13), 27.06 (C1), 24.87 (C11), 17.83 (C12). **HRMS** (ESI) m/z calculated for $[M+Na]^+$ 217.0391 found 217.0393. **ATR-FTIR** (cm^{-1}): 1666, 1597, 1580, 1449, 1400, 1338, 1312, 1266, 1227, 1216, 1178, 1135, 1105, 1077, 1051, 1033, 1021, 949, 909, 867, 778, 758, 731, 697, 650, 627.

((1*S**,2*S**)-2-(Iodomethyl)cyclopropyl)(phenyl)methanone (**16ae**)

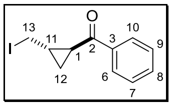

Synthesized by using the **General procedure II.** Nucleophile: Bu_4NI (1.5. eq.).

Isolated yield: 42.6 mg, 74%.^{1}**H NMR** (400 MHz, $CDCl_3$) δ 8.07 – 8.00 (m, 2H, C6/C10), 7.58 (t, *J* = 7.3 Hz, 1H, C8), 7.49 (t, *J* = 7.6 Hz, 2H, C7/C9), 3.33 (dd, *J* = 10.1, 7.3 Hz, 1H, C13), 3.22 (dd, J = 10.0, 8.0 Hz, 1H, C13), 2.68 – 2.61 (m, 1H, C1), 2.14 – 2.04 (m, 1H, C11), 1.78 (dt, J = 8.9, 4.6 Hz, 1H, C12), 1.15 – 1.06 (m, 1H, C12). ^{13}**C NMR** (101 MHz, $CDCl_3$) δ 198.28 (C2), 137.68 (C3), 133.17 (C8), 128.74 (C6/C10), 128.36 (C7/C9), 29.89 (C1), 29.28 (C11), 22.81 (C12), 8.93 (C13). **HRMS** (ESI) m/z calculated for $[M+Na]^+$ 308.9752 found 308.9745. **ATR-FTIR** (cm^{-1}): 2956, 2931, 1738, 1726, 1681, 1596, 1580, 1509, 1493, 1447, 1366, 1272, 1217, 1182, 1098, 1073, 1047, 1025, 1001, 764, 701, 674.

2-((1*R**,2*S**)-2-Benzoylcyclopropyl)acetonitrile (**16af**)

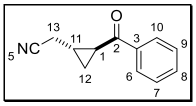

Synthesized by using the **General procedure II.** Nucleophile: Bu_4NCN (1.5. eq.).

Isolated yield: 26.6 mg, 72%.^{1}**H NMR** (400 MHz, $CDCl_3$) δ 8.05 – 7.97 (m, 2H, C6/C10), 7.63 – 7.56 (m, 1H, C8), 7.54 – 7.47 (m, 2H, C7/C9), 2.78 – 2.69 (m, 2H, C13), 2.61 (dd, *J* = 17.4, 6.0 Hz, 1H, C1), 2.02 – 1.78 (m, 1H, C11), 1.58 – 1.51 (m, 1H, C12), 1.23 – 1.13 (m, 1H, C12). ^{13}**C NMR** (101 MHz, $CDCl_3$) δ 198.19 (C2), 137.44 (C3), 133.40 (C8), 128.83 (C6/C10), 128.27 (C7/9), 117.37 (C5), 23.65 (C1), 20.56 (C13), 19.14 (C11), 17.28 (C2). HRMS (EI) m/z calculated for $[M]^{+\bullet}$ 185.0835 found 185.0834. **ATR-FTIR** (cm^{-1}): 2295, 1666, 1597, 1580, 1450, 1402, 1347, 1295, 1239, 1219, 1179, 1074, 1055, 1036, 1023, 962, 945, 751, 701, 654.

S-(((1*S**,2*S**)-2-Benzoylcyclopropyl)methyl) ethanethioate (**16ag**)

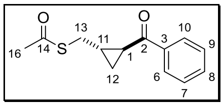
Synthesized by using the **General procedure II.** Nucleophile: Et_3NHOAc (1.5. eq.).

Isolated yield: 30.3 mg, 65%.**^{1}H NMR** (600 MHz, CDCl3) δ 8.02 – 7.97 (m, 2H, C6/C10), 7.57 (dd, *J* = 15.7, 8.4 Hz, 1H, C8), 7.48 (t, *J* = 7.7 Hz, 2H, C7/C9), 3.08 (dd, *J* = 13.9, 6.5 Hz, 1H, C13), 2.98 (dd, *J* = 13.9, 7.6 Hz, 1H, C13), 2.70 – 2.62 (m, 1H, C1), 2.34 (s, 3H, C16), 1.86 – 1.78 (m, 1H, C11), 1.57 – 1.52 (m, 1H, C12), 1.13 – 1.05 (m, 1H, C12). **^{13}C NMR** (101 MHz, $CDCl_3$) δ 198.94 (C2), 195.62 (C14), 137.80 (C3), 133.00 (C8), 128.67 (C6/C10), 128.21 (C7/C9), 32.58 (C13), 30.65 (C1), 25.73 (C16), 25.30 (C11), 18.59 (C12). **HRMS** (ESI) m/z calculated for $[M+Na]^+$ 257.0612 found 257.0607. **ATR-FTIR** (cm^{-1}): 1688, 1666, 1597, 1580, 1450, 1398, 1338, 1227, 1178, 1135, 1107, 1049, 1033, 1022, 960, 776, 759, 701, 652, 627.

((1*S**,2*S**)-2-Benzoylcyclopropyl)methyl acetate (**16ah**)

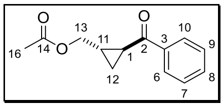
Synthesized by using the **General procedure II.** Nucleophile: Et_3NHOAc (1.5. eq.).

Isolated yield: 23.0 mg, 53%.**^{1}H NMR** (400 MHz, $CDCl_3$) δ 8.05 – 7.96 (m, 2H, C6/C10), 7.63 – 7.54 (m, 1H, C8), 7.48 (t, *J* = 7.6 Hz, 2H, C7/C9), 4.22 (dd, *J* = 11.7, 5.9 Hz, 1H, C13), 3.99 (dd, *J* = 11.7, 7.9 Hz, 1H, C13), 2.73 – 2.66 (m, 1H, C1), 2.06 (s, 3H, C16), 2.00 – 1.88 (m, 1H, C11), 1.56 – 1.46 (m, 1H, C12), 1.11 – 1.03 (m, 1H, C12). **^{13}C NMR** (101 MHz, $CDCl_3$) δ 198.86 (C2), 171.16 (C14), 137.79 (C3), 133.11 (C8), 128.72 (C6/C10), 128.24 (C7/C9), 66.46 (C13), 23.96 (C1), 23.43 (C16), 21.04 (C11), 15.70 (C12). HRMS (ESI) m/z calculated for $[M+Na]^+$ 241.0841 found 241.0836. **ATR-FTIR** (cm^{-1}): 1727, 1666, 1597, 1580, 1450, 1412, 1364, 1329, 1220, 1178, 1056, 1024, 973, 915, 888, 868, 810, 784, 754, 702, 654, 605, 546.

Phenyl((1*S**,2*S**)-2-(pyrrolidin-1-ylmethyl)cyclopropyl)methanone (**16ai**)

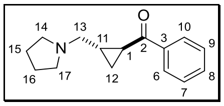
Synthesized by using the **General procedure II.** Nucleophile: Pyrrolidine (2.0 eq.).

Isolated yield: 31.4 mg, 69%.**^{1}H NMR** (600 MHz, $CDCl_3$) δ 8.03 – 7.96 (m, 2H, C6/C10), 7.55 (t, *J* = 7.4 Hz, 1H, C8), 7.46 (t, *J* = 7.7 Hz, 2H, C7/C9), 2.57 – 2.51 (m, 7H, C13/C14/C17/C1), 1.85 – 1.79 (m, 1H, C11), 1.79 – 1.74 (m, 4H, C15/C16), 1.54 – 1.48 (m, 1H, C12), 1.01 – 0.95 (m, 1H, C12). **^{13}C NMR** (151 MHz, $CDCl_3$) δ 199.66 (C2), 138.08 (C3), 132.85 (C8), 128.62 (C5/C10), 128.19 (C7/C9), 59.53 (C13), 54.32 (C14/C17),

54.20 (C14/C17), 25.14 (C1), 24.28 (C16/C15), 23.53 (C11), 17.84 (C12). **HRMS** (ESI) m/z calculated for $[M+H]^+$ 230.1539 found 230.1540. **ATR-FTIR** (cm^{-1}): 3370, 2959, 2923, 2574, 2479, 1663, 1596, 1579, 1450, 1413, 1380, 1349, 1302, 1225, 1179, 1160, 1063, 1037, 1022, 984, 910, 877, 818, 785, 762, 705, 654, 588, 571, 534.

(1*S**,2*S**)-2-Benzoylcyclobutyl methanesulfonate (**20aa**)

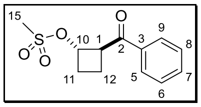

Synthesized by using the **General procedure II.** (Side product).

Isolated yield: 4.0 mg, 8%.^{1}**H NMR** (600 MHz, $CDCl_3$) δ 7.94 – 7.91 (m, 2H, C5/C9), 7.62 – 7.59 (m, 1H, C7)), 7.49 (t, *J* = 7.8 Hz, 2H, C6/C8), 5.37 – 5.28 (m, 1H, C10), 4.25 – 4.16 (m, 1H, C1), 3.04 (s, 3H, C15), 2.51 – 2.32 (m, 3H, C11/C12), 1.87 – 1.79 (m, 1H, C12). ^{13}**C NMR** (151 MHz, $CDCl_3$) δ 197.45 (C2), 135.12 (C3), 133.95 (C7), 129.01 (C5/C9), 128.65 (C6/C8), 73.04 (C10), 49.91 (C1), 37.75 (C15), 27.86 (C11), 19.34 (C12). **HRMS** (ESI): m/z calculated for $[M+Na]^+$ 277.0505 found: 277.0512.

(Bicyclo[2.2.1]heptan-2-ylidene(4-methoxyphenyl)methoxy)trimethylsilane (**17a**).

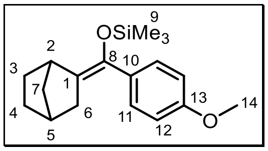

Synthesized by using **procedure K**.

Isolated yield: 140 mg, 49% (*E/Z* = 1.2/1). ^{1}**H NMR** (600 MHz, CDCl3) δ 7.34 (d, *J* = 8.9 Hz, 1H, C11b), 7.31 (d, *J* = 8.8 Hz, 1H, C11a), 6.85 (d, *J* = 8.8 Hz, 1H, C12a), 6.82 (d, *J* = 8.9 Hz, 1H, C12b), 3.81 (s, 1.4H, C14b), 3.80 (s, 1.6H, C14a), 3.18 (d, *J* = 3.2 Hz, 0.53H, C2a), 2.88 (d, *J* = 3.8 Hz, 0.47H, C2b), 2.39 (m, 1H), 2.37 – 2.32 (m, 0.47H, C6b'), 2.32 – 2.26 (m, 0.53H, C6a'), 2.02 (dd, *J* = 15.3, 2.5 Hz, 0.53H, C6a"), 1.91 (dd, *J* = 15.9, 2.9 Hz, 0.47H, C6b"), 1.76 – 1.56 (m, 2H), 1.52 – 1.47 (m, 0.5H), 1.46 – 1.40 (m, 0.5H), 1.36 (d, *J* = 9.2 Hz, 1H), 1.34 – 1.23 (m, 2H), 0.06 (s, 4.7H, C9a), 0.02 (s, 4.3H, C9b). ^{13}**C NMR** (151 MHz, $CDCl_3$) δ 158.56 (C13), 158.26 (C13), 139.40 (C8), 138.31 (C8), 132.22 (C9), 132.11 (C9), 128.94 (C11), 128.57 (C11), 127.47 (C1), 127.02 (C1), 113.23 (C12), 113.16 (C12), 55.36 (C14), 55.33 (C14), 41.33 (C2b), 41.06 (C2a), 40.31, 39.09, 37.42 (C6a), 37.37, 37.15 (C6b), 36.43, 30.11, 28.99, 28.88, 28.78, 0.68 (C9), 0.67 (C9). **HRMS** (ESI) m/z calculated for $[M+H]^+$ requires 303.1775, found 303.1773. **ATR-FTIR** (cm^{-1}): 3358, 2954, 2921,2851, 1659, 1632, 1600, 1467, 1256.

(Bicyclo[2.2.1]heptan-2-ylidene(phenyl)methoxy)trimethylsilane (**17b**)

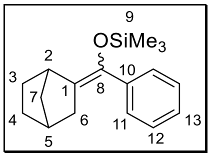

Synthesized by using **procedure K**.

Isolated yield: 437 mg, 43% (*E/Z* = 2.3/1). **^{1}H NMR** (400 MHz, $CDCl_3$) δ 7.45 – 7.35 (m, 2H), 7.33 – 7.27 (m, 2H), 7.25 – 7.15 (m, 1H, C13), 3.20 (d, *J* = 3.4 Hz, 0.3H, C2b), 2.92 (d, *J* = 3.8 Hz, 0.7H, C2a), 2.43 – 2.28 (m, 1.3H, C5, Calk), 2.37 – 2.33 (m, 0.6H), 2.06 (dd, *J* = 15.4, 2.6 Hz, 0.3H), 1.93 (dd, *J* = 15.6, 3.0 Hz, 0.7H), 1.79 – 1.58 (m, 2H), 1.58 – 1.48 (m, 0.6H), 1.48 – 1.40 (m, 0.3H), 1.40 – 1.20 (m, 3H), 0.07 (s, 2.8H, C9b), 0.03 (s, 6.2H, C9a). **^{13}C NMR** (101 MHz, $CDCl_3$) δ 127.9, 127.8, 127.8, 127.3, 126.9, 126.7, 41.4 (C2), 41.2 (C2), 40.3, 39.1, 37.5, 37.4 (C5), 37.2, 36.4 (C5), 30.1, 28.9, 28.9, 28.7, 0.7 (C9). **HRMS** (ESI) m/z calculated for $[M+H]^+$ requires 291.1575, found 291.1564. **ATR-FTIR** (cm^{-1}): 2954, 2867, 1680, 1491, 1445, 1303, 1267, 1250, 1218, 1164, 1094, 1071, 1028, 885, 865, 838, 725, 697, 667.

([1,1'-Biphenyl]-4-yl(bicyclo[2.2.1]heptan-2-ylidene)methoxy)trimethylsilane (**17c**).

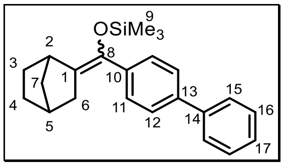

Synthesized by using **procedure J**.

Isolated yield: 313 mg, 45% (*E/Z* = 1.5/1). **^{1}H NMR** (600 MHz, $CDCl_3$) δ 7.62 (t, *J* = 6.6 Hz, 2H, C16), 7.56 (d, *J* = 8.3 Hz, 1H, C11), 7.52 (dd, *J* = 23.6, 8.4 Hz, 2H, C15), 7.46 (d, *J* = 8.3 Hz, 1H, C11), 7.45 – 7.41 (m, 2H, C12), 7.35 – 7.31 (m, 1H, C17), 3.23 (d, *J* = 3.1 Hz, 0.42H, C2a), 2.99 (d, *J* = 3.5 Hz, 0.58H, C2b), 2.45 – 2.40 (m, 1.5H), 2.39 – 2.36 (m, 0.58H), 2.12 (dd, *J* = 15.2, 2.4 Hz, 0.42H), 1.95 (dd, *J* = 15.6, 2.9 Hz, 0.58H), 1.78 (ddd, *J* = 16.0, 11.5, 4.4 Hz, 0.65H), 1.74 – 1.56 (m, 2H), 1.46 (dd, *J* = 13.2, 7.4 Hz, 0.48H), 1.42 – 1.36 (m, 1H), 1.37 – 1.22 (m, 3H), 0.11 (s, 3.6H, C9a), 0.06 (s, 5.4H, C9b). **^{13}C NMR** (151 MHz, $CDCl_3$) δ 141.00 (C14), 140.98 (C14), 139.56 (C13), 139.46 (C13), 139.18 (C10), 138.56 (C10), 138.40 (C8), 138.36 (C8), 129.24 (C1), 129.08 (C1), 128.89 (C16), 128.88 (C16), 128.05 (C17), 127.59 (C17), 127.31 (C12), 127.28 (C12), 127.09 (C15), 127.07 (C15), 126.53 (C11), 126.45 (C11), 41.49 (C2b), 41.35 (C2a), 40.39, 39.10, 37.70, 37.40, 36.37, 30.04, 28.91, 28.86, 28.71, 0.74 (C9), 0.73 (C9). **HRMS** (ESI) m/z calculated for $[M+H]^+$ requires 349.1982, found 349.1973. **ATR-FTIR** (cm^{-1}): 3393, 3383, 3370, 3360, 3347, 30557, 3031, 2954, 2870, 1670, 1602, 1486, 1351, 1311, 1299, 1253, 1186, 1167, 996, 985, 888, 864, 828.

(Bicyclo[2.2.1]heptan-2-ylidene(3-fluorophenyl)methoxy)trimethylsilane (**17d**).

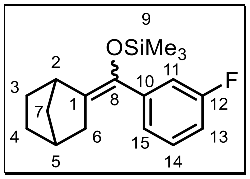

Synthesized by using **procedure J**.

Isolated yield: 312 mg, 74% (*E/Z* = 1/1). **^{1}H NMR** (600 MHz, $CDCl_3$) δ 7.28 – 7.22 (m, 1H), 7.20 (d, *J* = 7.8 Hz, 0.5H), 7.17 (d, *J* = 7.7 Hz, 0.5H), 7.12 (d, *J* = 10.7 Hz, 0.5H), 7.08 (d, *J* = 10.3 Hz, 0.5H), 6.92 (t, *J* = 8.4 Hz, 0.5H), 6.91 (td, *J* = 8.5, 2.3 Hz, 0.5H), 6.88 (dd, *J* = 11.4, 5.1 Hz, 0.5H), 3.19 (d, *J* = 2.5 Hz, 0.5H, H2), 2.92 (d, *J* = 2.8 Hz, 0.5H, C2), 2.41 (d, *J* = 2.9 Hz, 1H, C5), 2.38 – 2.30 (m, 1H), 2.06 (dd, *J* = 15.5, 2.1 Hz, 0.5H), 1.92 (dd, *J* = 16.2, 2.7 Hz, 0.5H), 1.76 (ddd, *J* = 15.9, 11.6, 4.3 Hz, 0.5H), 1.72 – 1.56 (m, 2H), 1.50 (t, *J* = 10.1 Hz, 0.5H), 1.43 (d, *J* = 9.6 Hz, 0.5H), 1.37 (d, *J* = 8.9 Hz, 1H), 1.28 (dd, *J* = 21.4, 10.2 Hz, 3H), 0.08 (s, 4.5H, C9), 0.04 (s, 4.5H, C9). **^{19}F NMR** (565 MHz, $CDCl_3$) δ -114.04 – -114.05 (m). **^{13}C NMR** (151 MHz, $CDCl_3$) δ 162.65 (d, *J* = 244.5 Hz, C12), 141.90 (d, *J* = 7.6 Hz, C10), 141.73 (d, *J* = 7.7 Hz, C10), 129.97 (d, *J* = 14.2 Hz, C8), 129.30 (d, *J* = 8.3 Hz, C14), 129.21 (d, *J* = 8.4 Hz, C14), 123.08 (d, *J* = 74.1 Hz, C15), 114.42 (d, *J* = 22.0 Hz, C11), 113.99 (d, *J* = 22.3 Hz, C13), 113.78 (d, *J* = 21.3 Hz, C11), 113.43 (d, *J* = 21.2 Hz, C13), 41.41 (C2), 41.35 (C2), 40.36, 39.06, 37.66, 37.37, 37.32, 36.29 (C5), 31.40, 29.88, 28.77, 28.63, 0.66 (C9), 0.63 (C9). **HRMS** (ESI) m/z calculated for $[M+H]^+$ requires 291.1575, found 291.1564. **ATR-FTIR** (cm^{-1}): 3484, 3075, 2958, 2958, 2876, 1680, 1610, 1586, 1482, 1436, 1309, 1295, 1269, 1182, 1080, 1050, 1020, 1004, 849, 829, 797, 766, 756, 741, 697.

(Bicyclo[2.2.1]heptan-2-ylidene(4-(trifluoromethyl)phenyl)methoxy)trimethylsilane (**17e**).

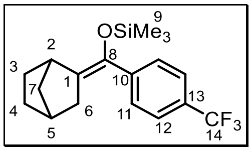

Synthesized by using **procedure J**.

Isolated yield: 420 mg, 49% (*E/Z* = 1.2/1). **^{1}H NMR** (400 MHz, $CDCl_3$) δ 7.59 – 7.48 (m, 4H, H12, C13), 3.22 (d, *J* = 2.9 Hz, 0.56H, C2b), 2.91 (d, *J* = 3.9 Hz, 0.44H, C2a), 2.41 (m, 1H), 2.38 – 2.34 (m, 0.57H), 2.34 – 2.29 (m, 0.45H), 2.06 (dd, *J* = 15.5, 2.5 Hz, 0.57H), 1.95 (dd, *J* = 16.1, 3.0 Hz, 0.45H), 1.83 – 1.56 (m, 2H), 1.51 – 1.22 (m, 4H), 0.08 (s, 4.8H, C9b), 0.04 (s, 4.2H, C9a). **^{13}C NMR** (151 MHz, $CDCl_3$) δ 143.41 (C10), 143.13 (C10), 138.62 (C8), 137.61 (C8), 131.22 (C1), 131.16 (C1), 130.45 (q, *J* = 32.5 Hz, C13), 127.79, 127.73, 127.24, 126.11 (q, *J* = 3.7 Hz), 124.91 (q, *J* = 3.8 Hz), 124.81 (q, *J* = 3.8 Hz), 124.25 (q, *J* = 272.0 Hz, C14). 41.46, 40.41, 39.06, 37.71, 37.47, 37.31, 36.26, 29.86, 28.71, 28.57, 22.49, 14.21, 0.66 (C9). **^{19}F NMR** (565

MHz, $CDCl_3$) δ -62.39 (s), -62.57 (s). **HRMS** (EI) m/z calculated for $M^{+\bullet}$ requires *m/z* 340.1470, found *m/z* 340.1464. **ATR-FTIR** (cm^{-1}): 3359, 2956, 2922, 2852, 1659, 1632, 1466, 1377, 1325, 1264, 741, 706.

(Bicyclo[2.2.1]heptan-2-ylidene(2-methoxyphenyl)methoxy)trimethylsilane (**17f**).

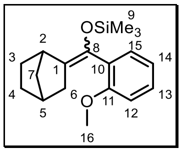

Synthesized by using **procedure J**.

Isolated yield: 295 mg, 52% (*E/Z* = 2/1). **^{1}H NMR** (600 MHz, $CDCl_3$) δ 7.25 – 7.14 (m, 2H, C13, C15), 6.93 – 6.81 (m, 2H, C12, C14), 3.81 (s, 1H, C16b), 3.81 (s, 2H, C16a), 3.21 (d, J = 3.4 Hz, 0.67H, C2a), 2.50 (s, 0.33H, C2b), 2.39 (s, 0.5H), 2.33 (dd, *J* = 4.1, 2.0 Hz, 0.3H), 2.29 (s, 0.8H), 2.03 – 1.91 (m, 1.3H), 1.66 (ddd, J = 15.9, 8.5, 3.5 Hz, 1.6H), 1.61 – 1.56 (m, 1H), 1.49 – 1.44 (m, 0.9H), 1.44 – 1.34 (m, 0.9H), 1.32 – 1.18 (m, 1.9H), 1.32 – 1.17 (m, 3.6H), -0.02 (s, 6H, C9), -0.03 (s, 3H, C9). **^{13}C NMR** (151 MHz, $CDCl_3$) δ 157.36 (C11), 157.12 (C11), 131.48 (C15), 131.28 (C15), 128.89 (C13), 128.85 (C13), 128.19 (C8), 128.17 (C8), 120.08 (C14), 120.00 (C14), 111.00 (C12), 110.85 (C12), 55.33 (C16), 55.24 (C16), 41.36 (C2), 40.03 (C2), 39.99, 39.32, 36.99, 36.75, 36.05, 36.01, 29.26, 28.88, 0.50 (C9). **HRMS** (ESI) m/z calculated for $[M+Na]^+$ requires 325.1594, found 325.1595. **ATR-FTIR** (cm^{-1}): 3360, 3188, 2922, 2851, 2360, 2340, 1658, 1632, 1551, 1467, 764, 751, 701.

(Bicyclo[2.2.1]heptan-2-ylidene(naphthalen-2-yl)methoxy)trimethylsilane (**17g**).

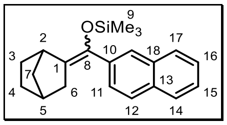

Synthesized by using **procedure J**.

Isolated yield: 210 mg, 46% (*E/Z* = 1.35/1). **^{1}H NMR** (600 MHz, $CDCl_3$) δ 7.89 – 7.69 (m, 4H), 7.57 (m, 1H), 7.52 – 7.38 (m, 2H), 3.26 (d, *J* = 2.9 Hz, 0.63H), 3.00 (d, *J* = 3.4 Hz, 0.37H), 2.46 – 2.41 (m, 1H, C5), 2.41 – 2.36 (m, 1H), 2.18 – 2.10 (m, 0.63H), 2.03 – 1.92 (m, 0.37H), 1.84 – 1.76 (m, 0.37H), 1.75 – 1.57 (m, 2H), 1.49 (t, *J* = 10.3 Hz, 0.63H), 1.40 (t, *J* = 8.9 Hz, 1H), 1.31 (dd, *J* = 15.1, 8.0 Hz, 1H), 0.08 (s, 5.7H, C9a), 0.04 (s, 3.4H, C9b). **^{13}C NMR** (151 MHz, $CDCl_3$) δ 139.57, 138.52, 136.93, 136.78, 133.05, 133.03, 132.49, 132.25, 129.46, 129.19, 128.07, 128.00, 127.56, 127.50, 127.20, 127.11, 126.16, 126.10, 125.90, 125.88, 125.64, 125.59, 41.30 (C2), 41.17 (C2), 40.23, 38.95, 37.48, 37.24, 37.22 (C5), 36.25 (C5), 34.11, 29.93, 28.77, 28.70, 28.57, 22.33, 14.05, 0.56 (C9), 0.54 (C9). **HRMS** (ESI) m/z calculated for $[M+H]^+$ requires 323.1826

found 323.1826. **ATR-FTIR** (cm^{-1}): 2956, 2925, 2870, 2853, 1673, 1626, 1276, 1260, 1117, 862, 843, 825, 751.

(Bicyclo[2.2.1]heptan-2-ylidene(furan-2-yl)methoxy)trimethylsilane (**17h**).

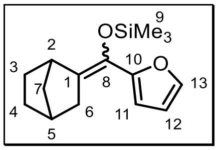

Synthesized by using **procedure K**.

Isolated yield: 998 mg, 38% (*E/Z* = 2.1/1). **^{1}H NMR** (400 MHz, $CDCl_3$) δ 7.35 (dd, *J* = 1.7, 0.7 Hz, 0.32H, C13b), 7.34 (dd, *J* = 1.7, 0.7 Hz, 0.68H, C13a), 6.39 – 6.35 (m, 1H, C12), 6.24 (d, *J* = 3.3 Hz, 0.32H, C11b), 6.19 (d, *J* = 3.3 Hz, 0.68H, C11a), 3.34 (d, *J* = 3.6 Hz, 0.32H, C2b), 3.17 (d, *J* = 2.7 Hz, 0.68H, C2a), 2.44 (d, *J* = 2.9 Hz, 1H, C5), 2.40 (dd, *J* = 3.9, 2.2 Hz, 0.67H), 2.35 – 2.27 (m, 0.32H), 2.13 (dd, *J* = 15.5, 2.7 Hz, 0.67H), 1.98 (dd, *J* = 16.3, 2.9 Hz, 0.33H), 1.78 – 1.56 (m, 2H), 1.45 – 1.35 (m, 2H), 1.35 – 1.23 (m, 2H), 0.19 (s, 6H, C9a), 0.15 (s, 3H, C9b). **^{13}C NMR** (151 MHz, $CDCl_3$) δ 153.06 (C10), 152.78 (C10), 140.94 (C13), 140.79 (C13), 131.90 (C8), 131.31 (C8), 129.76 (C1), 128.99 (C1), 110.93 (C12), 110.71 (C12), 106.66 (C11), 106.26 (C11), 40.94 (C2), 40.93, 40.31, 39.33, 37.70, 37.25, 37.14 (C5), 36.22 (C5), 29.68, 28.92, 28.56, 28.46, 0.57 (C9). **HRMS** (ESI): m/z calculated for $[M+H]^+$ requires 263.1462, found 263.1464. **ATR-FTIR** (cm^{-1}): 2954, 2924, 2868, 1668, 1490, 1312, 1219, 1167, 1156, 1135, 1122, 1045, 1010, 954, 942, 885, 868, 838.

Please note: the following nortricyclenes are C_2-symmetric.

(4-Methoxyphenyl)((2*R*,6*R*)-tricyclo[2.2.1.0^{2,6}]heptan-1-yl)methanone (**19a**)

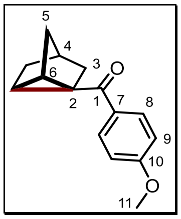

Synthesized by using **procedure III**. Alternative eluent for column chromatography – heptane : CH_2Cl_2 → 50 : 50 v/v%.

Isolated yield: 29 mg, 43%. **^{1}H NMR** (600 MHz, $CDCl_3$) δ 7.81 – 7.76 (m, 2H, C9), 6.92 – 6.87 (m, 2H, C8), 3.85 (s, 3H, C11), 2.19 (dq, *J* = 2.6, 1.4 Hz, 1H, C4), 2.10 (s, 2H, C6), 1.76 (d, *J* = 1.6 Hz, 2H, C3), 1.50 (dd, *J* = 10.7, 1.7 Hz, 2H, C5a), 1.44 – 1.39 (m, 2H, C5b). **^{13}C NMR** (151 MHz, $CDCl_3$) δ 201.00 (C1), 162.49 (C10), 132.28 (C7), 130.37 (2C, C9), 113.48 (2C, C8), 55.53 (C11), 36.41 (C3), 33.68 (C5), 31.94 (C4), 25.77 (C6).

HRMS (ESI) m/z calculated for $[M+H]^+$ requires 229.1223 found 229.1221. **ATR-FTIR** (cm^{-1}): 2924, 2853, 1637, 1600, 1509, 1464, 1309, 1256, 1228, 1031, 756.

Phenyl((2*R*,6*R*)-tricyclo[2.2.1.0^{2,6}]heptan-1-yl)methanone (**19b**)

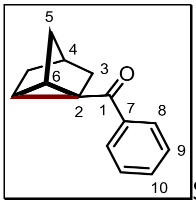

Synthesized by using **procedure III**.

Isolated yield: 14 mg, 71%. **^{1}H NMR** (600 MHz, $CDCl_3$) δ 7.73 – 7.68 (m, 2H), 7.50 – 7.44 (m, 1H), 7.40 (tt, *J* = 6.7, 1.4 Hz, 2H), 2.19 (hept, *J* = 1.4 Hz, 1H, C4), 2.14 (d, *J* = 0.9 Hz, 2H, C6), 1.72 (d, *J* = 1.7 Hz, 2H, C3), 1.54 – 1.48 (m, 2H, C5a), 1.41 (dd, *J* = 10.9, 1.7 Hz, 2H, C5b). **^{13}C NMR** (151 MHz, $CDCl_3$) δ 203.22 (C1), 139.79 (C7), 131.50 (C10), 128.31 (C9), 127.84 (C8), 35.81 (C3), 35.32 (C2), 33.69 (C5), 31.93 (C4), 26.32 (C6). **HRMS** (ESI) m/z calculated for $[M+H]^+$ requires 199.1117, found 199.1118. **ATR-FTIR** (cm^{-1}): 3061, 2941, 2865, 1648, 1598, 1578, 1446, 1302, 1283, 1200, 1178, 1073, 1046, 1005.

[1,1'-Biphenyl]-4-yl((2*R**,6*R**)-tricyclo[2.2.1.0^{2,6}]heptan-1-yl)methanone (**19c**)

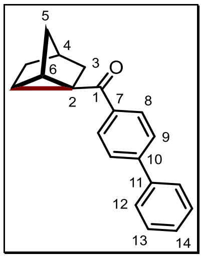

Synthesized by using **procedure III**.

Isolated yield: 67 mg, 81%. **^{1}H NMR** (600 MHz, $CDCl_3$) δ 7.84 – 7.79 (m, 2H, C8), 7.67 – 7.59 (m, 4H, H9, C12), 7.50 – 7.42 (m, 2H, C13), 7.42 – 7.36 (m, 1H, C14), 2.22 (dp, *J* = 2.8, 1.2 Hz, 1H, C4), 2.19 – 2.16 (m, 2H, C6), 1.78 (d, *J* = 1.6 Hz, 2H, C3), 1.56 – 1.50 (m, 2H, C5a), 1.44 (dd, *J* = 10.9, 1.6 Hz, 2H, C5b). **^{13}C NMR** (151 MHz, $CDCl_3$) δ 202.57 (C1), 144.33 (C11), 140.32 (C7), 138.39 (C10), 129.05 (C13), 128.52 (C8), 128.11 (C14), 127.37 (C9/C12), 126.99 (C9/C12), 35.98 (C3), 33.73 (C5), 31.91 (C4), 26.42 (C6). **HRMS** (ESI) m/z calculated for $[M+H]^+$ requires 275.1430 found 275.1430. **ATR-FTIR** (cm^{-1}): 2923, 2853, 1643, 1604, 1367, 1315, 1282, 1226, 744, 697.

(3-Fluorophenyl)((2*R**,6*R**)-tricyclo[2.2.1.$0^{2,6}$]heptan-1-yl)methanone (**19d**)

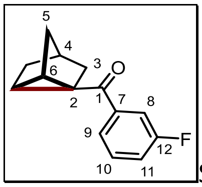

Synthesized by using **procedure III**.

Isolated yield: 33 mg, 51%. **^{1}H NMR** (600 MHz, $CDCl_3$) δ 7.49 (d, *J* = 7.7 Hz, 1H, C8), 7.38 (tt, *J* = 7.9, 4.1 Hz, 2H, H9, C10), 7.17 (td, *J* = 8.4, 2.6 Hz, 1H, C11), 2.21 (s, 1H, C4), 2.16 (s, 2H, C6), 1.72 (d, *J* = 1.7 Hz, 2H, C3), 1.51 (d, *J* = 10.7 Hz, 2H, C5a), 1.45 – 1.40 (m, 2H, C5b). **^{13}C NMR** (151 MHz, $CDCl_3$) δ 201.8 (C1), 162.61 (d, *J* = 247.5 Hz, C12), 141.70 (d, *J* = 6.2 Hz, C7), 129.98 (d, *J* = 7.8 Hz, C9/C10), 123.50 (d, *J* = 3.1 Hz, C8), 118.42 (d, *J* = 21.3 Hz, C11), 114.79 (d, *J* = 22.5 Hz, C9/C10) 35.67 (C3), 33.70 (C5), 31.84 (C4), 26.92 (C6). **^{19}F NMR** (565 MHz, $CDCl_3$) δ -112.33 (m). **HRMS** (ESI) m/z calculated for $[M+H]^+$ requires 217.1023, found 217.1022. **ATR-FTIR** (cm^{-1}): 3069, 2947, 2868, 1724, 1610, 0585, 1223, 1029, 638.

((2*R**,6*R**)-Tricyclo[2.2.1.$0^{2,6}$]heptan-1-yl)(4-(trifluoromethyl)phenyl)methanone (**19e**)

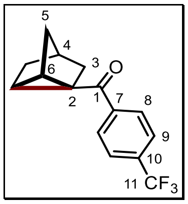

Synthesized by using **procedure III**. Alternative eluent for column chromatography – heptane : CH_2Cl_2 → 50 : 50 v/v%.

Isolated yield: 42 mg, 53%. **^{1}H NMR** (600 MHz, $CDCl_3$) δ 7.79 – 7.75 (m, 2H, C8), 7.69 – 7.65 (m, 2H, C9), 2.22 (hept, *J* = 1.4 Hz, 1H, C4), 2.18 (s, 2H, C6), 1.70 (d, *J* = 1.6 Hz, 2H, C3), 1.54 – 1.49 (m, 2H, C5a), 1.44 (dd, *J* = 10.9, 1.6 Hz, 2H, C5b). **^{13}C NMR** (151 MHz, $CDCl_3$) δ 202.58 (C1), 142.97 (C7), 133.00 (q, *J* = 32.8 Hz, C10), 128.06 (C8), 125.49 (q, *J* = 3.7 Hz, C9), 123.98 (d, *J* = 272.5 Hz, C11), 35.82 (C2), 35.55 (C3), 33.82 (C5), 31.95 (C4), 27.31 (C6). **^{19}F NMR** (565 MHz, $CDCl_3$) δ -62.97. **HRMS (EI):** m/z calculated for $M^{+\bullet}$ requires 266.0918 found 266.0906. **ATR-FTIR** (cm^{-1}): 2925, 2867, 1655, 1407, 1369, 1324, 1282, 1224, 1129, 1091, 1016, 835, 764, 749.

(2-Methoxyphenyl)((2*R*,6*R*)-tricyclo[2.2.1.0^{2,6}]heptan-1-yl)methanone (**19f**)

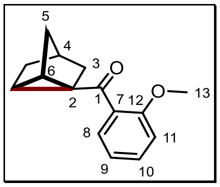

Synthesized by using **procedure III**. Alternative eluent for column chromatography – heptane : CH_2Cl_2 → 50 : 50 v/v%.

Isolated yield: 8 mg, 12%. **^{1}H NMR** (600 MHz, $CDCl_3$) δ 7.33 (ddd, *J* = 8.3, 7.4, 1.8 Hz, 1H, C10), 7.16 (dd, *J* = 7.4, 1.8 Hz, 1H, C8), 6.95 (td, *J* = 7.4, 0.9 Hz, 1H, C9), 6.90 (dd, *J* = 8.4, 0.9 Hz, 1H, C11), 3.83 (s, 3H, C13), 2.11 (p, *J* = 1.5 Hz, 1H, C4), 2.05 (s, 2H, C6), 1.50 (d, *J* = 1.7 Hz, 2H, H3), 1.45 (dd, *J* = 10.2, 1.5 Hz, 2H, C5a), 1.36 (dd, *J* = 10.7, 1.6 Hz, 2H, C5b). **^{13}C NMR** (151 MHz, $CDCl_3$) δ 206.05 (C1), 155.95 (C12), 131.20 (C7), 130.71 (C10), 127.51 (C8), 120.54 (C9), 111.20 (C11), 55.65 (C13), 33.89 (C3), 33.49 (C5), 31.46 (C4), 26.77 (C6). **HRMS** (ESI) m/z calculated for $[M+H]^+$ requires 229.1223, found 229.1222. **ATR-FTIR** (cm^{-1}): 2922, 2852, 1655, 1276, 1261, 764, 750.

Naphthalen-2-yl((2*R*,6*R*)-tricyclo[2.2.1.0^{2,6}]heptan-1-yl)methanone (**19g**)

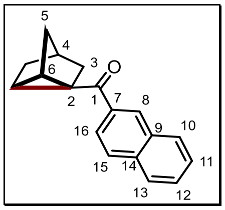

Synthesized by using **procedure III**. Alternative eluent for column chromatography – heptane : CH_2Cl_2 → 50 : 50 v/v%.

Isolated yield: 41 mg, 55%. **^{1}H NMR** (600 MHz, $CDCl_3$) δ 8.24 (s, 1H), 7.92 (d, *J* = 8.0 Hz, 1H), 7.86 (d, *J* = 8.4 Hz, 2H), 7.81 (dd, *J* = 8.6, 1.7 Hz, 1H), 7.54 (dtd, *J* = 16.1, 6.9, 1.2 Hz, 2H), 2.24 – 2.22 (m, 1H, C4), 2.22 (s, 2H, C6), 1.81 (d, *J* = 1.4 Hz, 2H, C3), 1.55 (d, *J* = 10.7 Hz, 2H, C5a), 1.45 (d, *J* = 10.0 Hz, 2H, C5b). **^{13}C NMR** (151 MHz, $CDCl_3$) δ 203.01, 137.04, 134.81, 132.54, 129.32, 128.54, 128.08, 127.85, 126.69, 124.61, 36.01 (C3), 33.74 (C5), 31.93 (C4), 26.52 (C6). **HRMS** (ESI) m/z calculated for $[M+H]^+$ requires 249.1274 found 249.1272. **ATR-FTIR** (cm^{-1}): 2941, 2865, 1649, 1466, 1421, 1370, 1325, 1305, 1283, 1248, 1189, 1140, 1121, 1087, 789, 771, 758.

Furan-2-yl((2*R*,6*R*)-tricyclo[2.2.1.0^{2,6}]heptan-1-yl)methanone (**19h**)

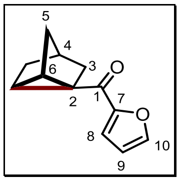

Synthesized by using **procedure III**. Alternative eluent for column chromatography – heptane : CH_2Cl_2 → 50 : 50 v/v%.

Isolated yield: 29 mg, 51%. **^{1}H NMR** (600 MHz, $CDCl_3$) δ 7.51 (dd, *J* = 1.7, 0.8 Hz, 1H, C10), 7.13 (dd, *J* = 3.6, 0.8 Hz, 1H, C8), 6.48 (dd, *J* = 3.6, 1.7 Hz, 1H, C9), 2.31 (s, 2H, C6), 2.16 (dtt, *J* = 2.4, 1.6, 0.8 Hz, 1H, C4), 1.79 (d, *J* = 1.6 Hz, 2H, C3), 1.49 – 1.43 (m, 2H, C5a), 1.40 (dd, *J* = 10.8, 1.6 Hz, 2H, C5b). **^{13}C NMR** (151 MHz, $CDCl_3$) δ 189.53 (C1), 153.45 (C7), 145.24 (C10), 116.42 (C8), 111.87 (C9), 34.29 (C3), 33.87 (C5), 30.95 (C4), 27.93 (C6). **HRMS** (ESI) m/z calculated for $[M+H]^+$ requires 189.0910, found 189.0911. **ATR-FTIR** (cm^{-1}): 3134, 3117, 2984, 2942, 2900, 2865, 1635, 1565, 1465, 1447, 1370, 1286, 1258, 1227, 1167, 1149, 1077, 1030, 955, 934, 911, 902, 876.

(*Z*)-((2-Cyclopropyl-1-phenylvinyl)oxy)trimethylsilane (**31**)

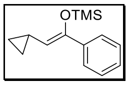

Synthesized by using the **General procedure A.** (*Z/E* > 20: 1).

Isolated yield: 120 mg*, 73% from the* carboxylic acid.

Spectroscopic properties match with the literature.[15]

N,N-Dimethylbicyclo[2.2.1]heptane-2-carboxamide (**34a**)

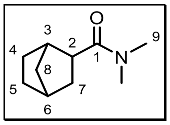

Synthesized by using **procedure H**, step 1 using dimethylamine in THF (1 M solution).

Isolated yield: 869 mg, 79%.-Spectroscopic properties match with the literature.[17]

N,N-Diethylbicyclo[2.2.1]heptane-2-carboxamide (**34b**)

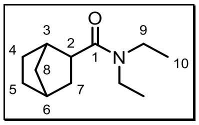

Synthesized by using **procedure H**, step 1 using diethylamine.

Isolated yield: 567 mg, 71%. (undefined d.r.) **^{1}H NMR** (600 MHz, $CDCl_3$) δ 3.59 (dtd, *J* = 14.1, 8.1, 7.6, 6.7 Hz, 0.77H, C9), 3.51 (dtd, *J* = 14.2, 7.1, 0.9 Hz, 0.77H, C9), 3.40 (tq, *J* = 14.4, 7.1 Hz, 0.50H, C9), 3.31 (ddq, *J* = 18.7, 14.3, 7.1 Hz, 1.24H), 3.20 (dq, *J* = 14.0, 7.1 Hz, 0.76H, C9), 2.88 (dtd, *J* = 10.8, 4.3, 1.9 Hz, 0.74H, C2), 2.41 (tt, *J* = 4.3, 1.9 Hz, 0.72H, C3), 2.39 – 2.35 (m, 0.26H, C2), 2.35 – 2.33 (m, 0.26H, C3), 2.32 – 2.29 (m, 0.24H, C6), 2.27 (q, *J* = 3.1, 1.7 Hz, 0.72H, C6), 1.93 (ddd, *J* = 12.0, 4.5, 2.6 Hz, 0.72H), 1.89 (dddd, *J* = 12.0, 6.4, 4.1, 2.4 Hz, 0.27H), 1.68 (dt, *J* = 9.7, 2.0 Hz, 0.26H), 1.59 (dd, *J* = 4.5, 3.0 Hz, 0.24H), 1.58 – 1.53 (m, 1H), 1.50 (ddtt, *J* = 12.4, 7.4, 4.7, 2.5 Hz, 0.76H), 1.48 – 1.39 (m, 2.6H), 1.39 – 1.36 (m, 0.76H), 1.36 – 1.26 (m, 1H), 1.26 – 1.21 (m, 0.5H), 1.19 (td, *J* = 7.1, 2.8 Hz, 3.21H, C10), 1.12 (dt, *J* = 12.5, 7.1 Hz, 3H, C10). **^{13}C NMR** (151 MHz, $CDCl_3$) δ 172.79 (C1), 174.81 (C1), 43.68 (C2), 43.90 (C2), 41.41 (C9a), 41.44 (C9a), 40.97, 40.58 (C3), 41.24 (C3), 39.83 (C9b), 40.01 (C9b), 36.64, 35.99 (C6), 37.27 (C6), 35.28, 32.52, 29.83, 28.93, 28.84, 24.46, 14.67 (C10a), 14.46 (C10a), 13.21 (C10b), 13.05 (C10b). **HRMS** (ESI) m/z calculated for $[M+H]^+$ requires 196.1696, found 196.1696. **ATR-FTIR** (cm^{-1}): 2360, 2341, 1275, 1260, 764, 750.

N,N-Dibenzylbicyclo[2.2.1]heptane-2-carboxamide (**34c**)

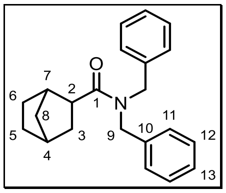

Synthesized by using **procedure H**, step 1 using dibenzylamine.

Isolated yield: 616 mg, 47% (undefined d.r.). **^{1}H NMR** (600 MHz, $CDCl_3$) δ 7.42 – 7.34 (m, 2H), 7.34 – 7.26 (m, 3H), 7.25 – 7.21 (m, 2H), 7.22 – 7.12 (m, 3H), 4.99 (d, *J* = 14.8 Hz, 0.79H), 4.72 (dd, *J* = 15.9, 9.3 Hz, 0.84H), 4.53 (d, *J* = 16.9 Hz,0.21H), 4.45 – 4.35 (m, 1H), 4.30 (d, *J* = 15.1 Hz, 0.25H), 4.18 (d, *J* = 14.8 Hz, 0.70H), 4.07 (d, *J* = 15.1 Hz, 0.26H), 3.97 (dq, *J* = 10.0, 7.1 Hz, 0.15H), 3.83 (dq, *J* = 10.0, 7.1 Hz, 0.15H), 3.00 (dtd, *J* = 10.7, 4.4, 1.8 Hz, 0.83H), 2.48 (ddd, *J* = 8.5, 5.7, 2.9 Hz, 0.21H), 2.44 (s, 0.16H), 2.43 – 2.38 (m, 0.67H), 2.33 (s, 0.19H), 2.29 (t, *J* = 4.3 Hz, 0.67H), 2.01 (ddd, *J* = 12.0, 4.6, 2.5 Hz, 0.88H), 1.75 (dq, *J* = 9.6, 2.0 Hz, 0.22H), 1.65 – 1.56 (m, 2H), 1.55 – 1.46 (m, 2H), 1.46 – 1.33 (m, 2H). **^{13}C NMR** (151 MHz, $CDCl_3$) δ 174.51, 137.92, 137.04, 129.06, 129.03, 128.71, 128.68, 128.61, 128.37, 128.26, 127.72, 127.68, 127.66, 127.37, 126.71, 126.65, 60.03, 49.68, 49.63, 47.92, 47.88, 46.15, 44.16, 44.05, 41.46, 41.14, 40.84, 37.41, 36.85, 36.25, 35.37, 32.88, 29.82, 29.23, 28.92, 24.73, 15.71. **HRMS** (ESI) m/z calculated for $[M+H]^+$ requires 320.2009, found 320.2007. **ATR-FTIR** (cm^{-1}): 3064, 3048, 3028, 2948, 2868, 1642, 1604, 1494, 1452, 1420, 1361, 1214, 1193, 1214, 1193, 1080, 1020, 995.

Bicyclo[2.2.1]heptan-2-yl(pyrrolidin-1-yl)methanone (**34d**)

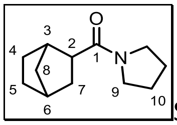

Synthesized by using **procedure H**, step 1 using pyrrolidine .

Isolated yield: 728 mg, 93% undefined d.r. **^{1}H NMR** (600 MHz, $CDCl_3$) δ 3.55 (dt, *J* = 11.9, 7.0 Hz, 0.89H, C9), 3.47 (dddd, *J* = 21.6, 14.2, 9.3, 5.4 Hz, 3.11H, C9), 2.81 (dtd, *J* = 11.0, 4.5, 1.8 Hz, 0.87H, C2), 2.49 (t, *J* = 4.4 Hz, 0.84H, C3), 2.39 (d, *J* = 3.2 Hz, 0.16H, C3), 2.35 – 2.31 (m, 0.20H, C2), 2.31 (s, 0.15H, C6), 2.29 – 2.26 (m, 0.80H, C6), 1.95 (ddt, *J* = 12.4, 9.0, 6.4 Hz, 2H, C10), 1.90 (dd, *J* = 4.7, 2.5 Hz, 0.50H), 1.89 – 1.82 (m, 2H, C10), 1.67 (t, *J* = 2.0 Hz, 0.12H), 1.62 – 1.54 (m, 1.17H), 1.54 – 1.46 (m, 1.72H), 1.45 – 1.36 (m, 1.74H), 1.33 (ddtd, *J* = 15.8, 11.5, 4.2, 2.0 Hz, 0.91H), 1.19 (ddt, *J* = 29.6, 9.7, 2.2 Hz, 0.56H).**^{13}C NMR** (151 MHz, $CDCl_3$) δ 172.75 (174.37 – C1), 46.48 (46.05 – C9), 45.93, 45.73 (46.01 –C2), 41.05, 39.42 (40.79 – C3), 37.33 (36.10 – C6), 34.37, 32.13, 30.01, 29.24, 29.01, 26.46, 26.34 (24.34 –C10), 24.64, 24.47. **HRMS** (ESI) m/z calculated for $[M+H]^+$ requires 194.1539, found 194.1541. **ATR-FTIR** (cm^{-1}): 2949, 2868, 1636, 1427.

N,N-Dimethyltricyclo[2.2.1.0^{2,6}]heptane-1-carboxamide (**35b**)

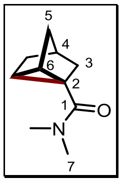

^{1}H NMR (700 MHz, $CDCl_3$) δ 3.05 (bs, 6H, C7), 2.11 (s, 1H, C4), 1.59 (s, 2H, C6), 1.53 (d, *J* = 1.4 Hz, 2H, C3), 1.46 (d, *J* = 10.7 Hz, 2H, C5b), 1.32 (d, *J* = 10.2 Hz, 2H, C5a). **^{13}C NMR** (176 MHz, CDCl3) δ 172.87 (C1), 36.39 (C7), 33.54 (C3), 31.64 (C5), 26.87 (C4), 18.54 (C6). **HRMS** (ESI) m/z calculated for $[M+Na]^+$ 188.1051 found 188.1045. **ATR-FTIR** (cm^{-1}): 2981, 2943, 2865, 2356, 1624, 1501, 1447, 1397, 1367, 1290, 1274, 1196, 1177, 1119, 1071, 1053, 922, 884, 833, 764, 731, 691, 643.

N,N-Diethyltricyclo[2.2.1.0^{2,6}]heptane-1-carboxamide (**35b**)

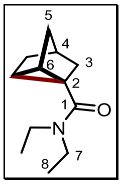

Synthesized by using **procedure IV**.

Isolated yield: 29 mg, 59%. **^{1}H NMR** (700 MHz, $CDCl_3$) δ 3.39 (s, 4H, C7), 2.10 (h, *J* = 1.5 Hz, 1H, C4), 1.60 (s, 2H, C6), 1.53 (d, *J* = 1.7 Hz, 2H, C3), 1.45 (dd, *J* = 10.4, 1.5 Hz, 2H, C5b), 1.32 (dd, *J* = 10.8, 1.6 Hz, 2H, C5a), 1.13 (s, 6H, C8). **^{13}C NMR** (176 MHz, $CDCl_3$) δ 172.03 (C1), 40.60 (by HSQC – C7), 37.00 (C3), 33.50 (C5), 31.67 (C4), 18.72 (C6), 13.46 (by HSQC – C8). **HRMS** (ESI) m/z calculated for $[M+H]^+$ requires 194.1539, found 194.1542. **ATR-FTIR** (cm^{-1}): 2922, 2851.

N,N-Dibenzyltricyclo[2.2.1.0^{2,6}]heptane-1-carboxamide (**35c**)

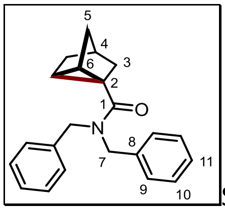

Synthesized by using **procedure IV**.

Isolated yield: 57 mg, 60%. **^{1}H NMR** (600 MHz, $CDCl_3$) δ 7.37 – 7.30 (m, 4H, C10), 7.28 (d, *J* = 6.2 Hz, 2H, C11), 7.14 (d, *J* = 7.2 Hz, 4H, C9), 4.55 (s, 4H, C7), 2.06 (s, 1H, C4), 1.73 (s, 2H, C6), 1.53 (d, *J* = 1.5 Hz, 2H, C3), 1.42 (d, *J* = 10.8 Hz, 2H, C5a), 1.32 (d, *J* = 10.2 Hz, 2H, C5b). **^{13}C NMR** (151 MHz, $CDCl_3$) δ 203.4 (by HMBC, C1) 173.50, 137.39, 128.66, 127.31, 49.4 (by HSQC, C7) 36.96 (C3), 33.31 (C5), 31.52 (C4), 19.20 (C6). **HRMS** (ESI) m/z calculated for $[M+H]^+$ requires 318.1852, found 318.1850. **ATR-FTIR** (cm^{-1}): 3062, 3027, 2939, 2864, 1633, 1495, 1452, 1419, 1360, 1305, 1275, 1143, 1075, 1029, 763, 751, 699, 667, 648.

Pyrrolidin-1-yl(tricyclo[2.2.1.0^{2,6}]heptan-1-yl)methanone (**35d**)

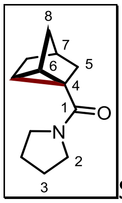

Synthesized by using **procedure IV**.

Isolated yield: 34 mg, 72%. **^{1}H NMR** (600 MHz, $CDCl_3$) δ 3.53 (t, *J* = 6.7 Hz, 2H, C2a), 3.47 (t, *J* = 6.9 Hz, 2H, C2b), 2.10 (p, *J* = 1.7 Hz, 1H, C7), 1.90 (s, 2H, C3a), 1.82 (s, 2H, C3b), 1.71 (s, 2H, C6), 1.58 (d, *J* = 1.6 Hz, 2H, C5), 1.40 (dd, *J* = 10.3, 1.4 Hz, 2H, C8a), 1.30 (dd, *J* = 10.8, 1.6 Hz, 2H, C8b). **^{13}C NMR** (151 MHz, $CDCl_3$) δ 171.59 (C1), 46.96 (C2), 46.90 (C2), 35.57 (C5), 33.33 (C8), 32.05 (C7), 31.76 (C3), 24.04 (C3), 20.26 (C6). **HRMS** (ESI) m/z calculated for $[M+H]^+$ requires 192.1383, found 192.1429. **ATR-FTIR** (cm^{-1}): 2913, 2849, 1658, 1460, 1387, 1283, 1141, 1119, 785, 760.

N,N-Diethylbicyclo[2.2.1]hept-2-ene-2-carboxamide (**36**)

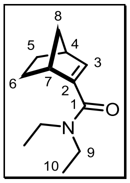

Synthesized by using **procedure IV**.

Isolated yield: 21 mg, 10%. ^{1}**H NMR** (600 MHz,$CDCl_3$) δ 6.12 (d, *J* = 3.0 Hz, 1H, C3), 3.48 (t, *J* = 8.9 Hz, 2H, C9a, C9b), 3.33 (dq, *J* = 13.8, 6.9 Hz, 2H, C9a', C9b'), 3.13 (dq, *J* = 2.6, 1.4 Hz, 1H, C7), 2.98 (dp, *J* = 3.2, 1.6 Hz, 1H, C4), 1.77 – 1.69 (m, 2H, C8), 1.44 – 1.36 (m, 2H, C6a, C5a), 1.15 (t, *J* = 7.1 Hz, 8H, C10, C5b , C6b). ^{13}**C NMR** (151 MHz, $CDCl_3$) δ 168.63 (C1), 143.31 (C2), 135.88 (C3), 47.42 (C6), 45.50 (C7), 43.58 (C4), 42.66 (C9), 39.42 (C9), 25.54 (C8), 25.41 (C5), 14.85 (C10), 13.02 (C10). **HRMS** (ESI) m/z calculated for $[M+H]^+$ requires 194.1539, found 194.1541. **ATR-FTIR** (cm^{-1}): 2969, 2935, 2871, 1619, 1584, 1472, 1423, 1363, 1221, 1150, 1064, 874, 837, 812, 743.

5) NMR spectra

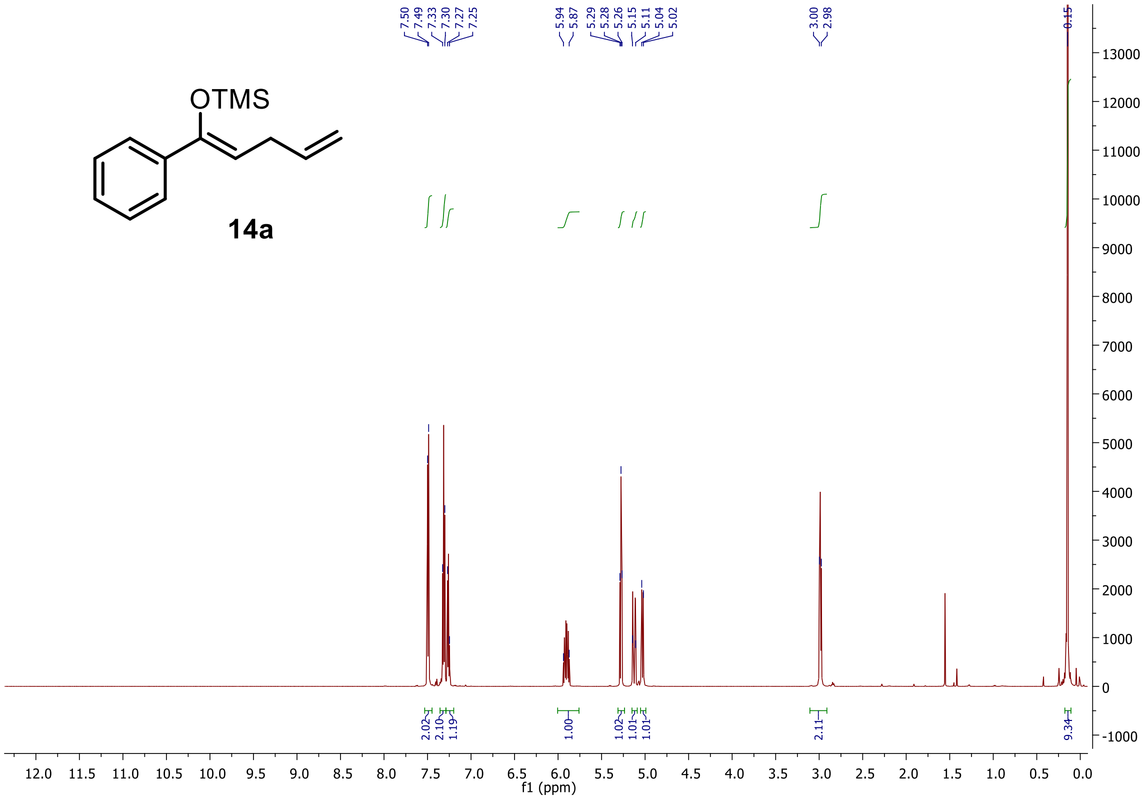

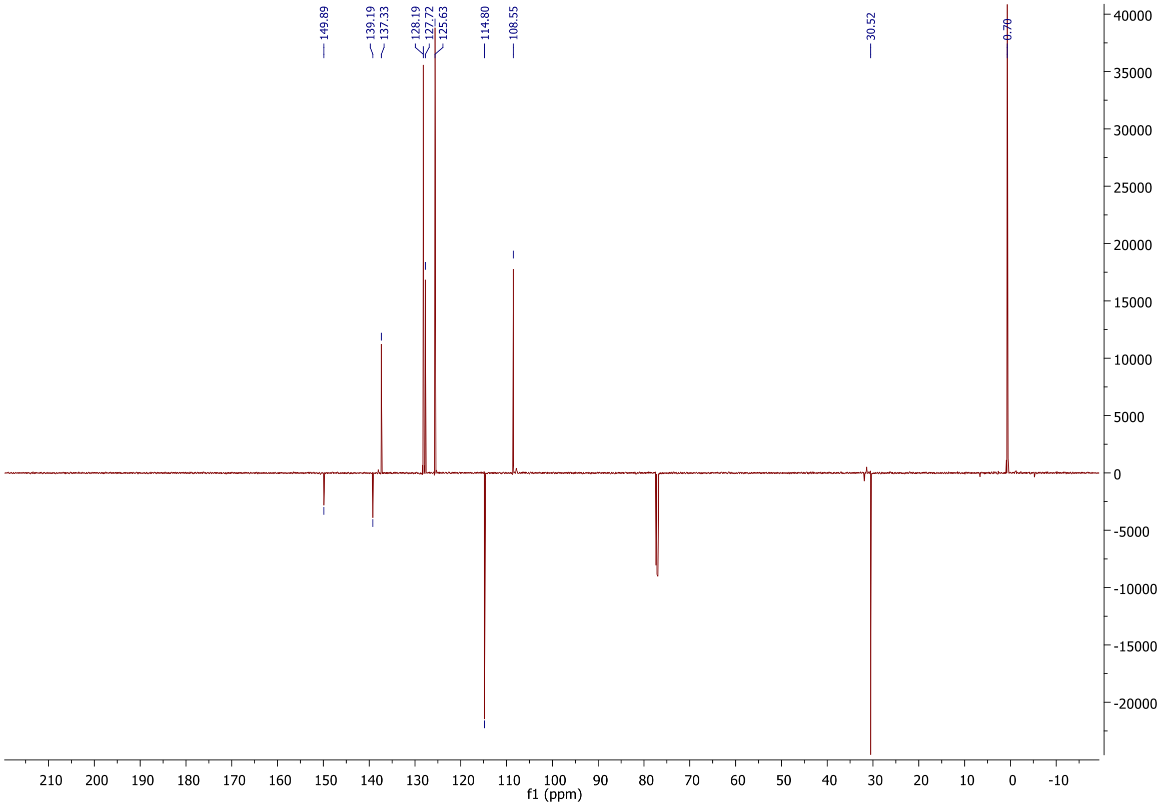

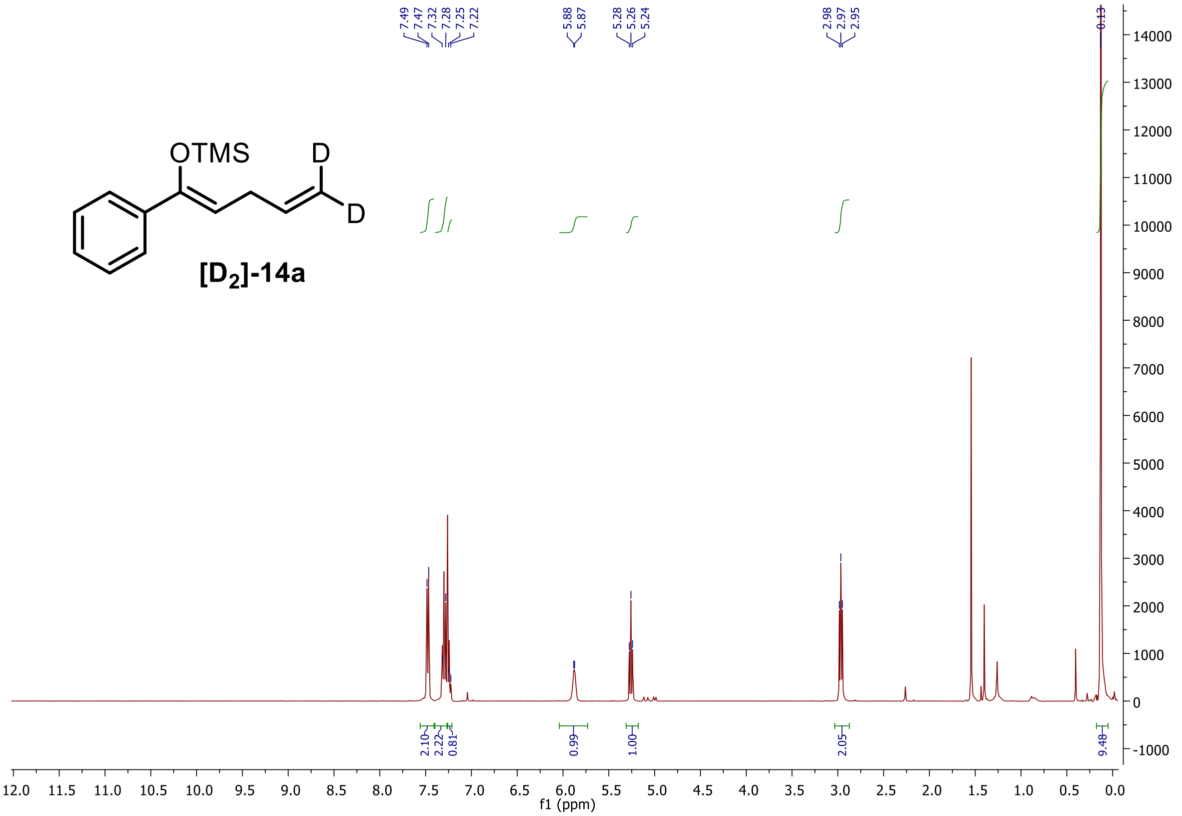
OTMS
D
D
7.49
7.47
7.32
7.28
7.25
7.22
5.88
5.87
5.28
5.26
5.24
2.98
2.97
2.95
0.13
2.10
2.22
0.81
0.99
1.00
2.05
9.48
f1 (ppm)

[D$_2$]-14a

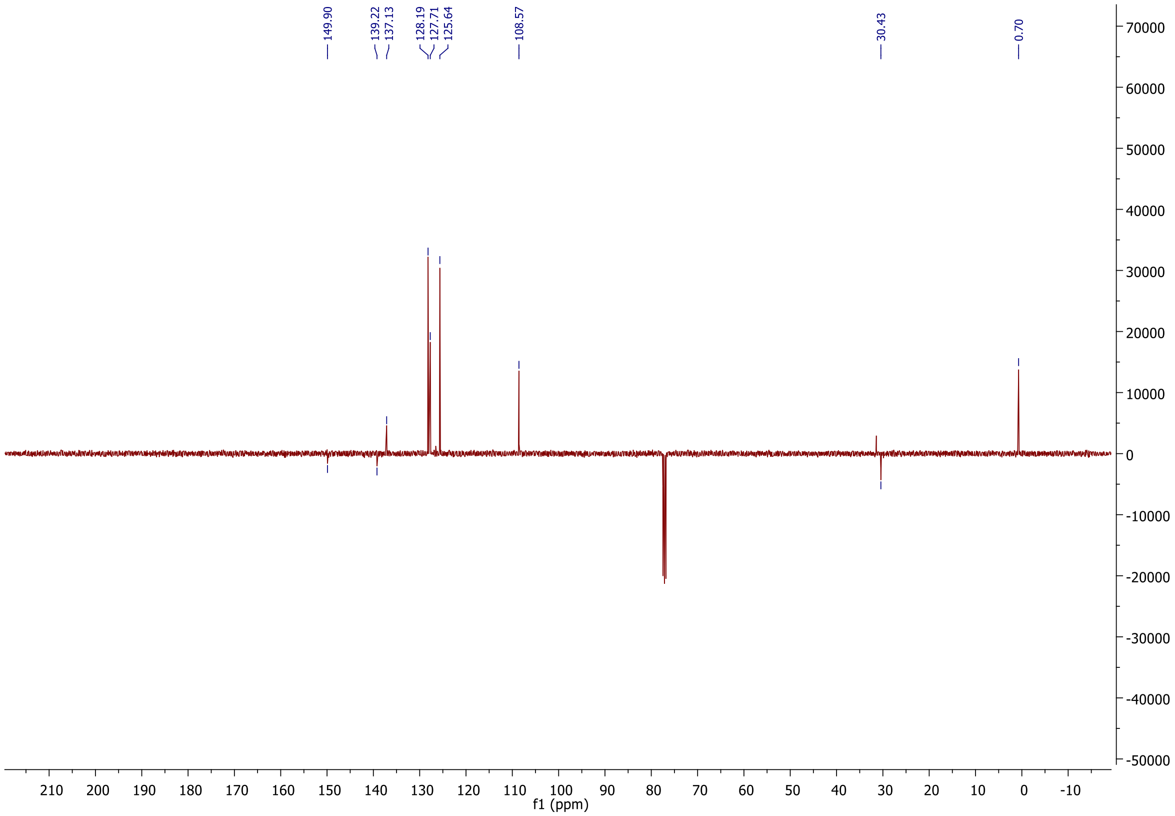
149.90
139.22
137.13
128.19
127.71
125.64
108.57
30.43
0.70
f1 (ppm)

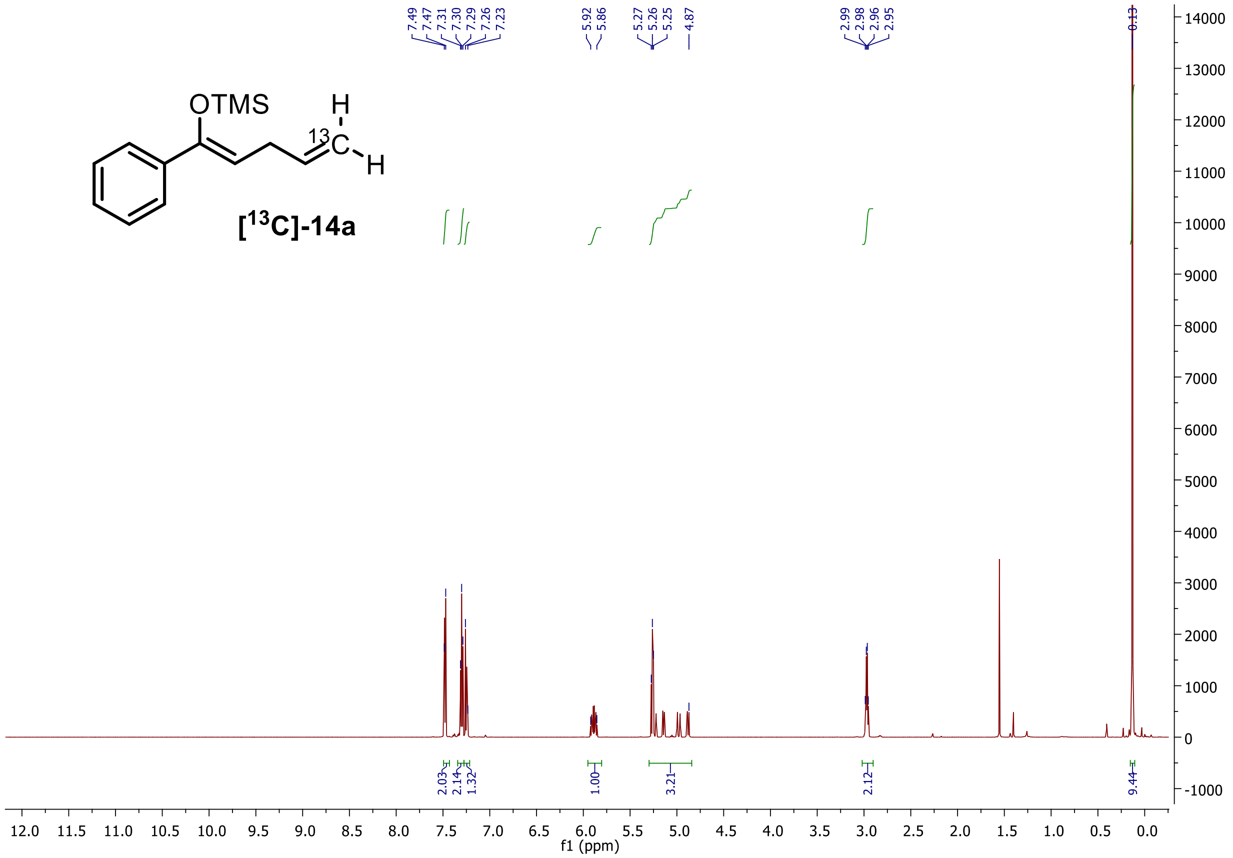
OTMS
H
13C
H
[13C]-14a
7.49
7.47
7.31
7.30
7.29
7.26
7.23
5.92
5.86
5.27
5.26
5.25
4.87
2.99
2.98
2.96
2.95
0.13
2.03
2.14
1.32
1.00
3.21
2.12
9.44
f1 (ppm)

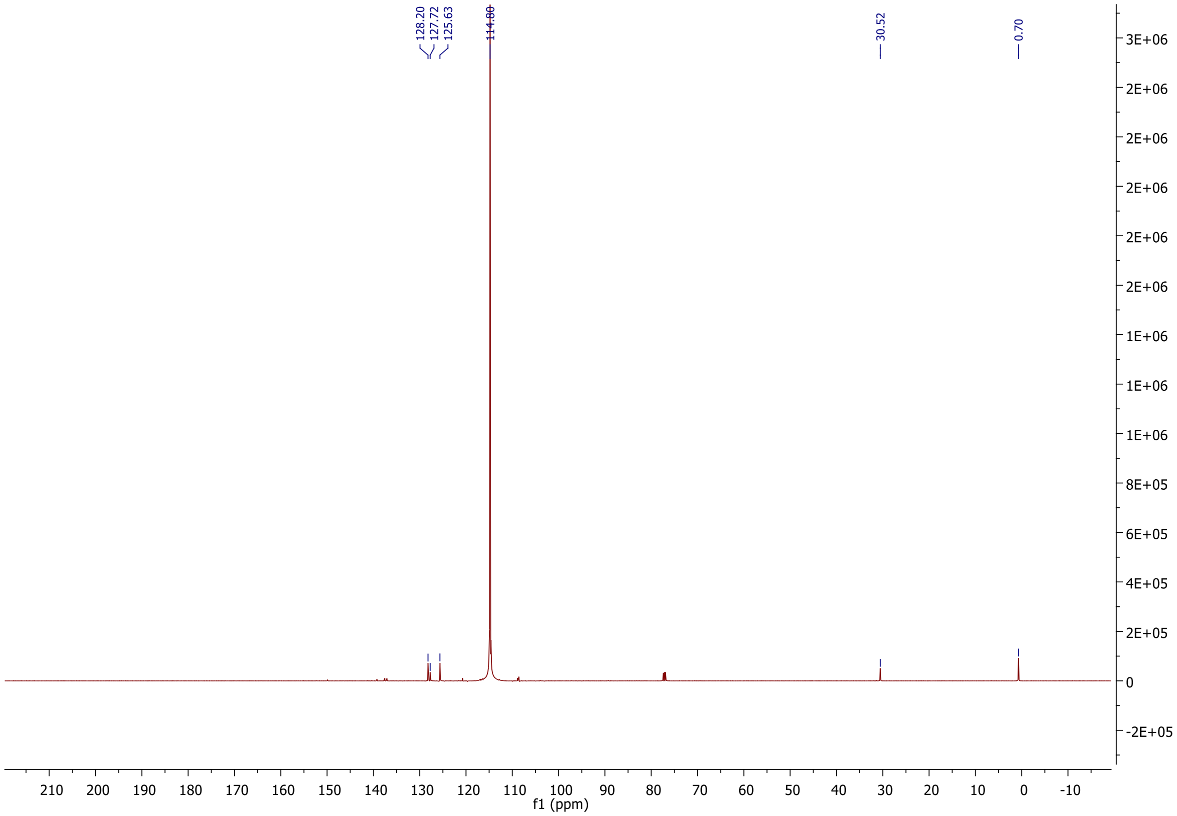
128.20
127.72
125.63
114.80
30.52
0.70
f1 (ppm)

14b

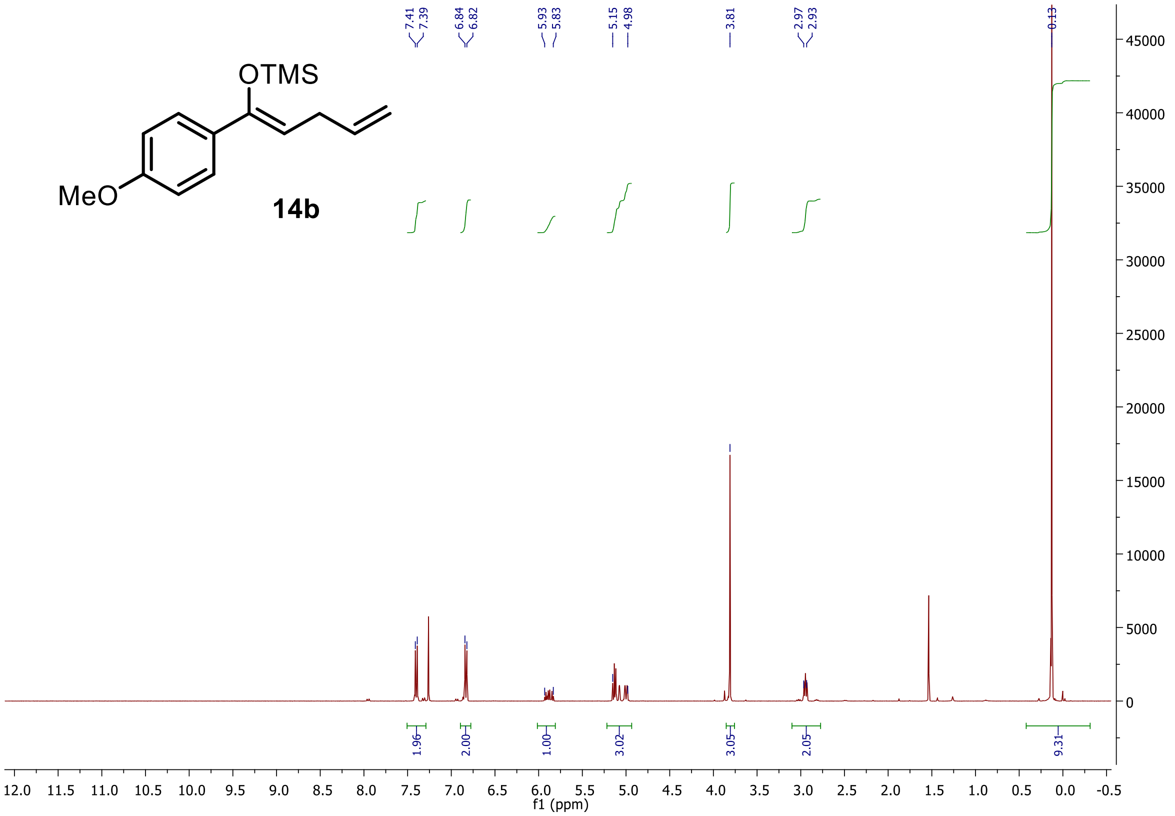
OTMS
MeO
7.41
7.39
6.84
6.82
5.93
5.83
5.15
4.98
3.81
2.97
2.93
0.13
1.96
2.00
1.00
3.02
3.05
2.05
9.31
f1 (ppm)

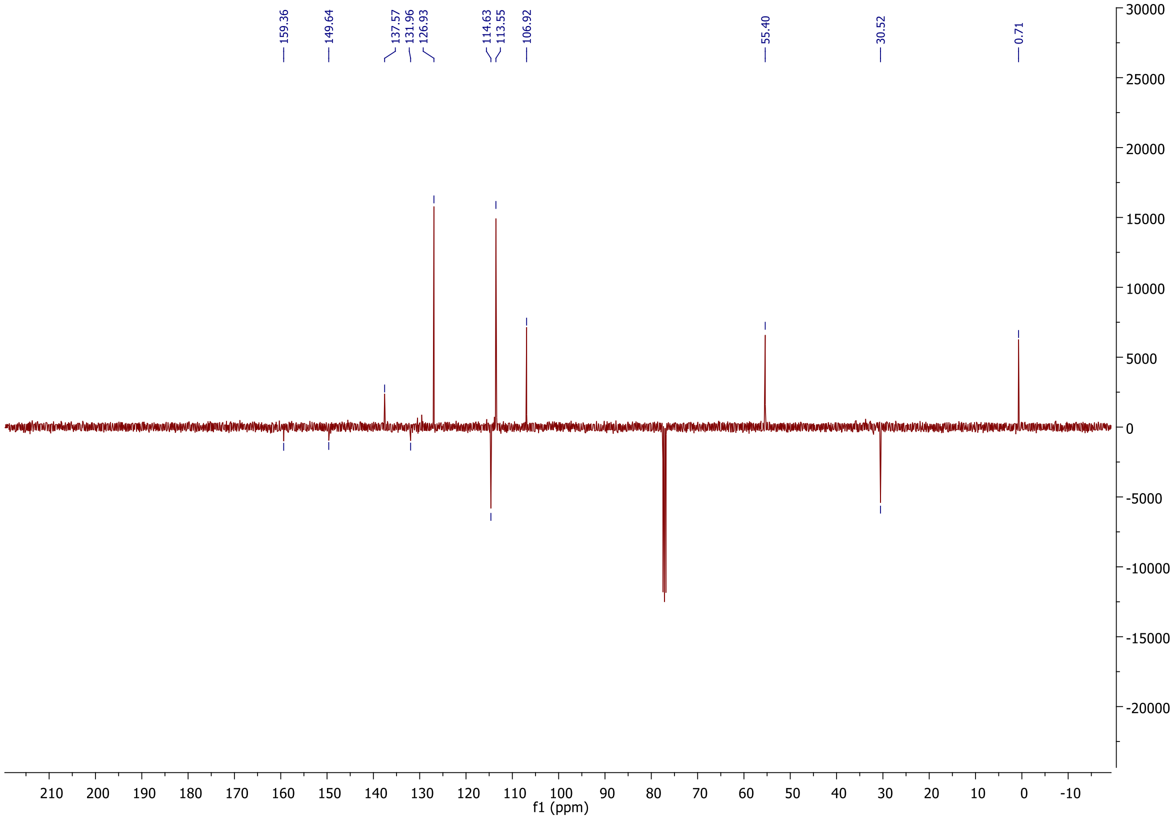
159.36
149.64
137.57
131.96
126.93
114.63
113.55
106.92
55.40
30.52
0.71
f1 (ppm)

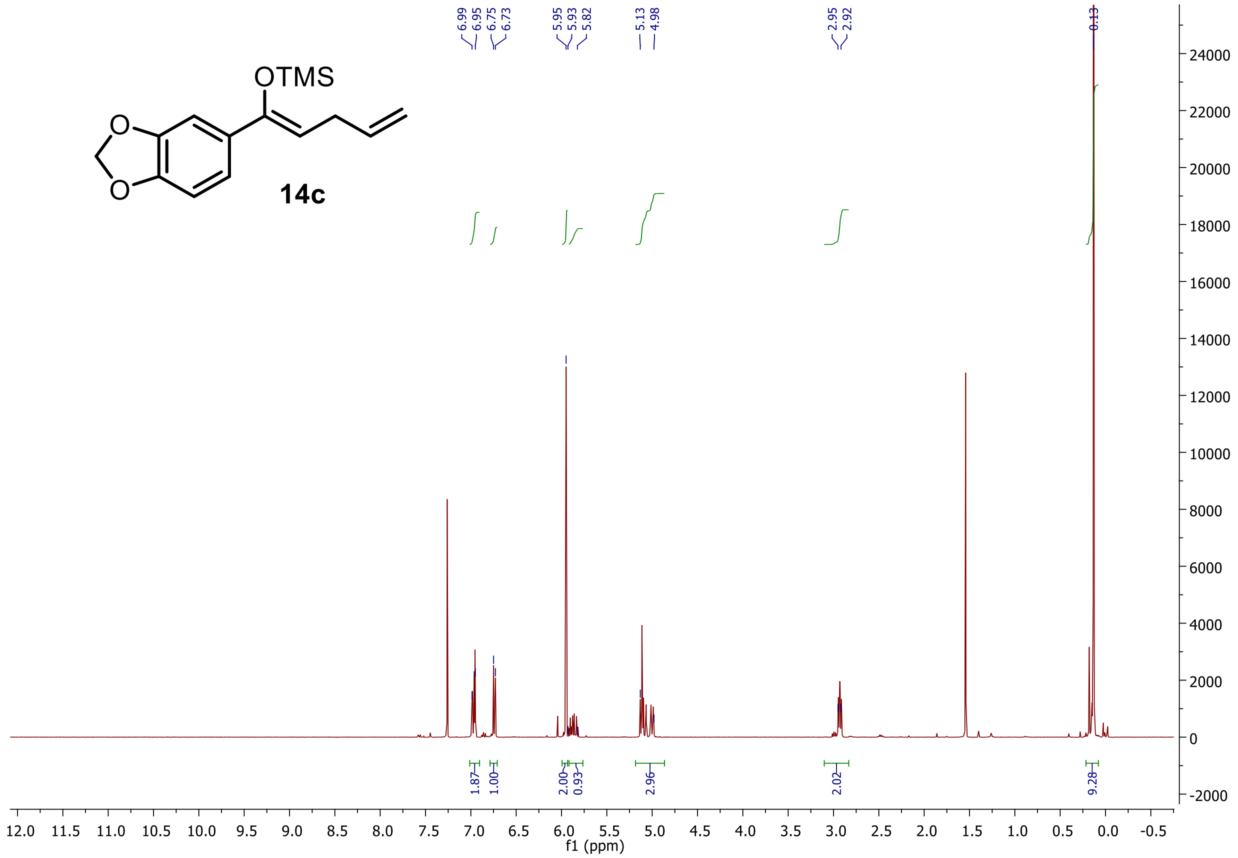
OTMS
O
O
14c
6.99
6.95
6.75
6.73
5.95
5.93
5.82
5.13
4.98
2.95
2.92
0.13
1.87
1.00
2.00
0.93
2.96
2.02
9.28
f1 (ppm)

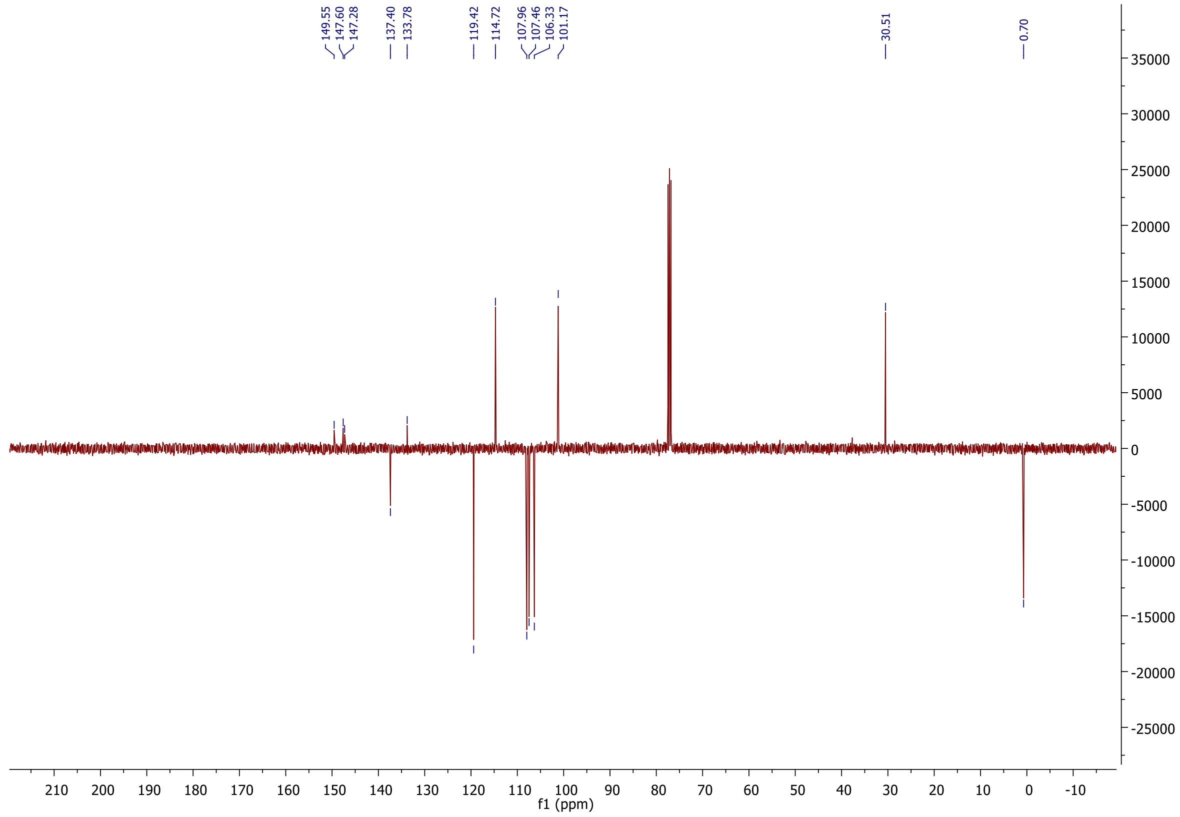
149.55
147.60
147.28
137.40
133.78
119.42
114.72
107.96
107.46
106.33
101.17
30.51
0.70
f1 (ppm)

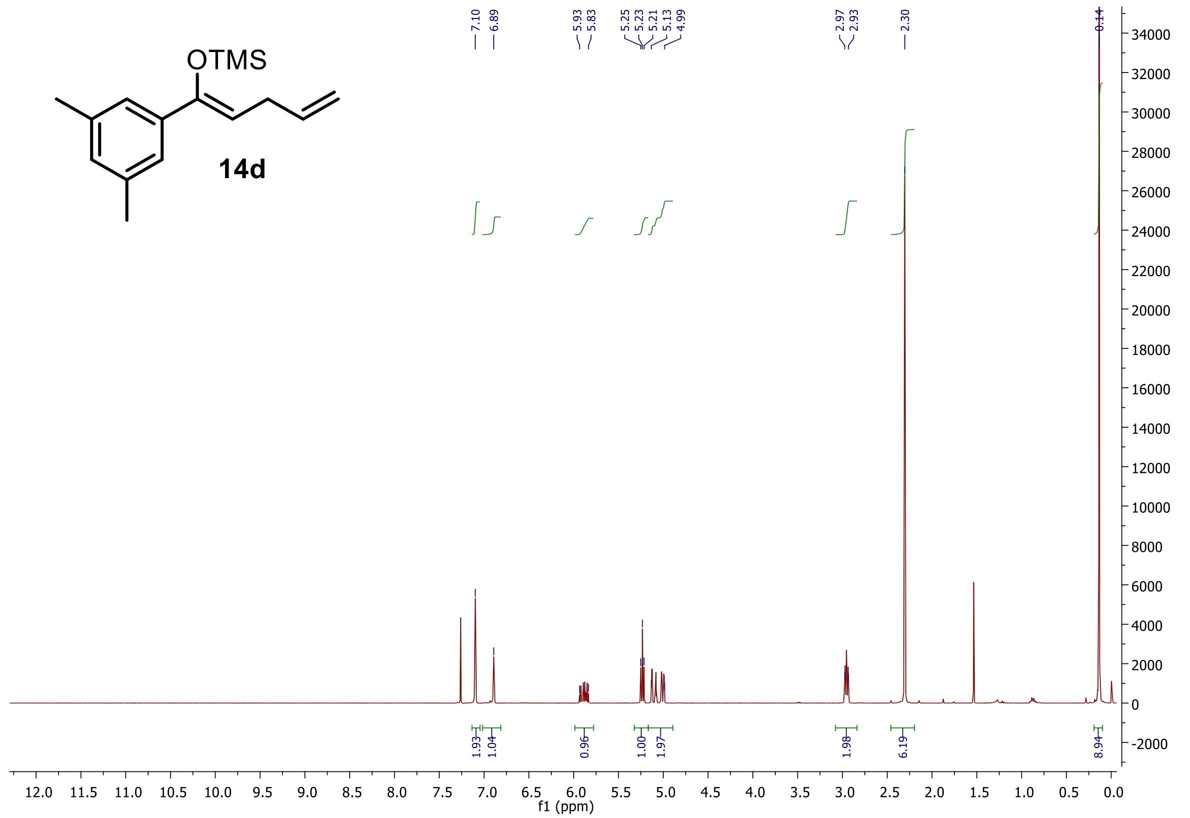

14d

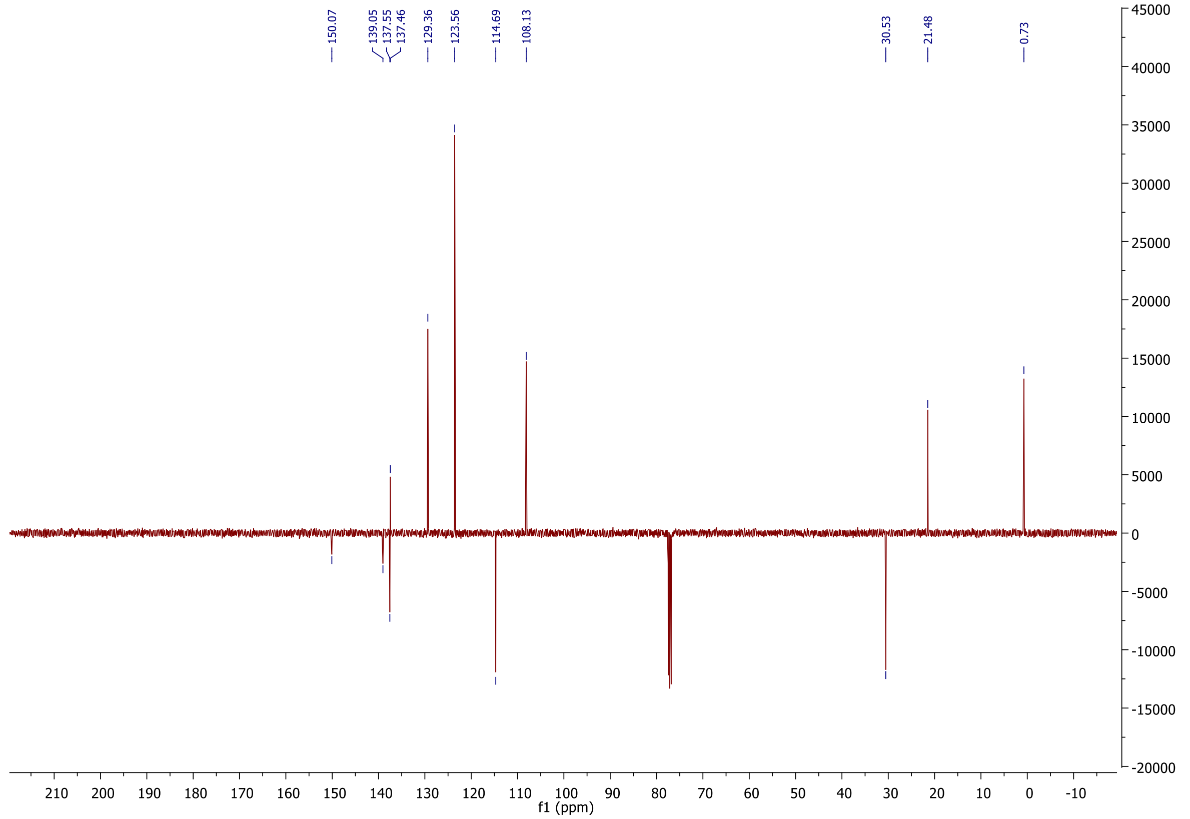

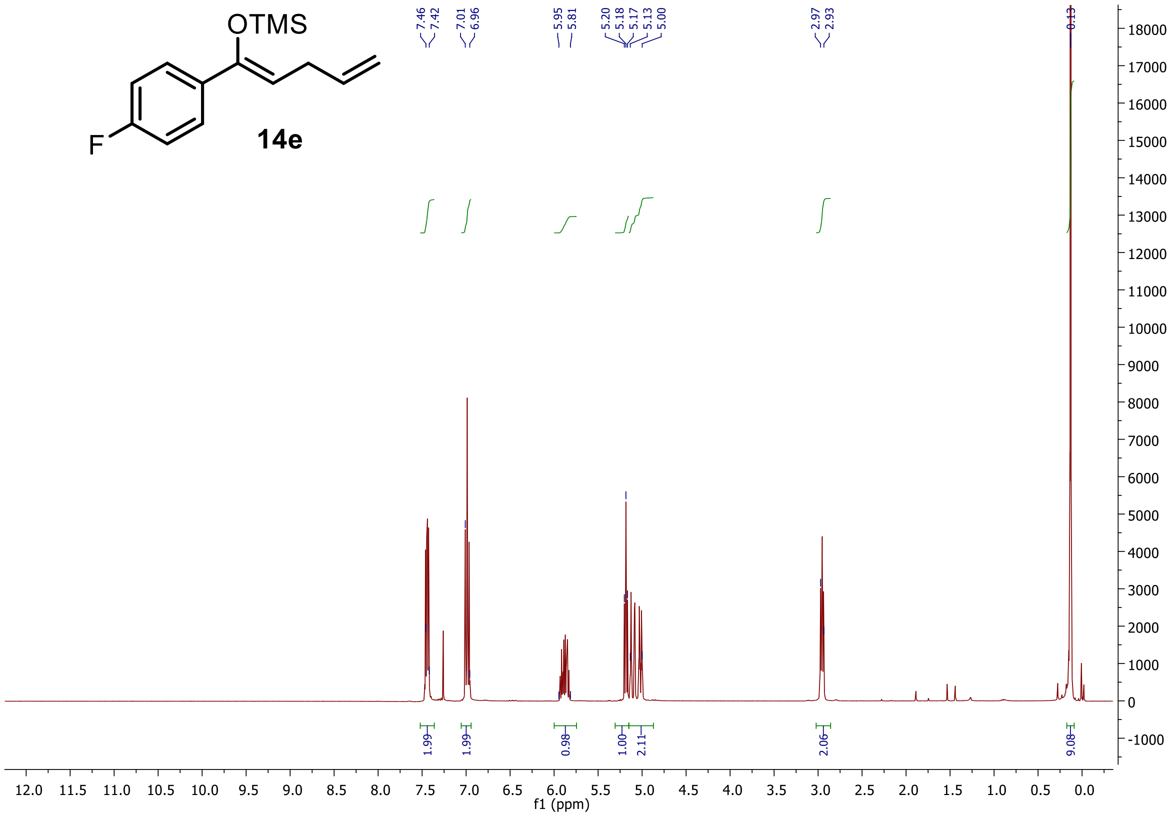
OTMS
F
14e
7.46
7.42
7.01
6.96
5.95
5.81
5.20
5.18
5.17
5.13
5.00
2.97
2.93
0.13
1.99
1.99
0.98
1.00
2.11
2.06
9.08
f1 (ppm)

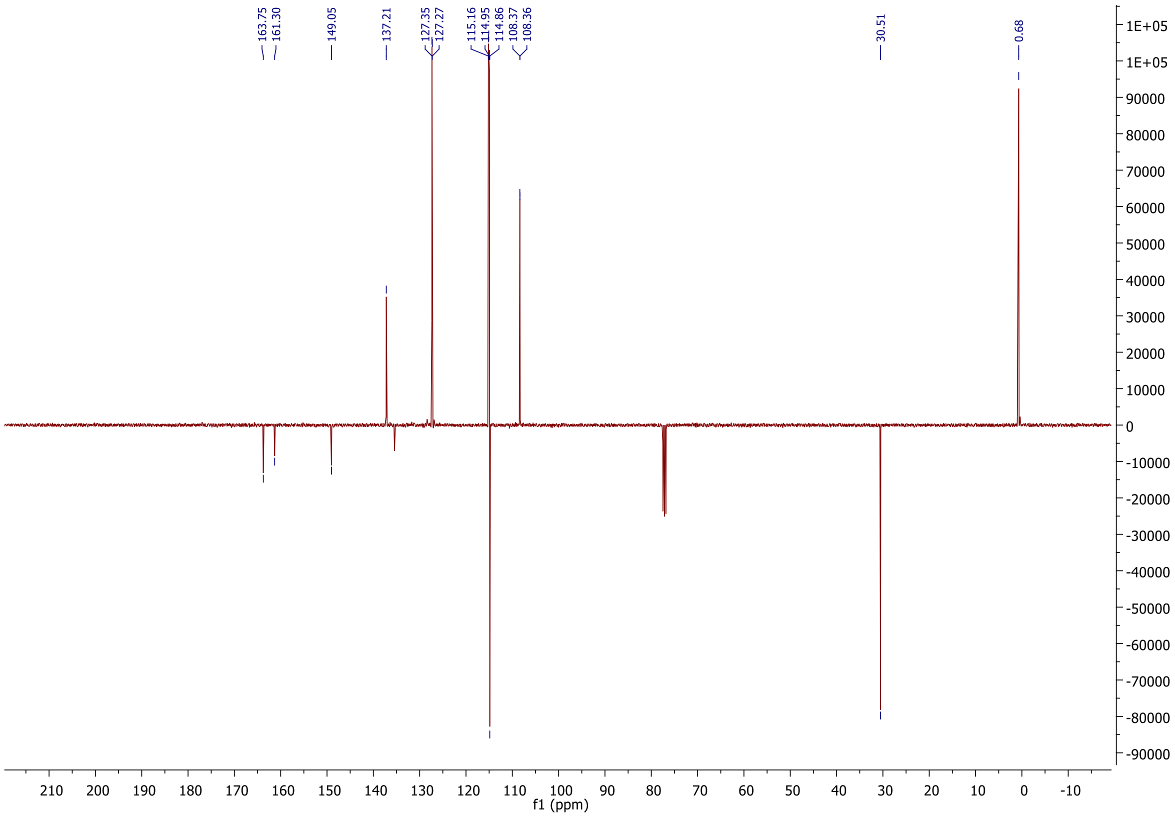
163.75
161.30
149.05
137.21
127.35
127.27
115.16
114.95
114.86
108.37
108.36
30.51
0.68
f1 (ppm)

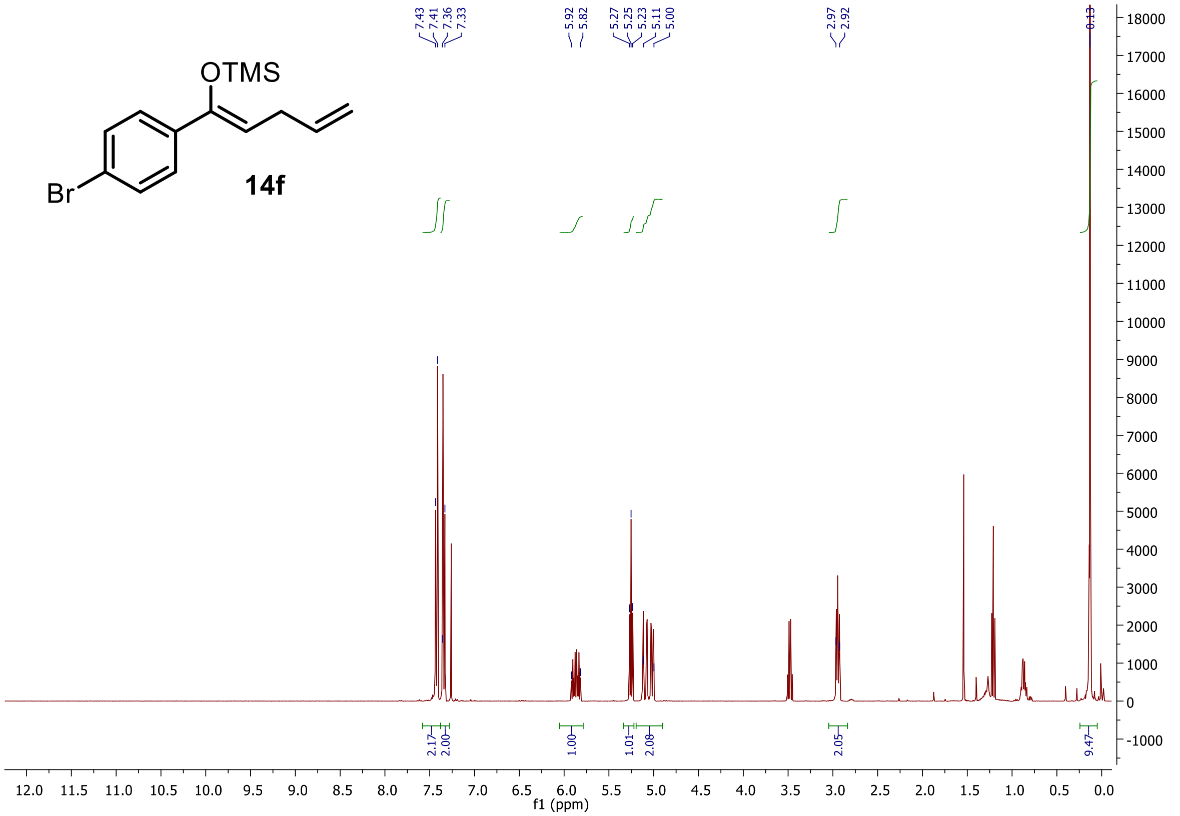
OTMS
Br
14f
7.43
7.41
7.36
7.33
5.92
5.82
5.27
5.25
5.23
5.11
5.00
2.97
2.92
0.13
2.17
2.00
1.00
1.01
2.08
2.05
9.47
f1 (ppm)

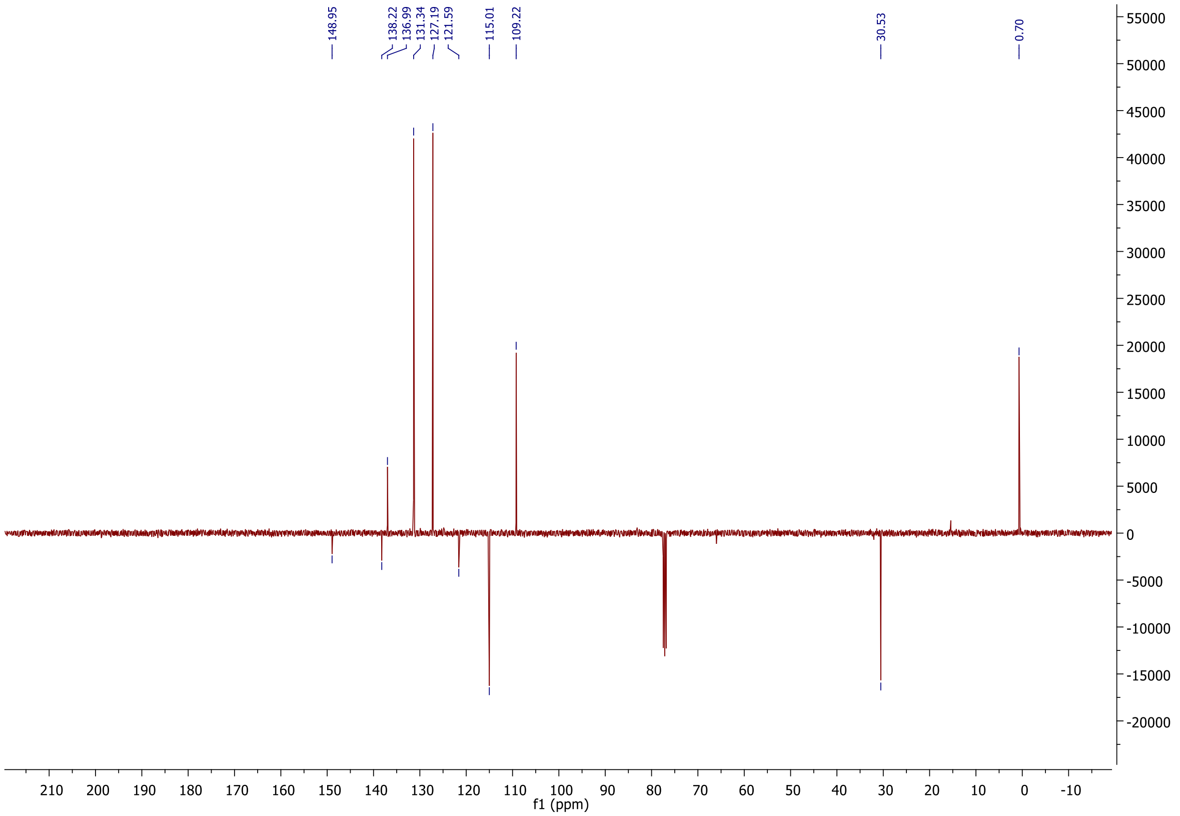
148.95
138.22
136.99
131.34
127.19
121.59
115.01
109.22
30.53
0.70
f1 (ppm)

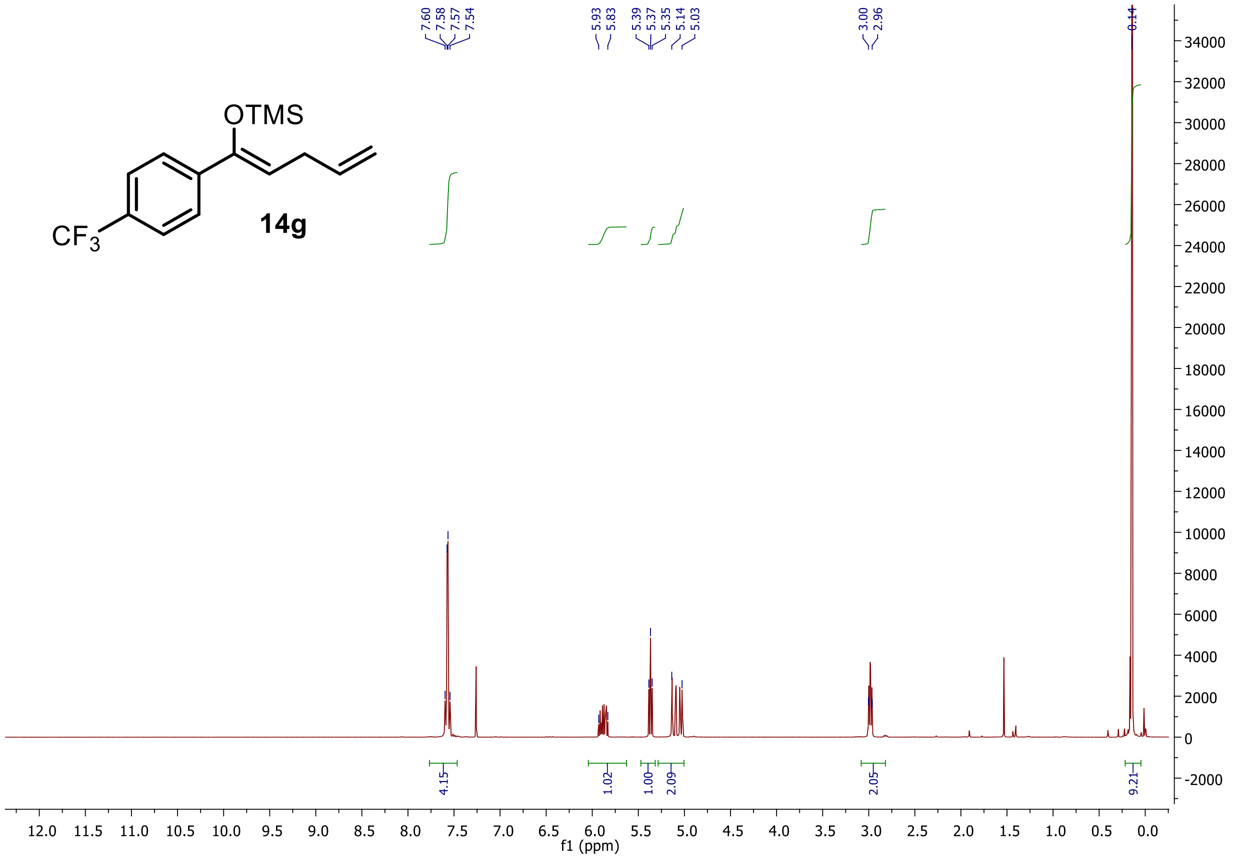
OTMS
CF3
14g
7.60
7.58
7.57
7.54
5.93
5.83
5.39
5.37
5.35
5.14
5.03
3.00
2.96
0.14
4.15
1.02
1.00
2.09
2.05
9.21
f1 (ppm)

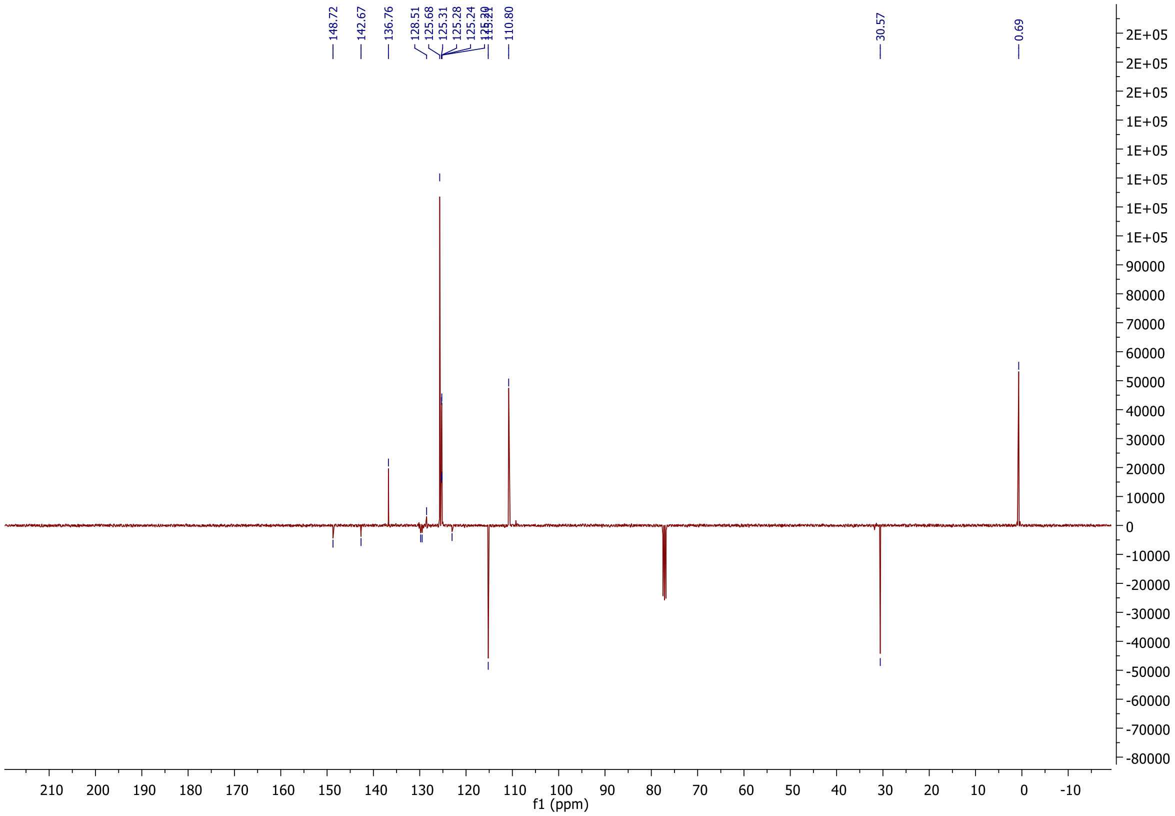
148.72
142.67
136.76
128.51
125.68
125.31
125.28
125.24
110.80
30.57
0.69
f1 (ppm)

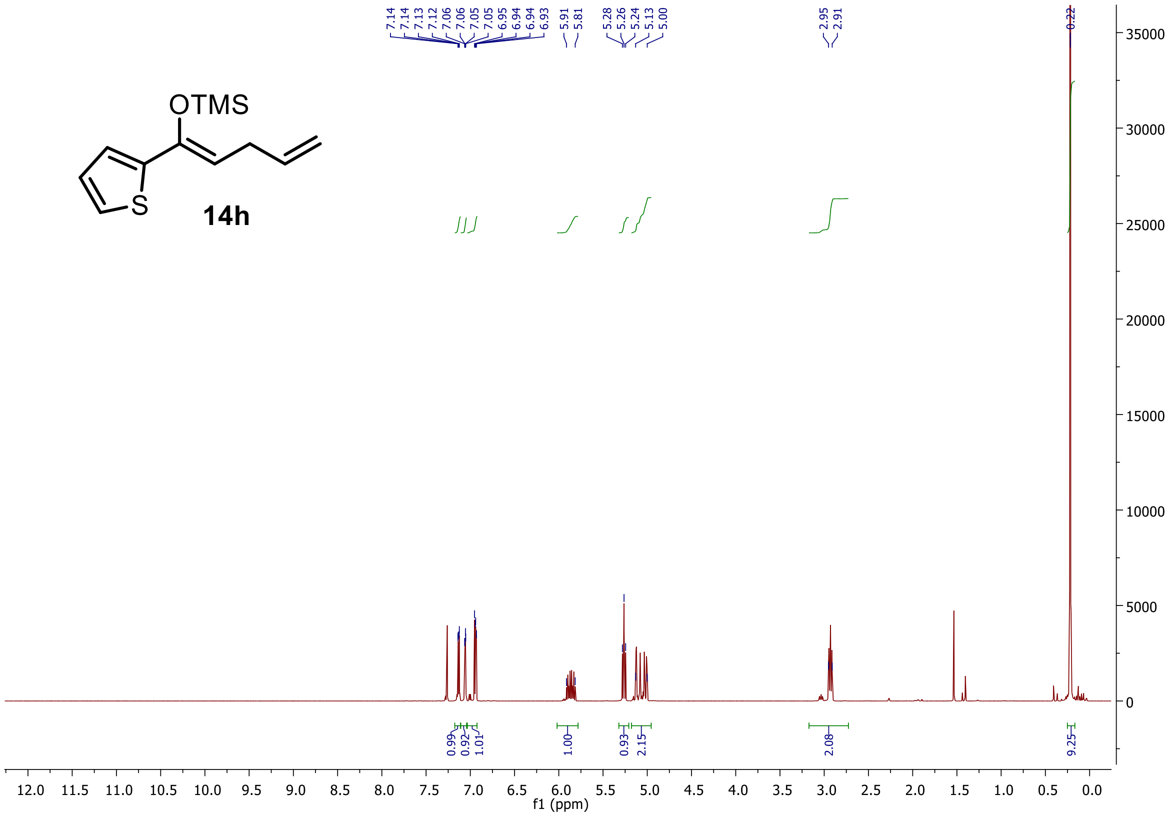
OTMS
S
14h
7.14 7.14 7.13 7.12 7.06 7.06 7.05 7.05 6.95 6.94 6.94 6.93
5.91 5.81
5.28 5.26 5.24 5.13 5.00
2.95 2.91
0.22
0.99 0.92 1.01 1.00 0.93 2.15 2.08 9.25
f1 (ppm)

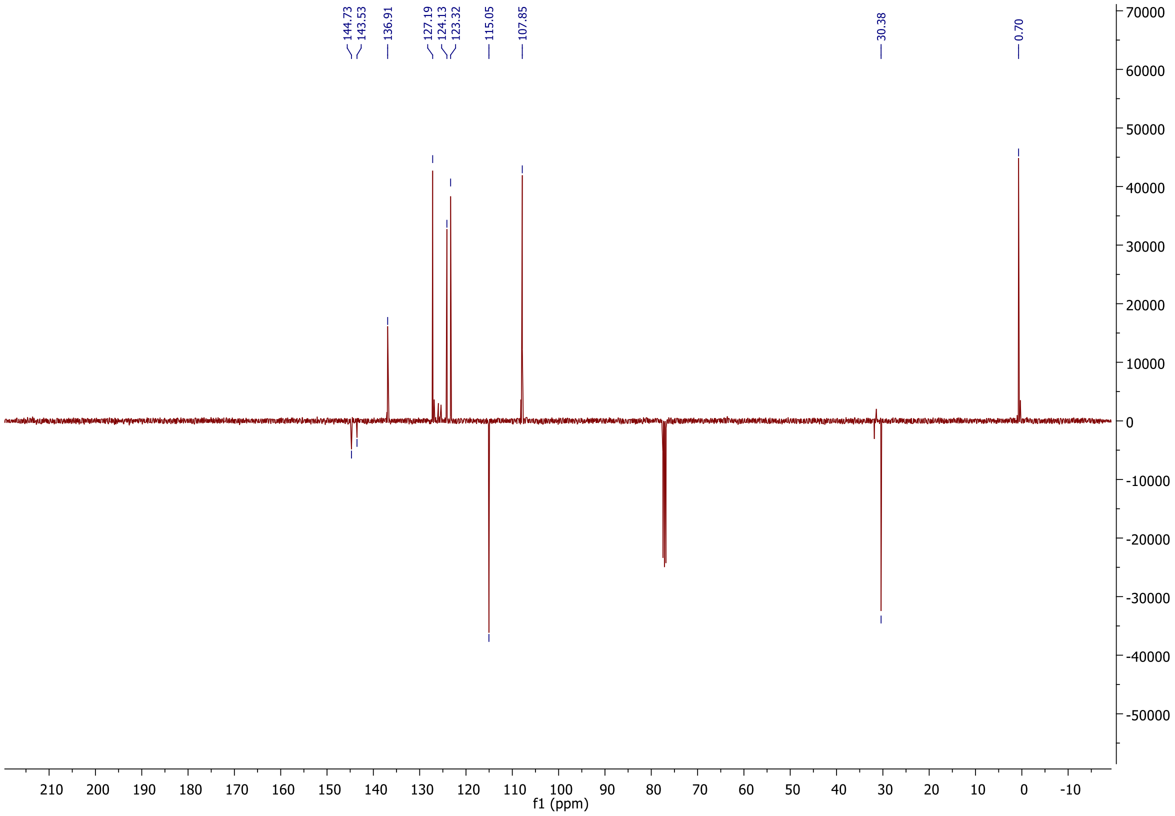
144.73 143.53 136.91 127.19 124.13 123.32 115.05 107.85 30.38 0.70
f1 (ppm)

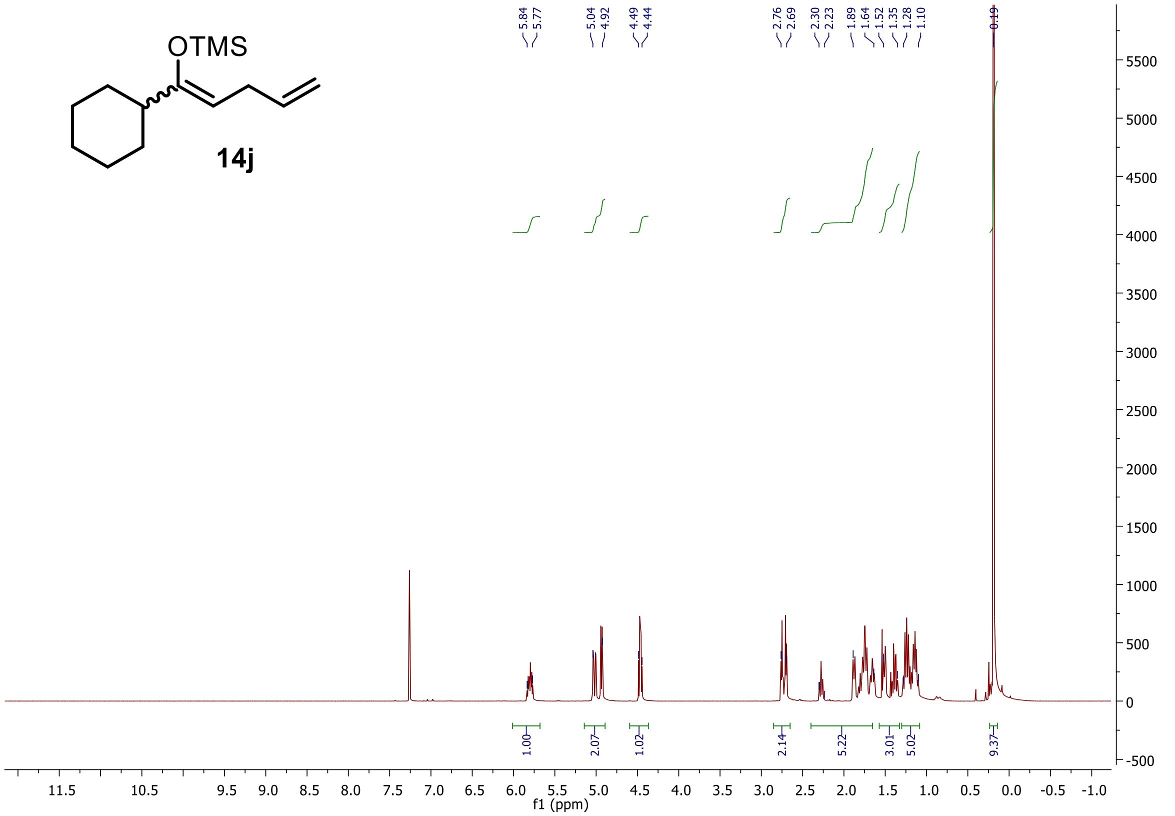
OTMS
14j
5.84
5.77
5.04
4.92
4.49
4.44
2.76
2.69
2.30
2.23
1.89
1.64
1.52
1.35
1.28
1.10
0.19
1.00
2.07
1.02
2.14
5.22
3.01
5.02
9.37
f1 (ppm)

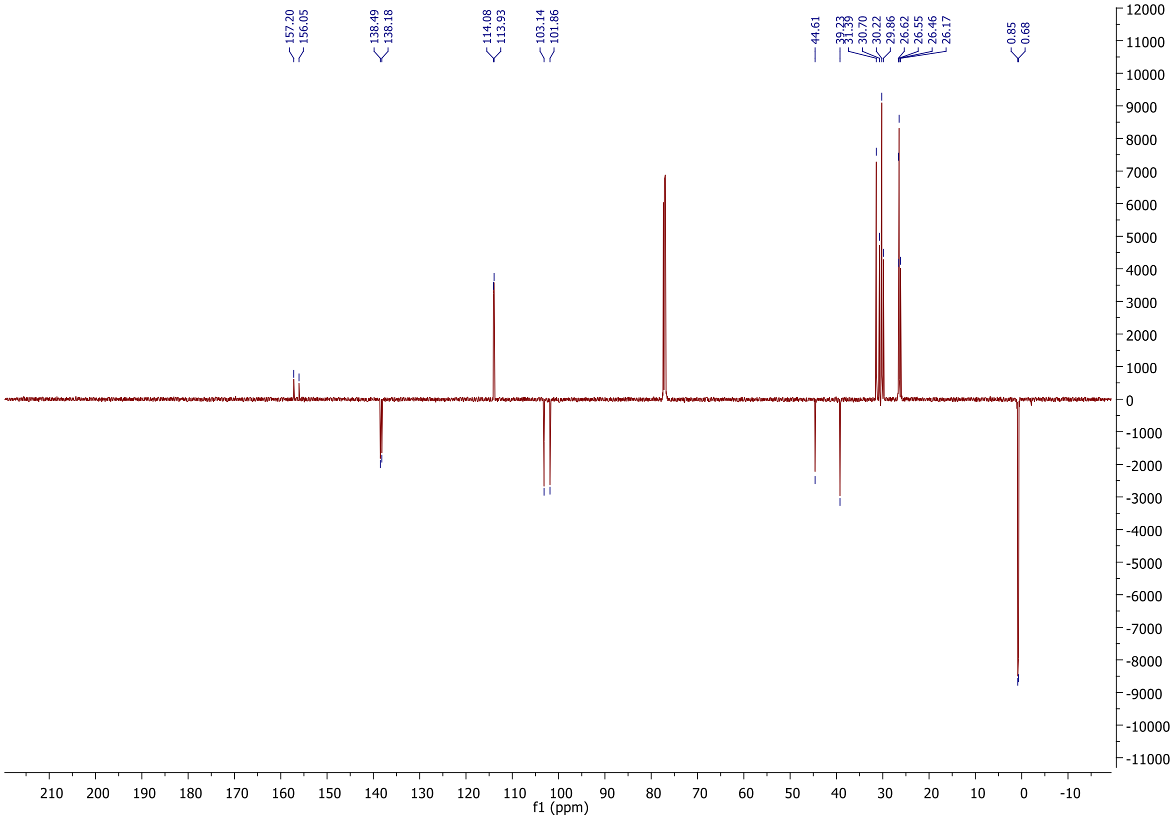
157.20
156.05
138.49
138.18
114.08
113.93
103.14
101.86
44.61
39.23
31.39
30.70
30.22
29.86
26.62
26.55
26.46
26.17
0.85
0.68
f1 (ppm)

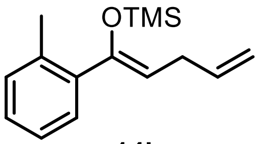
OTMS

14k

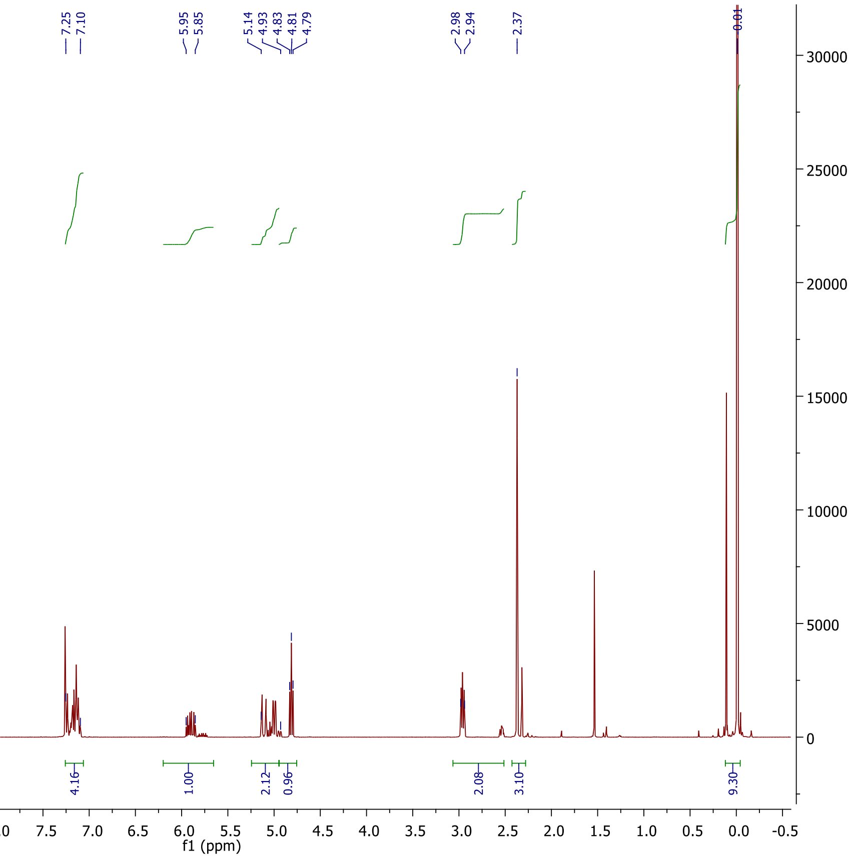
7.25
7.10
5.95
5.85
5.14
4.93
4.83
4.81
4.79
2.98
2.94
2.37
0.01
4.16
1.00
2.12
0.96
2.08
3.10
9.30
f1 (ppm)

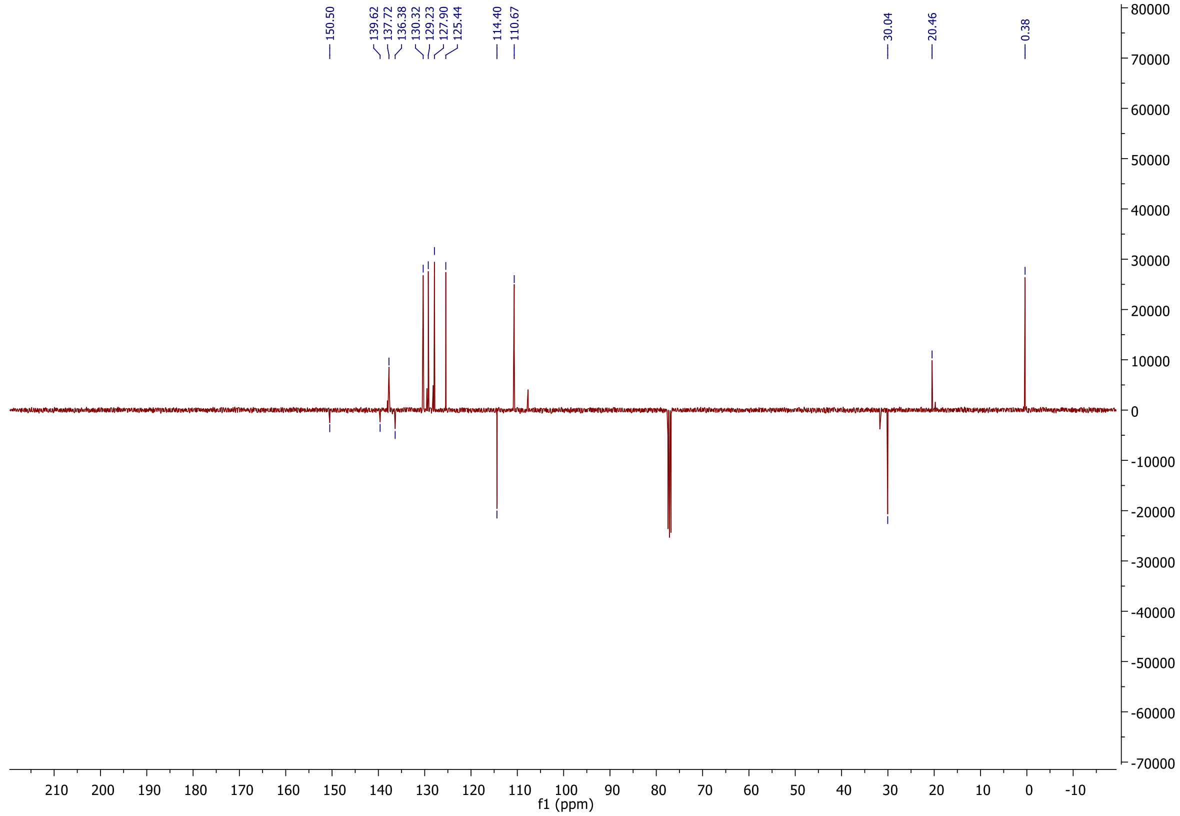
150.50
139.62
137.72
136.38
130.32
129.23
127.90
125.44
114.40
110.67
30.04
20.46
0.38
f1 (ppm)

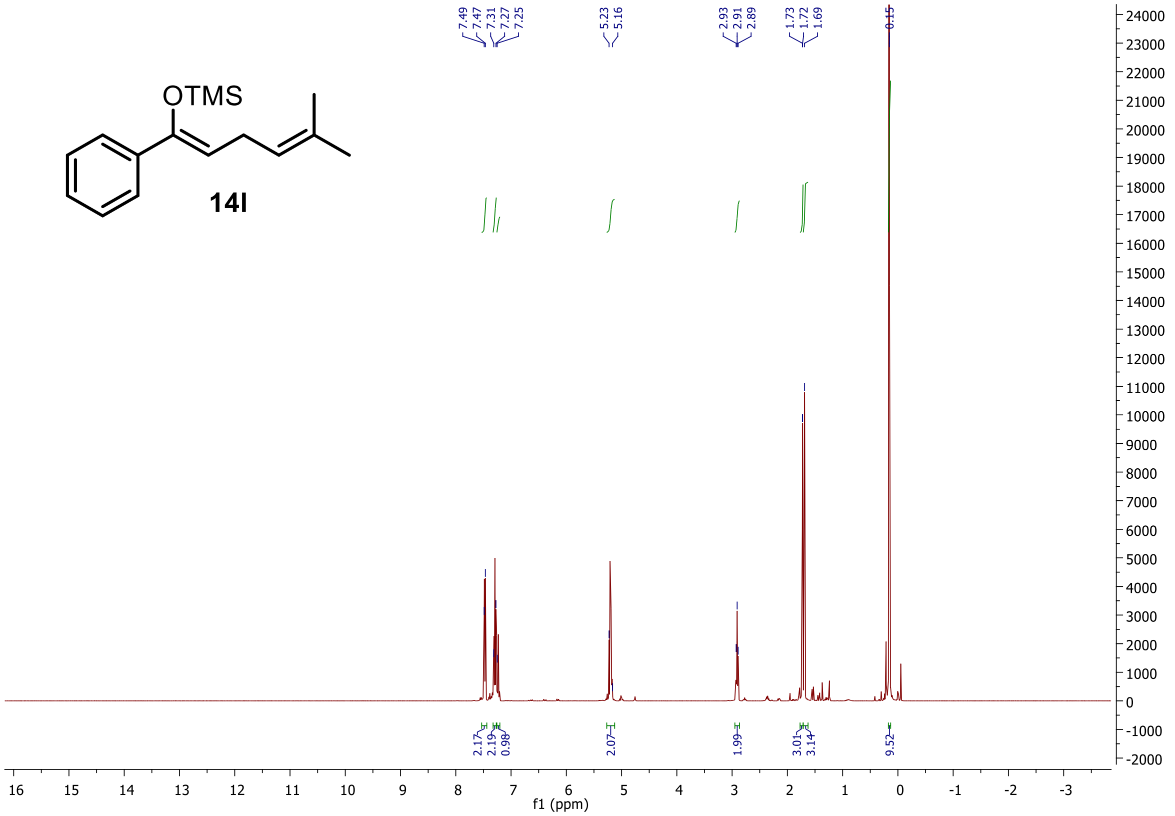

14l

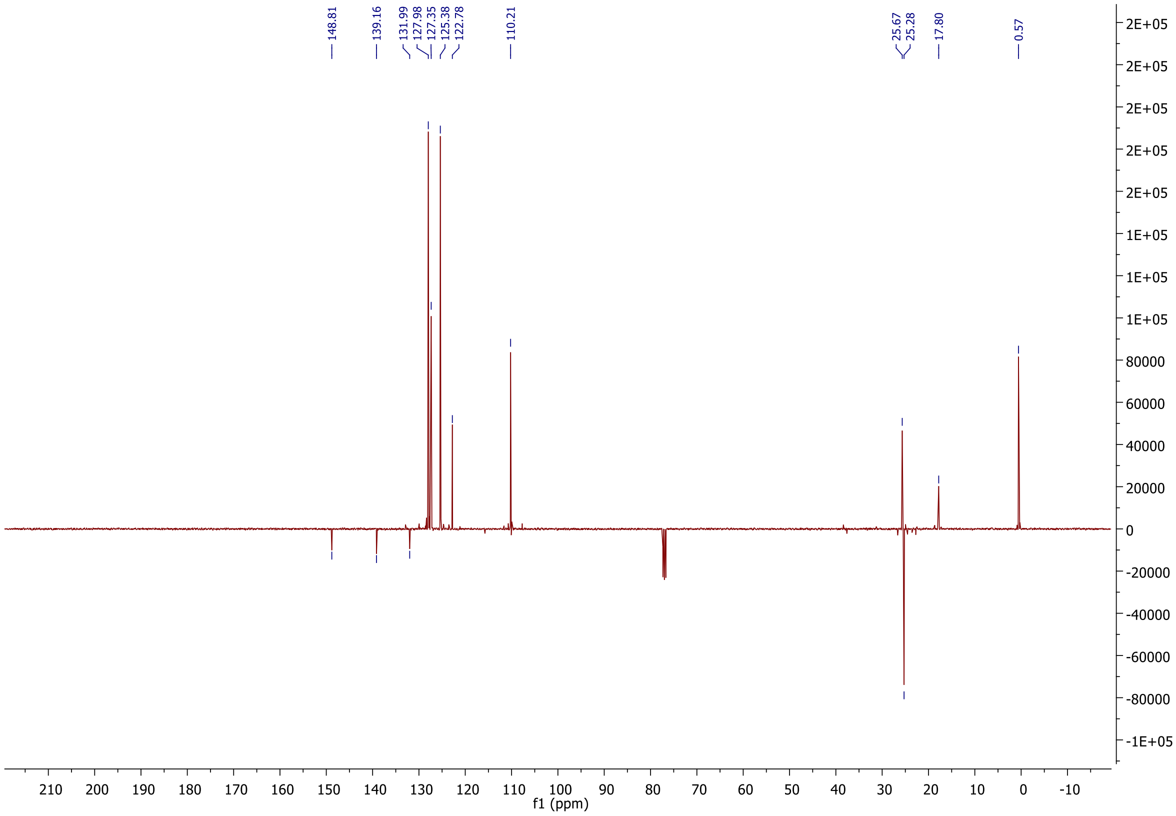

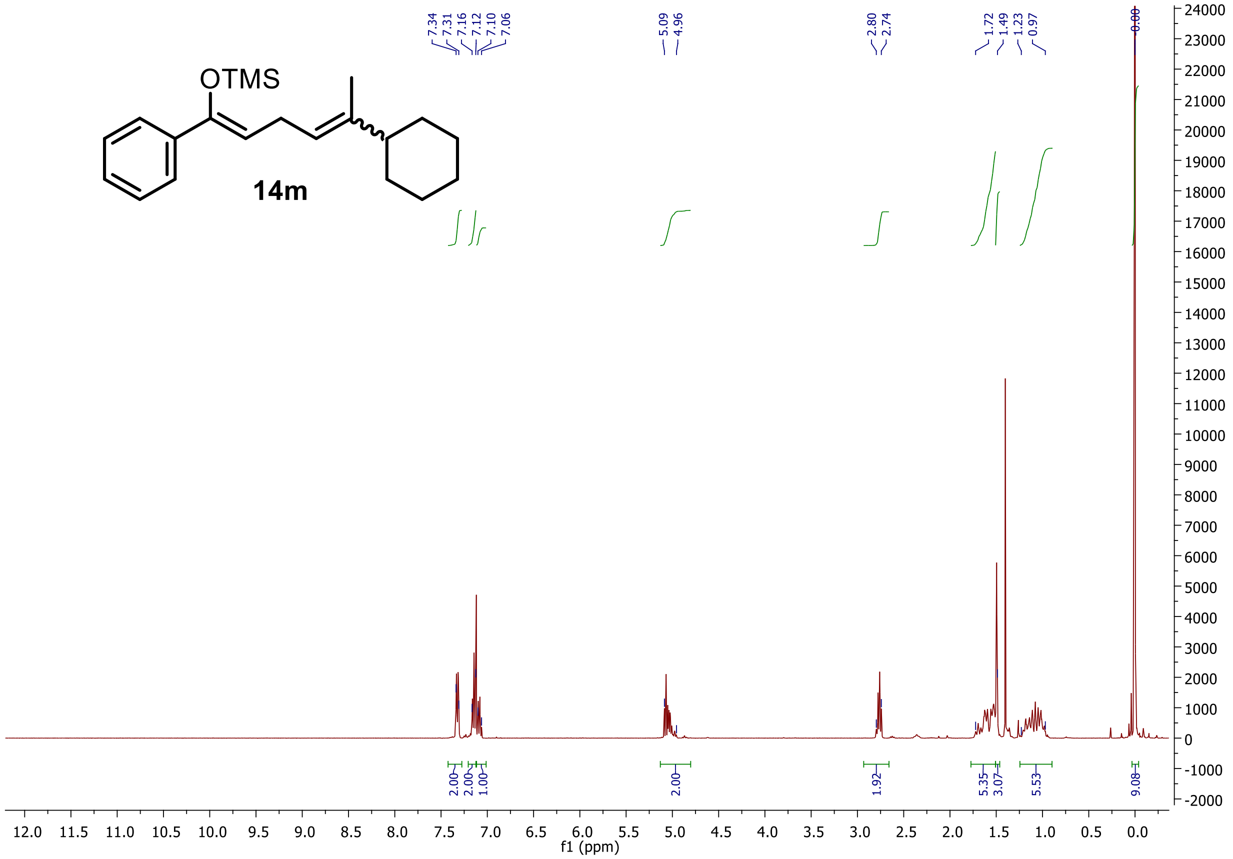
OTMS
14m

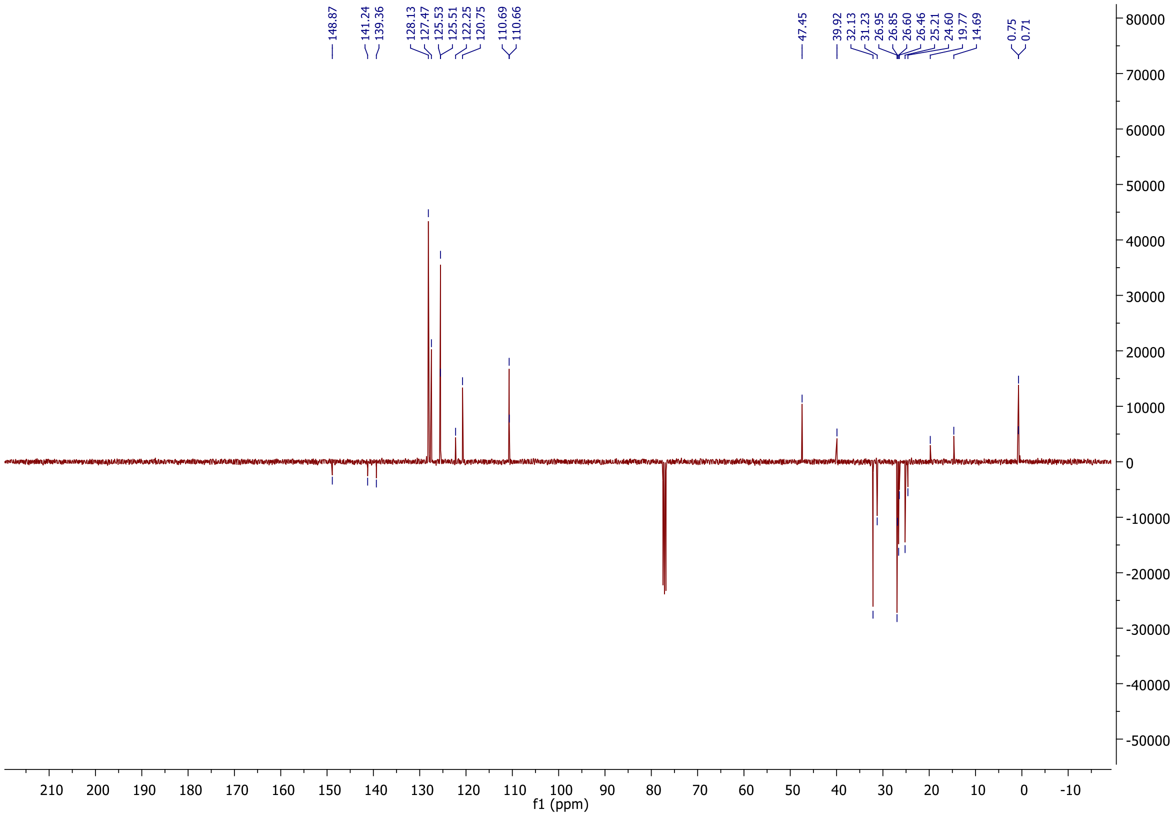

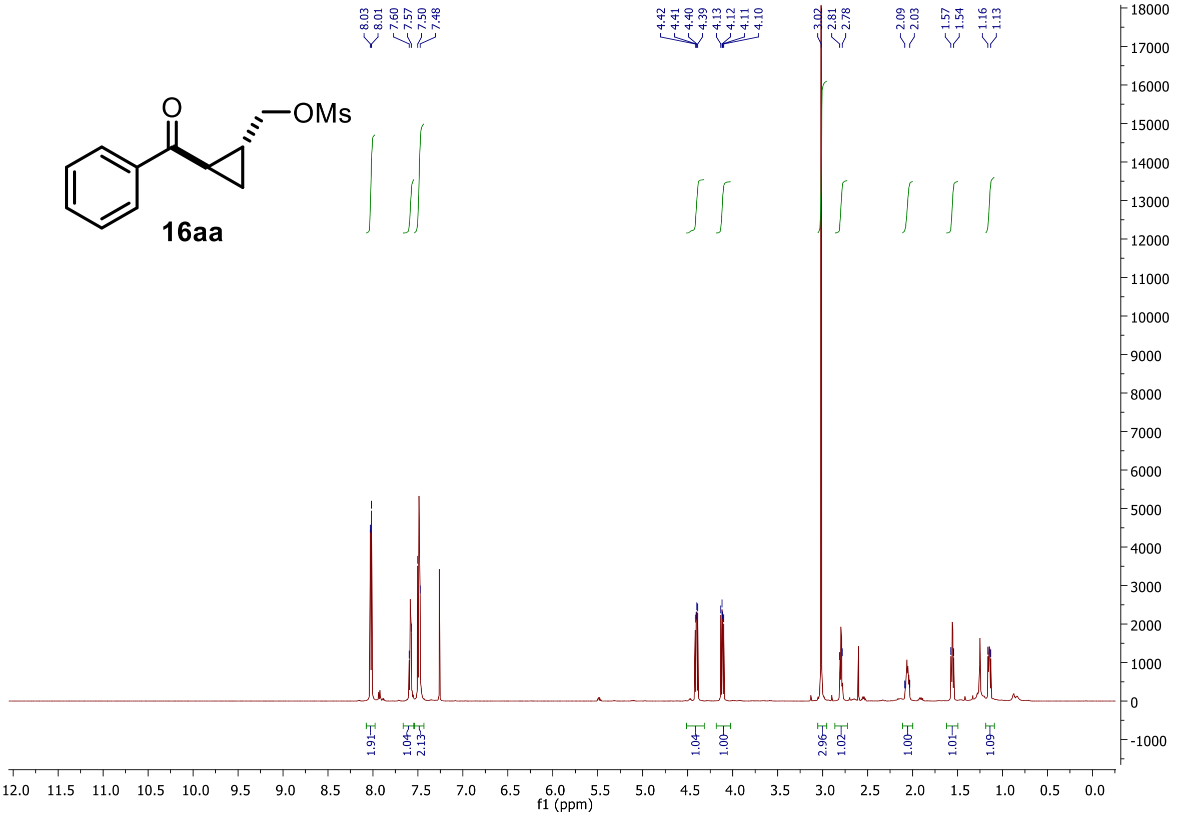
OMs
O
16aa
8.03
8.01
7.60
7.57
7.50
7.48
4.42
4.41
4.40
4.39
4.13
4.12
4.11
4.10
3.02
2.81
2.78
2.09
2.03
1.57
1.54
1.16
1.13
1.91
1.04
2.13
1.04
1.00
2.96
1.02
1.00
1.01
1.09
f1 (ppm)

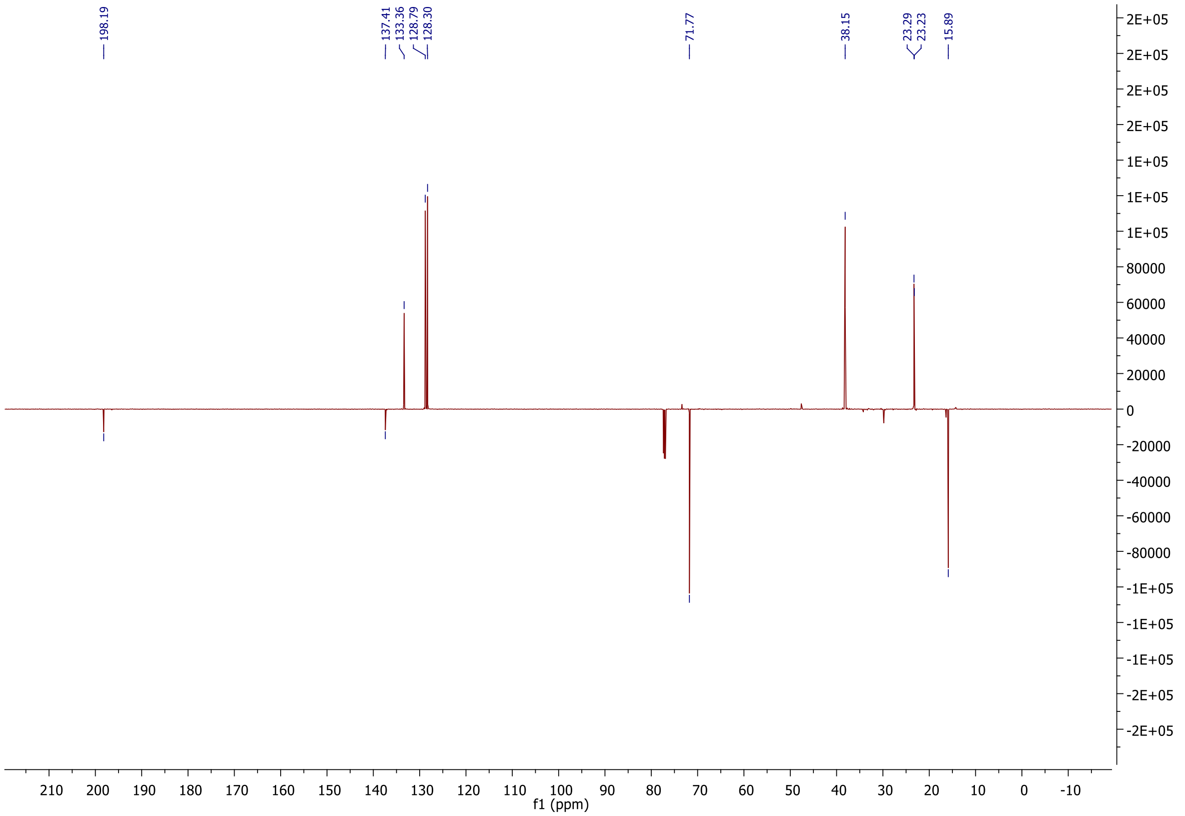
198.19
137.41
133.36
128.79
128.30
71.77
38.15
23.29
23.23
15.89
f1 (ppm)

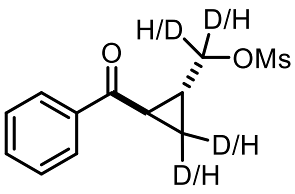
H/D
D/H
OMs
O
D/H
D/H

[D$_2$]-16a/[D$_2$]-16a'

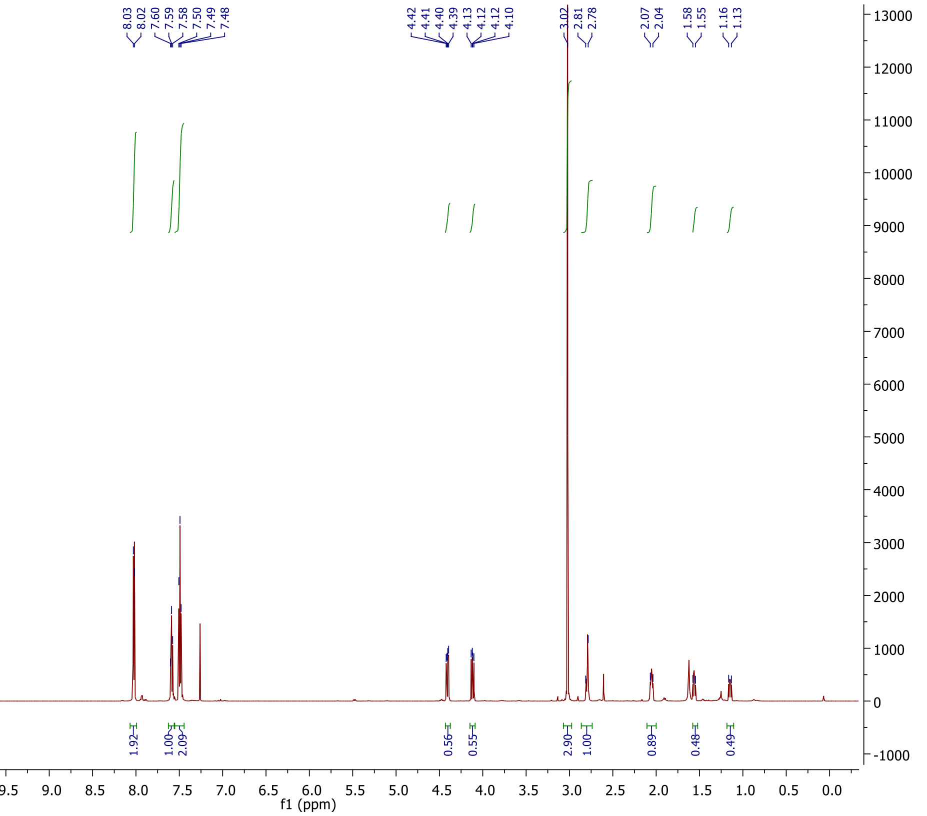
8.03
8.02
7.60
7.59
7.58
7.50
7.49
7.48
4.42
4.41
4.40
4.39
4.13
4.12
4.12
4.10
3.02
2.81
2.78
2.07
2.04
1.58
1.55
1.16
1.13
1.92
1.00
2.09
0.56
0.55
2.90
1.00
0.89
0.48
0.49
f1 (ppm)

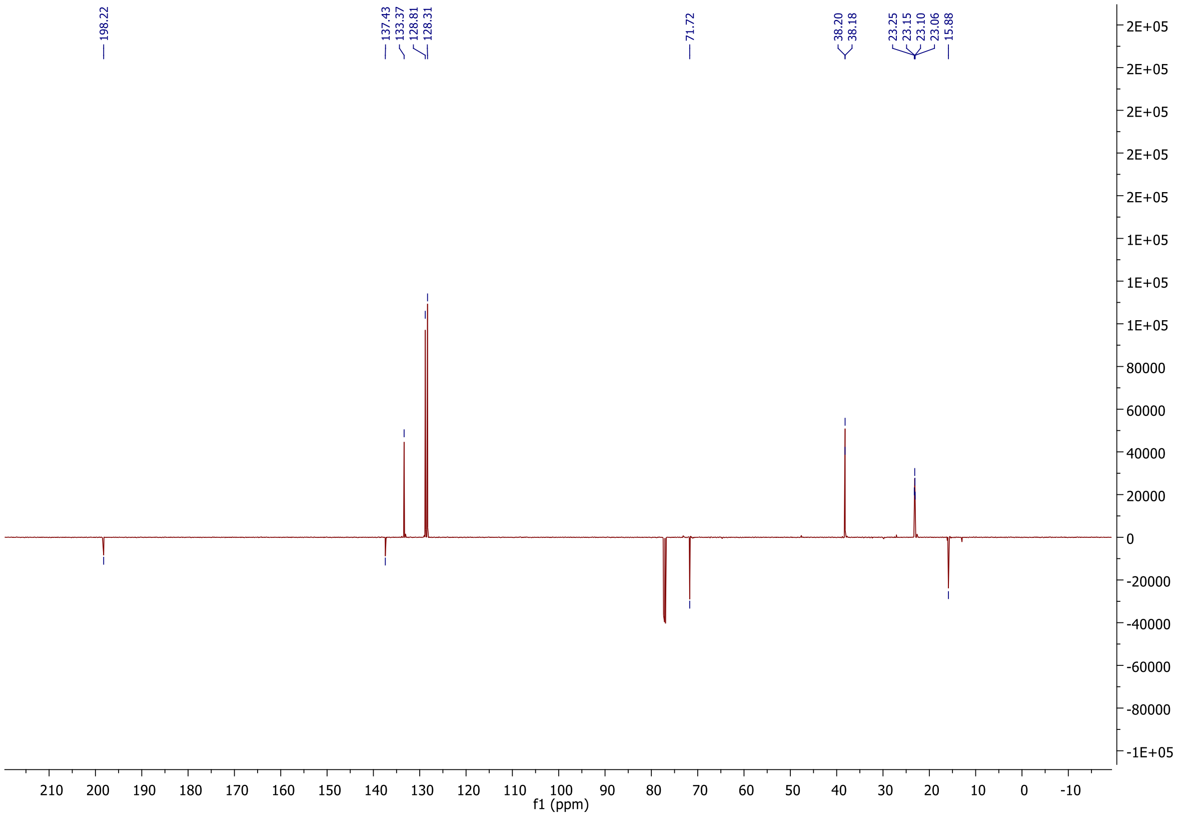
198.22
137.43
133.37
128.81
128.31
71.72
38.20
38.18
23.25
23.15
23.10
23.06
15.88
f1 (ppm)

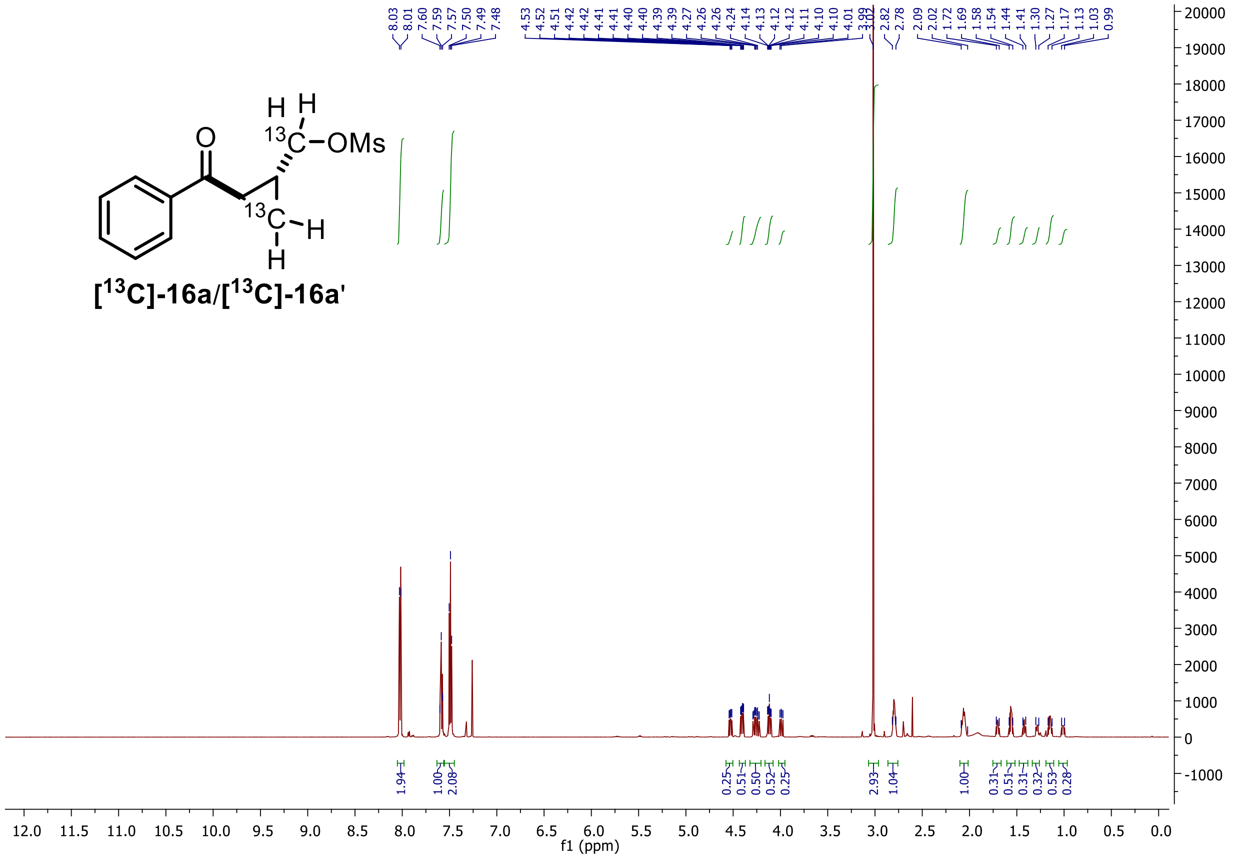
[13C]-16a/[13C]-16a'
f1 (ppm)

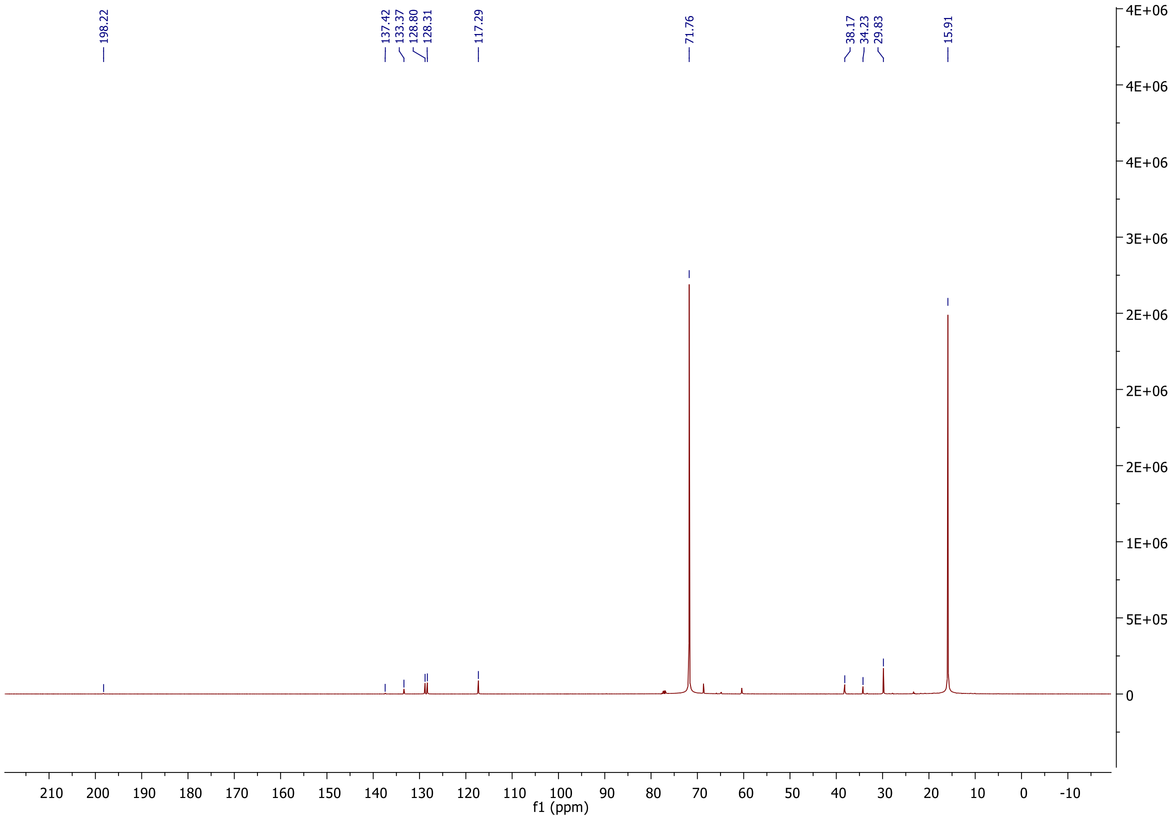
f1 (ppm)

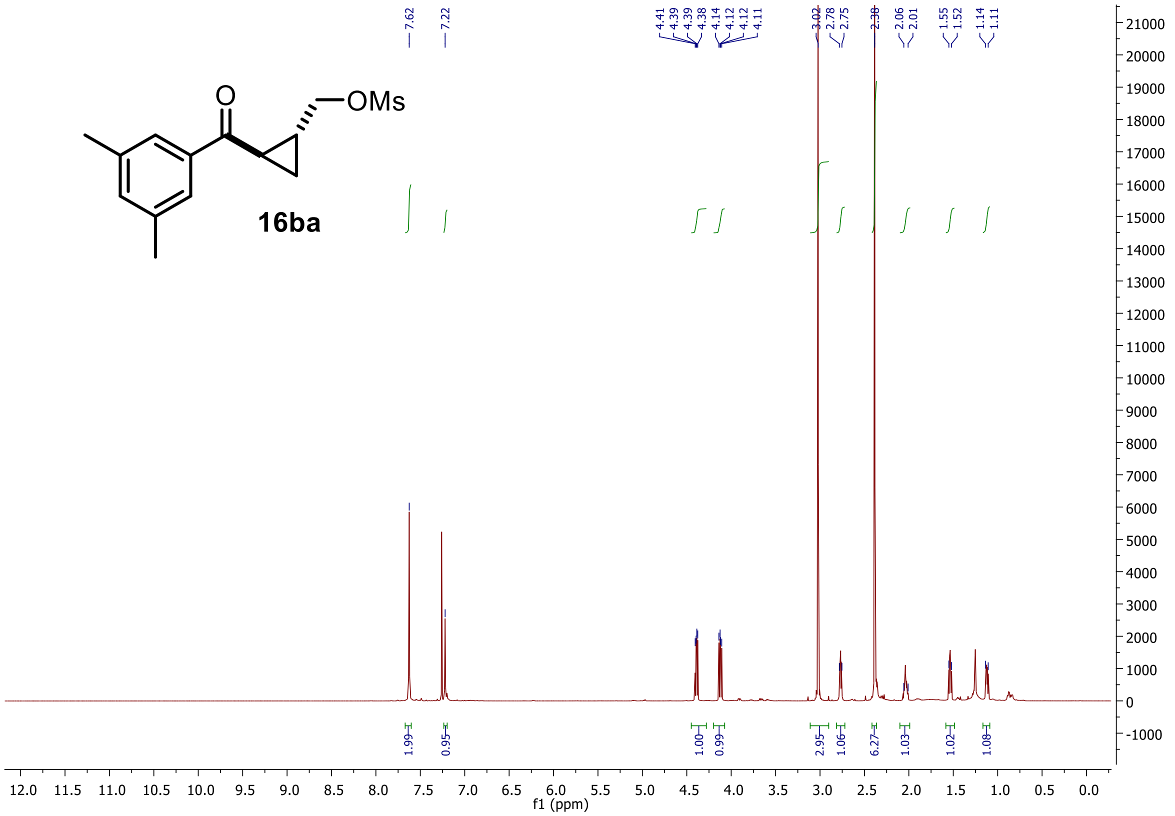
O
OMs
16ba
7.62
7.22
4.41
4.39
4.39
4.38
4.14
4.12
4.12
4.11
3.02
2.78
2.75
2.38
2.06
2.01
1.55
1.52
1.14
1.11
1.99
0.95
1.00
0.99
2.95
1.06
6.27
1.03
1.02
1.08
f1 (ppm)

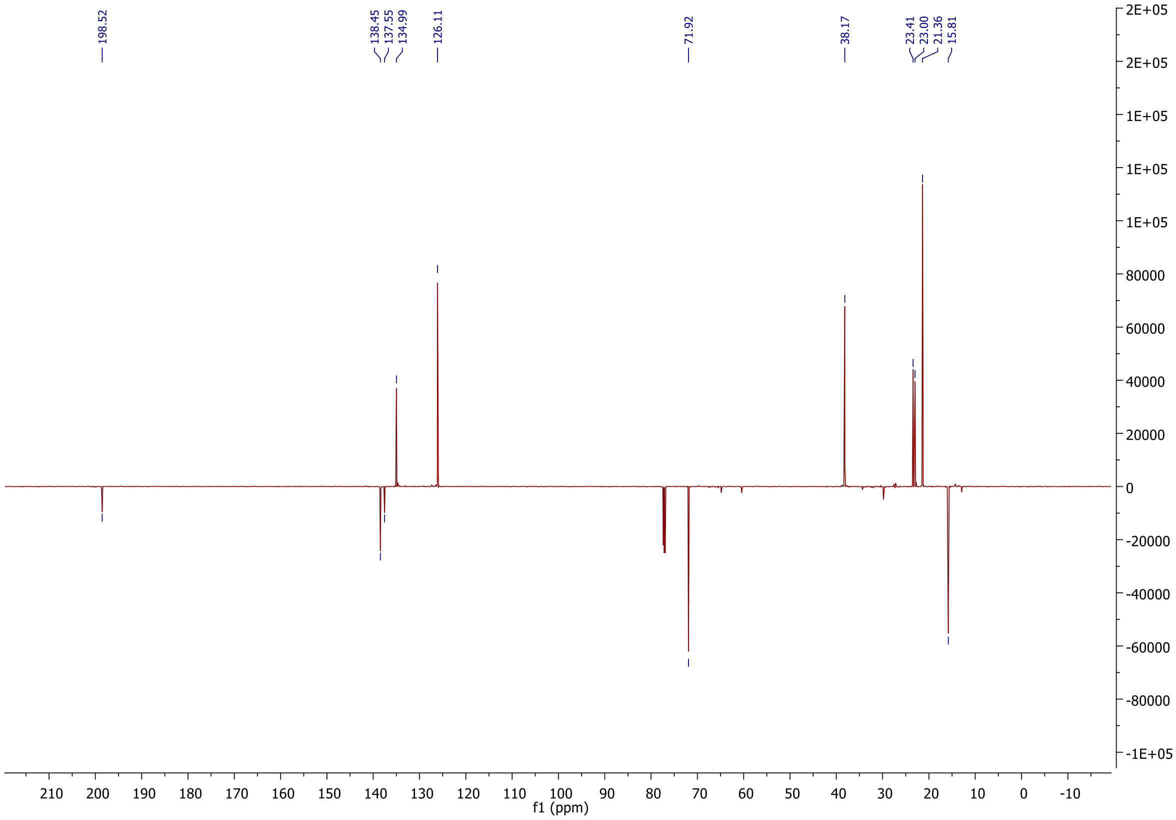
198.52
138.45
137.55
134.99
126.11
71.92
38.17
23.41
23.00
21.36
15.81
f1 (ppm)

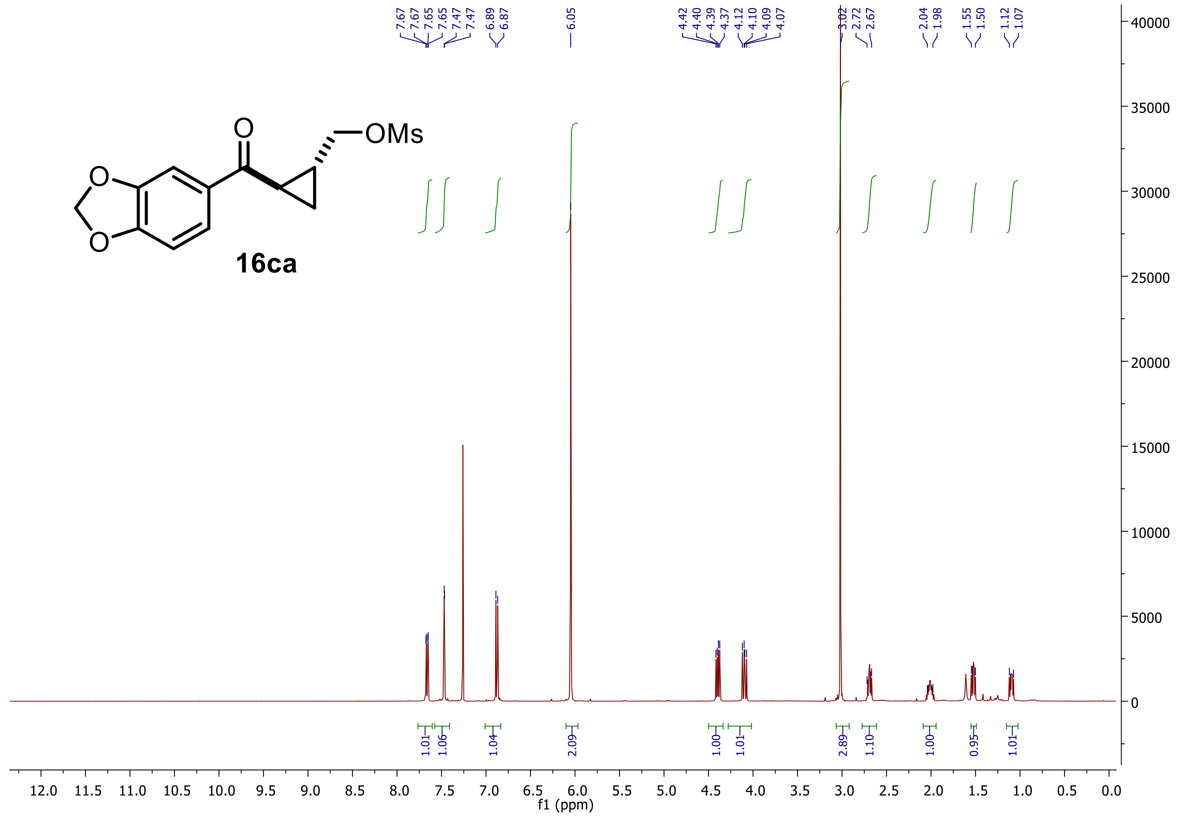
O
O
O
OMs
16ca
f1 (ppm)

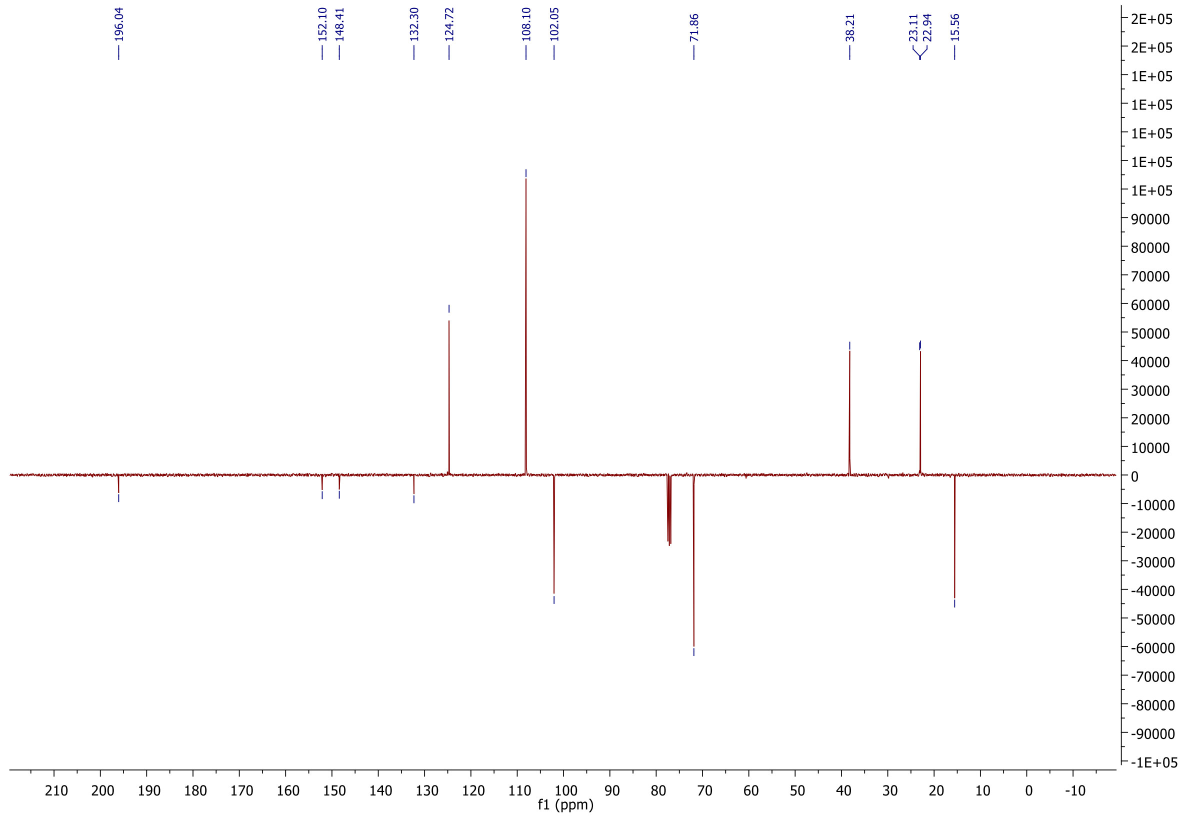
f1 (ppm)

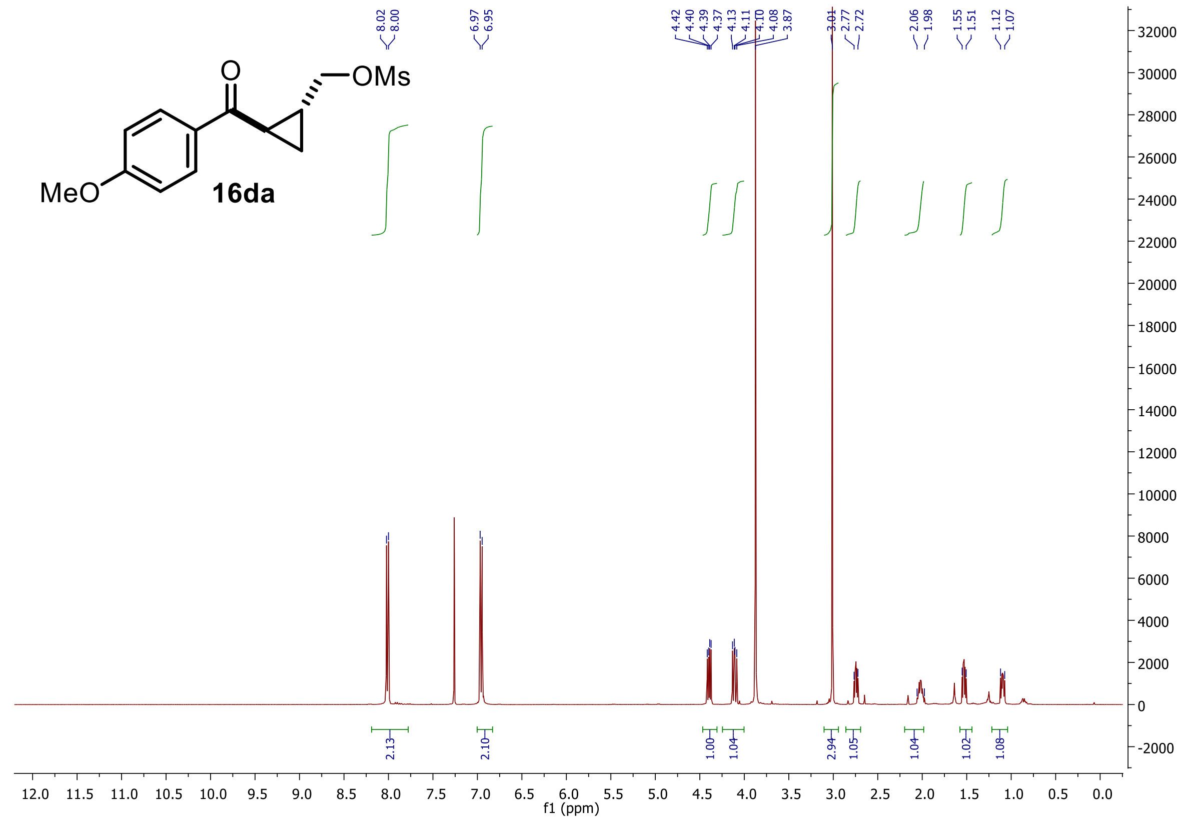
8.02
8.00
6.97
6.95
4.42
4.40
4.39
4.37
4.13
4.11
4.10
4.08
3.87
3.01
2.77
2.72
2.06
1.98
1.55
1.51
1.12
1.07
OMs
MeO
16da
2.13
2.10
1.00
1.04
2.94
1.05
1.04
1.02
1.08
f1 (ppm)

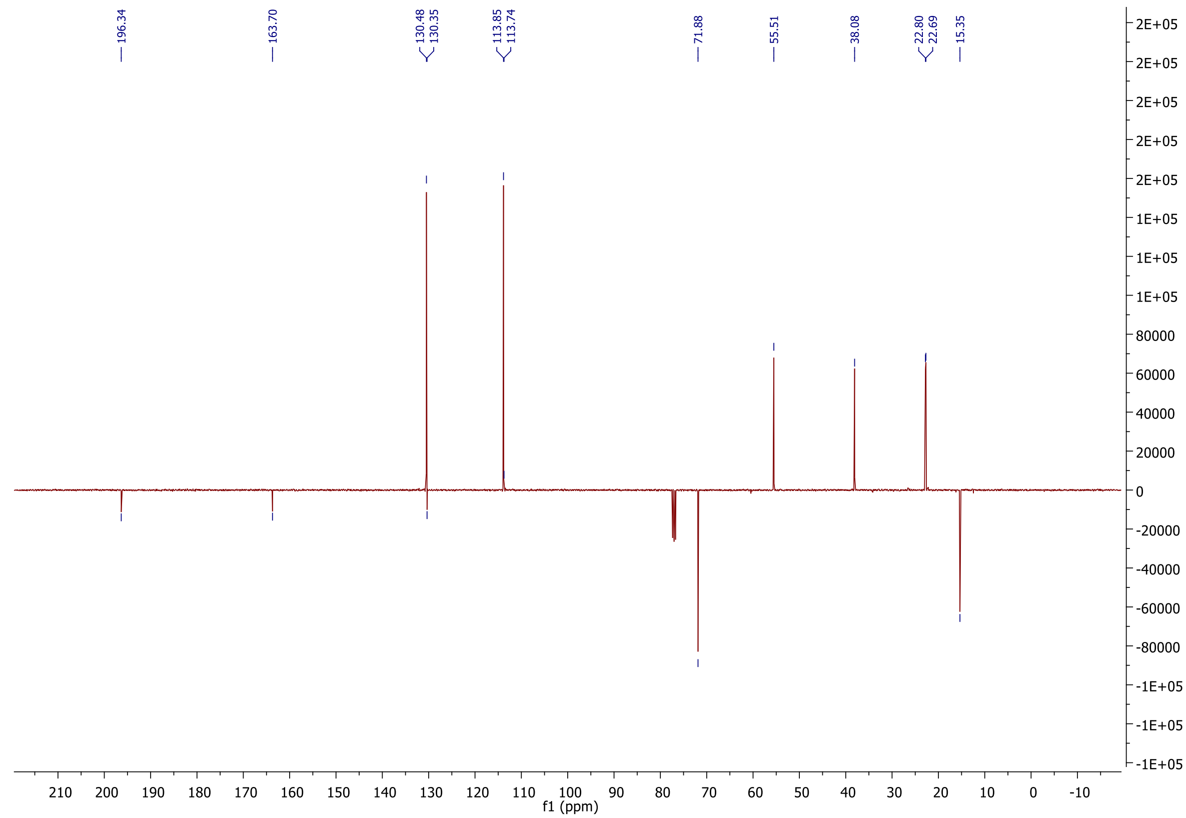
196.34
163.70
130.48
130.35
113.85
113.74
71.88
55.51
38.08
22.80
22.69
15.35
f1 (ppm)

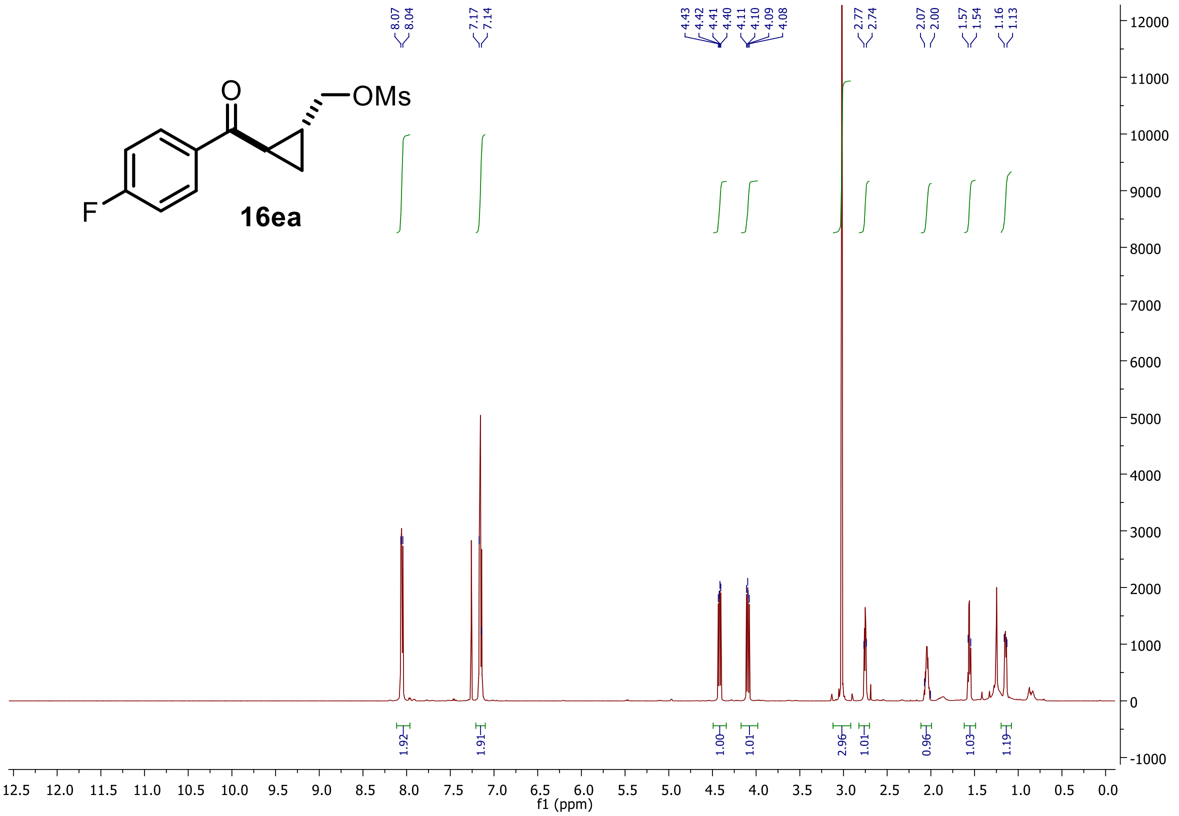
OMs
O
F
16ea
8.07
8.04
7.17
7.14
4.43
4.42
4.41
4.40
4.11
4.10
4.09
4.08
2.77
2.74
2.07
2.00
1.57
1.54
1.16
1.13
1.92
1.91
1.00
1.01
2.96
1.01
0.96
1.03
1.19
f1 (ppm)

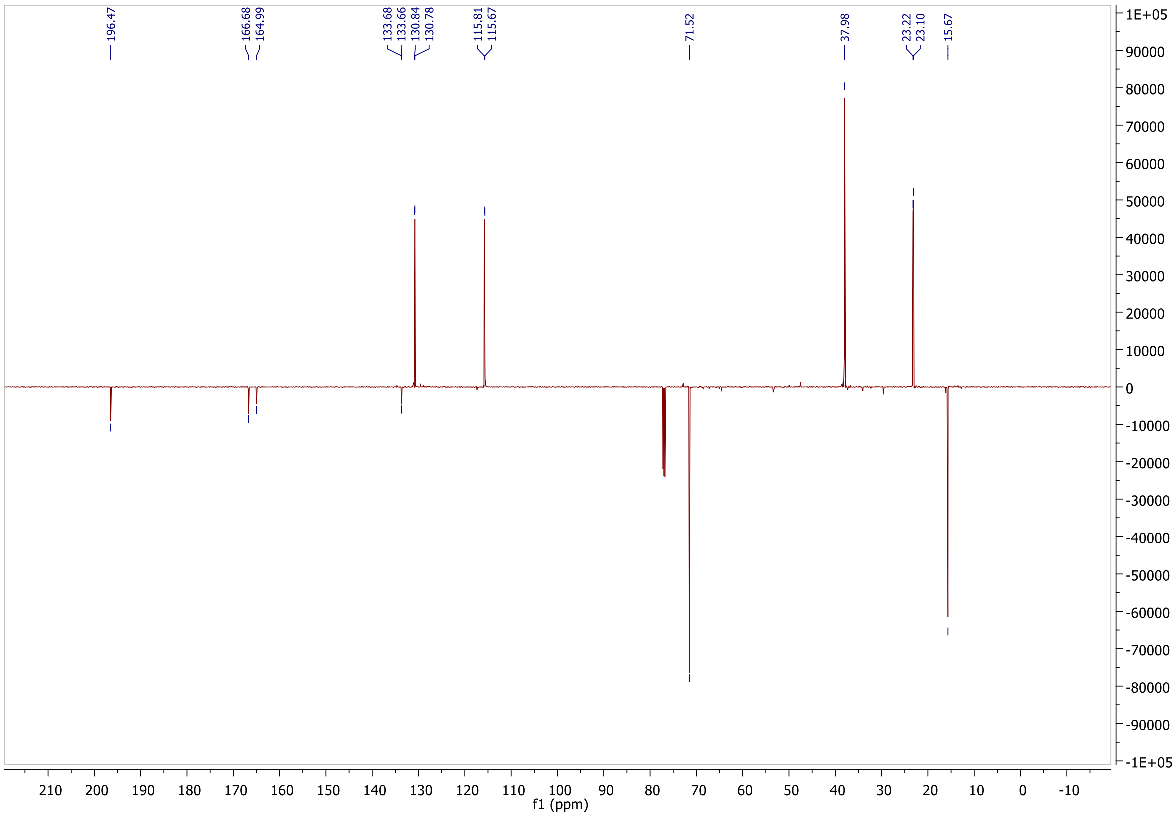
196.47
166.68
164.99
133.68
133.66
130.84
130.78
115.81
115.67
71.52
37.98
23.22
23.10
15.67
f1 (ppm)

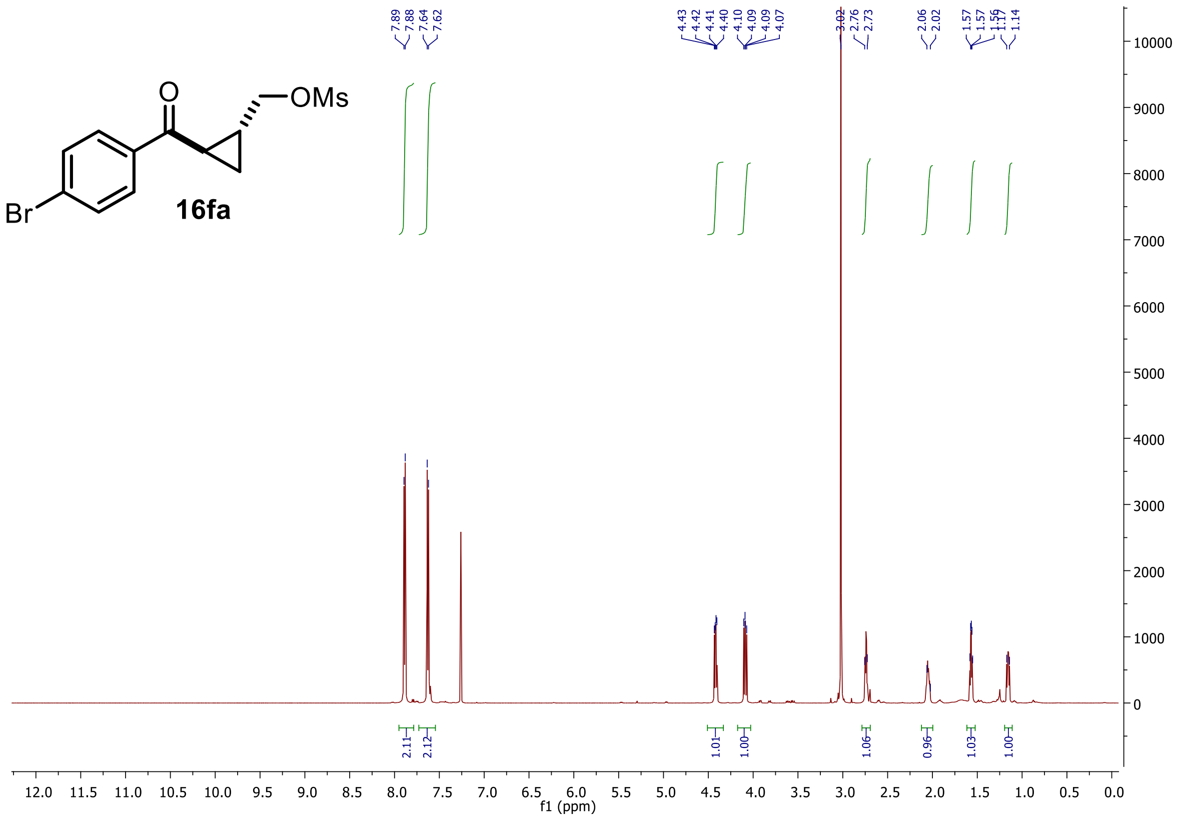
O
OMs
Br
7.89
7.88
7.64
7.62
4.43
4.42
4.41
4.40
4.10
4.09
4.09
4.07
3.02
2.76
2.73
2.06
2.02
1.57
1.57
1.56
1.17
1.14
2.11
2.12
1.01
1.00
1.06
0.96
1.03
1.00
f1 (ppm)

16fa

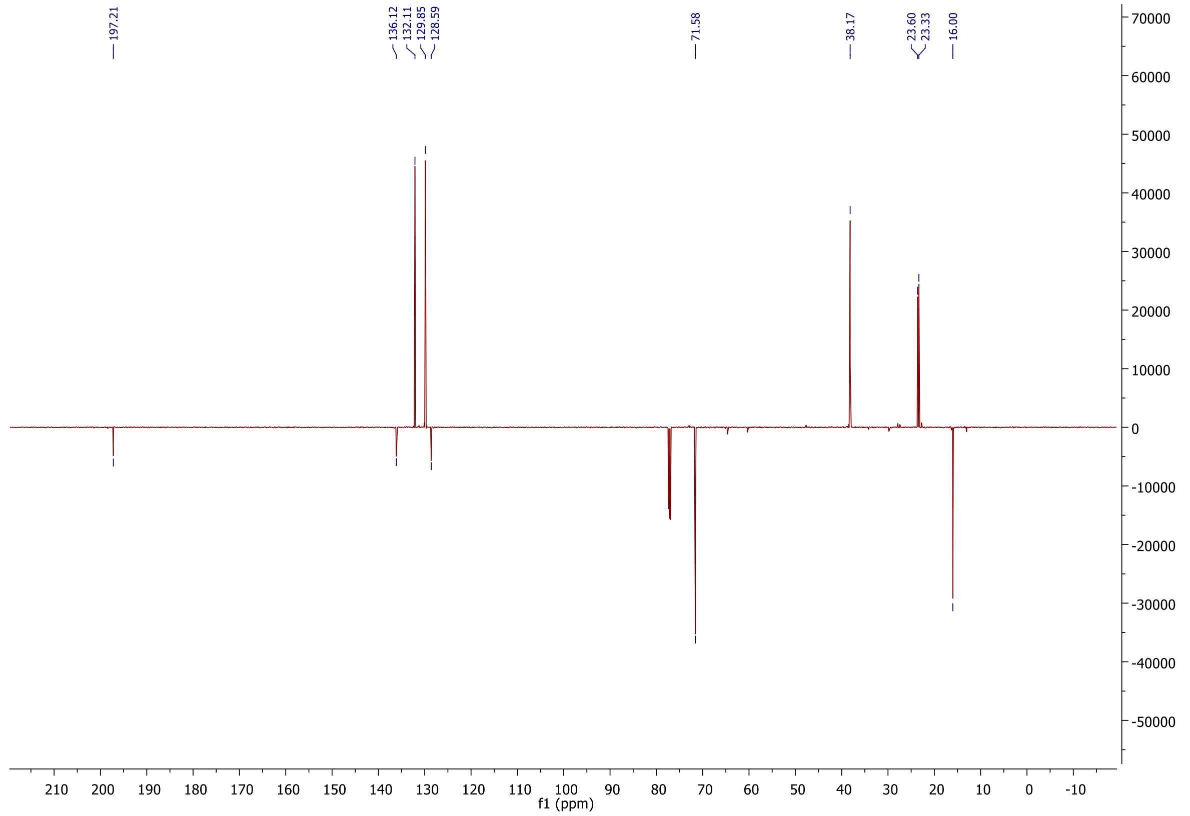
197.21
136.12
132.11
129.85
128.59
71.58
38.17
23.60
23.33
16.00
f1 (ppm)

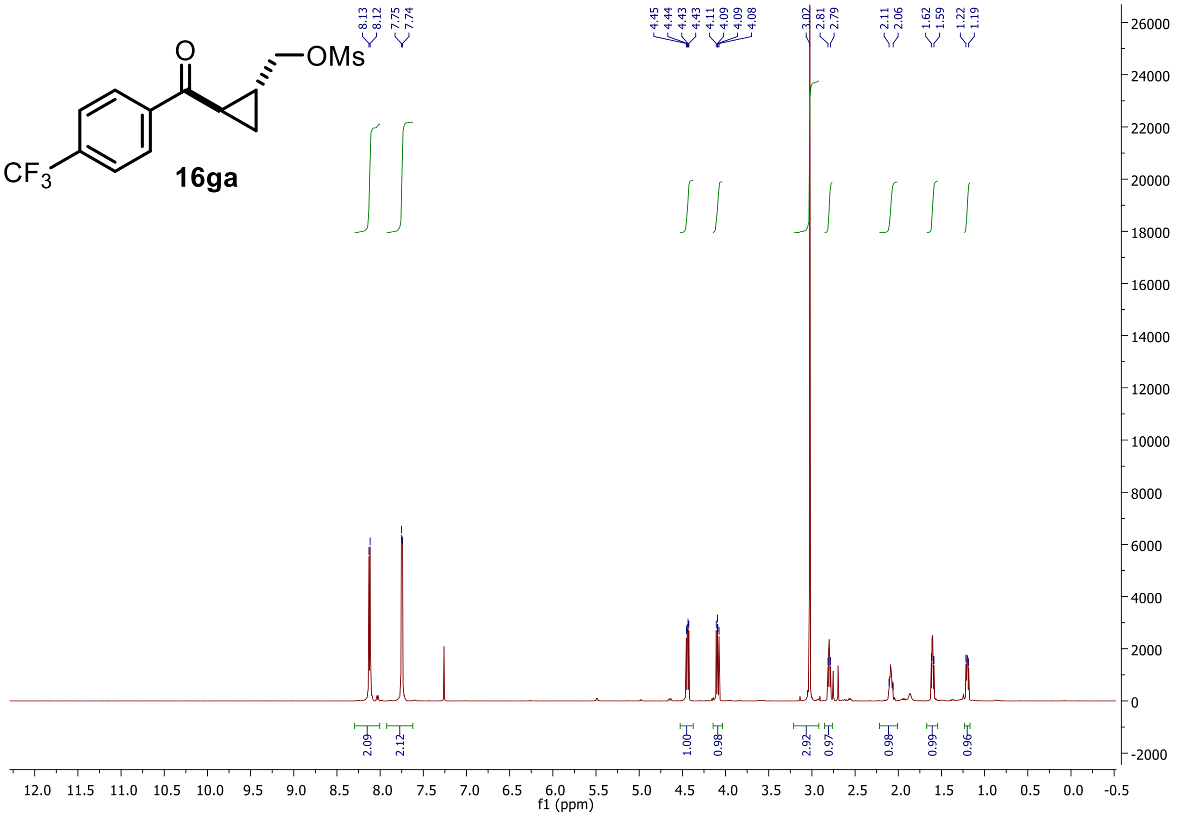
O
OMs
CF3
16ga
8.13
8.12
7.75
7.74
4.45
4.44
4.43
4.43
4.11
4.09
4.09
4.08
3.02
2.81
2.79
2.11
2.06
1.62
1.59
1.22
1.19
2.09
2.12
1.00
0.98
2.92
0.97
0.98
0.99
0.96
f1 (ppm)

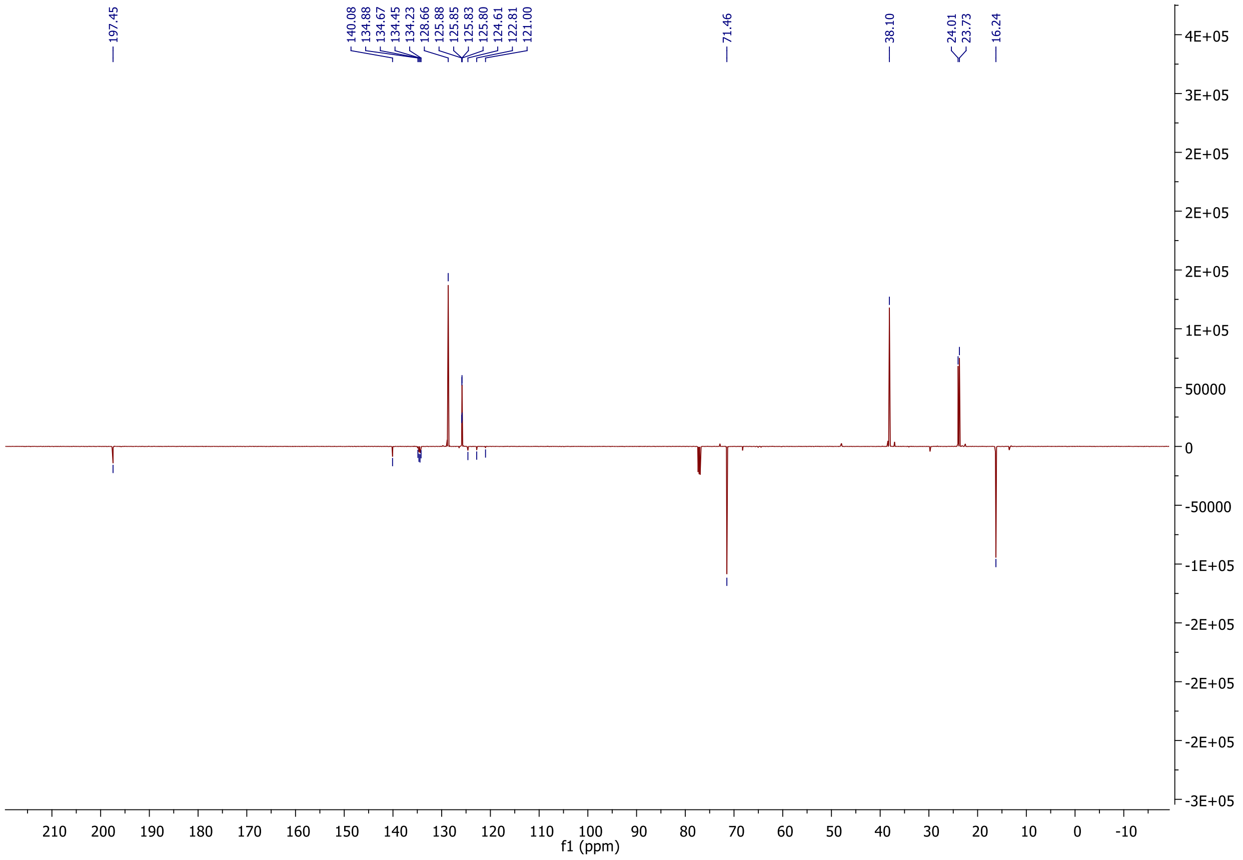
197.45
140.08
134.88
134.67
134.45
134.23
128.66
125.88
125.85
125.83
125.80
124.61
122.81
121.00
71.46
38.10
24.01
23.73
16.24
f1 (ppm)

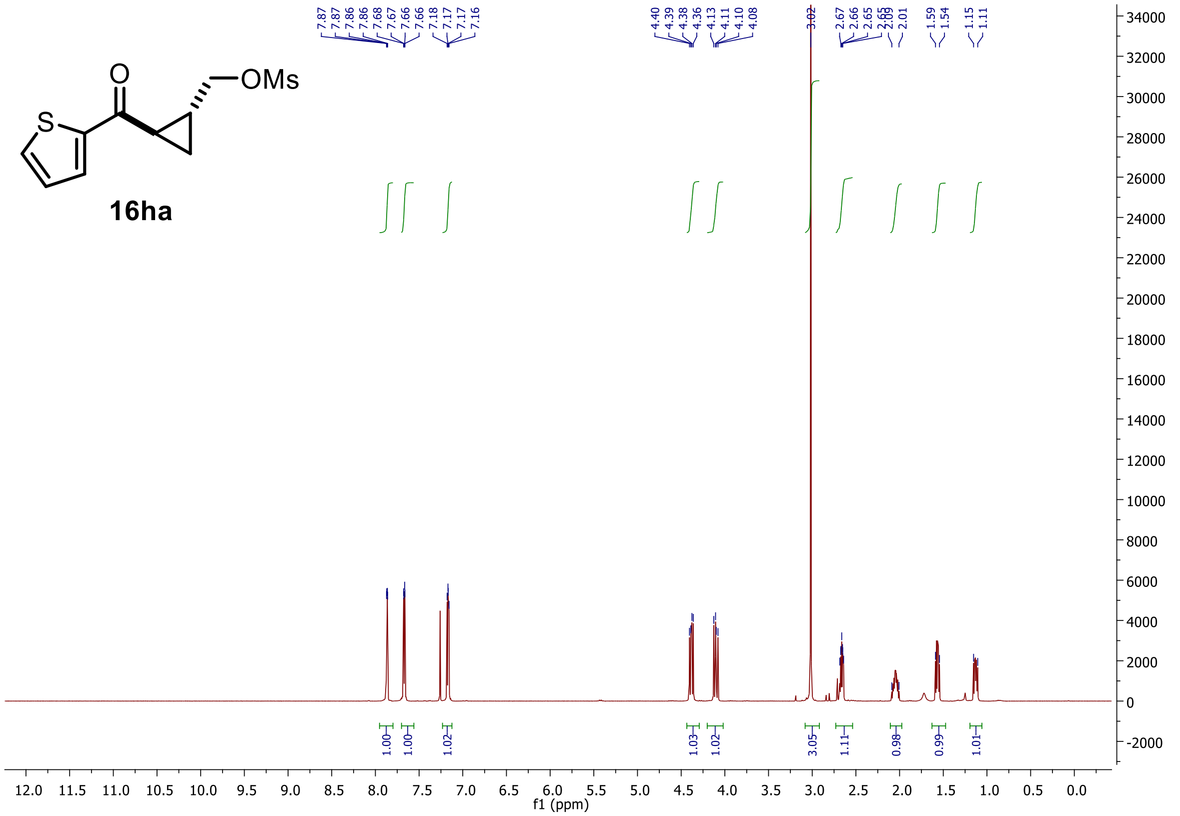

16ha

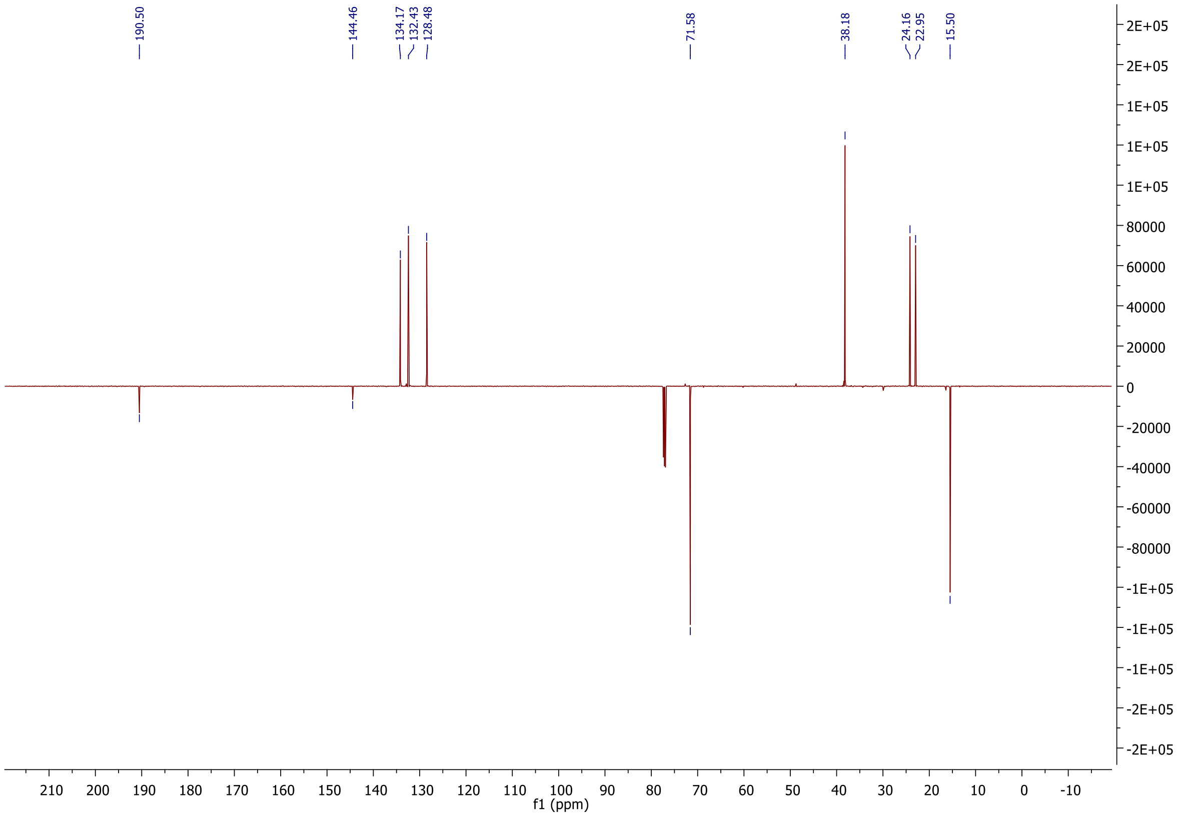

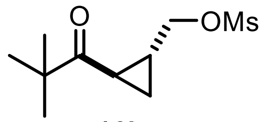
O
OMs

16ia

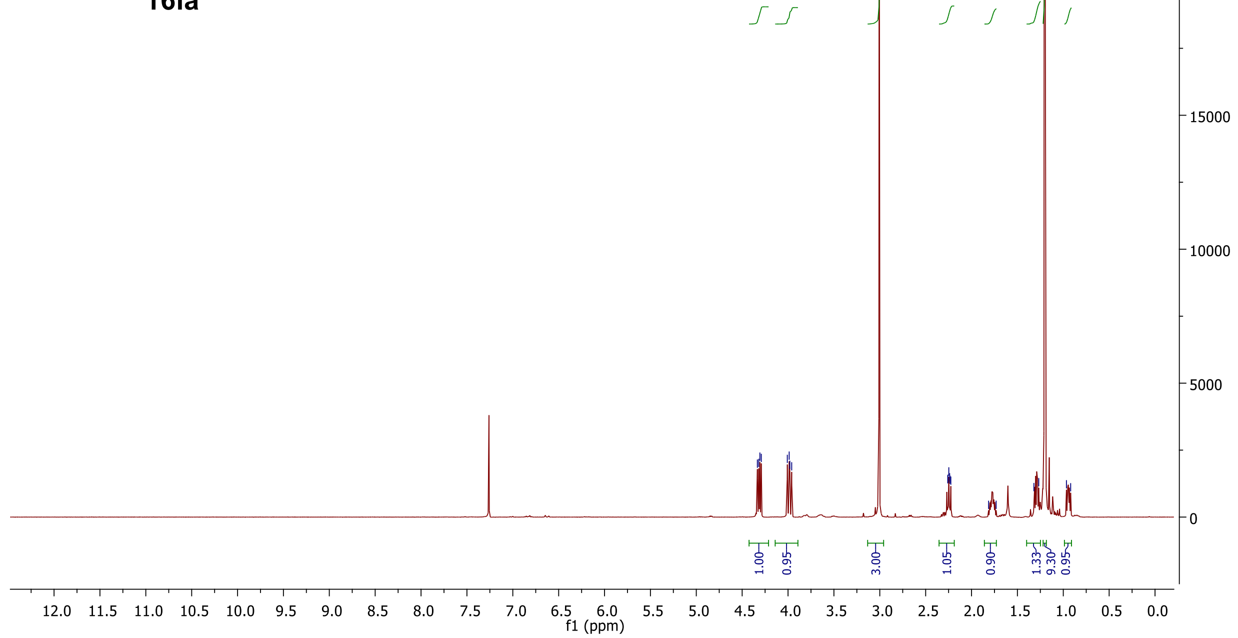
4.33 4.32 4.31 4.29 4.01 3.99 3.98 3.96 3.01 2.26 2.26 2.25 2.24 2.24 2.23 1.81 1.73 1.32 1.27 1.20 0.97 0.92
1.00 0.95 3.00 1.05 0.90 1.33 9.30 0.95
f1 (ppm)

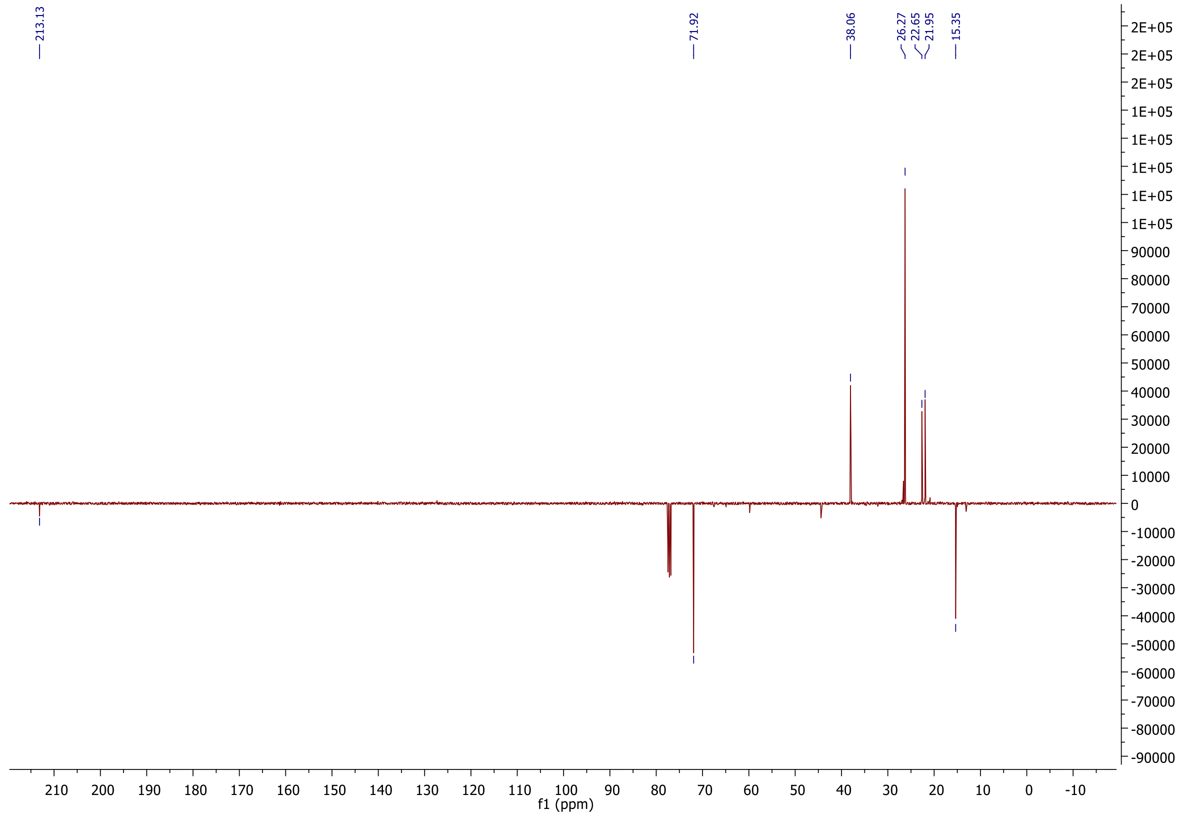
213.13
71.92
38.06
26.27 22.65 21.95
15.35
f1 (ppm)

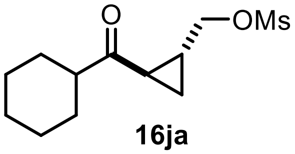
O
OMs

16ja

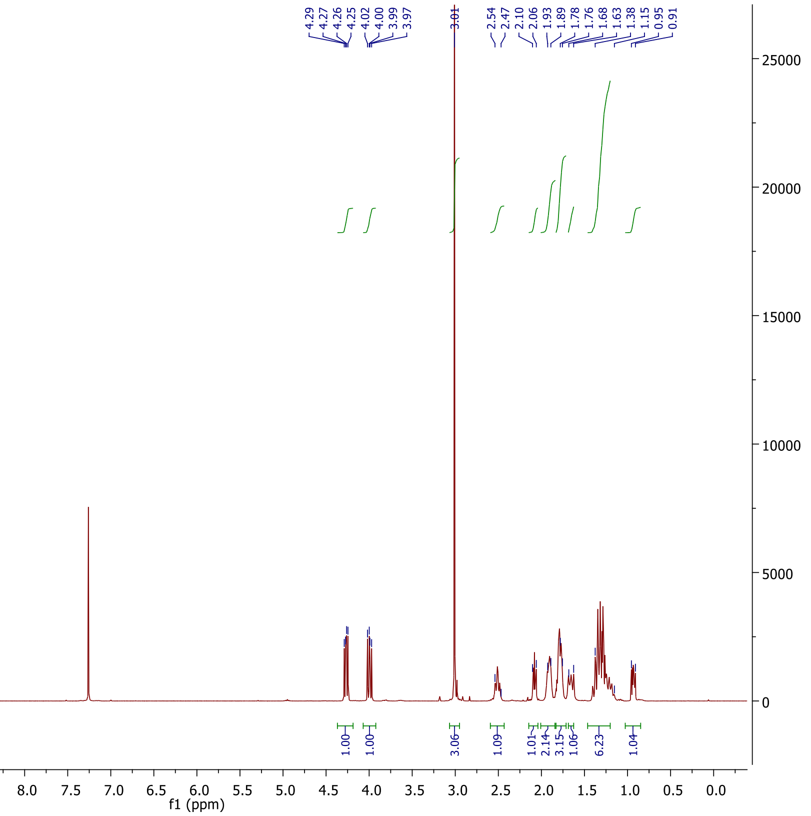
4.29
4.27
4.26
4.25
4.02
4.00
3.99
3.97
3.01
2.54
2.47
2.10
2.06
1.93
1.89
1.78
1.76
1.68
1.63
1.38
1.15
0.95
0.91
1.00
1.00
3.06
1.09
1.01
2.14
3.15
1.06
6.23
1.04
f1 (ppm)

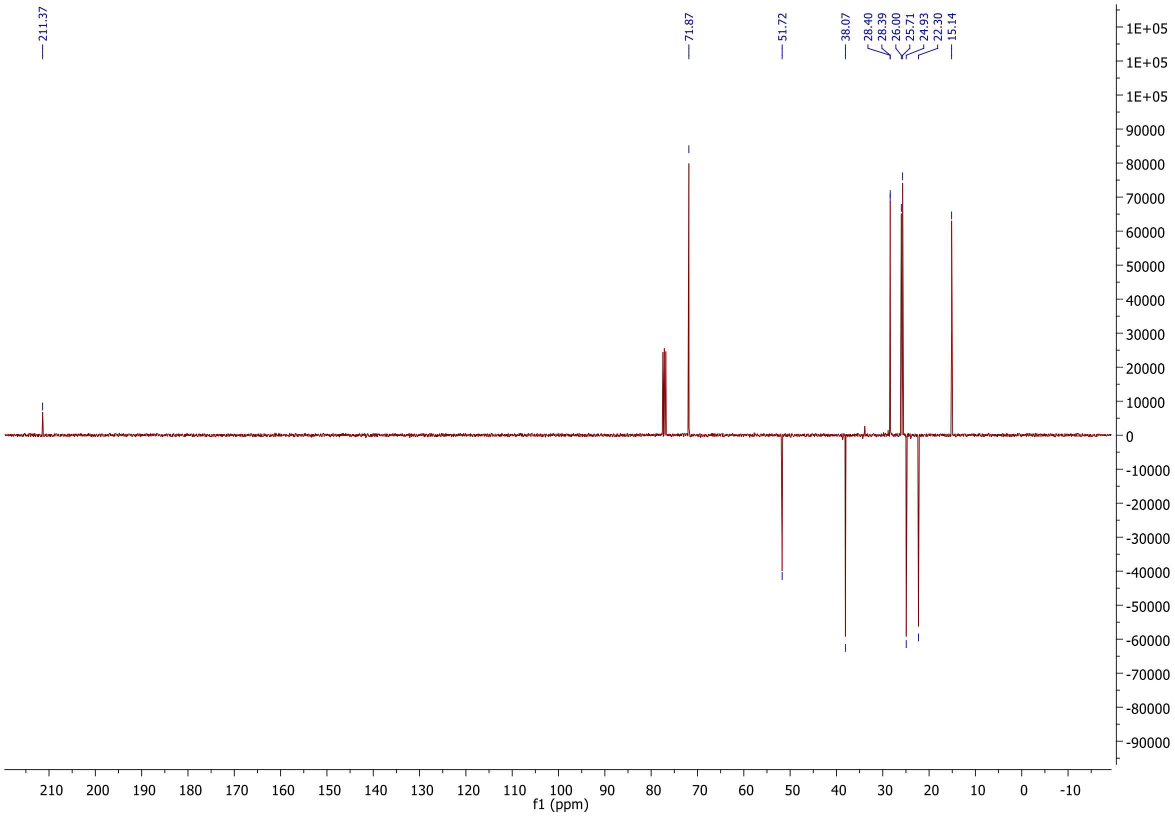
211.37
71.87
51.72
38.07
28.40
28.39
26.00
25.71
24.93
22.30
15.14
f1 (ppm)

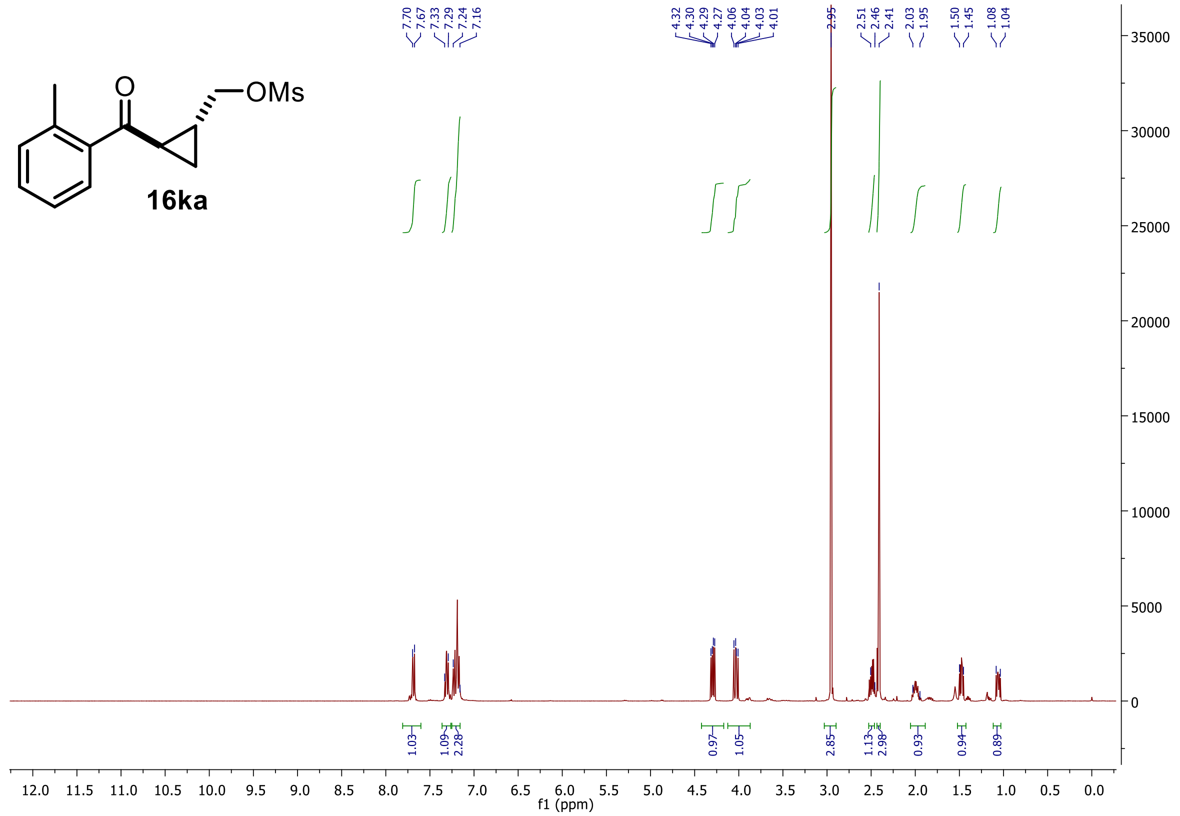
O
OMs
16ka
7.70
7.67
7.33
7.29
7.24
7.16
4.32
4.30
4.29
4.27
4.06
4.04
4.03
4.01
2.95
2.51
2.46
2.41
2.03
1.95
1.50
1.45
1.08
1.04
1.03
1.09
2.28
0.97
1.05
2.85
1.13
2.98
0.93
0.94
0.89
f1 (ppm)

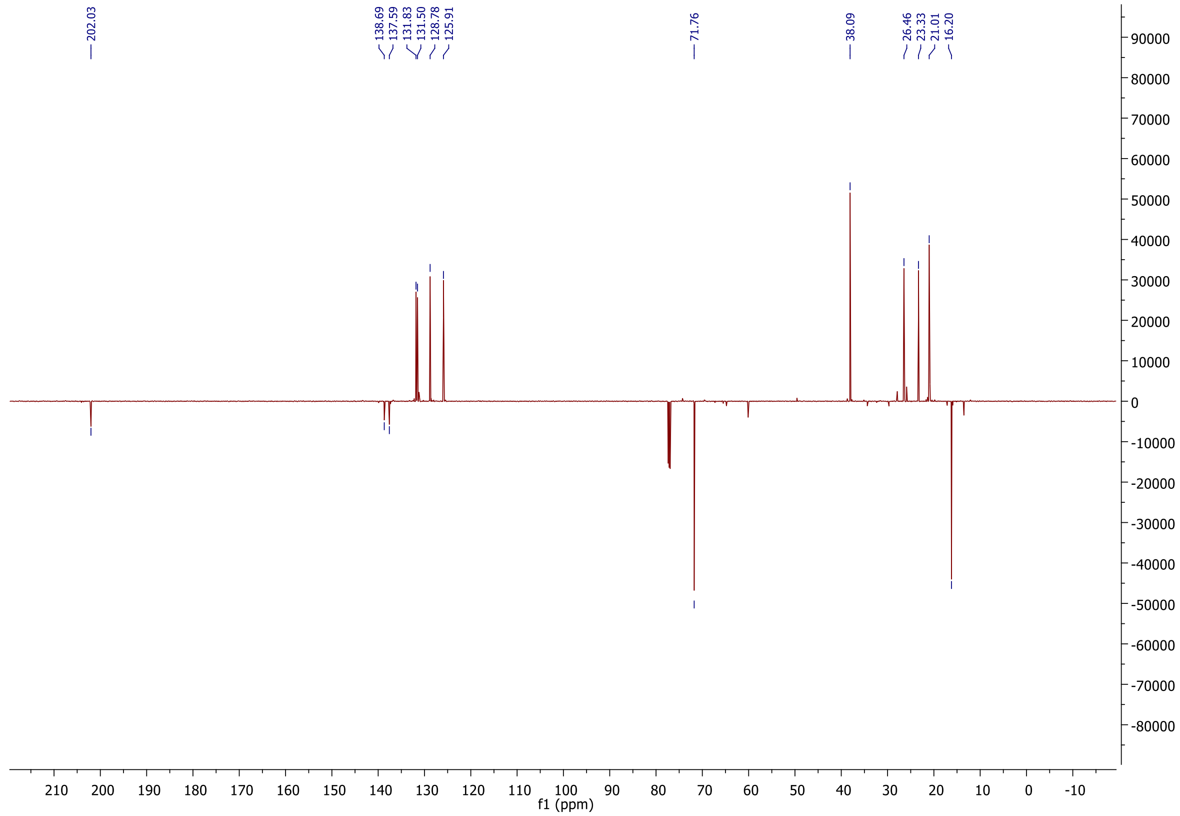
202.03
138.69
137.59
131.83
131.50
128.78
125.91
71.76
38.09
26.46
23.33
21.01
16.20
f1 (ppm)

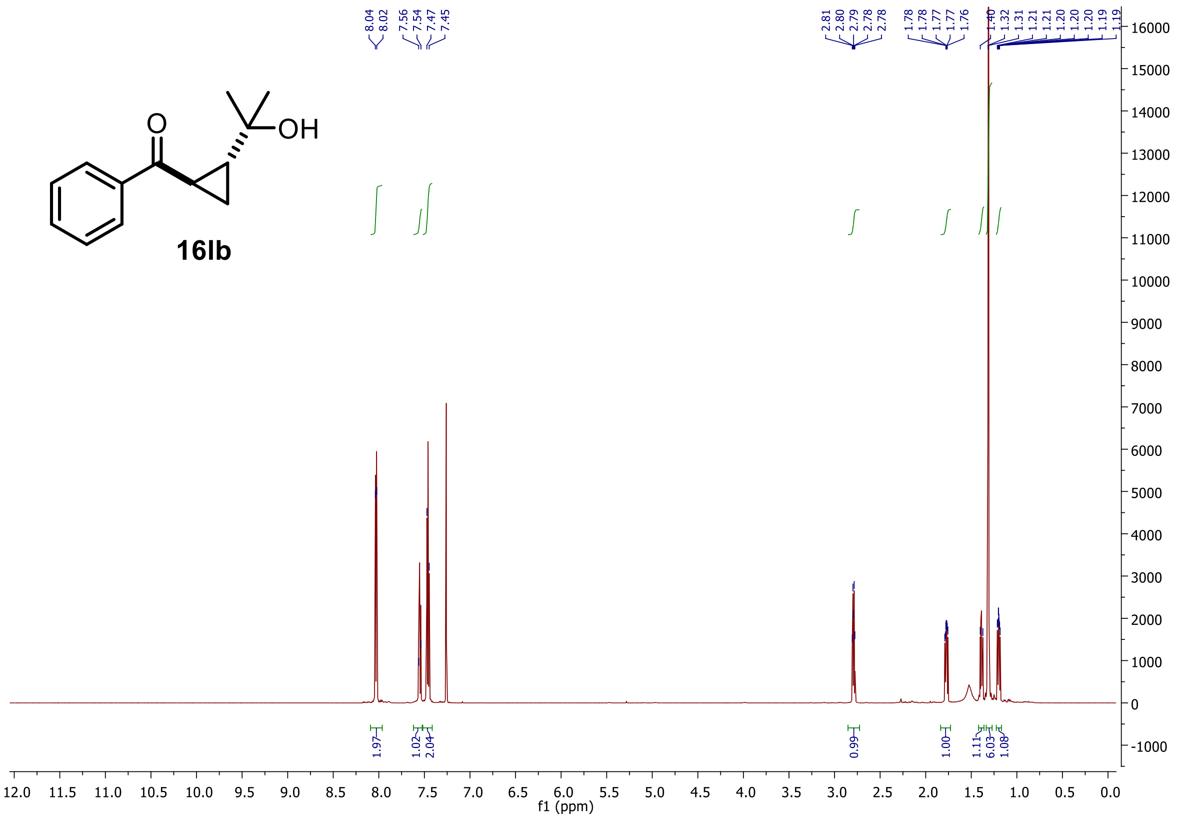

16lb

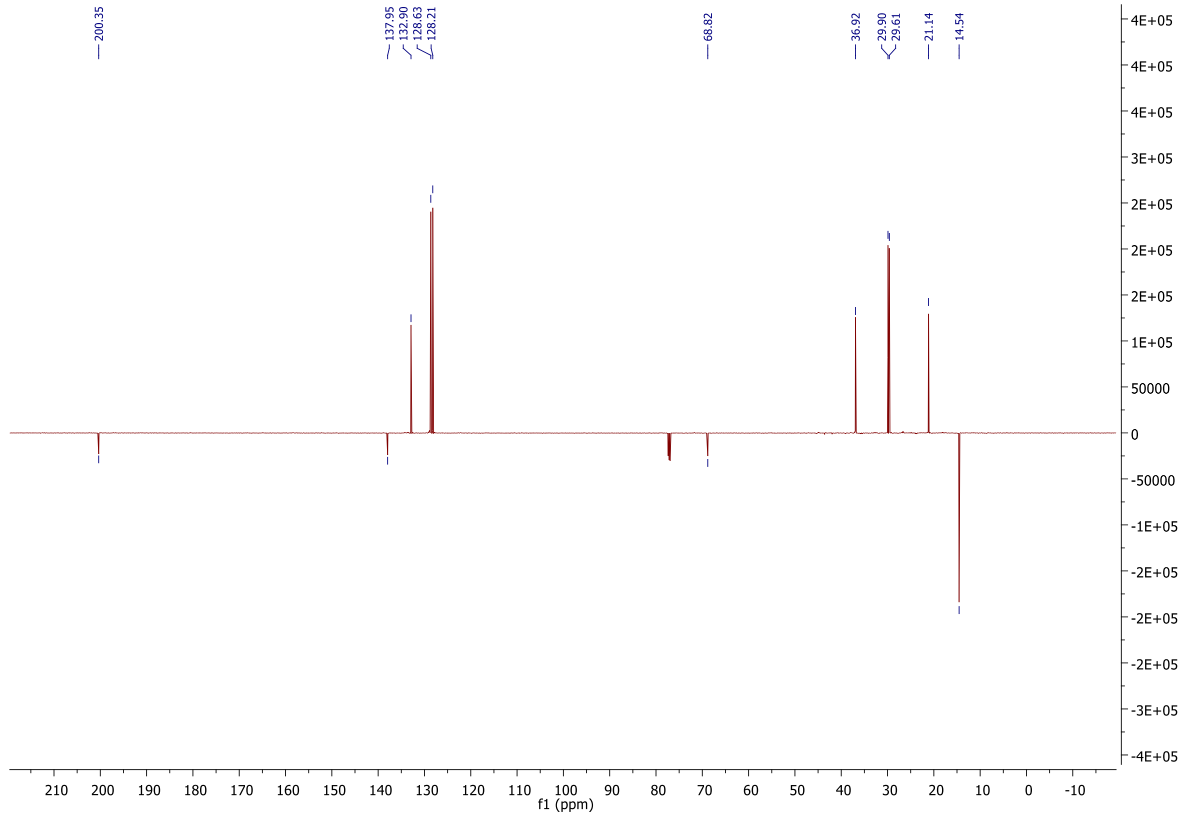

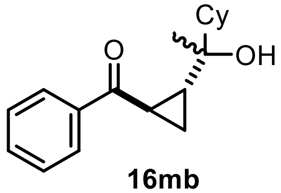
Cy
O
OH

16mb

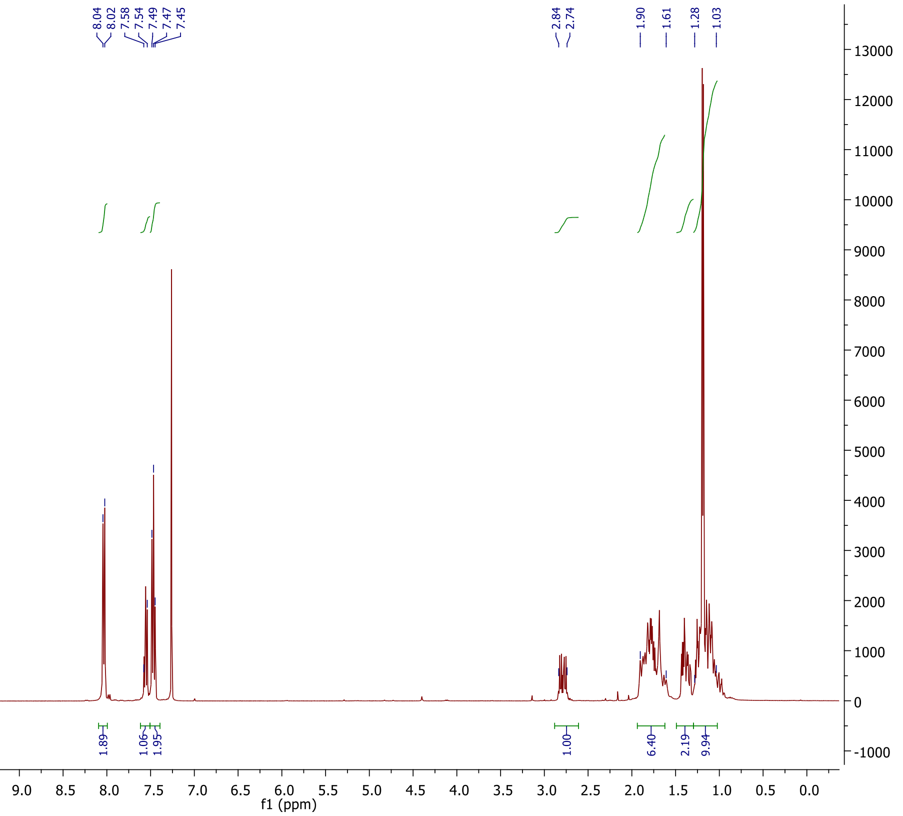
8.04
8.02
7.58
7.54
7.49
7.47
7.45
2.84
2.74
1.90
1.61
1.28
1.03
1.89
1.06
1.95
1.00
6.40
2.19
9.94
f1 (ppm)

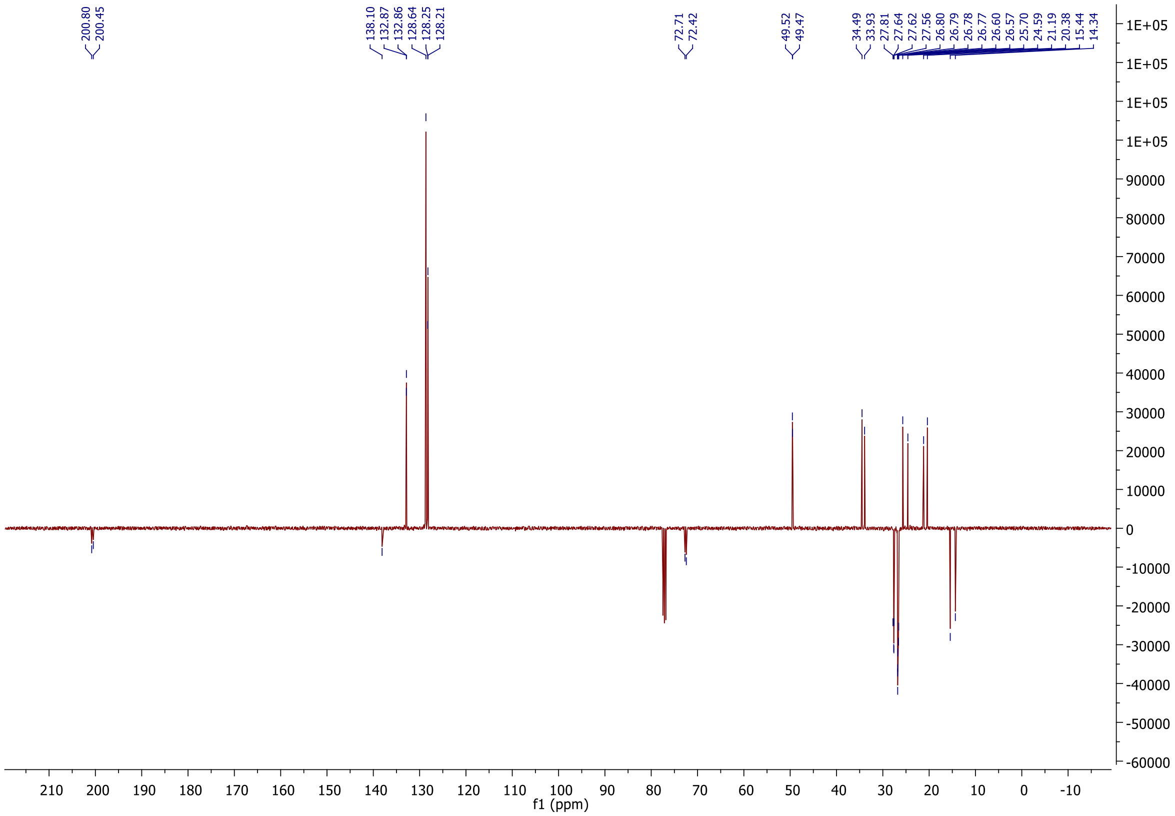
200.80
200.45
138.10
132.87
132.86
128.64
128.25
128.21
72.71
72.42
49.52
49.47
34.49
33.93
27.81
27.64
27.62
27.56
26.80
26.79
26.78
26.77
26.60
26.57
25.70
24.59
21.19
20.38
15.44
14.34
f1 (ppm)

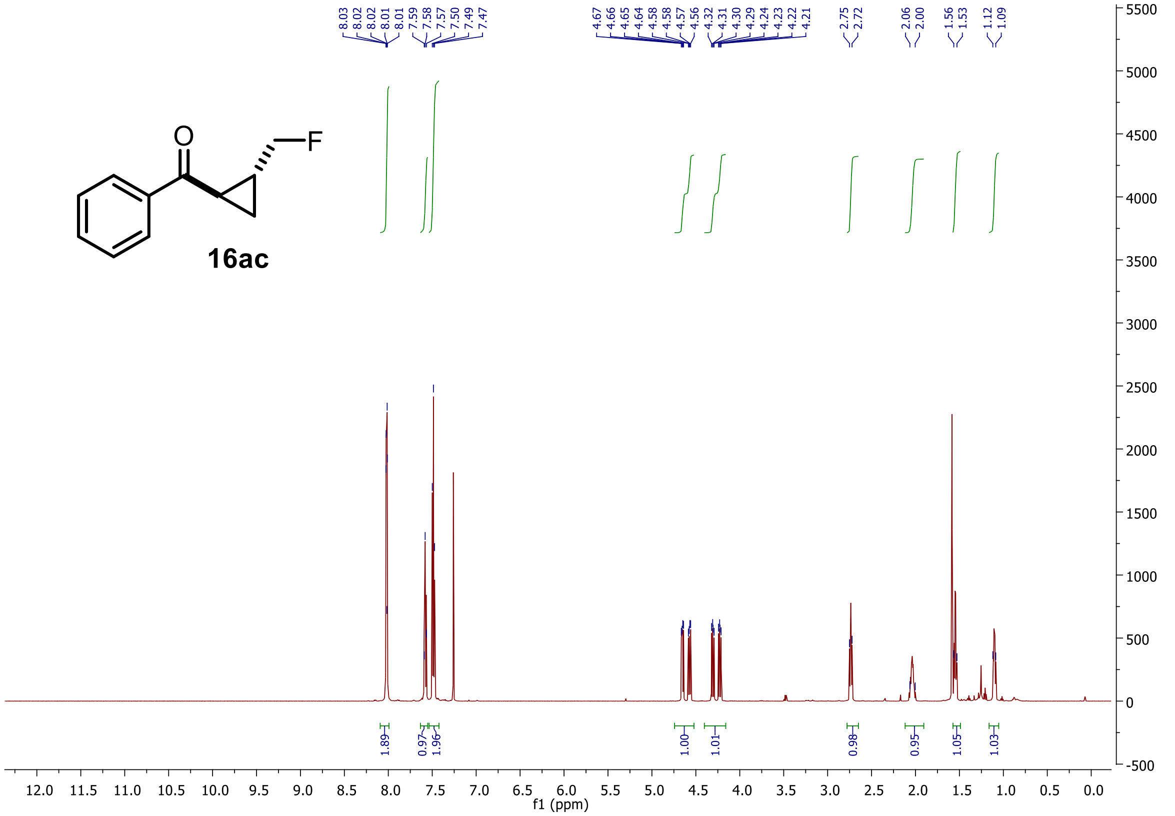

16ac

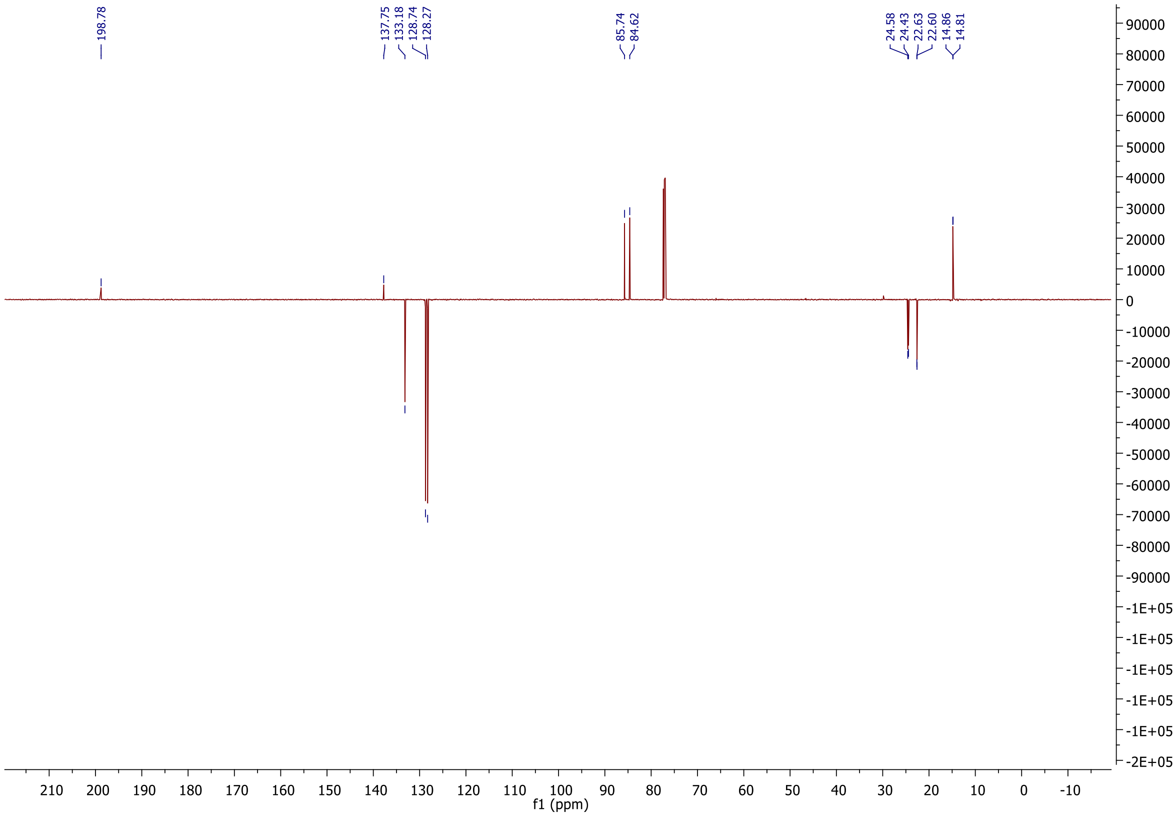

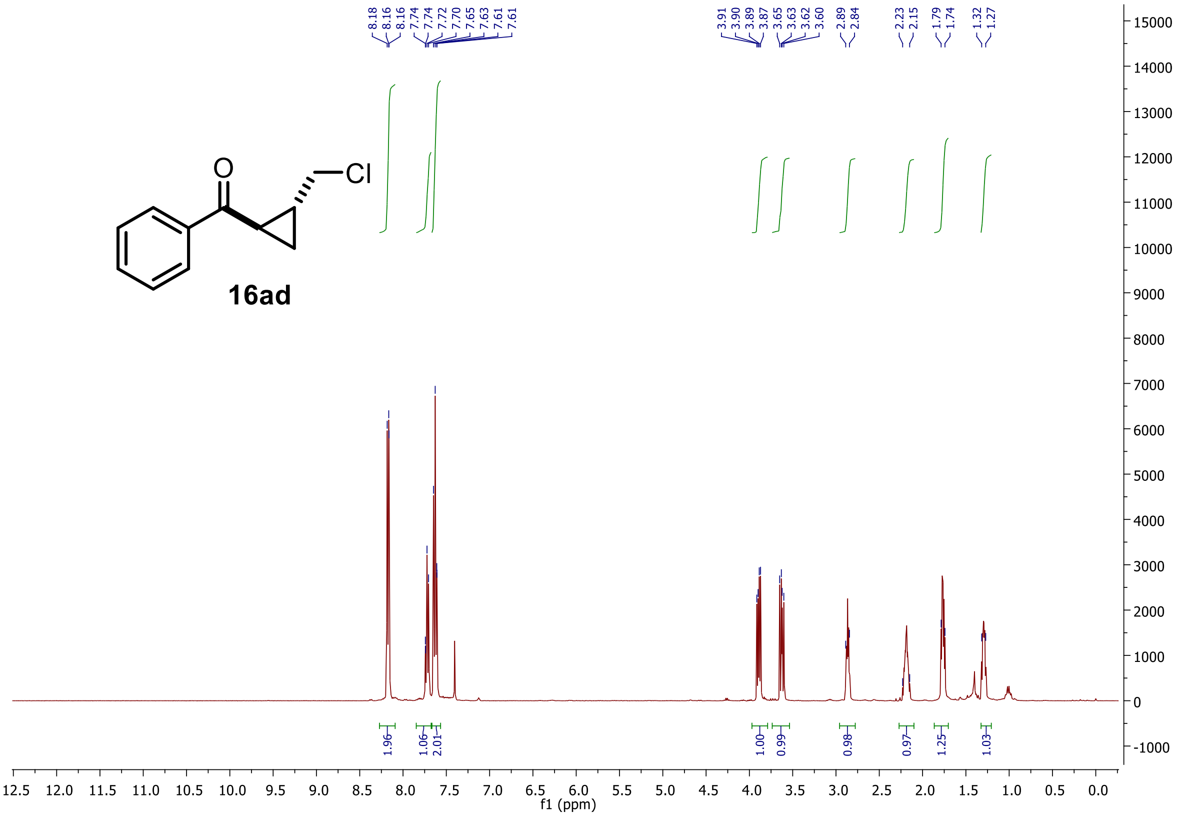
8.18 8.16 8.16 7.74 7.74 7.72 7.70 7.65 7.63 7.61 7.61
3.91 3.90 3.89 3.87 3.65 3.63 3.62 3.60 2.89 2.84 2.23 2.15 1.79 1.74 1.32 1.27
O
Cl
16ad
1.96 1.06 2.01 1.00 0.99 0.98 0.97 1.25 1.03
f1 (ppm)

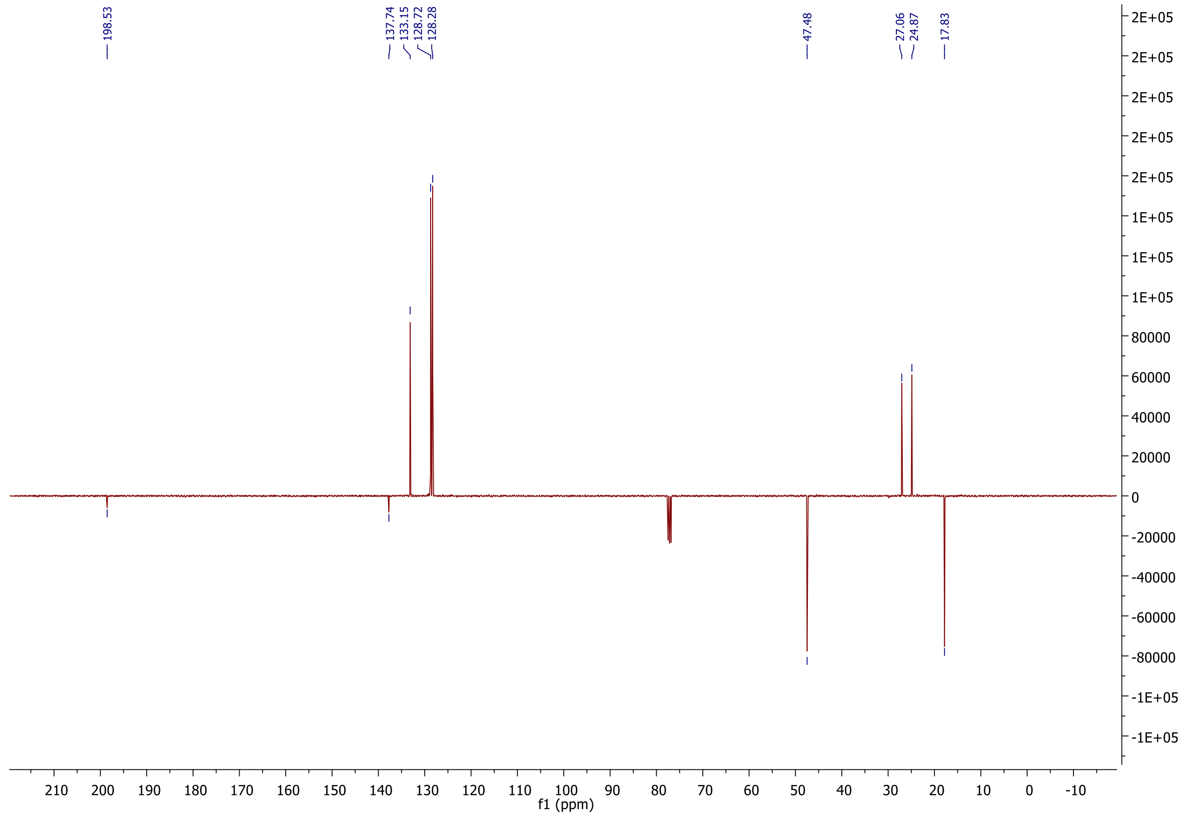
198.53
137.74 133.15 128.72 128.28
47.48
27.06 24.87 17.83
f1 (ppm)

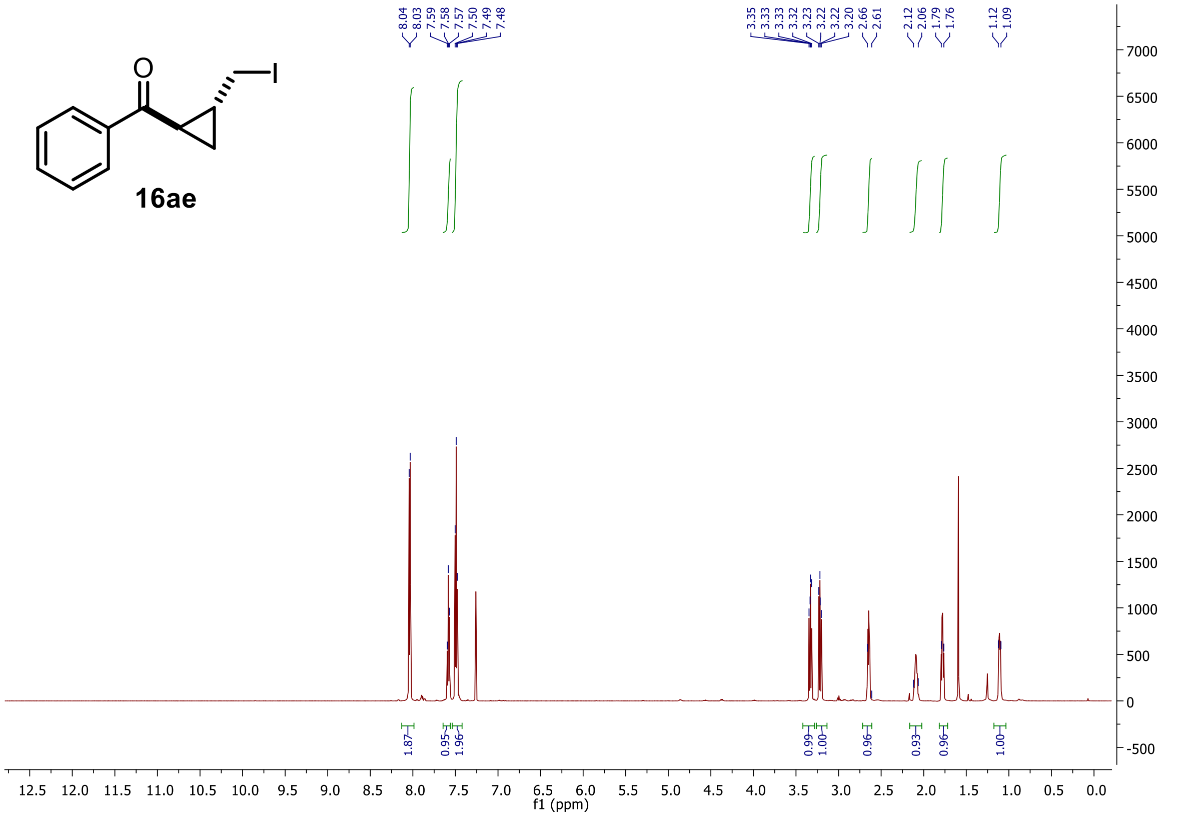
O
I
16ae
8.04
8.03
7.59
7.58
7.57
7.50
7.49
7.48
3.35
3.33
3.33
3.32
3.23
3.22
3.22
3.20
2.66
2.61
2.12
2.06
1.79
1.76
1.12
1.09
1.87
0.95
1.96
0.99
1.00
0.96
0.93
0.96
1.00
f1 (ppm)

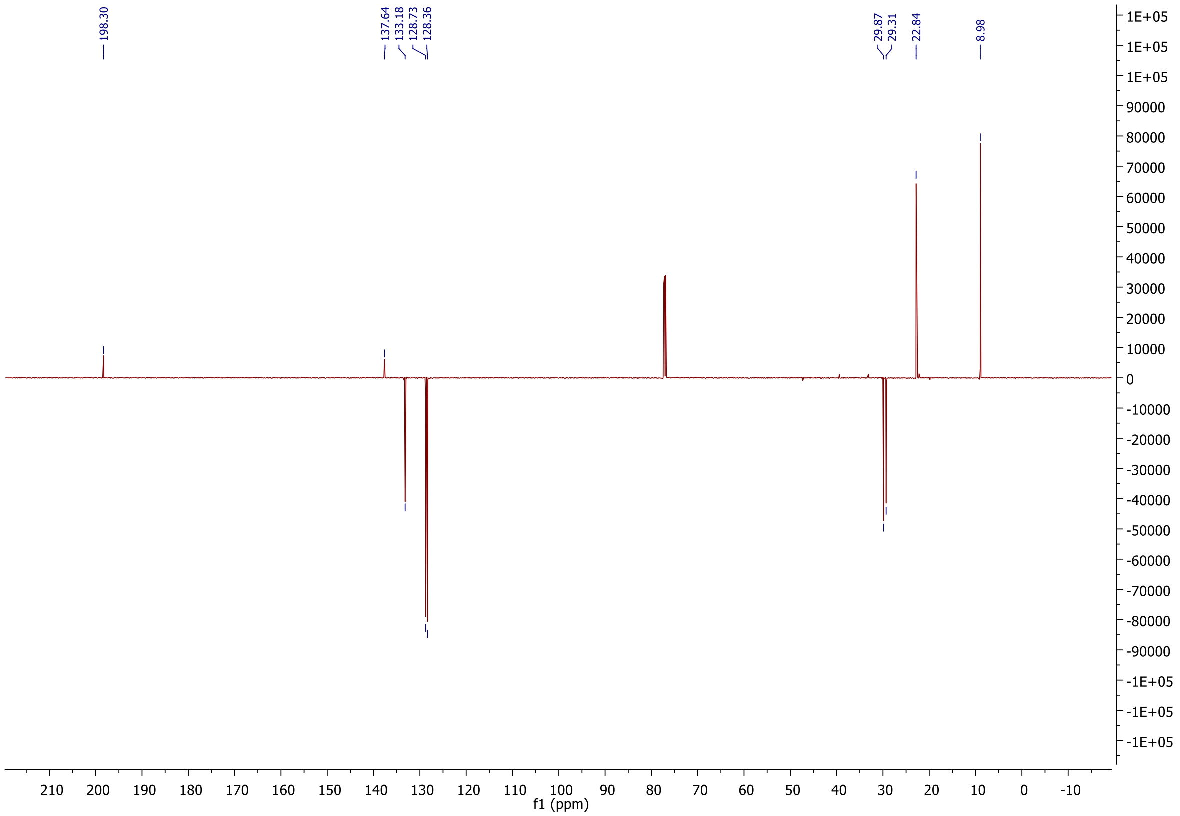
198.30
137.64
133.18
128.73
128.36
29.87
29.31
22.84
8.98
f1 (ppm)

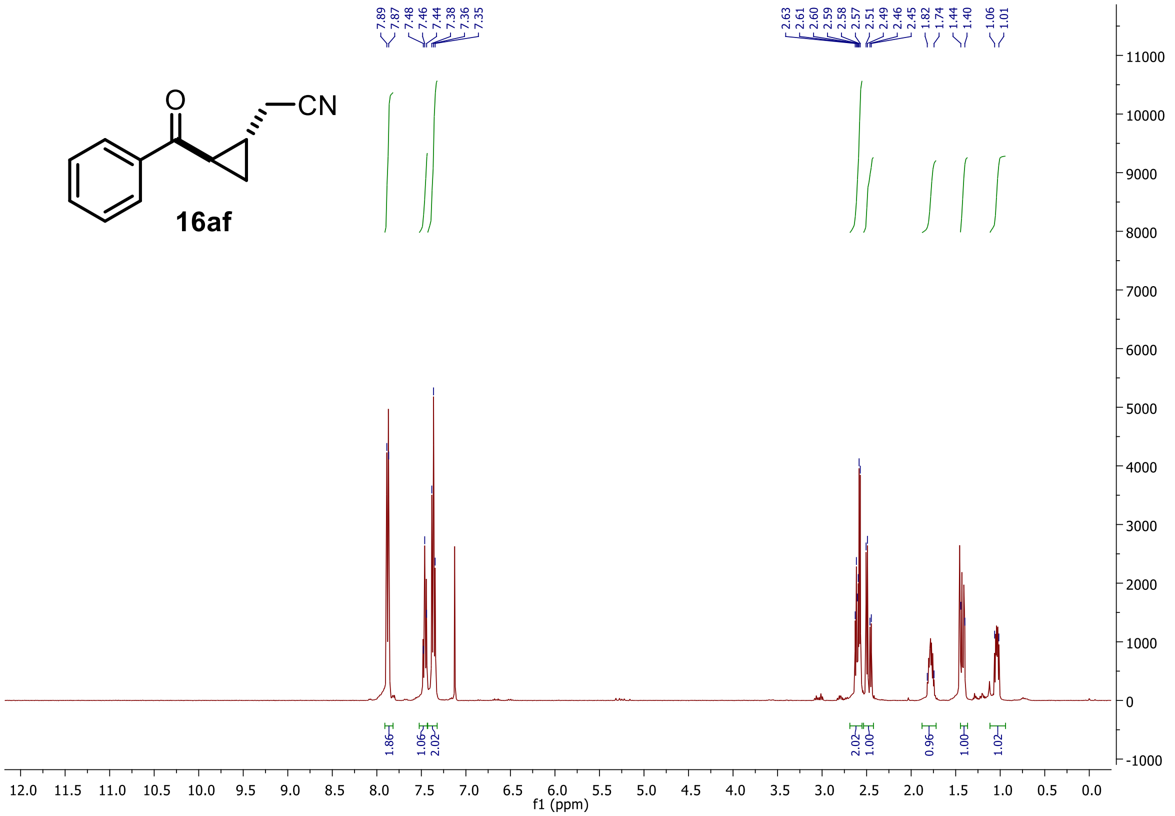
16af
CN
O
f1 (ppm)

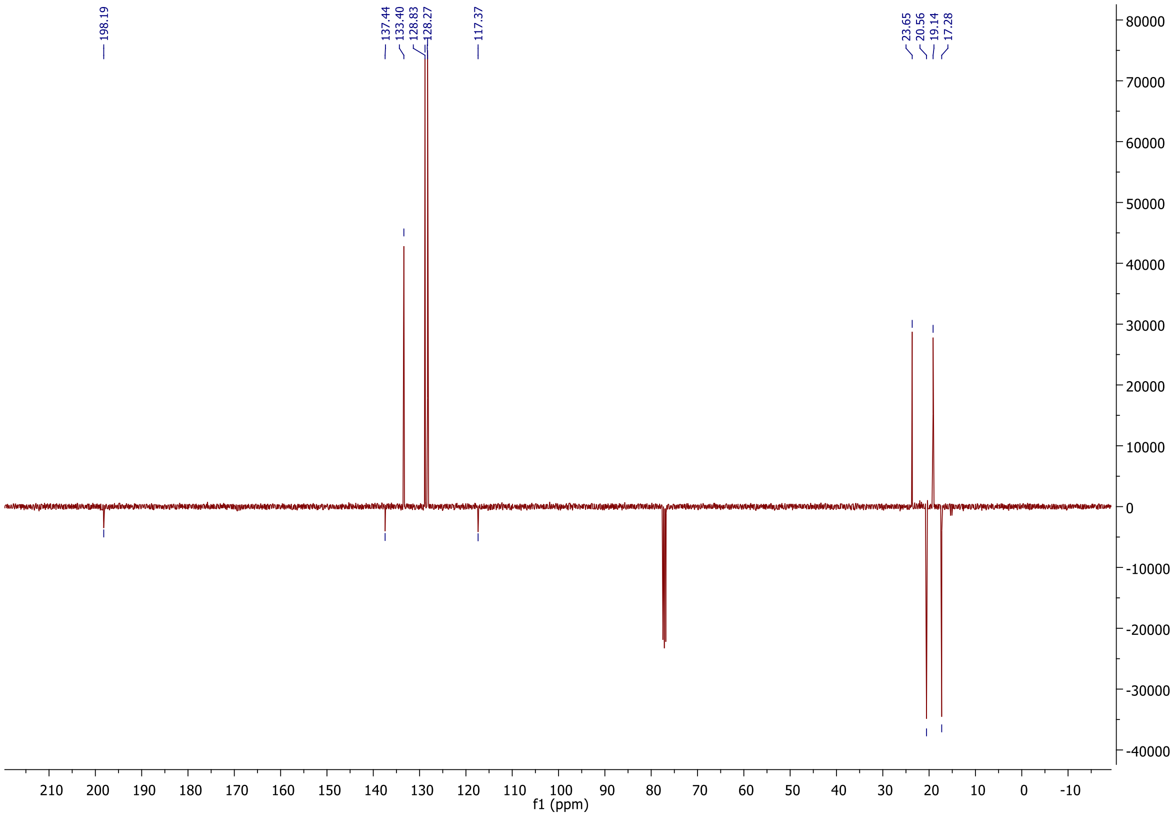
198.19
137.44
133.40
128.83
128.27
117.37
23.65
20.56
19.14
17.28
f1 (ppm)

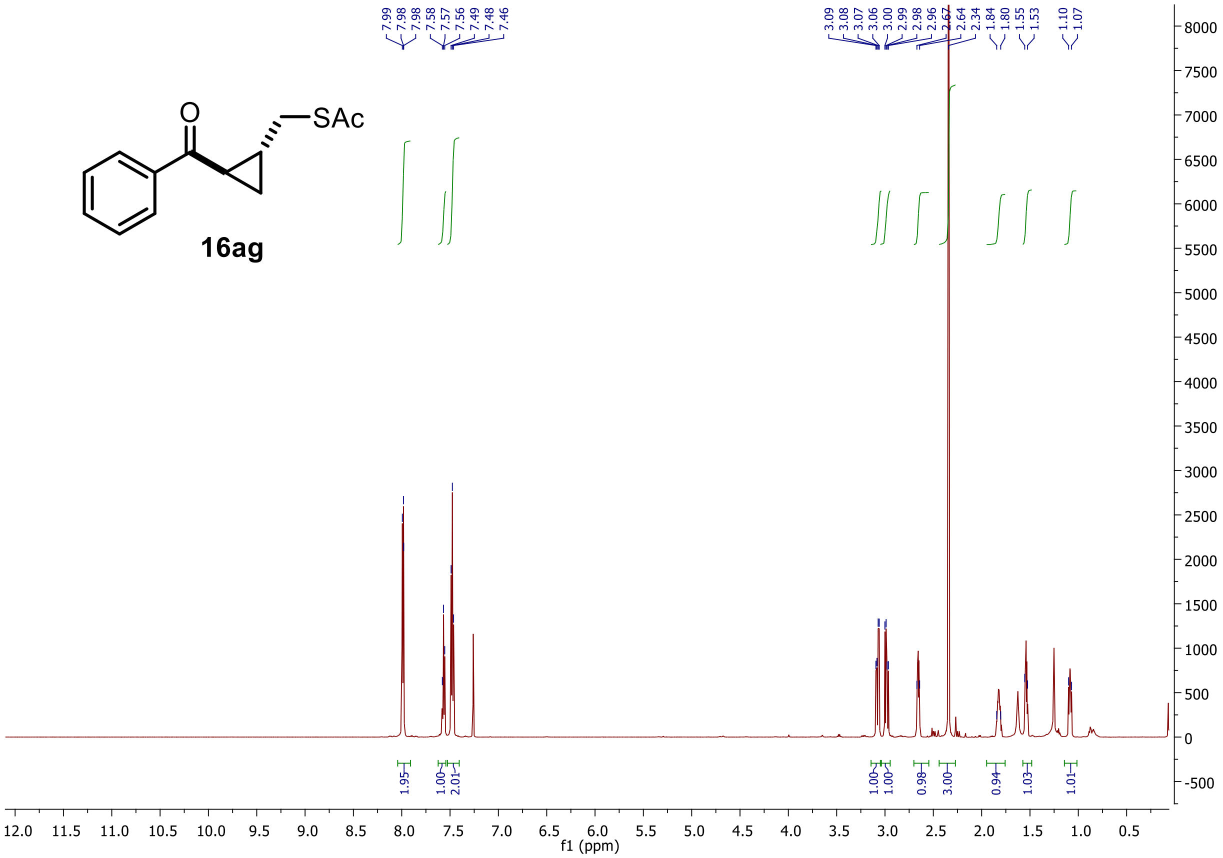
SAc
O
16ag
7.99
7.98
7.98
7.58
7.57
7.56
7.49
7.48
7.46
3.09
3.08
3.07
3.06
3.00
2.99
2.98
2.96
2.67
2.64
2.34
1.84
1.80
1.55
1.53
1.10
1.07
1.95
1.00
2.01
1.00
1.00
0.98
3.00
0.94
1.03
1.01
f1 (ppm)

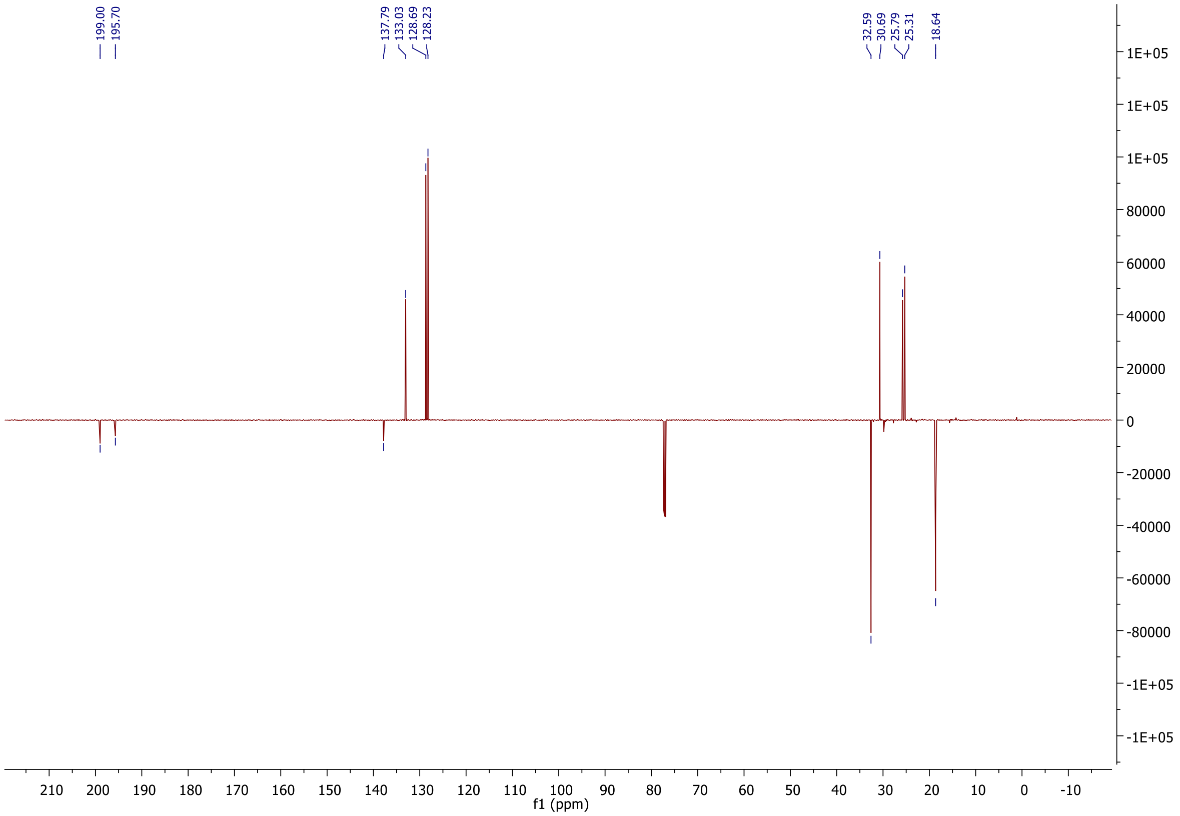
199.00
195.70
137.79
133.03
128.69
128.23
32.59
30.69
25.79
25.31
18.64
f1 (ppm)

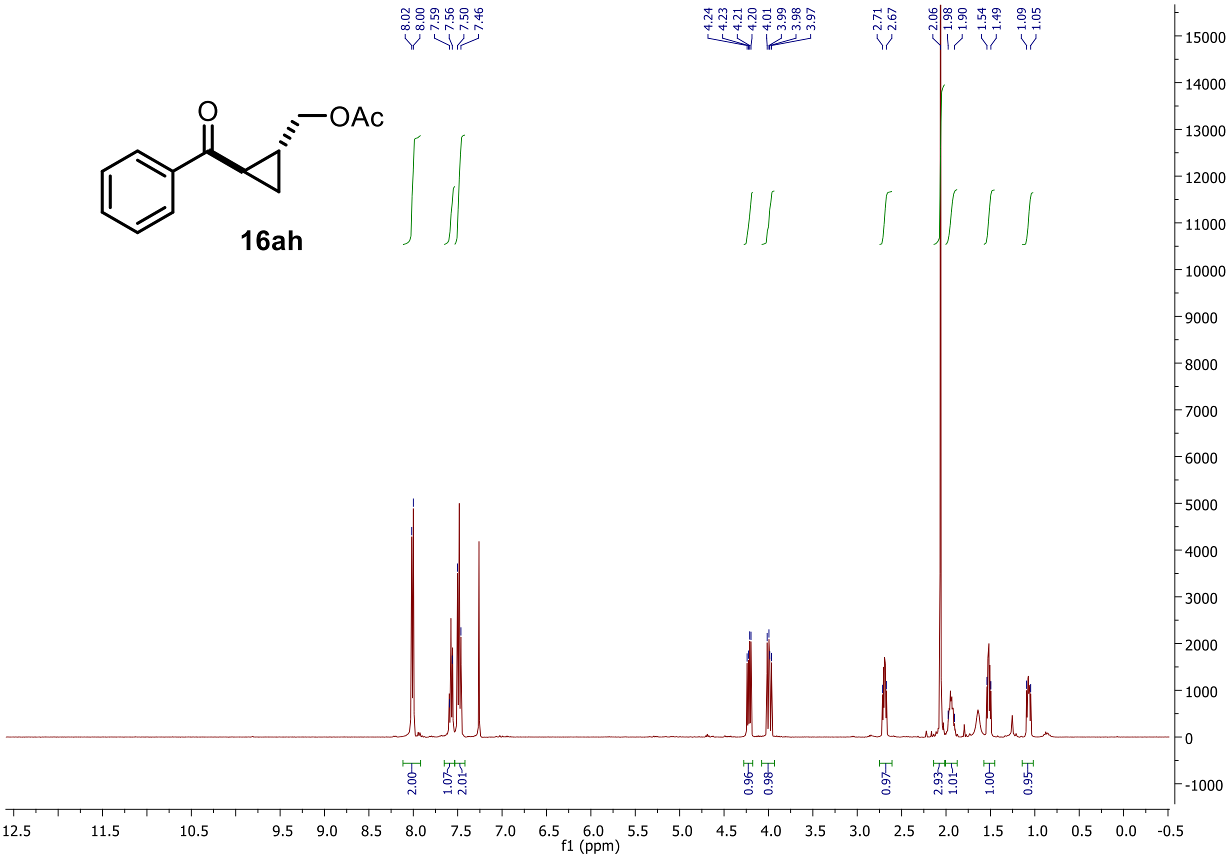
O
OAc
16ah
8.02
8.00
7.59
7.56
7.50
7.46
4.24
4.23
4.21
4.20
4.01
3.99
3.98
3.97
2.71
2.67
2.06
1.98
1.90
1.54
1.49
1.09
1.05
2.00
1.07
2.01
0.96
0.98
0.97
2.93
1.01
1.00
0.95
f1 (ppm)

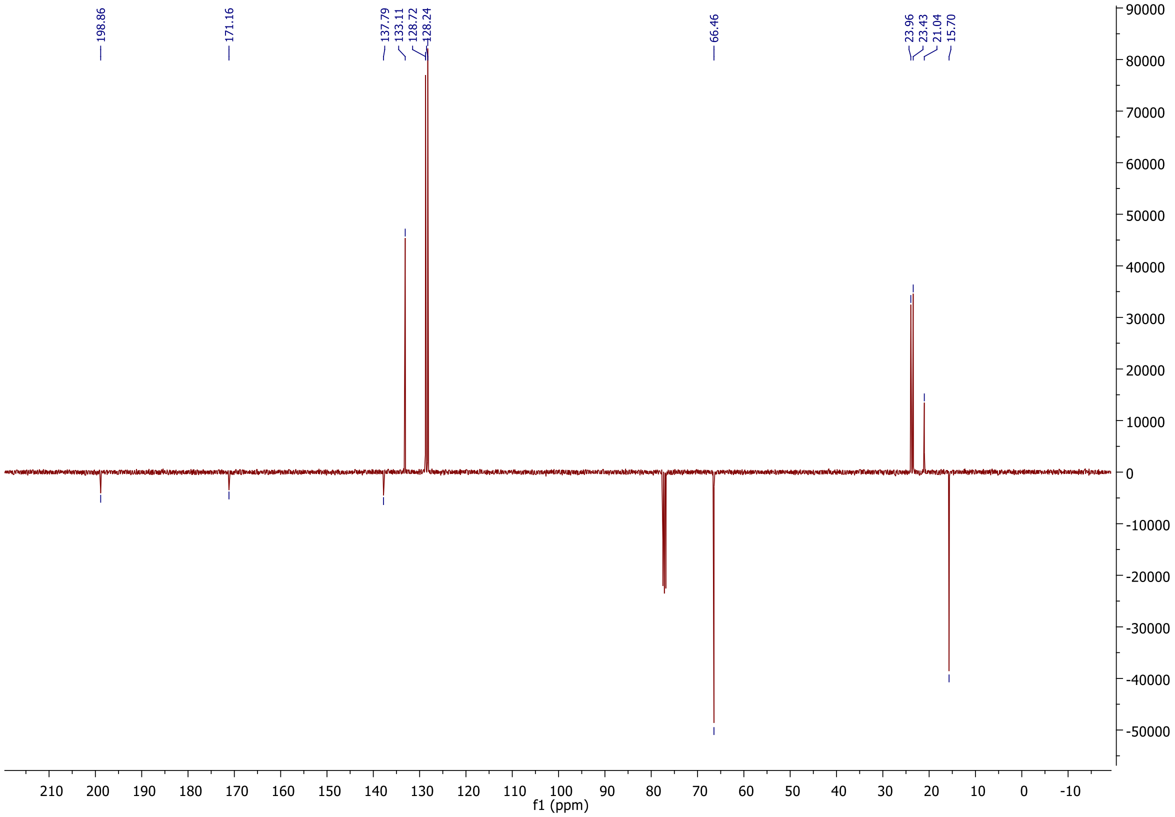
198.86
171.16
137.79
133.11
128.72
128.24
66.46
23.96
23.43
21.04
15.70
f1 (ppm)

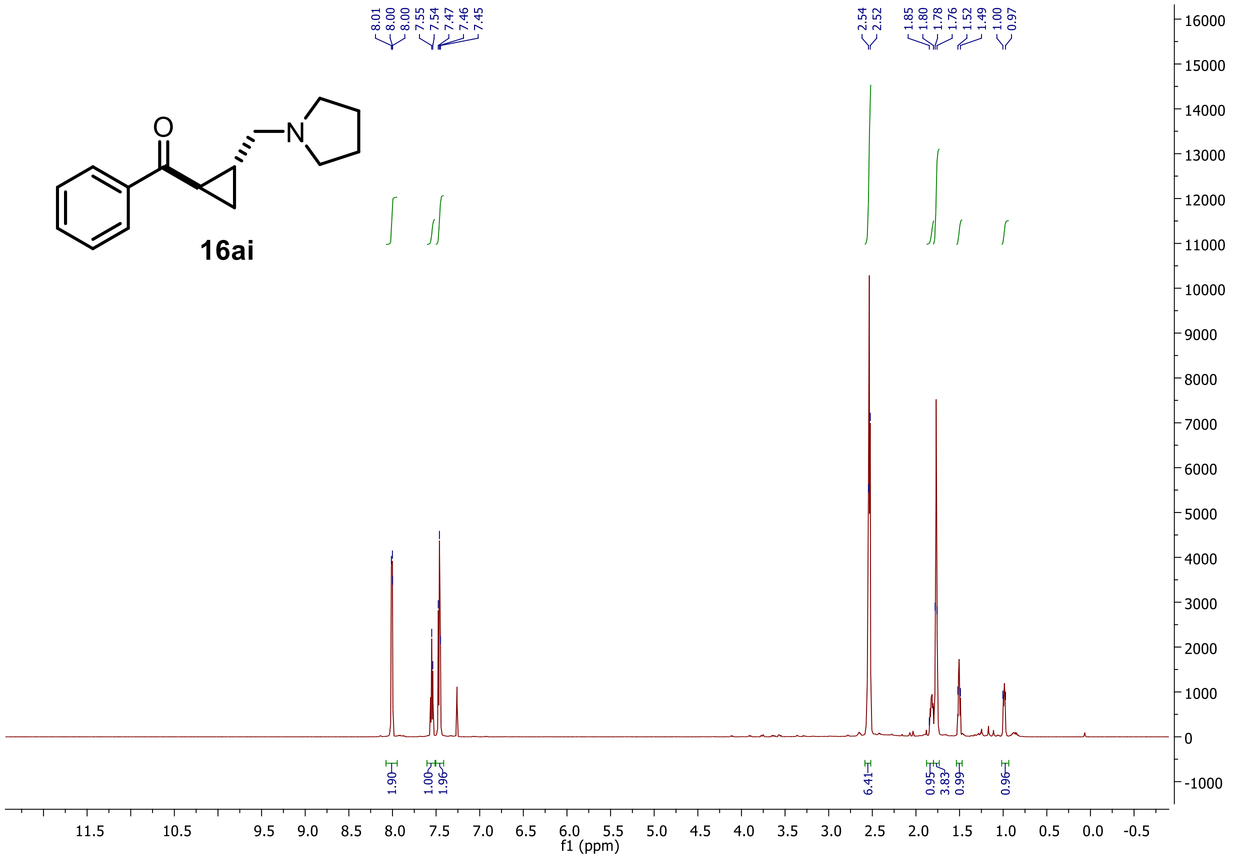
O
N
16ai
8.01 8.00 8.00 7.55 7.54 7.47 7.46 7.45
2.54 2.52
1.85 1.80 1.78 1.76 1.52 1.49 1.00 0.97
1.90 1.00 1.96 6.41 0.95 3.83 0.99 0.96
f1 (ppm)

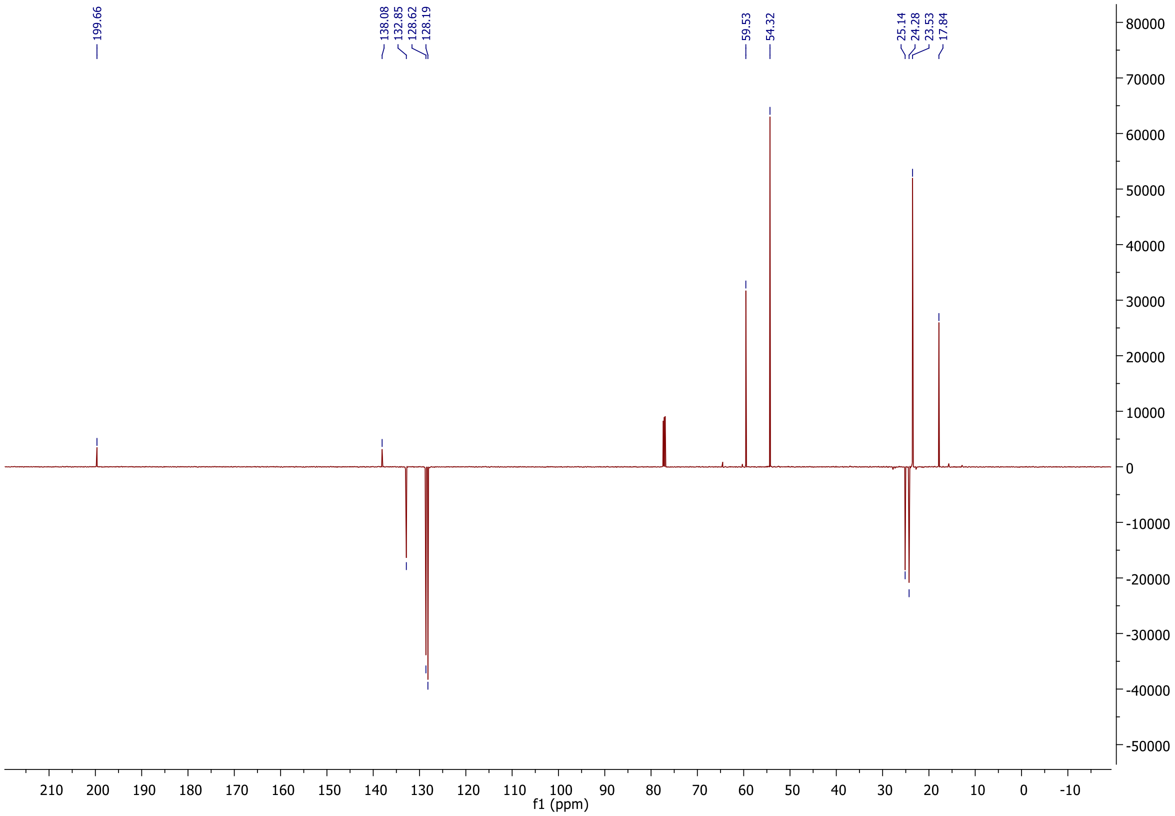
199.66
138.08 132.85 128.62 128.19
59.53 54.32
25.14 24.28 23.53 17.84
f1 (ppm)

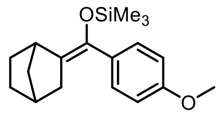
OSiMe3
O

17a

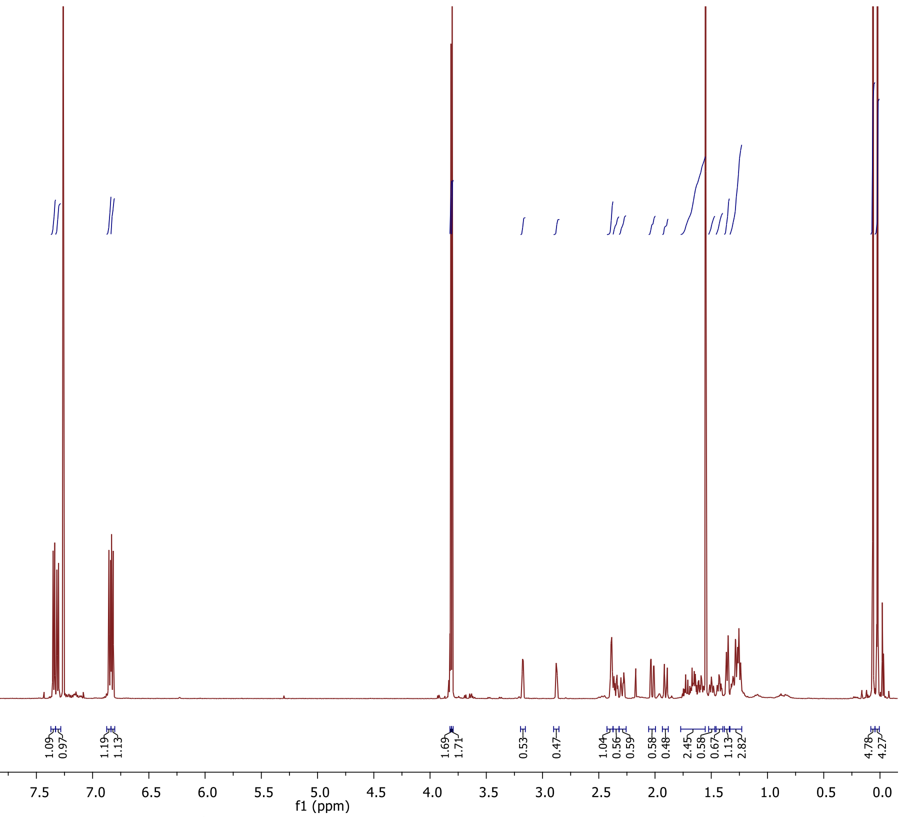
1.09
0.97
1.19
1.13
1.69
1.71
0.53
0.47
1.04
0.56
0.59
0.58
0.48
2.45
0.56
0.67
1.13
2.82
4.78
4.27
10.0 9.5 9.0 8.5 8.0 7.5 7.0 6.5 6.0 5.5 5.0 4.5 4.0 3.5 3.0 2.5 2.0 1.5 1.0 0.5 0.0
f1 (ppm)

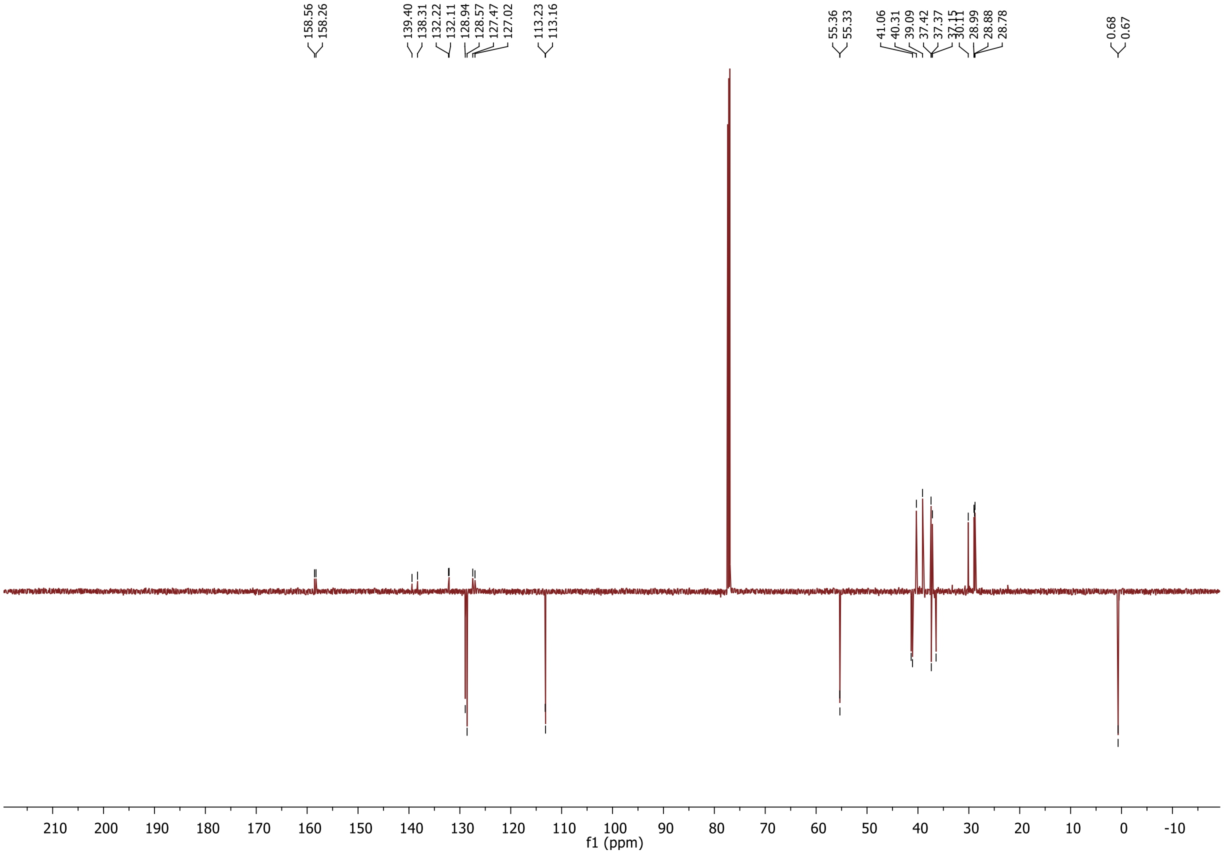
158.56
158.26
139.40
138.31
132.22
132.11
128.94
128.57
127.47
127.02
113.23
113.16
55.36
55.33
41.06
40.31
39.09
37.42
37.37
37.15
30.11
28.99
28.88
28.78
0.68
0.67
210 200 190 180 170 160 150 140 130 120 110 100 90 80 70 60 50 40 30 20 10 0 -10
f1 (ppm)

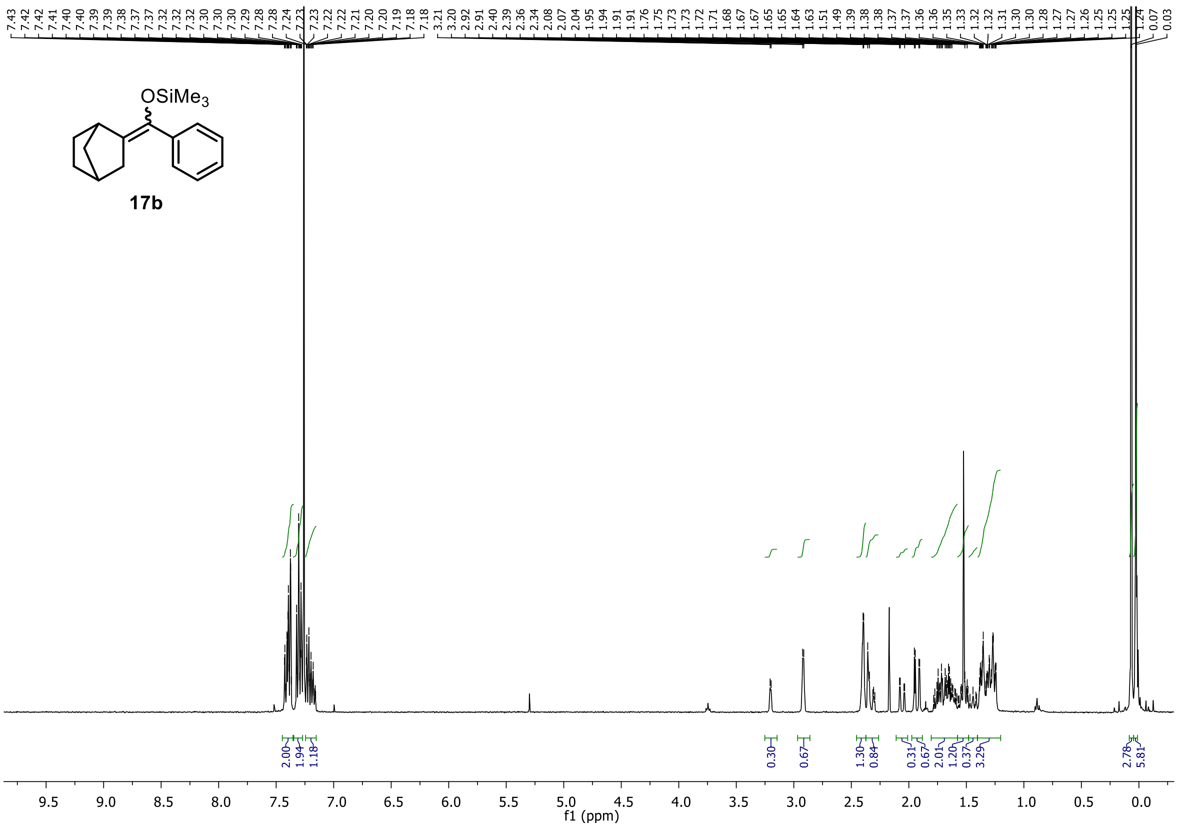
OSiMe3
17b
f1 (ppm)

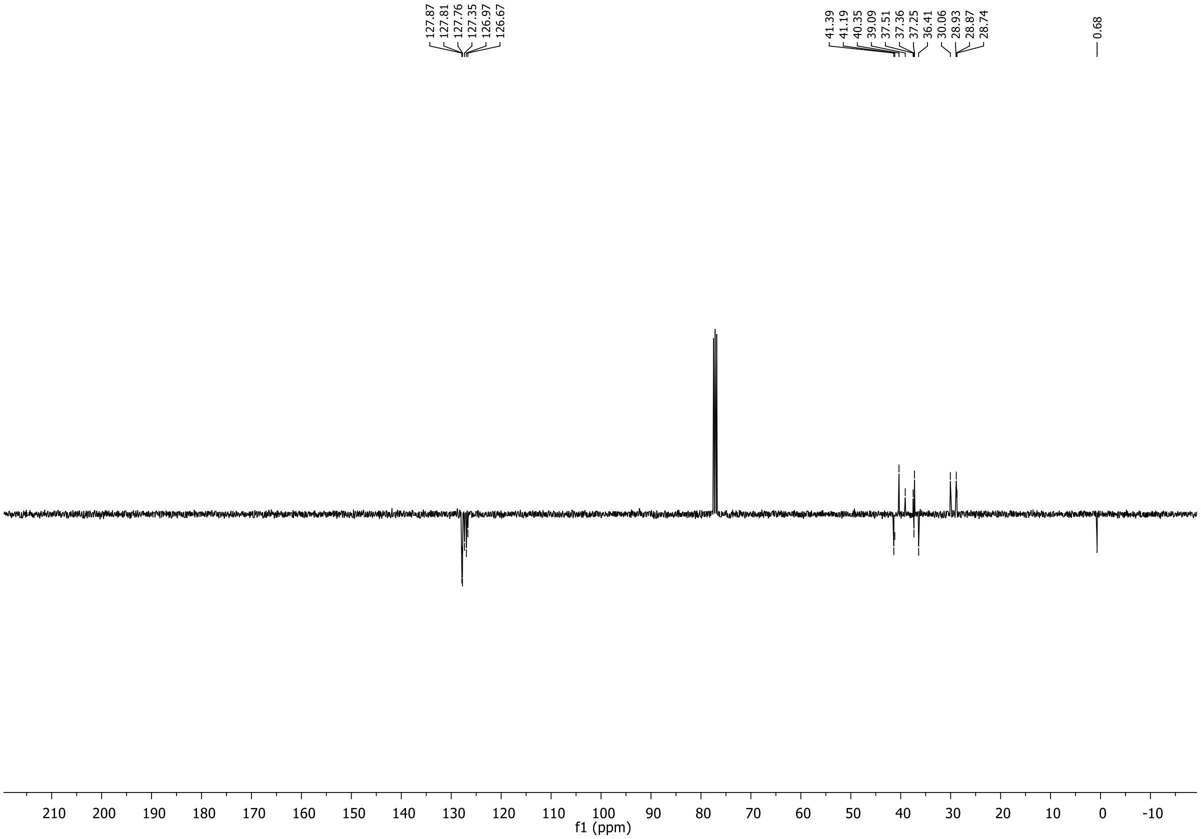
f1 (ppm)

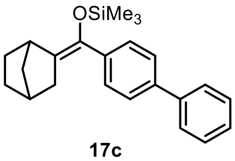
OSiMe3

17c

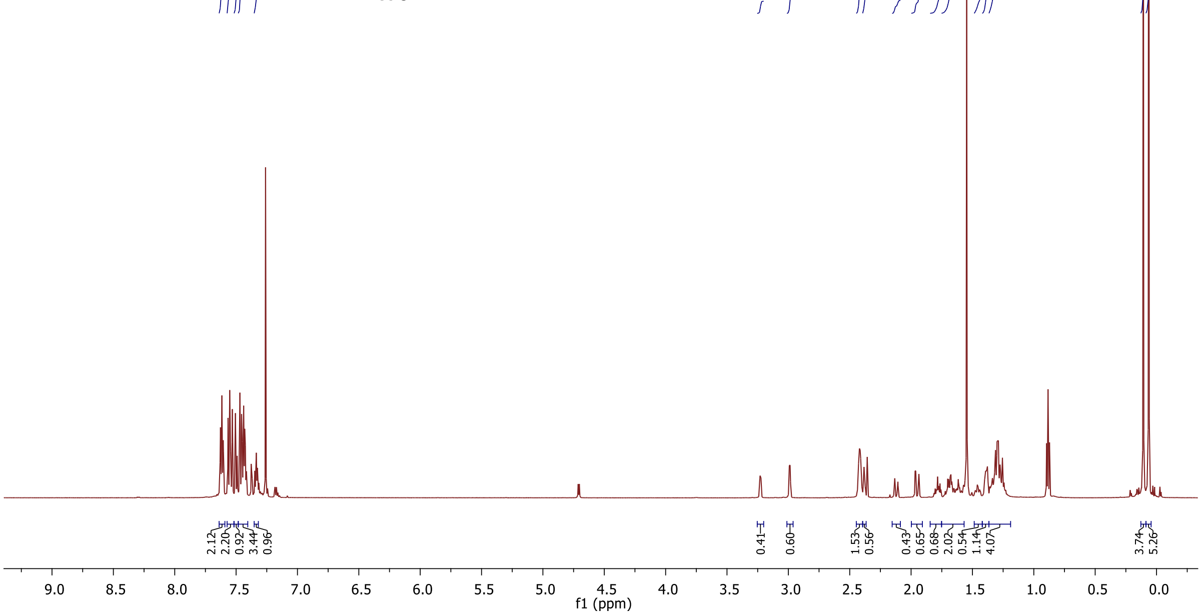
2.12
2.20
0.92
3.44
0.96
0.41
0.60
1.53
0.56
0.43
0.65
0.68
2.02
0.54
1.14
4.07
3.74
5.26
9.0 8.5 8.0 7.5 7.0 6.5 6.0 5.5 5.0 4.5 4.0 3.5 3.0 2.5 2.0 1.5 1.0 0.5 0.0
f1 (ppm)

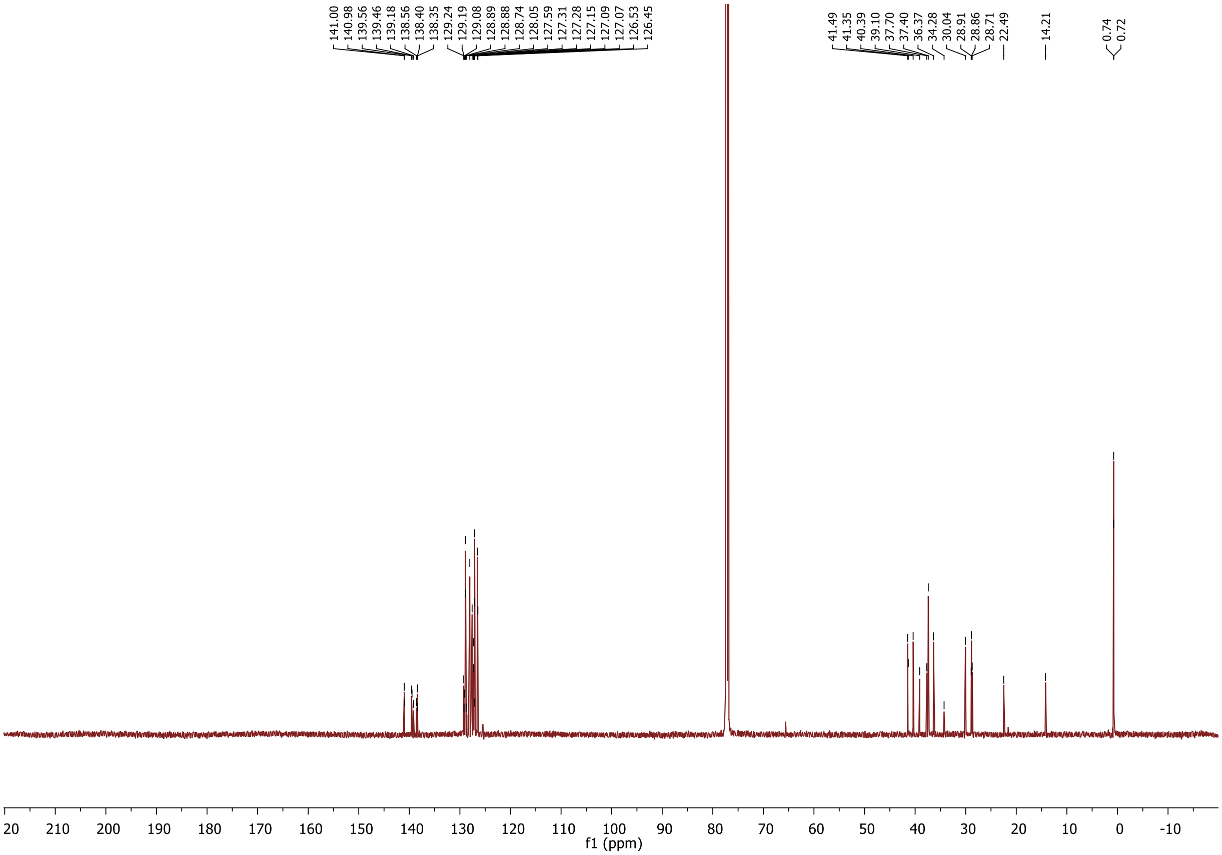
141.00
140.98
139.56
139.46
139.18
138.56
138.40
138.35
129.24
129.19
129.08
128.89
128.88
128.74
128.05
127.59
127.31
127.28
127.15
127.09
127.07
126.53
126.45
41.49
41.35
40.39
39.10
37.70
37.40
36.37
34.28
30.04
28.91
28.86
28.71
22.49
14.21
0.74
0.72
20 210 200 190 180 170 160 150 140 130 120 110 100 90 80 70 60 50 40 30 20 10 0 -10
f1 (ppm)

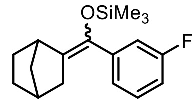
OSiMe3
F

17d

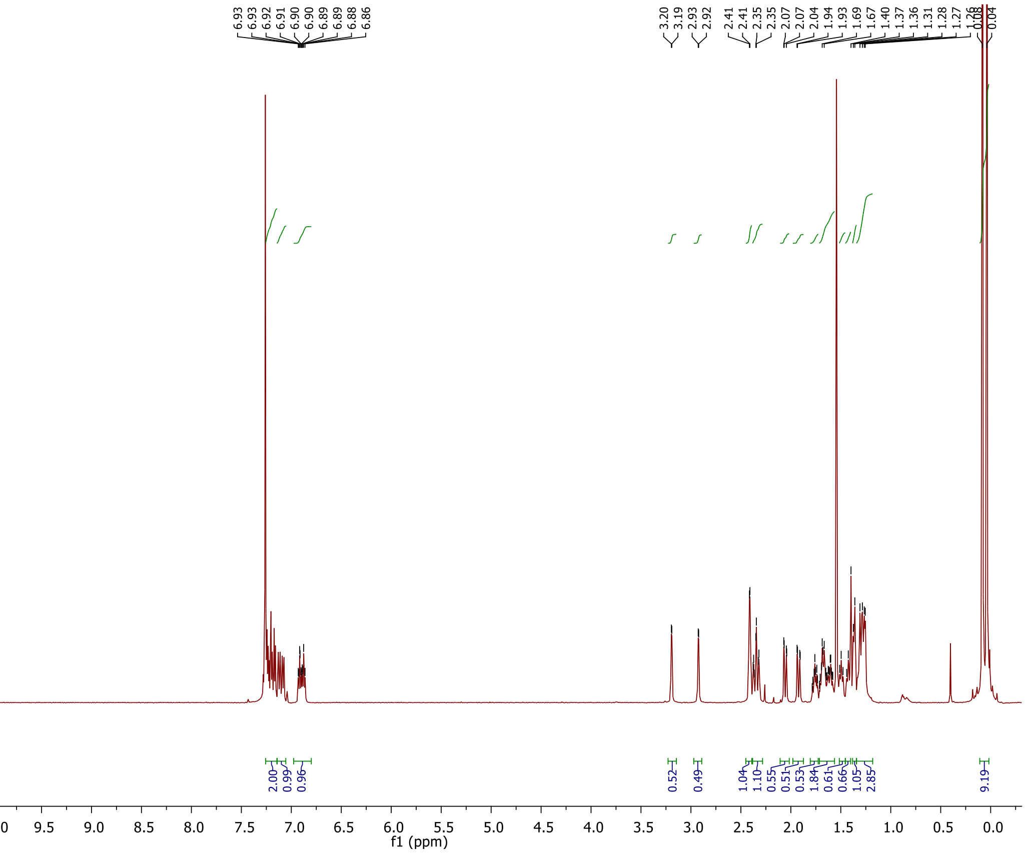
6.93 6.93 6.92 6.91 6.90 6.90 6.89 6.89 6.88 6.86
3.20 3.19 2.93 2.92 2.41 2.41 2.35 2.35 2.07 2.07 2.04 1.94 1.93 1.69 1.67 1.40 1.37 1.36 1.31 1.28 1.27 1.26 0.08 0.04
2.00 0.99 0.96 0.52 0.49 1.04 1.10 0.55 0.51 0.53 1.84 0.61 0.66 1.05 2.85 9.19
11.5 11.0 10.5 10.0 9.5 9.0 8.5 8.0 7.5 7.0 6.5 6.0 5.5 5.0 4.5 4.0 3.5 3.0 2.5 2.0 1.5 1.0 0.5 0.0
f1 (ppm)

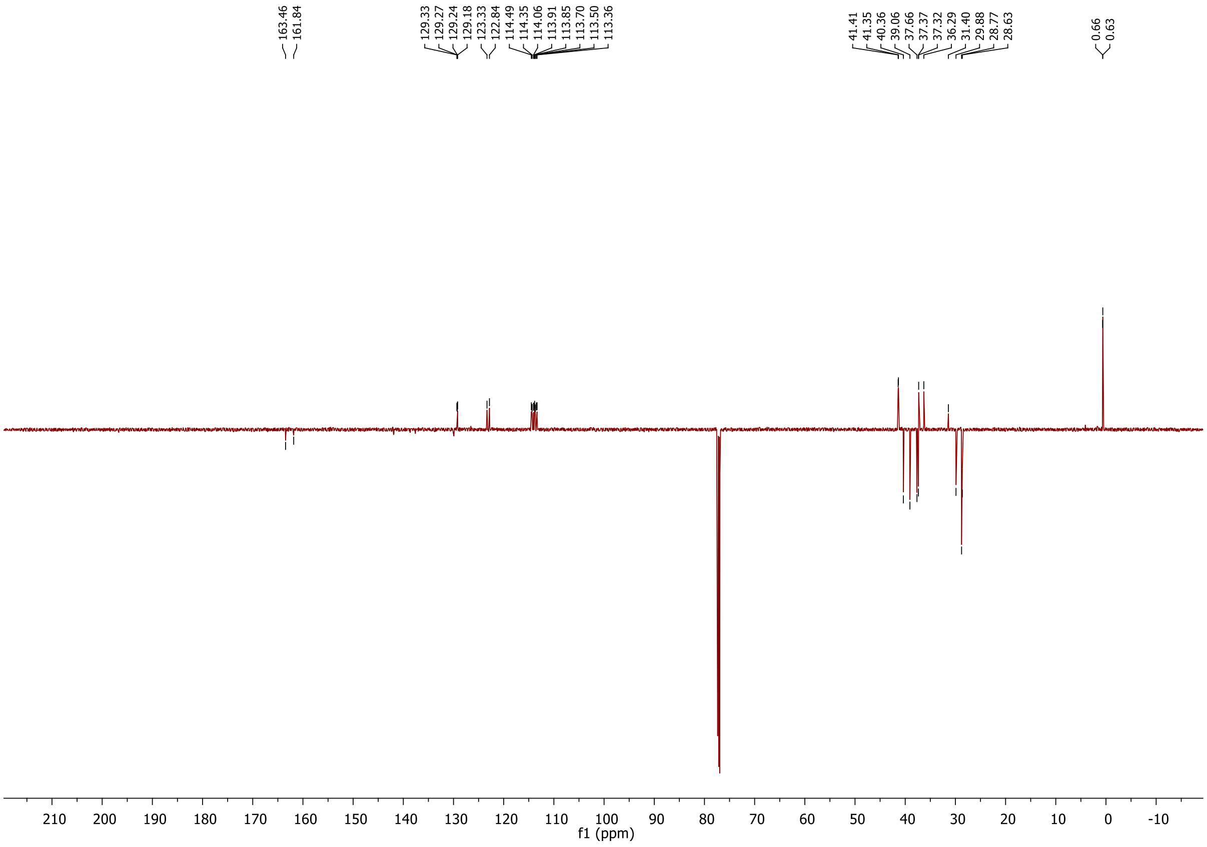
163.46 161.84
129.33 129.27 129.24 129.18 123.33 122.84 114.49 114.35 114.06 113.91 113.85 113.70 113.50 113.36
41.41 41.35 40.36 39.06 37.66 37.37 37.32 36.29 31.40 29.88 28.77 28.63
0.66 0.63
210 200 190 180 170 160 150 140 130 120 110 100 90 80 70 60 50 40 30 20 10 0 -10
f1 (ppm)

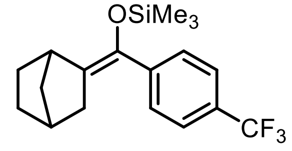
OSiMe3
CF3

17e

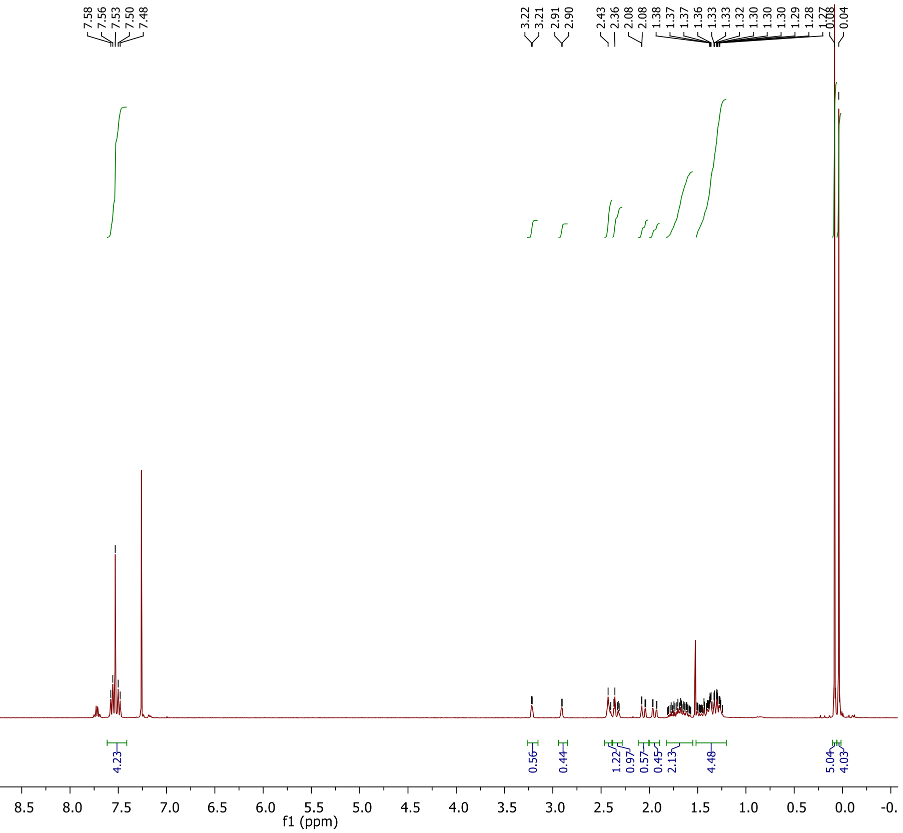
7.58
7.56
7.53
7.50
7.48
3.22
3.21
2.91
2.90
2.43
2.36
2.08
2.08
1.38
1.37
1.37
1.36
1.33
1.33
1.32
1.30
1.30
1.30
1.29
1.28
1.27
0.08
0.04
4.23
0.56
0.44
1.22
0.97
0.57
0.45
2.13
4.48
5.04
4.03
11.5 11.0 10.5 10.0 9.5 9.0 8.5 8.0 7.5 7.0 6.5 6.0 5.5 5.0 4.5 4.0 3.5 3.0 2.5 2.0 1.5 1.0 0.5 0.0 -0.
f1 (ppm)

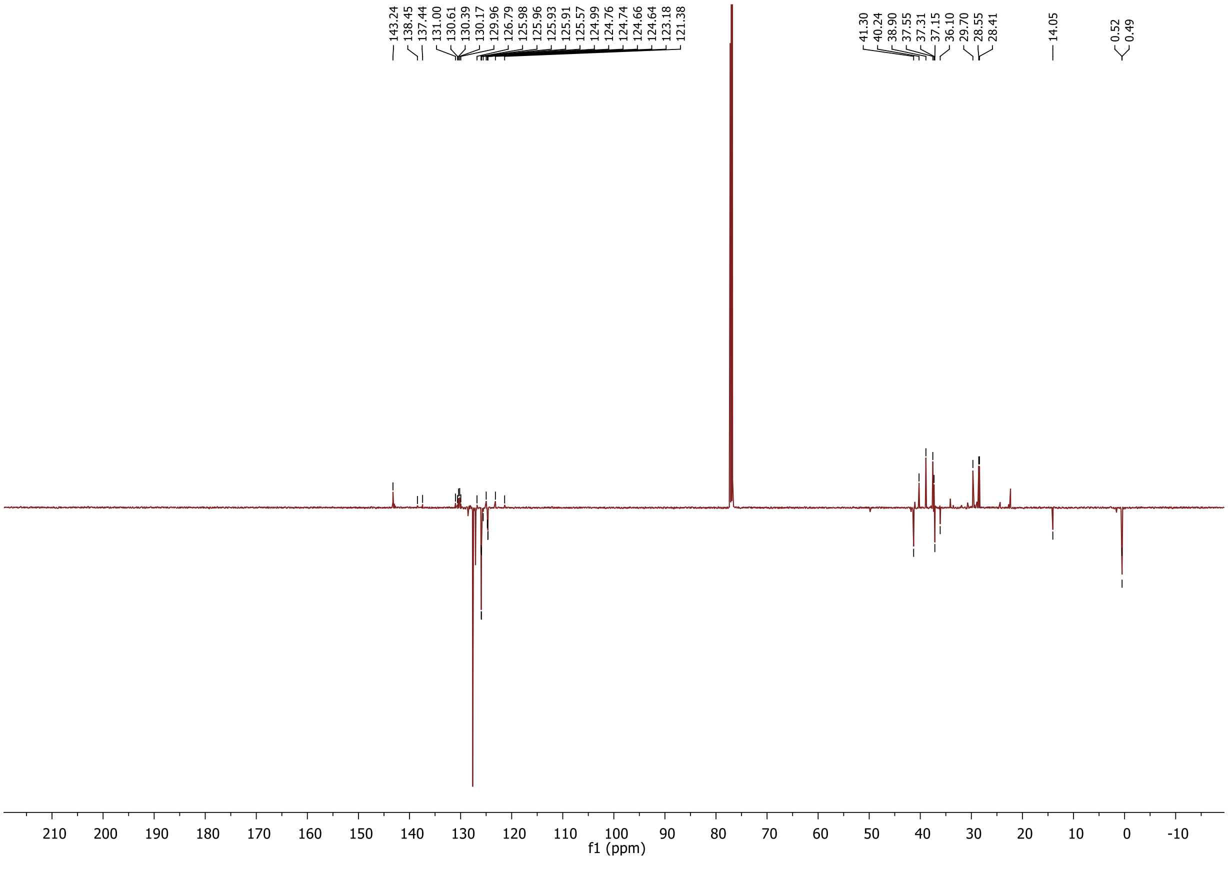
143.24
138.45
137.44
131.00
130.61
130.39
130.17
129.96
126.79
125.98
125.96
125.93
125.91
125.57
124.99
124.76
124.74
124.66
124.64
123.18
121.38
41.30
40.24
38.90
37.55
37.31
37.15
36.10
29.70
28.55
28.41
14.05
0.52
0.49
210 200 190 180 170 160 150 140 130 120 110 100 90 80 70 60 50 40 30 20 10 0 -10
f1 (ppm)

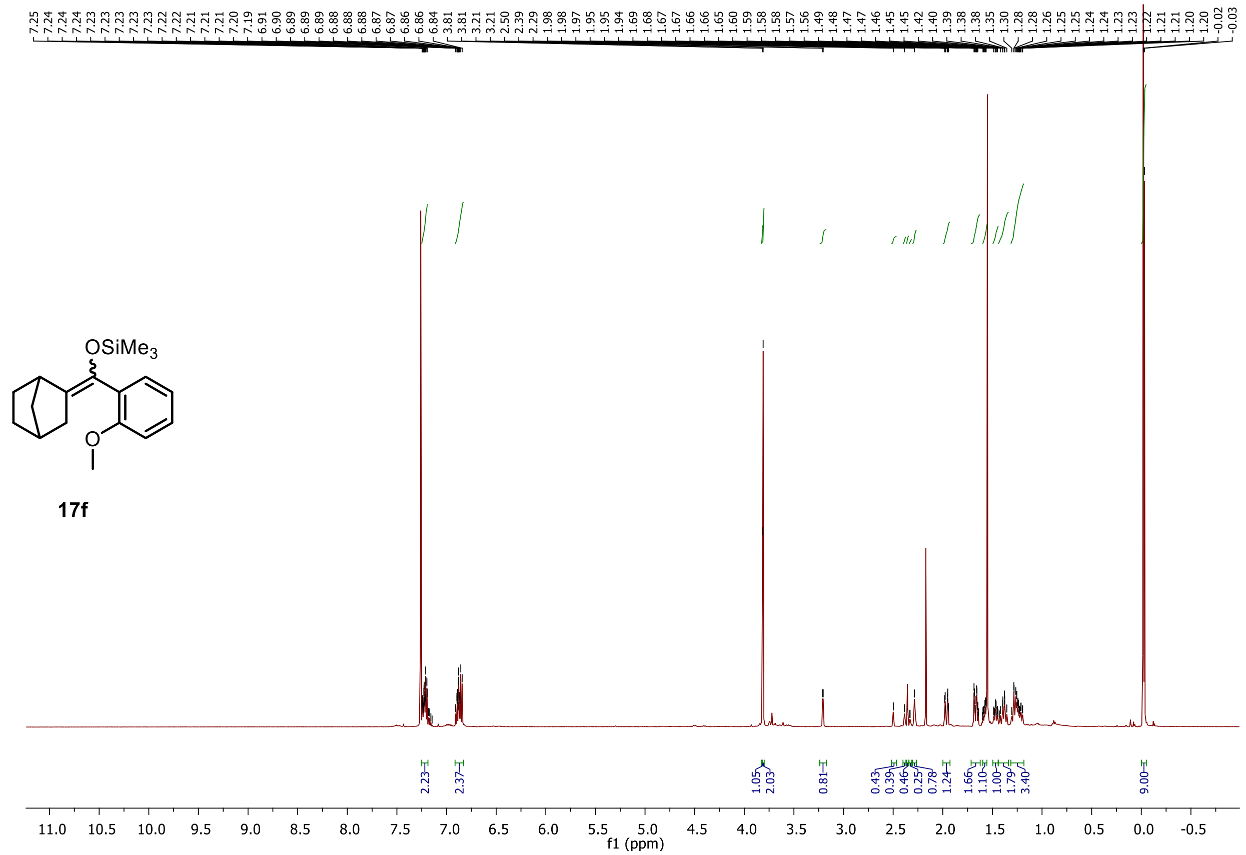
OSiMe3
O
f1 (ppm)

17f

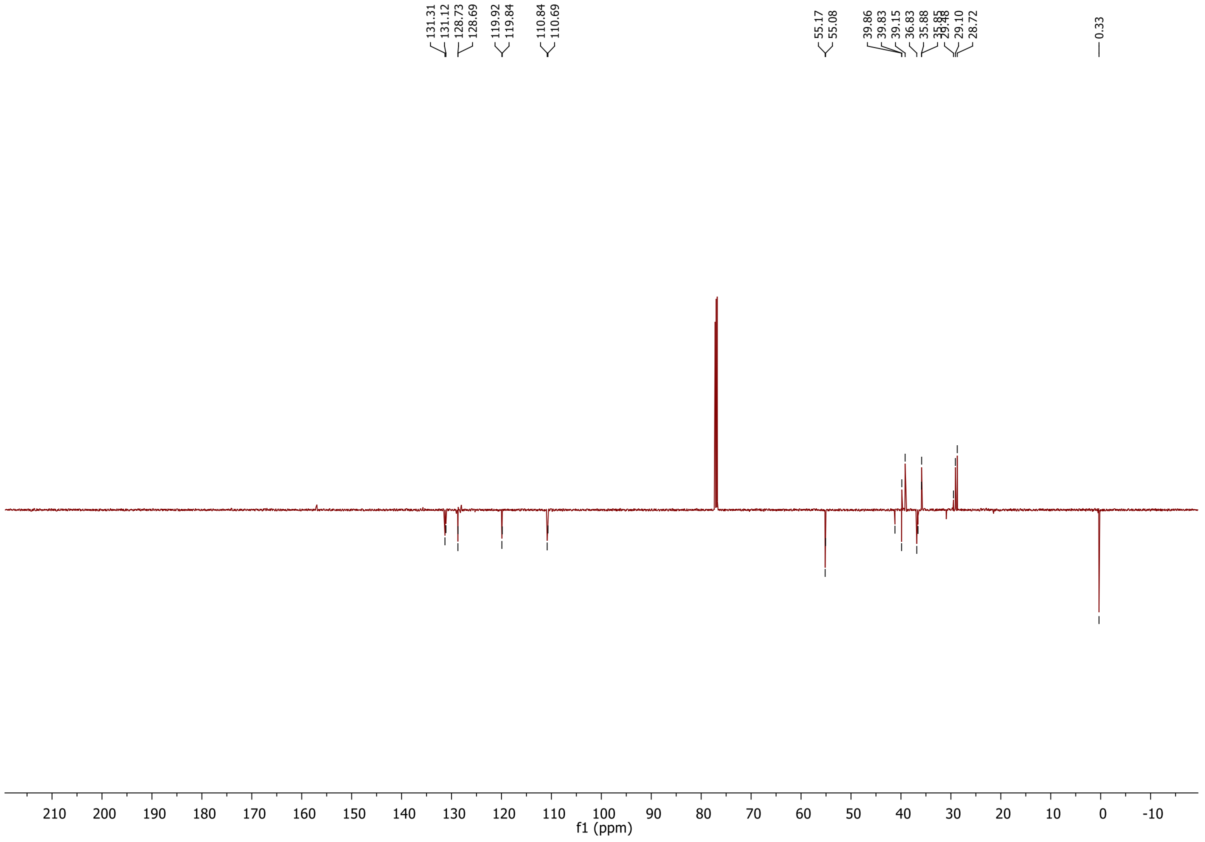
131.31
131.12
128.73
128.69
119.92
119.84
110.84
110.69
55.17
55.08
39.86
39.83
39.15
36.83
35.88
29.10
28.72
0.33
f1 (ppm)

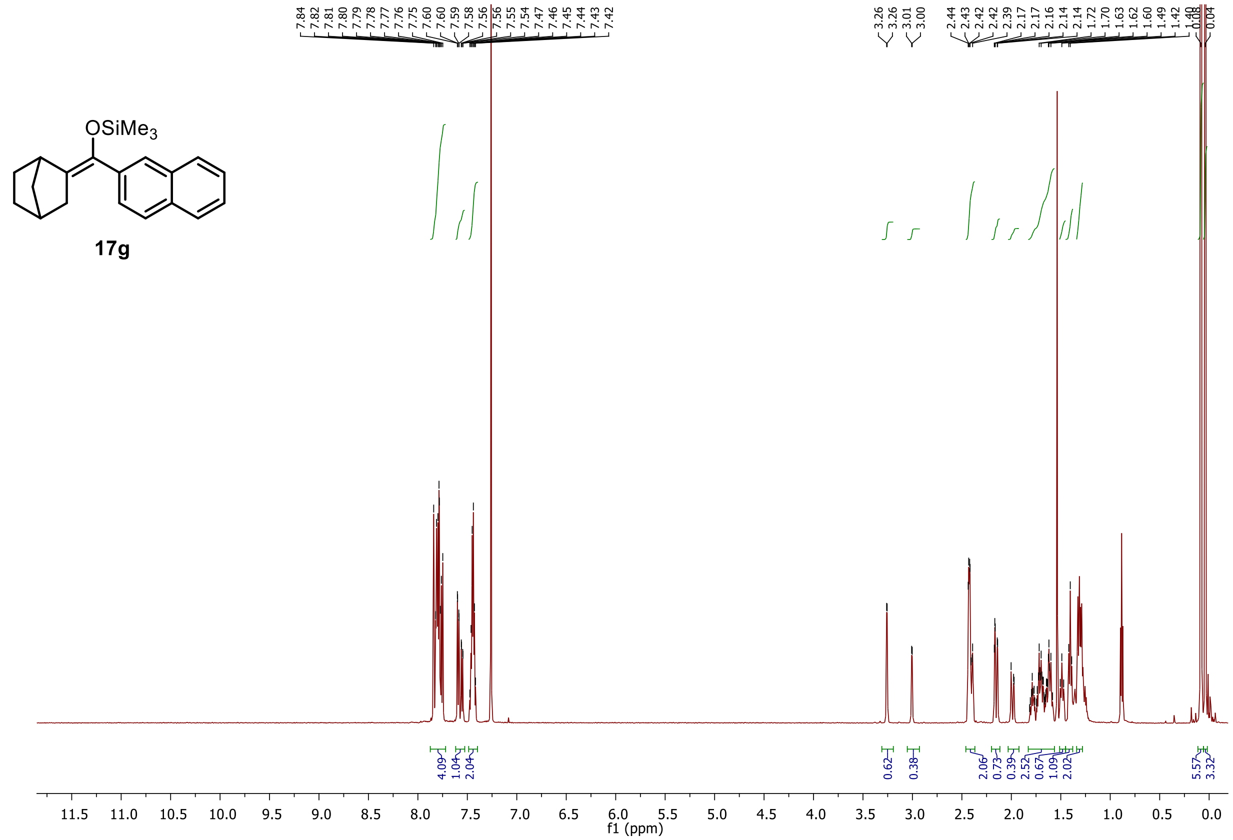
OSiMe3
17g
7.84 7.82 7.81 7.80 7.79 7.78 7.77 7.76 7.75 7.60 7.59 7.58 7.56 7.56 7.55 7.54 7.47 7.46 7.45 7.44 7.43 7.42
3.26 3.26 3.01 3.00
2.44 2.43 2.42 2.42 2.39 2.17 2.16 2.14 2.14 1.72 1.70 1.63 1.62 1.60 1.49 1.42 [illegible] 0.08 0.04
4.09 1.04 2.04 0.62 0.38 2.06 0.73 0.39 2.52 0.67 1.09 2.02 5.57 3.32
11.5 11.0 10.5 10.0 9.5 9.0 8.5 8.0 7.5 7.0 6.5 6.0 5.5 5.0 4.5 4.0 3.5 3.0 2.5 2.0 1.5 1.0 0.5 0.0
f1 (ppm)

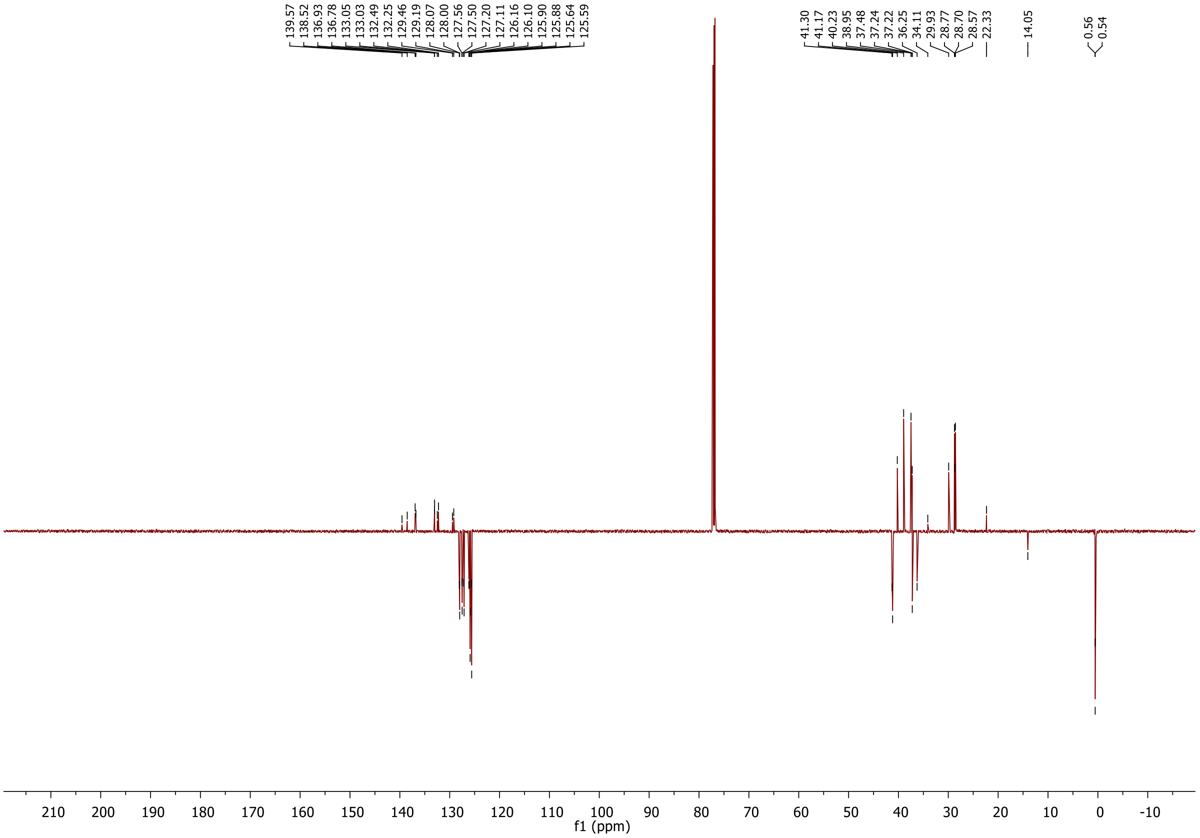
139.57 138.52 136.93 136.78 133.05 133.03 132.49 132.25 129.46 129.19 128.07 128.00 127.56 127.50 127.20 127.11 126.16 126.10 125.90 125.88 125.64 125.59
41.30 41.17 40.23 38.95 37.48 37.24 37.22 36.25 34.11 29.93 28.77 28.70 28.57 22.33
14.05
0.56 0.54
210 200 190 180 170 160 150 140 130 120 110 100 90 80 70 60 50 40 30 20 10 0 -10
f1 (ppm)

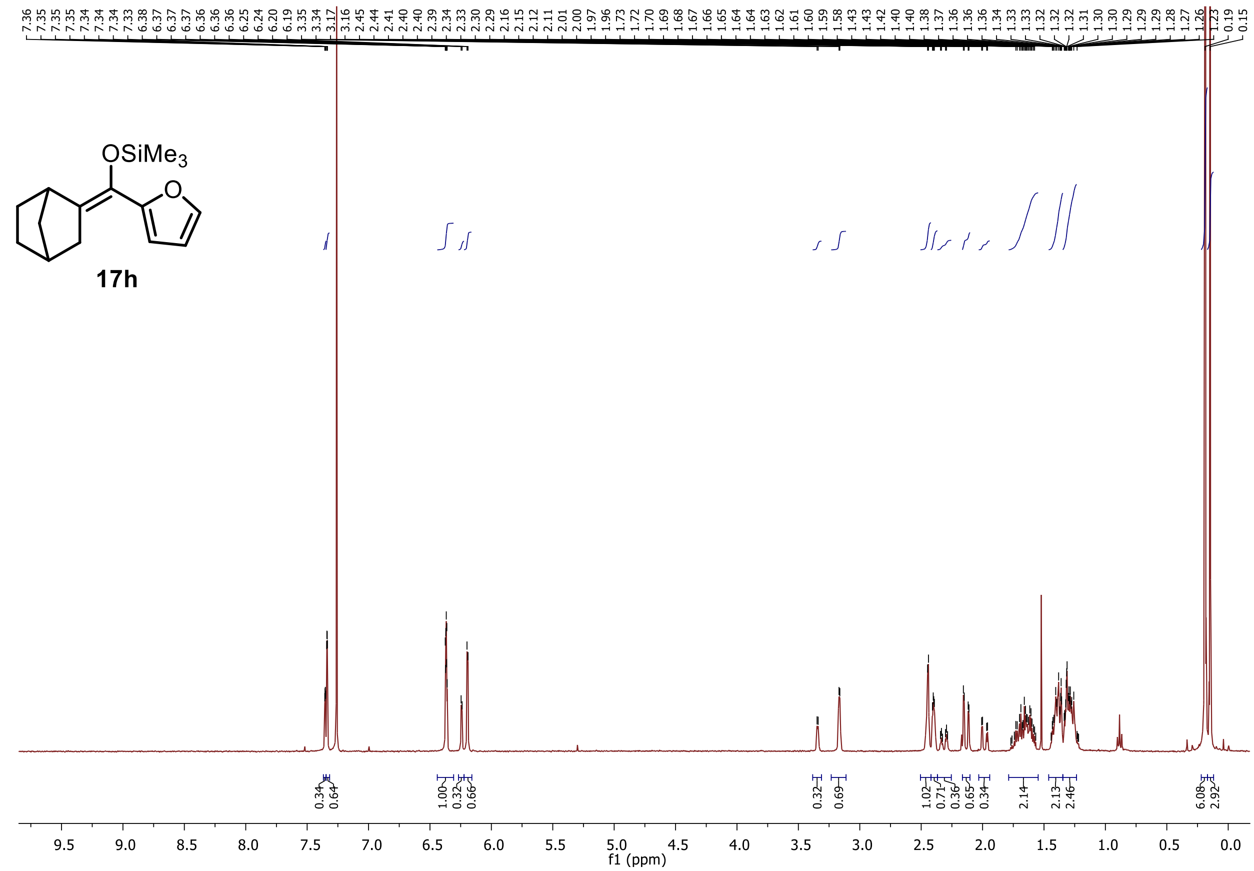
OSiMe3
O
17h
7.36
7.35
7.35
7.35
7.34
7.34
7.34
7.33
6.38
6.37
6.37
6.36
6.36
6.36
6.25
6.24
6.20
6.19
3.35
3.34
3.17
3.16
2.45
2.44
2.41
2.40
2.40
2.39
2.34
2.33
2.30
2.29
2.16
2.15
2.12
2.11
2.01
2.00
1.97
1.96
1.73
1.72
1.70
1.69
1.68
1.67
1.66
1.65
1.64
1.63
1.62
1.61
1.60
1.59
1.58
1.43
1.43
1.42
1.40
1.38
1.37
1.36
1.36
1.36
1.34
1.33
1.33
1.32
1.32
1.32
1.31
1.30
1.30
1.29
1.29
1.28
1.27
1.26
1.23
0.19
0.15
0.34
0.64
1.00
0.32
0.66
0.32
0.69
1.02
0.71
0.36
0.65
0.34
2.14
2.13
2.46
6.08
2.92
9.5 9.0 8.5 8.0 7.5 7.0 6.5 6.0 5.5 5.0 4.5 4.0 3.5 3.0 2.5 2.0 1.5 1.0 0.5 0.0
f1 (ppm)

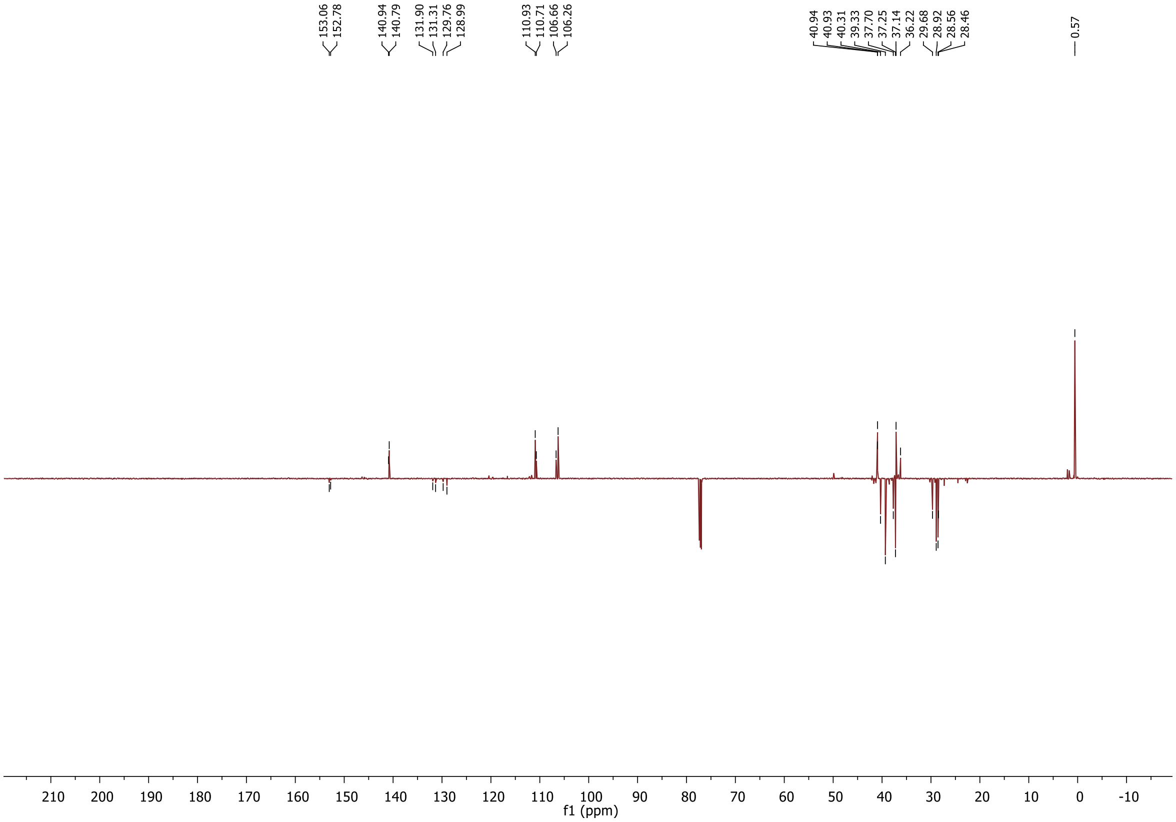
153.06
152.78
140.94
140.79
131.90
131.31
129.76
128.99
110.93
110.71
106.66
106.26
40.94
40.93
40.31
39.33
37.70
37.25
37.14
36.22
29.68
28.92
28.56
28.46
0.57
210 200 190 180 170 160 150 140 130 120 110 100 90 80 70 60 50 40 30 20 10 0 -10
f1 (ppm)

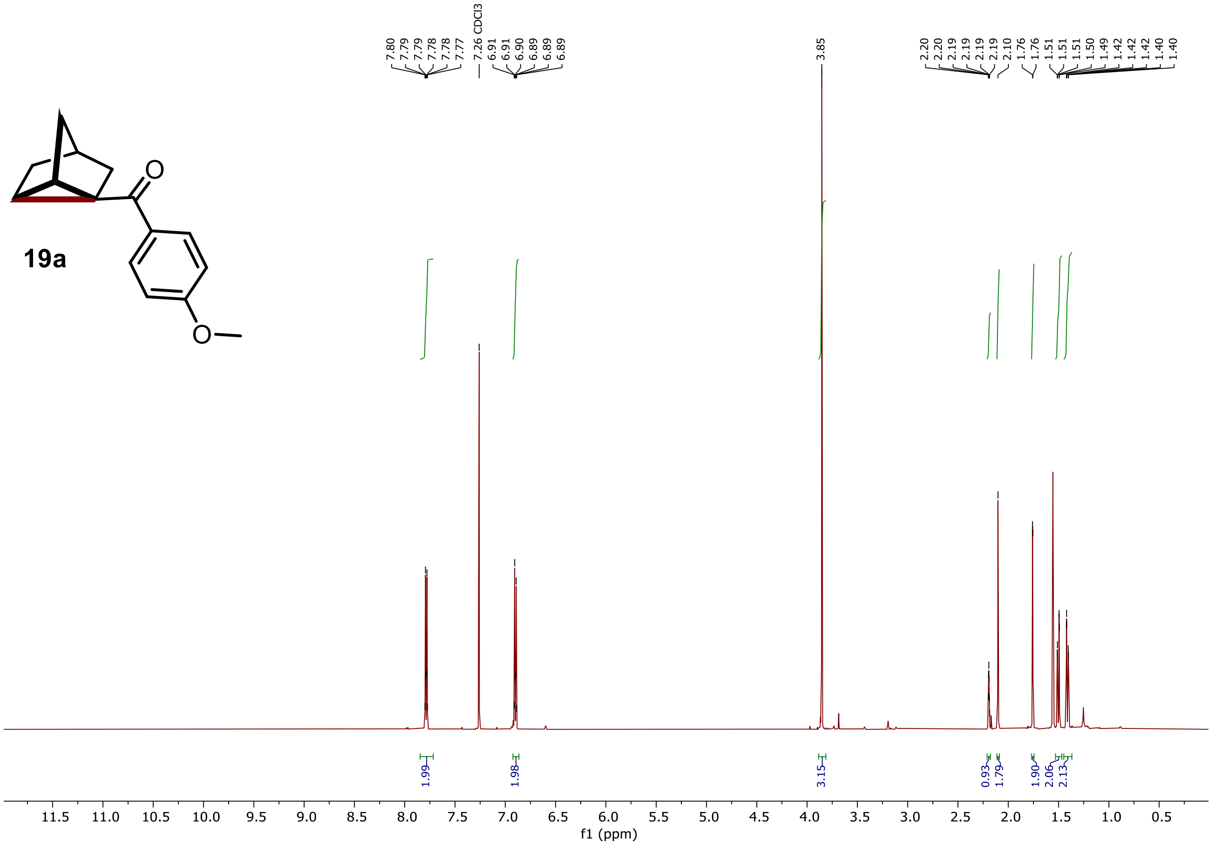
19a
O
7.80
7.79
7.79
7.78
7.78
7.77
7.26 CDCl3
6.91
6.91
6.90
6.89
6.89
6.89
3.85
2.20
2.20
2.19
2.19
2.19
2.19
2.10
1.76
1.76
1.51
1.51
1.51
1.50
1.49
1.42
1.42
1.42
1.40
1.40
1.99
1.98
3.15
0.93
1.79
1.90
2.06
2.13
11.5 11.0 10.5 10.0 9.5 9.0 8.5 8.0 7.5 7.0 6.5 6.0 5.5 5.0 4.5 4.0 3.5 3.0 2.5 2.0 1.5 1.0 0.5
f1 (ppm)

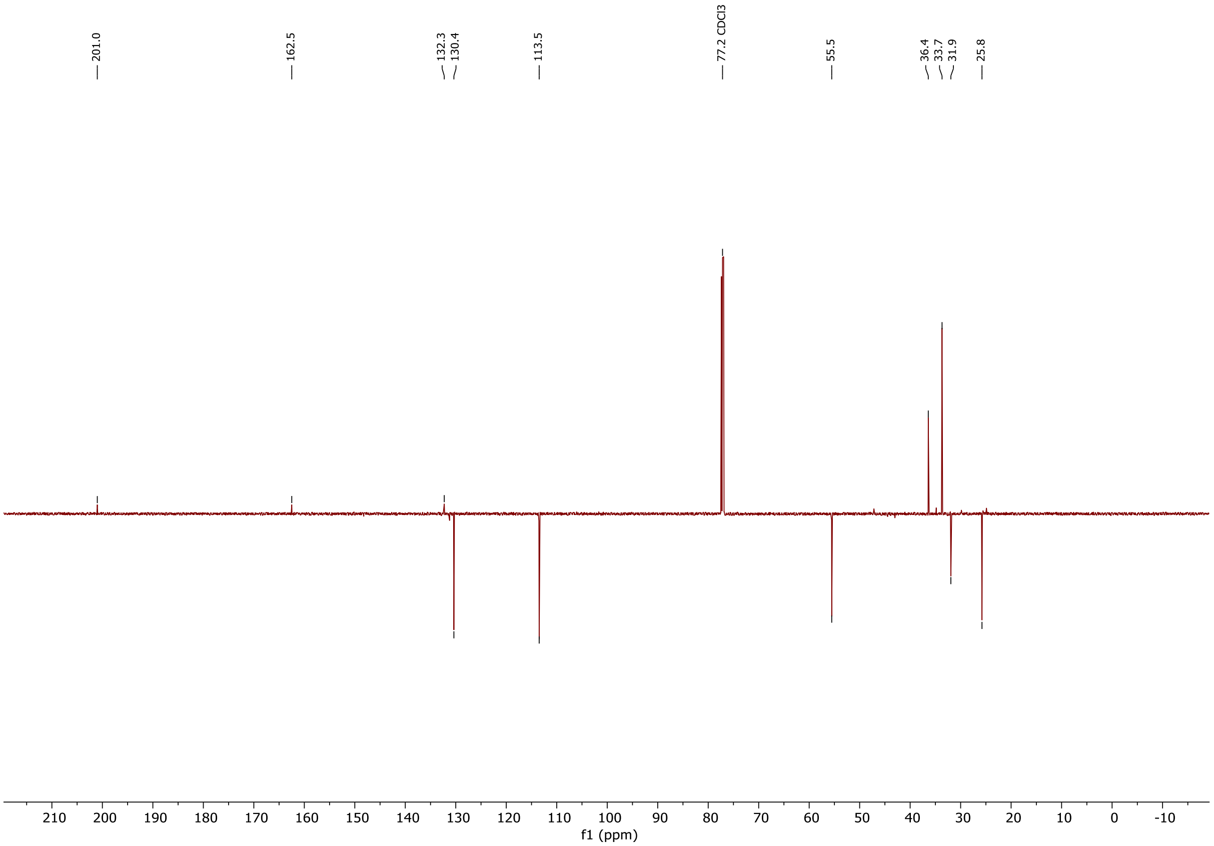
201.0
162.5
132.3
130.4
113.5
77.2 CDCl3
55.5
36.4
33.7
31.9
25.8
210 200 190 180 170 160 150 140 130 120 110 100 90 80 70 60 50 40 30 20 10 0 -10
f1 (ppm)

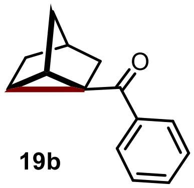

19b

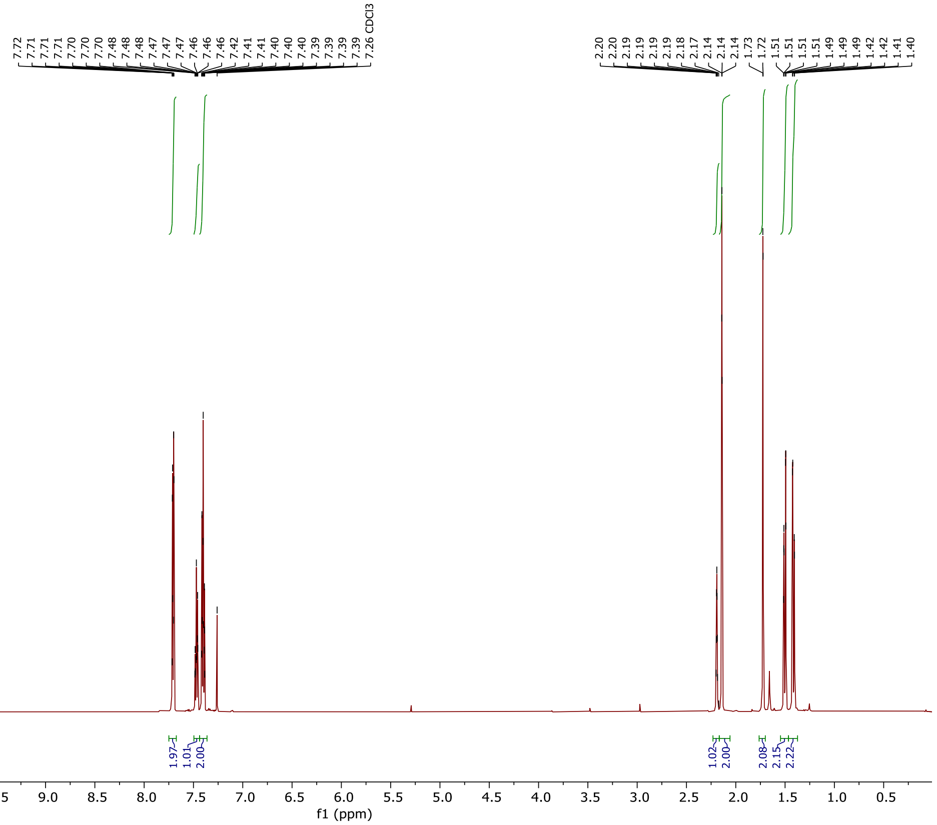
7.72
7.71
7.71
7.71
7.70
7.70
7.70
7.48
7.48
7.48
7.47
7.47
7.47
7.46
7.46
7.46
7.42
7.41
7.41
7.40
7.40
7.40
7.39
7.39
7.39
7.26 CDCl3
2.20
2.20
2.19
2.19
2.19
2.19
2.18
2.17
2.14
2.14
2.14
1.73
1.72
1.51
1.51
1.51
1.51
1.49
1.49
1.42
1.42
1.41
1.40
1.97
1.01
2.00
1.02
2.00
2.08
2.15
2.22
f1 (ppm)

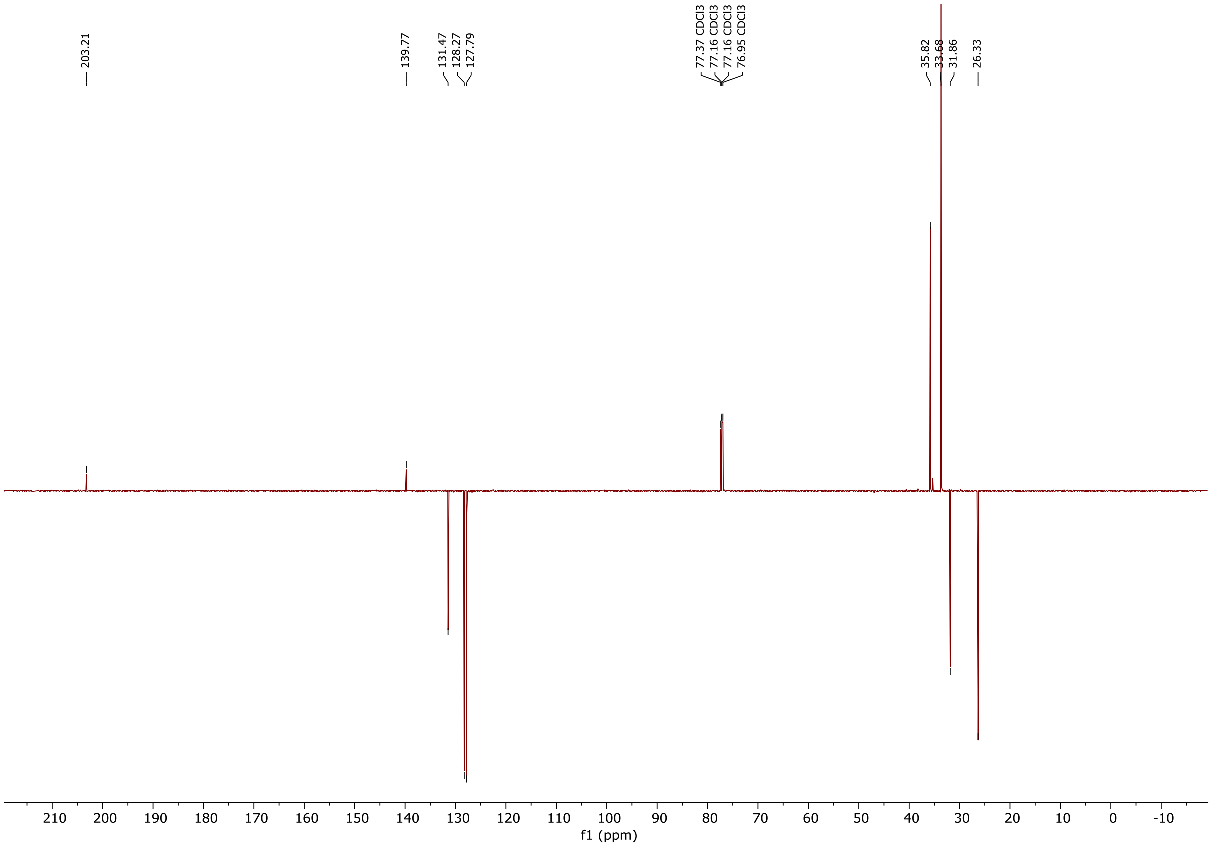
203.21
139.77
131.47
128.27
127.79
77.37 CDCl3
77.16 CDCl3
77.16 CDCl3
76.95 CDCl3
35.82
33.68
31.86
26.33
f1 (ppm)

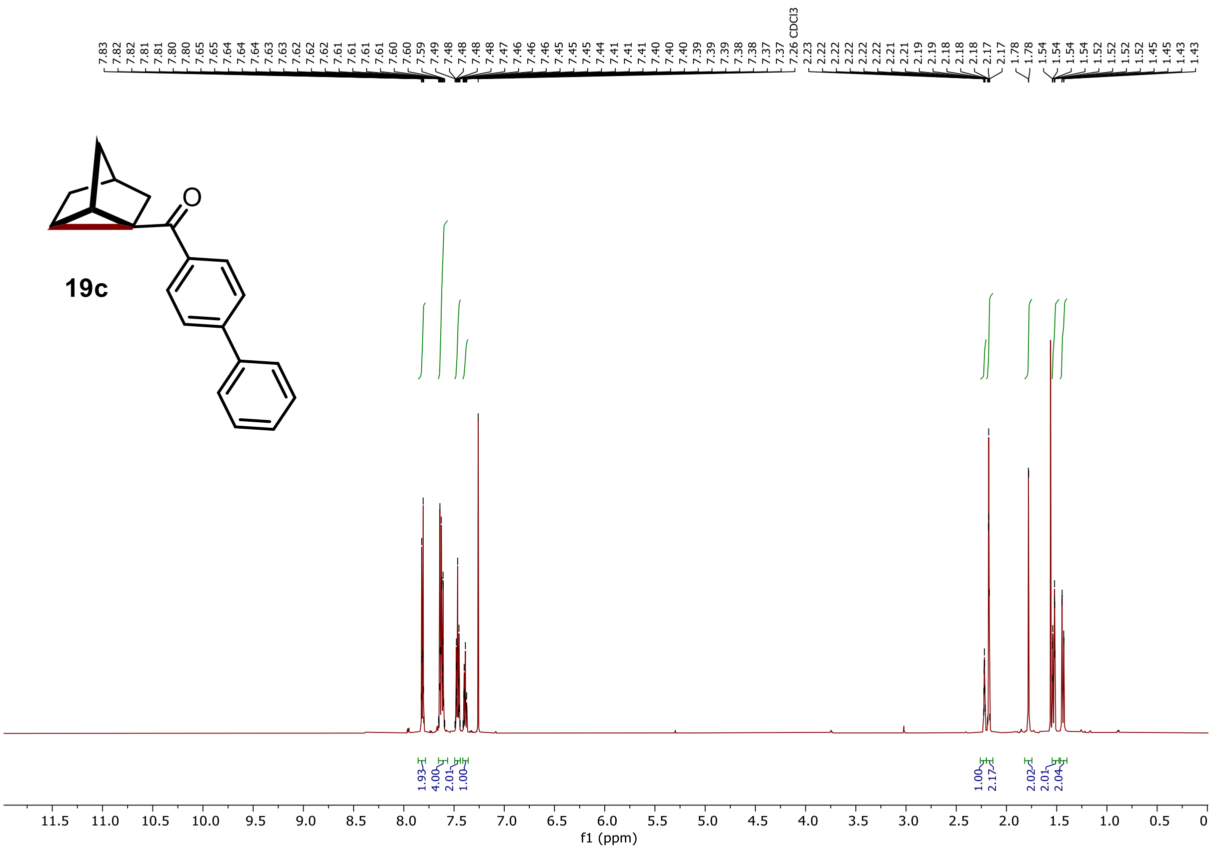

19c
7.83
7.82
7.82
7.81
7.81
7.80
7.65
7.64
7.64
7.63
7.63
7.62
7.62
7.61
7.61
7.61
7.60
7.59
7.49
7.48
7.48
7.48
7.48
7.47
7.46
7.46
7.45
7.45
7.44
7.41
7.41
7.41
7.40
7.40
7.40
7.39
7.39
7.39
7.38
7.38
7.37
7.37
7.26 CDCl3
2.23
2.22
2.22
2.22
2.22
2.22
2.21
2.21
2.19
2.19
2.19
2.18
2.18
2.17
2.17
1.78
1.78
1.54
1.54
1.54
1.54
1.52
1.52
1.52
1.52
1.45
1.43
1.43
1.43
1.93
4.00
2.01
1.00
1.00
2.17
2.02
2.01
2.04
11.5
11.0
10.5
10.0
9.5
9.0
8.5
8.0
7.5
7.0
6.5
6.0
5.5
5.0
4.5
4.0
3.5
3.0
2.5
2.0
1.5
1.0
0.5
0
f1 (ppm)

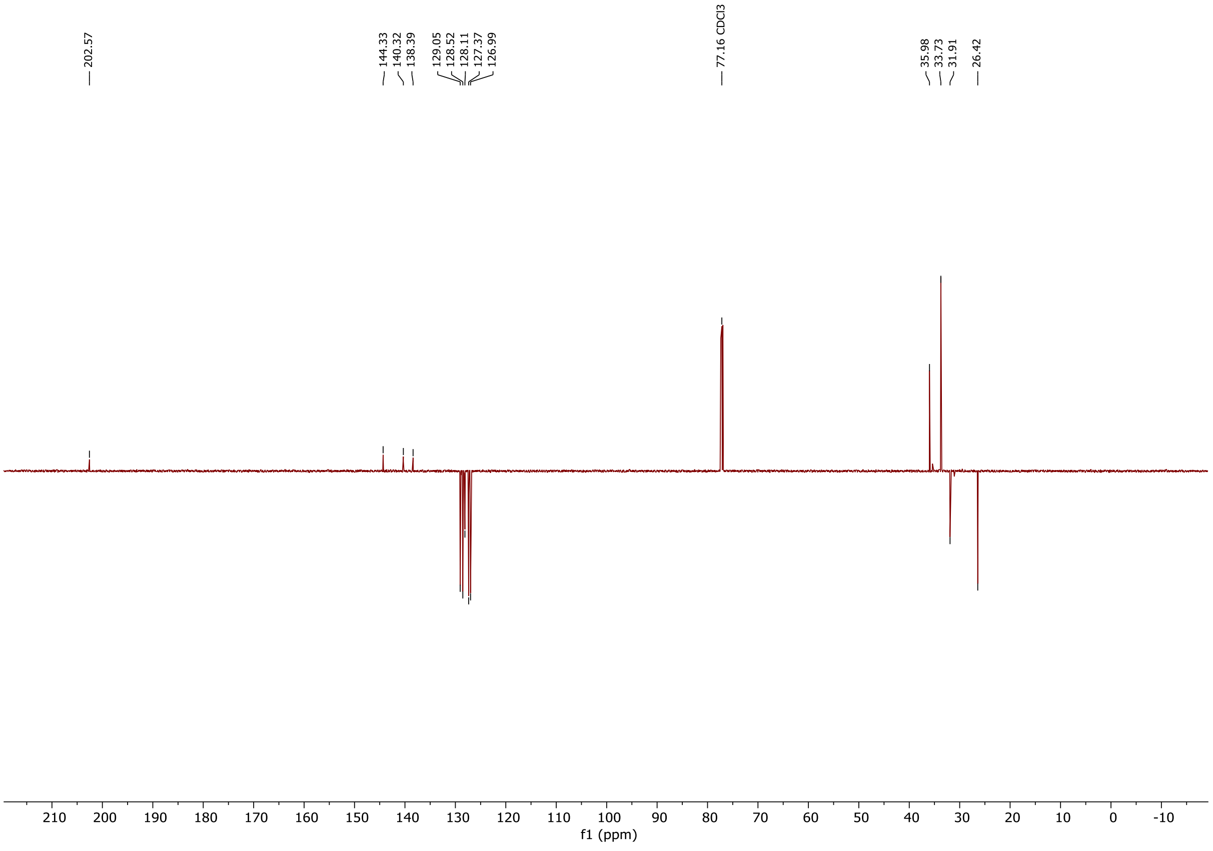
202.57
144.33
140.32
138.39
129.05
128.52
128.11
127.37
126.99
77.16 CDCl3
35.98
33.73
31.91
26.42
210
200
190
180
170
160
150
140
130
120
110
100
90
80
70
60
50
40
30
20
10
0
-10
f1 (ppm)

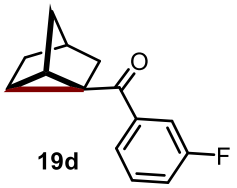

19d

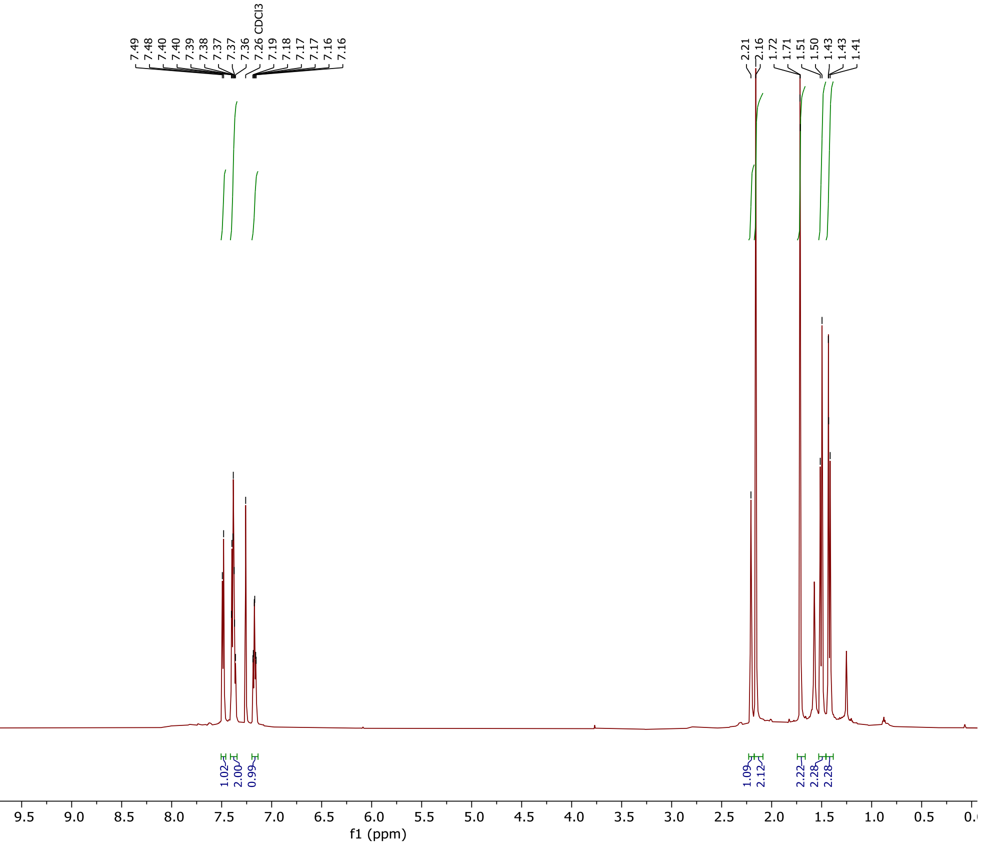
7.49
7.48
7.40
7.40
7.39
7.38
7.37
7.37
7.36
7.26 CDCl3
7.19
7.18
7.17
7.17
7.16
7.16
2.21
2.16
1.72
1.71
1.51
1.50
1.43
1.43
1.41
1.02
2.00
0.99
1.09
2.12
2.22
2.28
2.28
f1 (ppm)

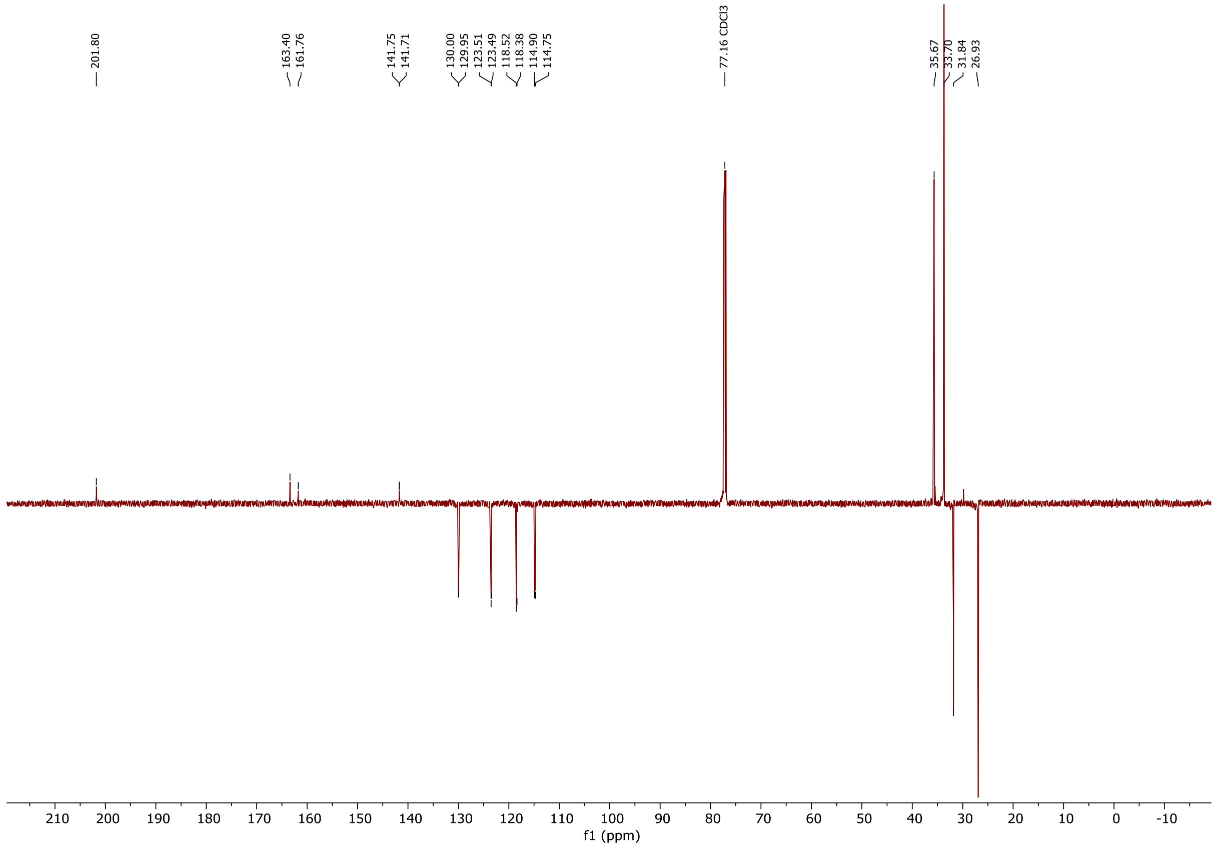
201.80
163.40
161.76
141.75
141.71
130.00
129.95
123.51
123.49
118.52
118.38
114.90
114.75
77.16 CDCl3
35.67
33.70
31.84
26.93
f1 (ppm)

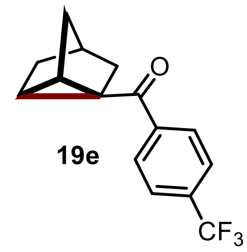

19e

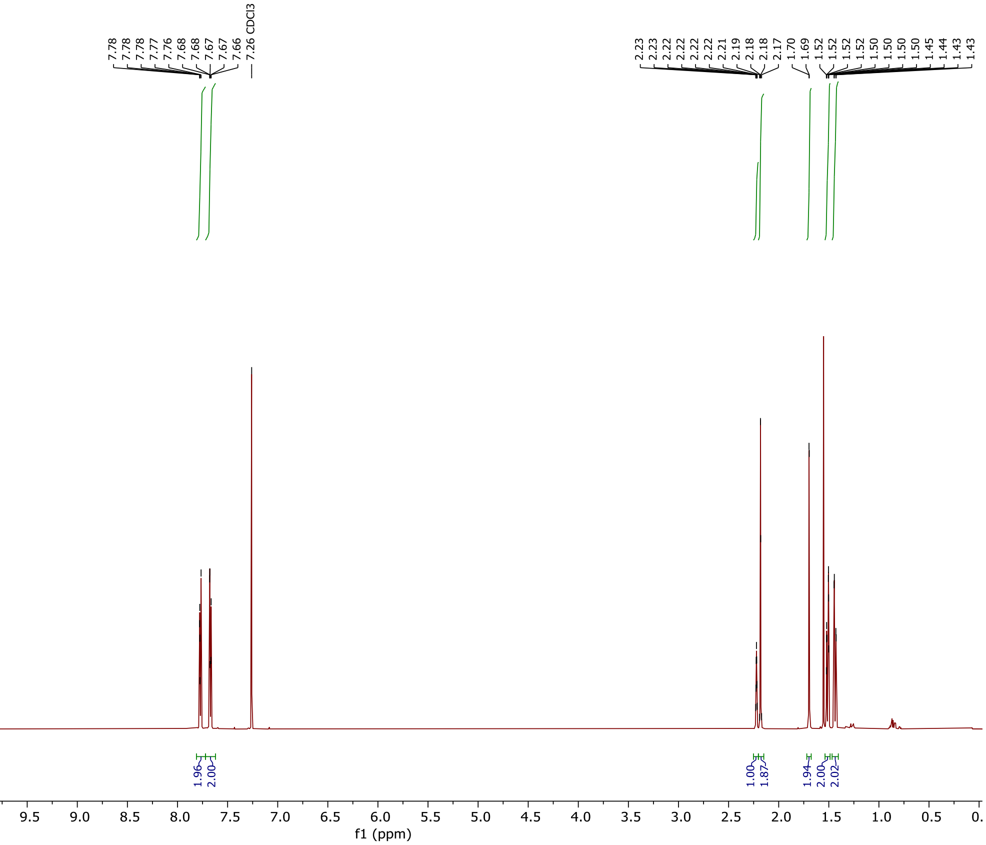

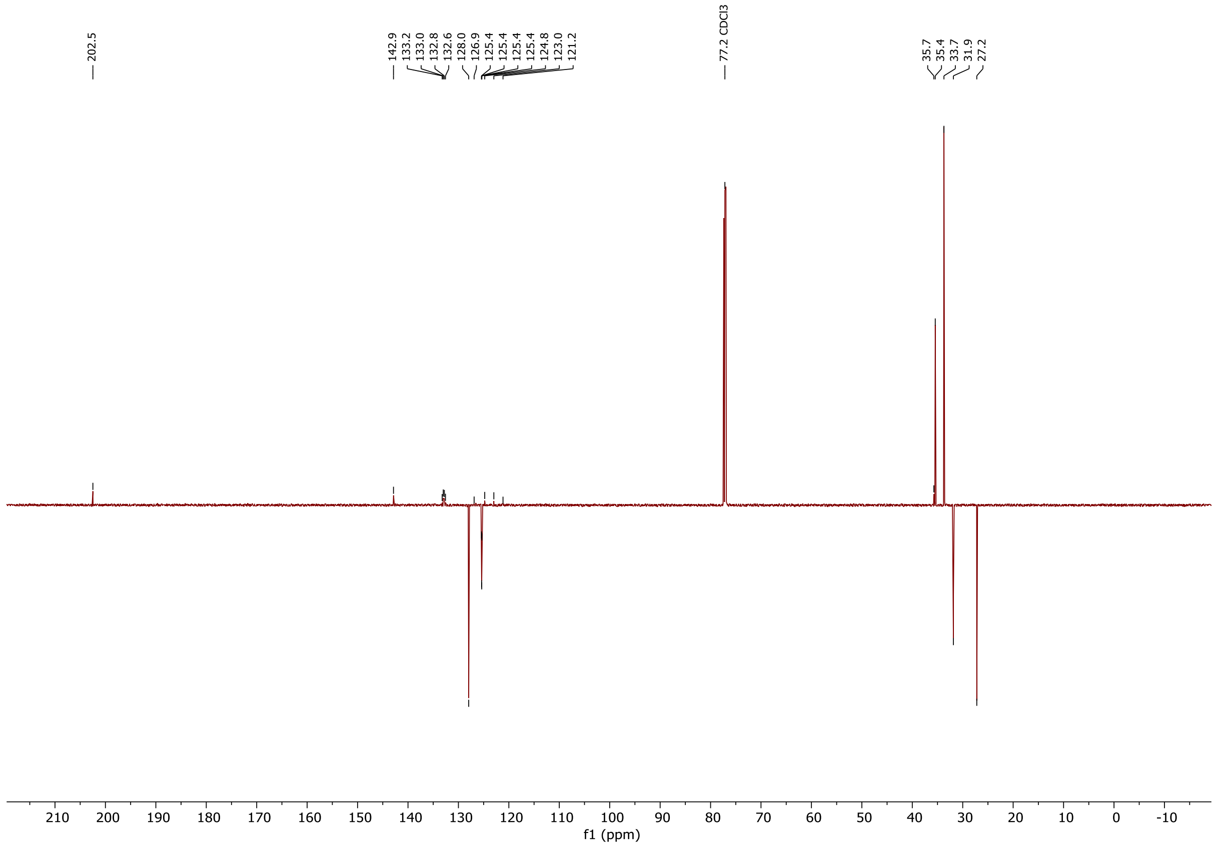

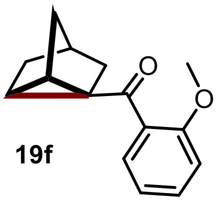

19f

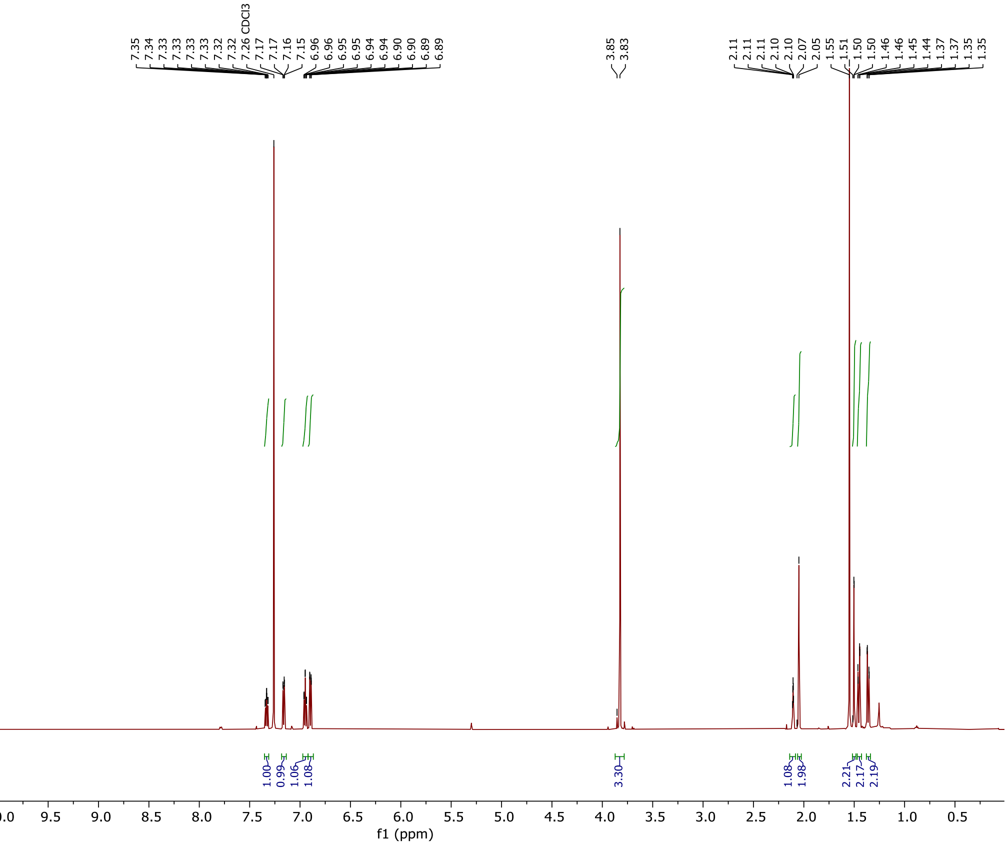
7.35
7.34
7.33
7.33
7.33
7.33
7.32
7.32
7.26 CDCl3
7.17
7.17
7.16
7.15
6.96
6.96
6.95
6.95
6.94
6.94
6.90
6.90
6.89
6.89
3.85
3.83
2.11
2.11
2.11
2.10
2.10
2.07
2.05
1.55
1.51
1.50
1.50
1.46
1.46
1.45
1.44
1.37
1.37
1.35
1.35
1.00
0.99
1.06
1.08
3.30
1.08
1.98
2.21
2.17
2.19
f1 (ppm)

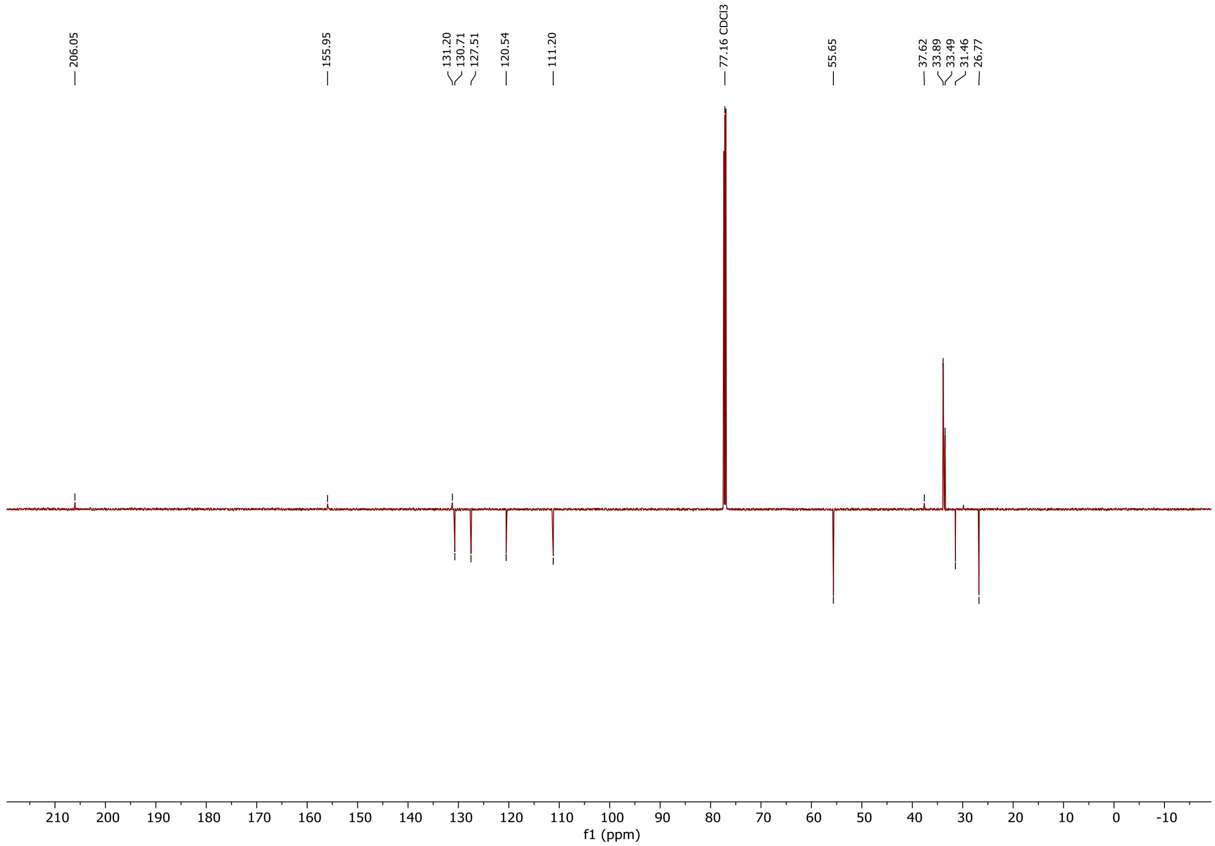
206.05
155.95
131.20
130.71
127.51
120.54
111.20
77.16 CDCl3
55.65
37.62
33.89
33.49
31.46
26.77
f1 (ppm)

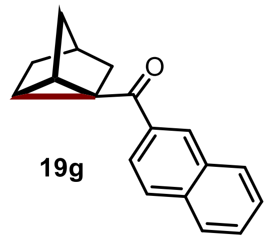
O

19g

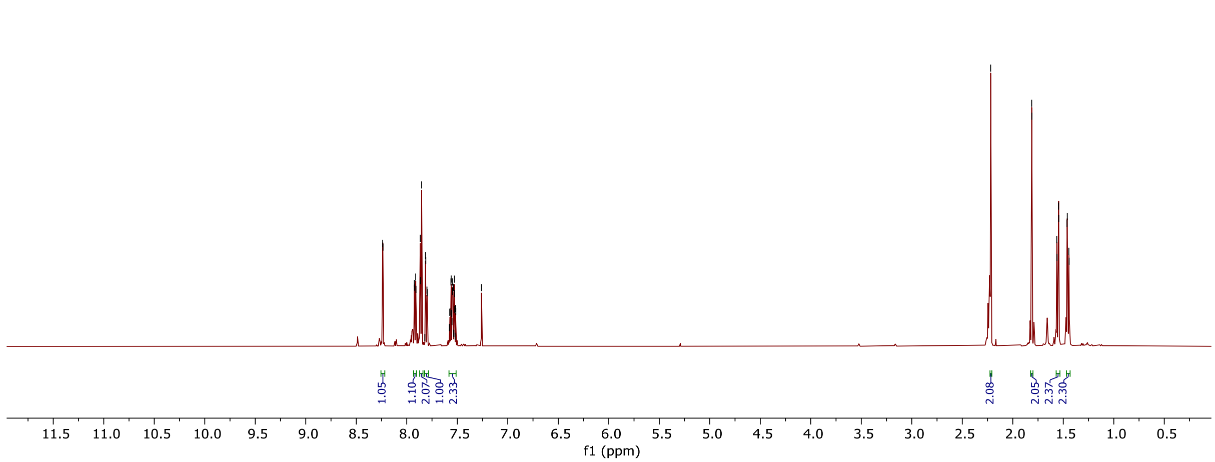
7.3 CDCl3
1.05
1.10
2.07
1.00
2.33
2.08
2.05
2.37
2.30
f1 (ppm)

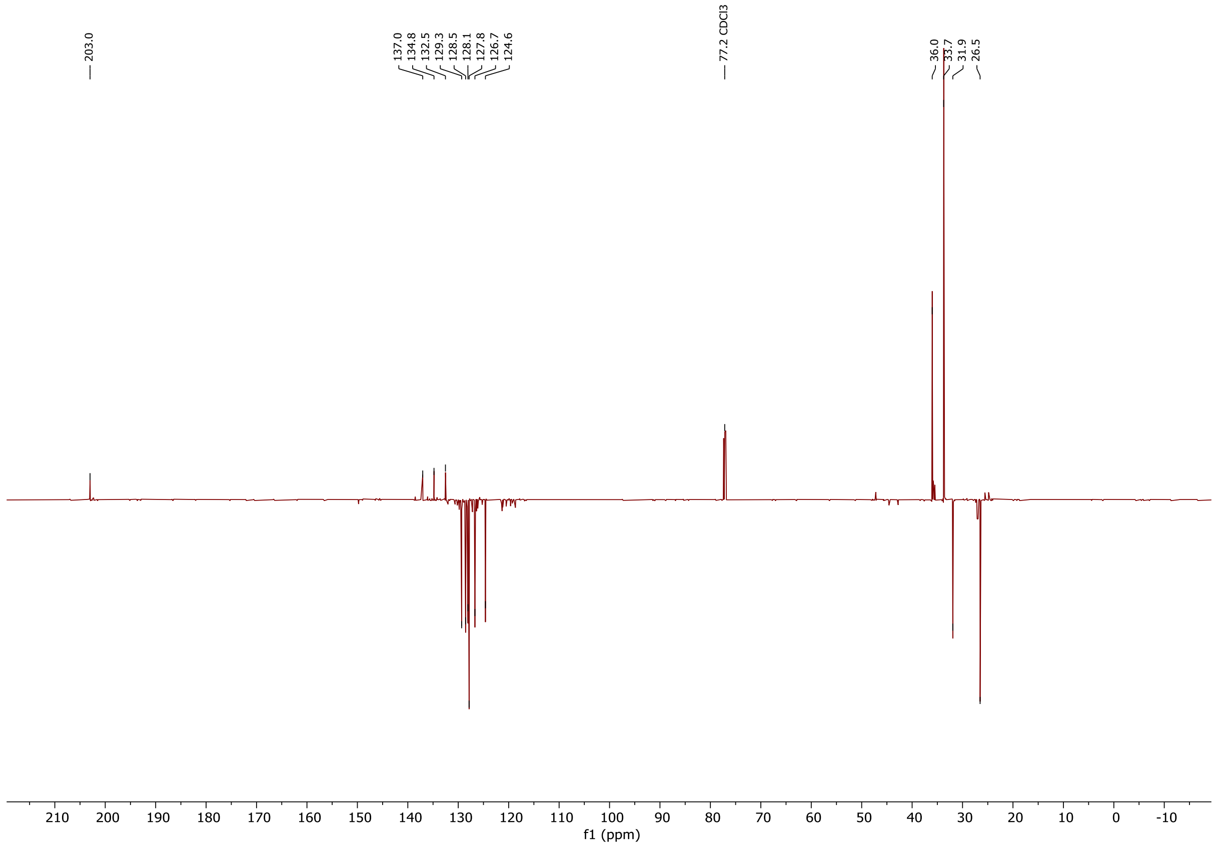
203.0
137.0
134.8
132.5
129.3
128.5
128.1
127.8
126.7
124.6
77.2 CDCl3
36.0
33.7
31.9
26.5
f1 (ppm)

19h

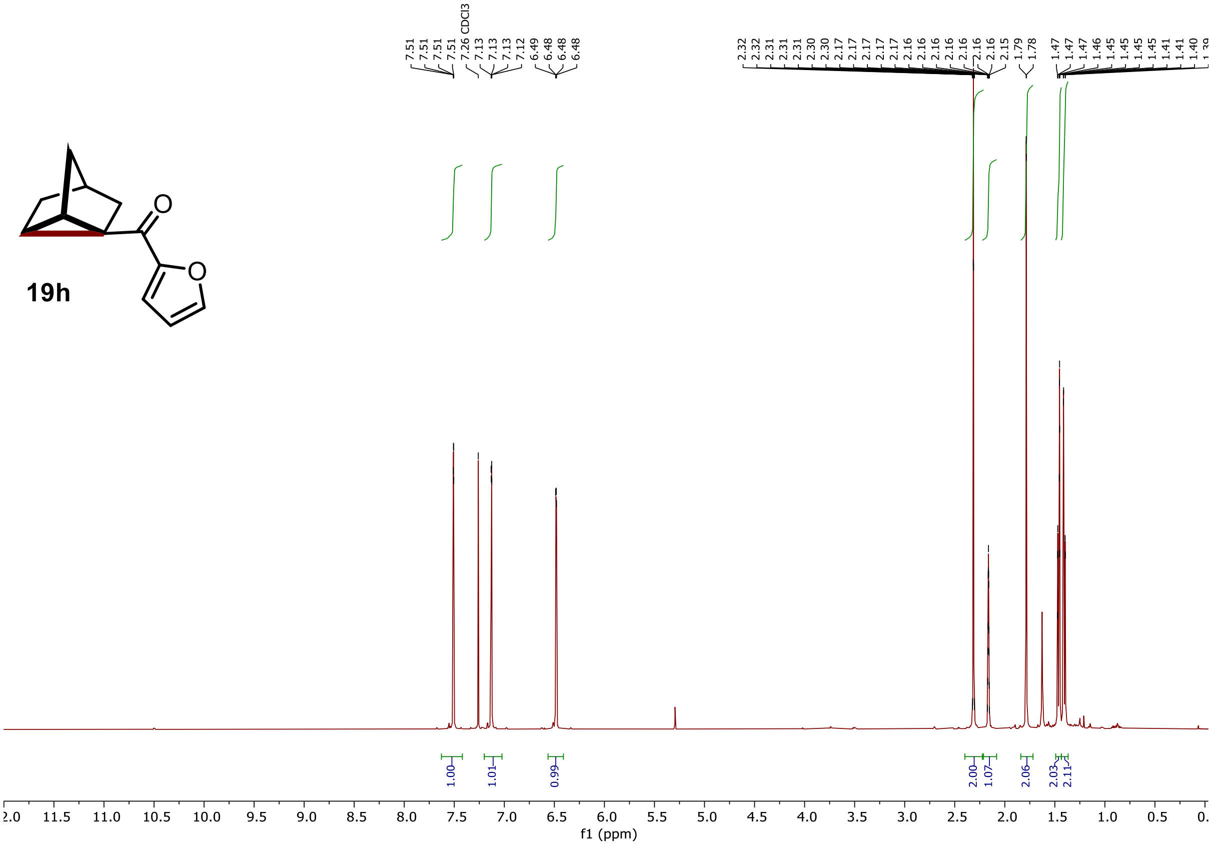
7.51
7.51
7.51
7.51
7.26 CDCl3
7.13
7.13
7.13
7.13
7.12
6.49
6.48
6.48
6.48
2.32
2.32
2.31
2.31
2.31
2.30
2.30
2.17
2.17
2.17
2.17
2.17
2.16
2.16
2.16
2.16
2.16
2.16
2.15
1.79
1.78
1.47
1.47
1.47
1.46
1.45
1.45
1.45
1.45
1.41
1.41
1.40
1.00
1.01
0.99
2.00
1.07
2.06
2.03
2.11
f1 (ppm)

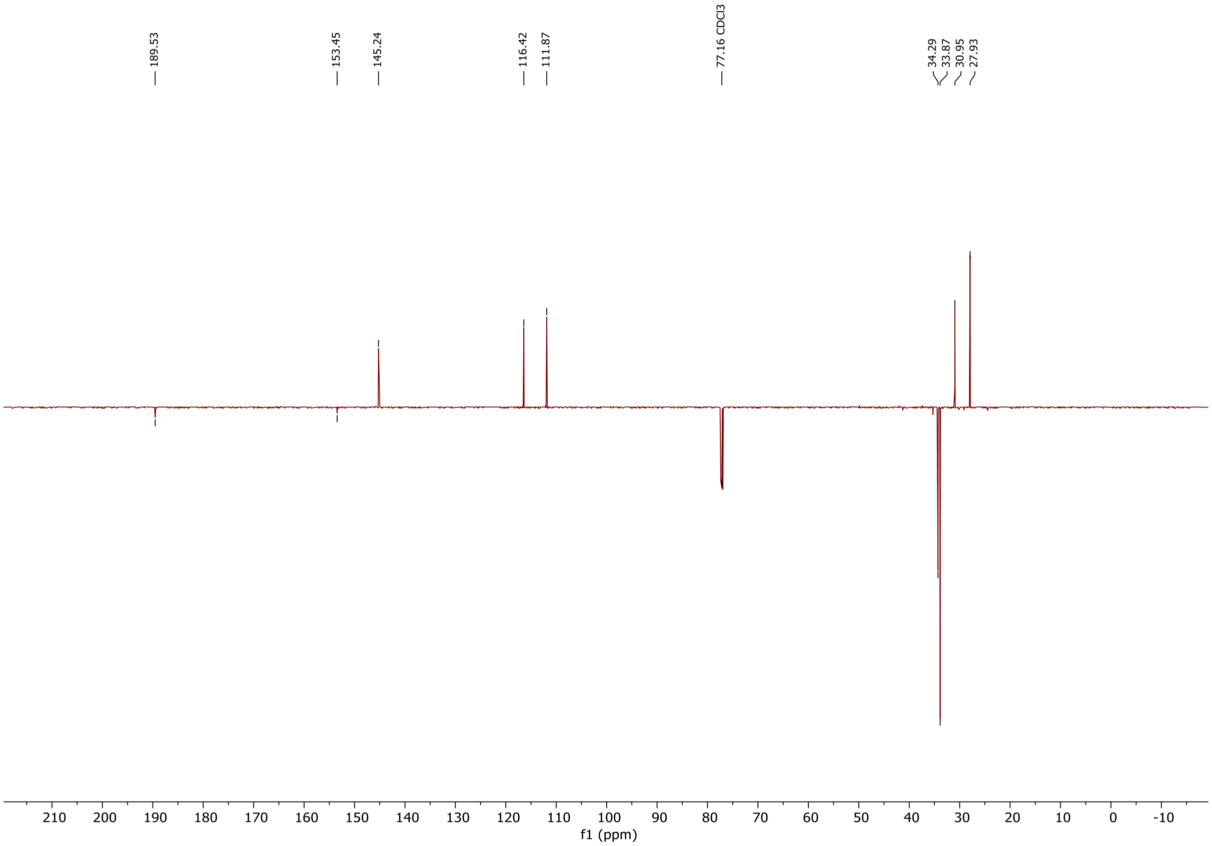
189.53
153.45
145.24
116.42
111.87
77.16 CDCl3
34.29
33.87
30.95
27.93
f1 (ppm)

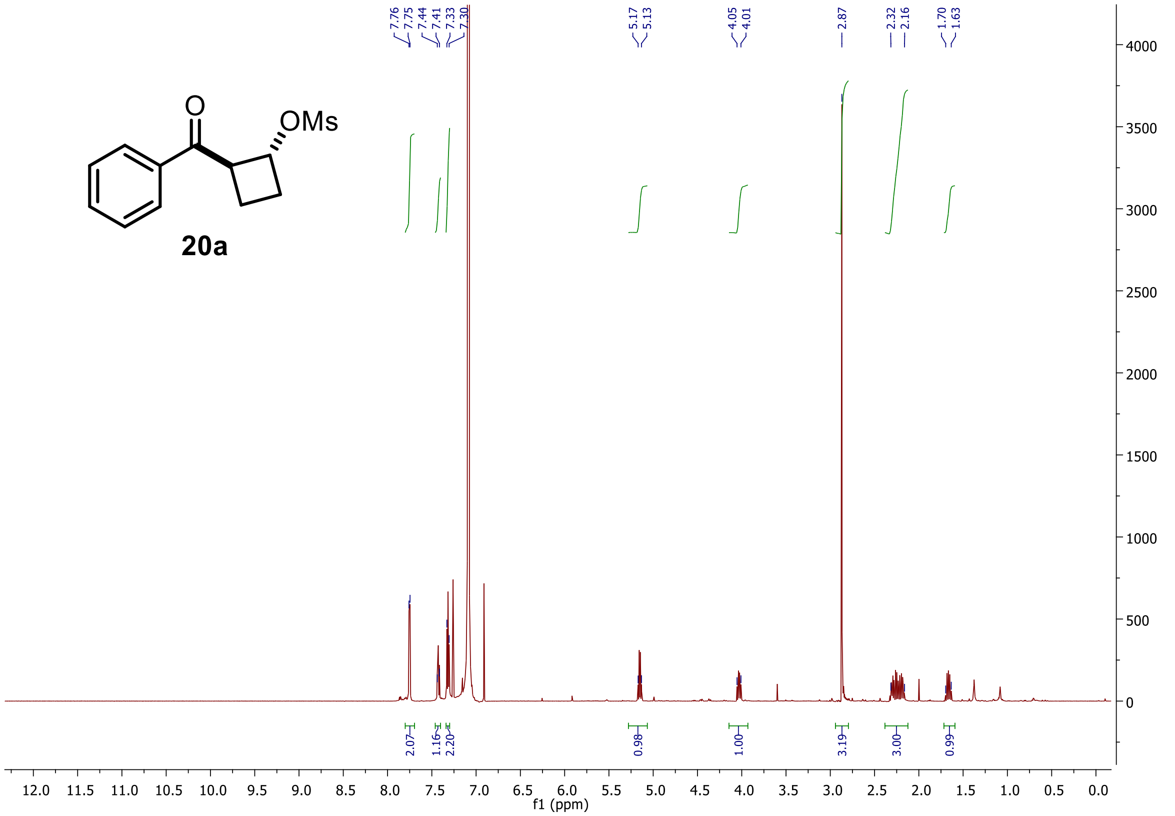
O
OMs
20a
7.76
7.75
7.44
7.41
7.33
7.30
5.17
5.13
4.05
4.01
2.87
2.32
2.16
1.70
1.63
2.07
1.16
2.20
0.98
1.00
3.19
3.00
0.99
f1 (ppm)

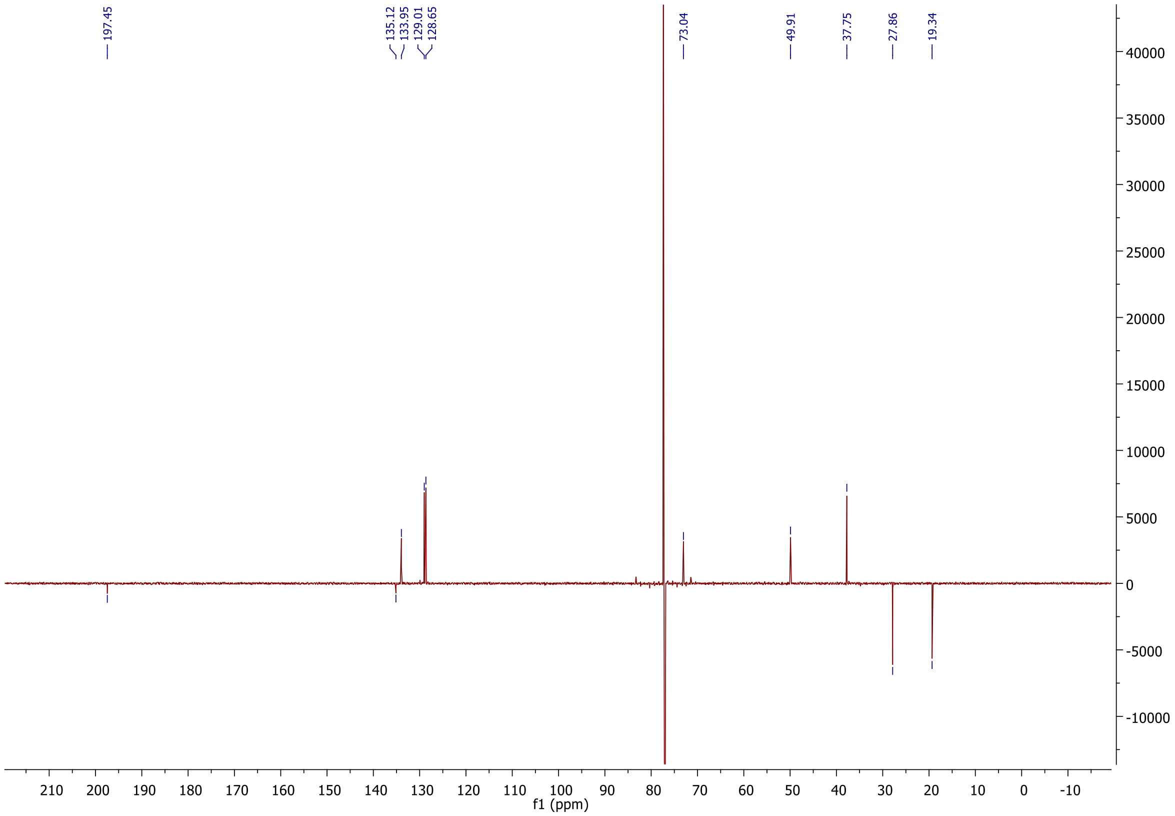
197.45
135.12
133.95
129.01
128.65
73.04
49.91
37.75
27.86
19.34
f1 (ppm)

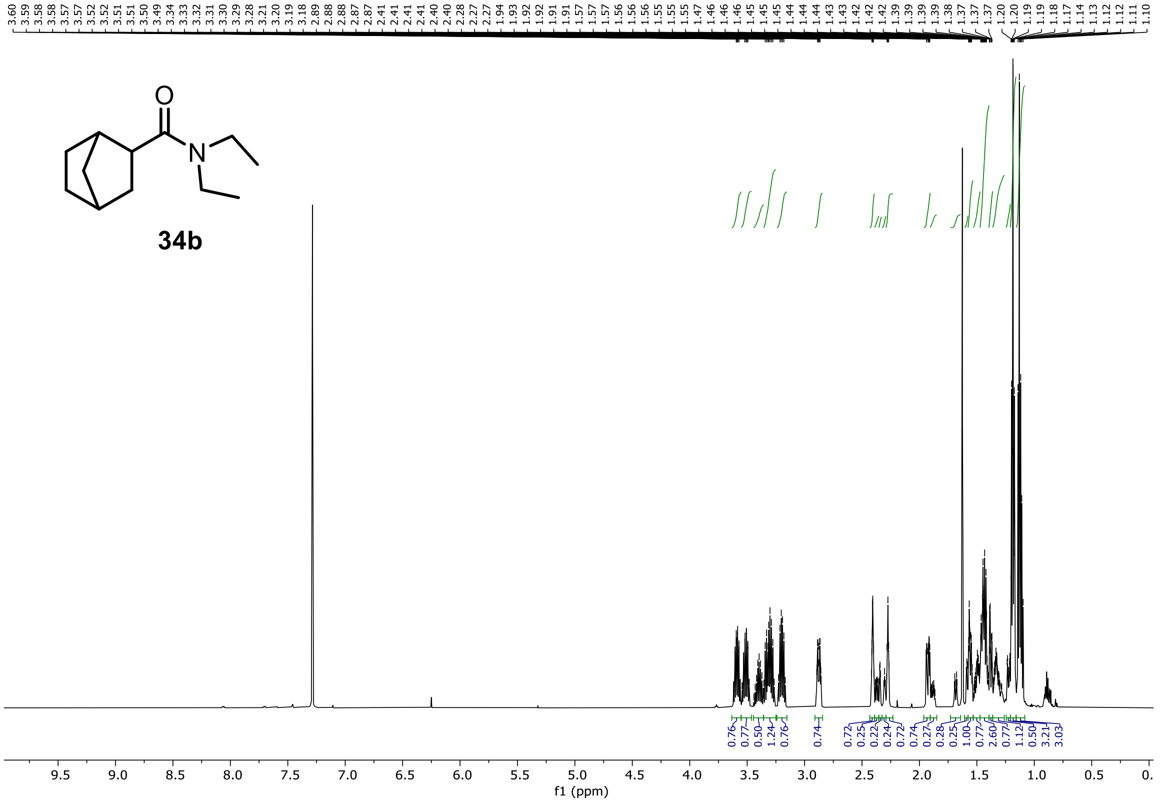

34b

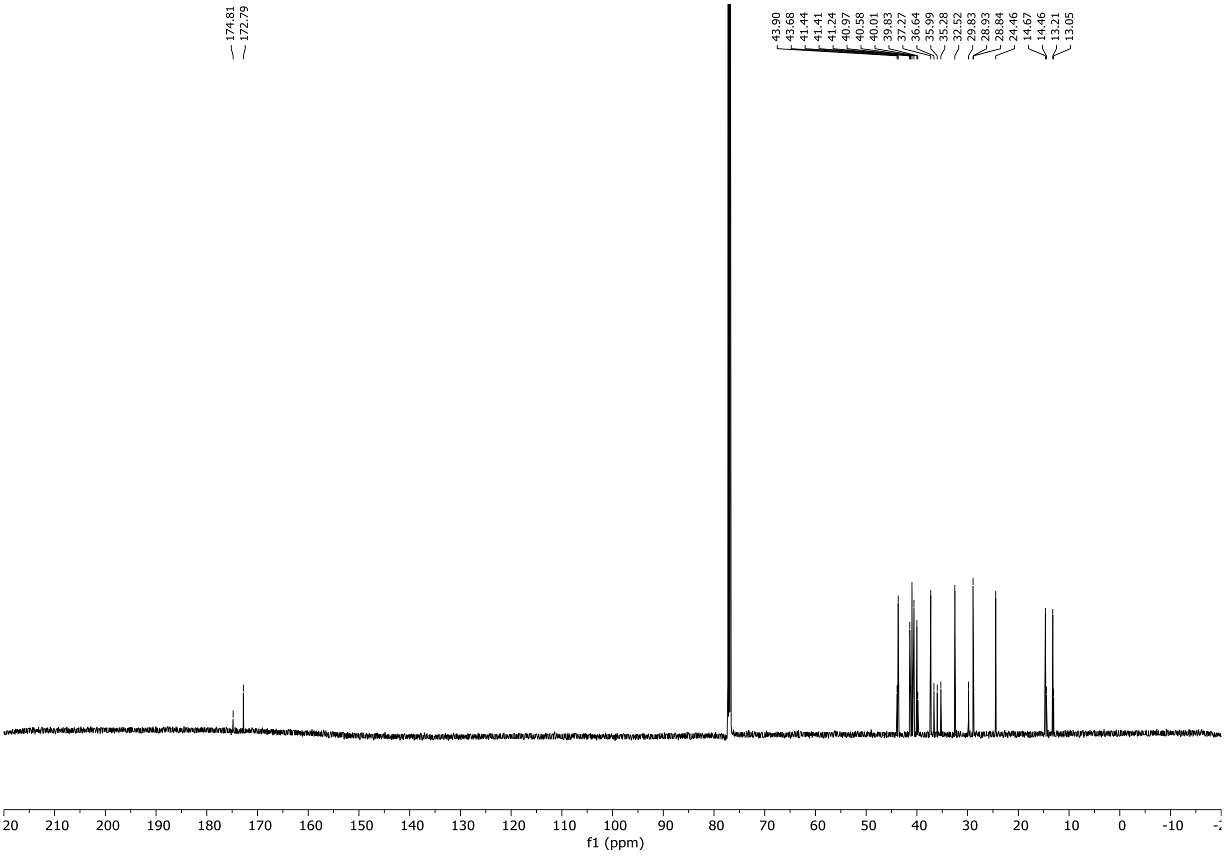

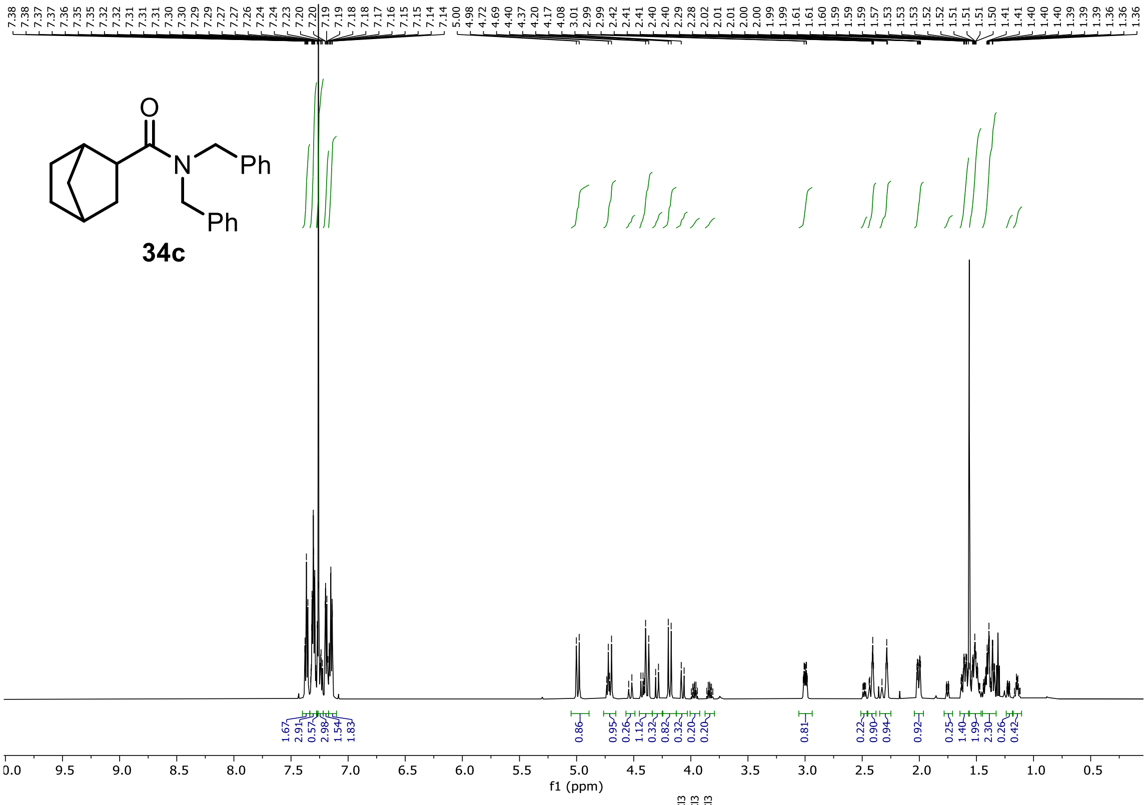
Ph
Ph
O
N
34c
f1 (ppm)

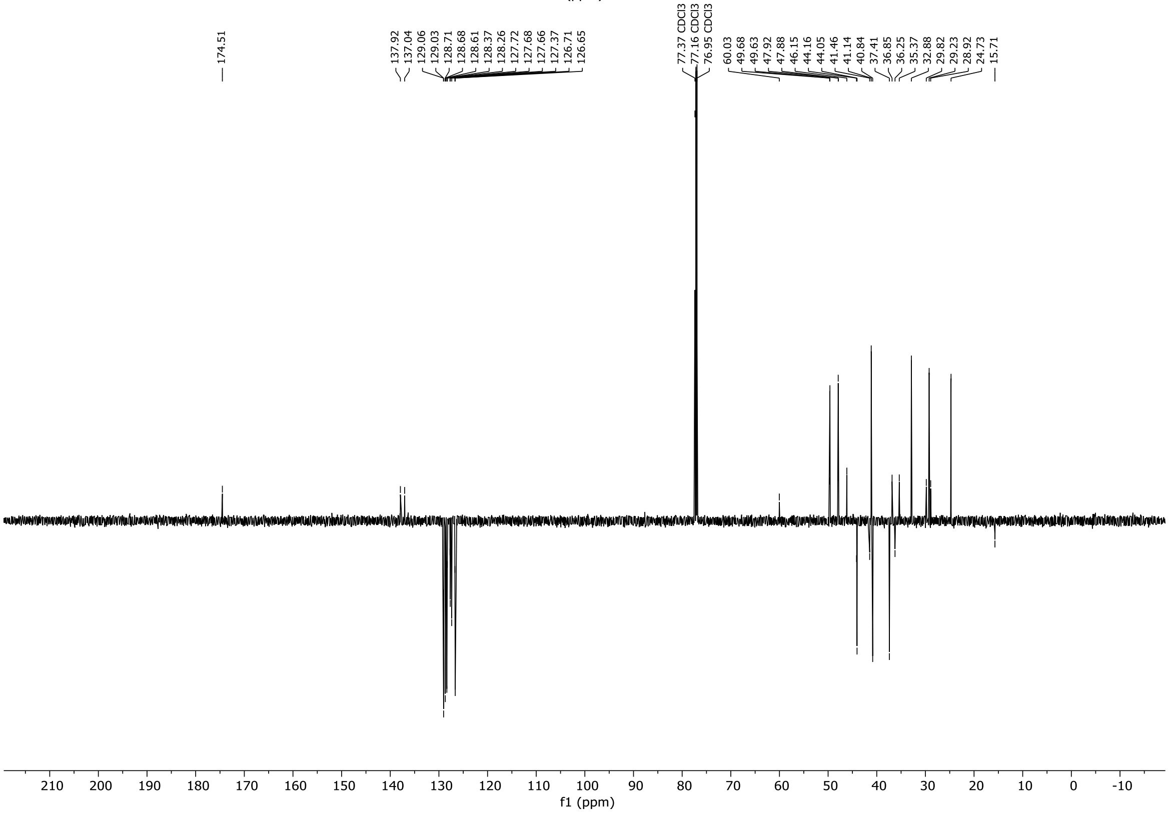
f1 (ppm)

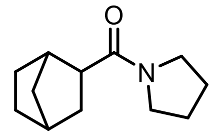

34d

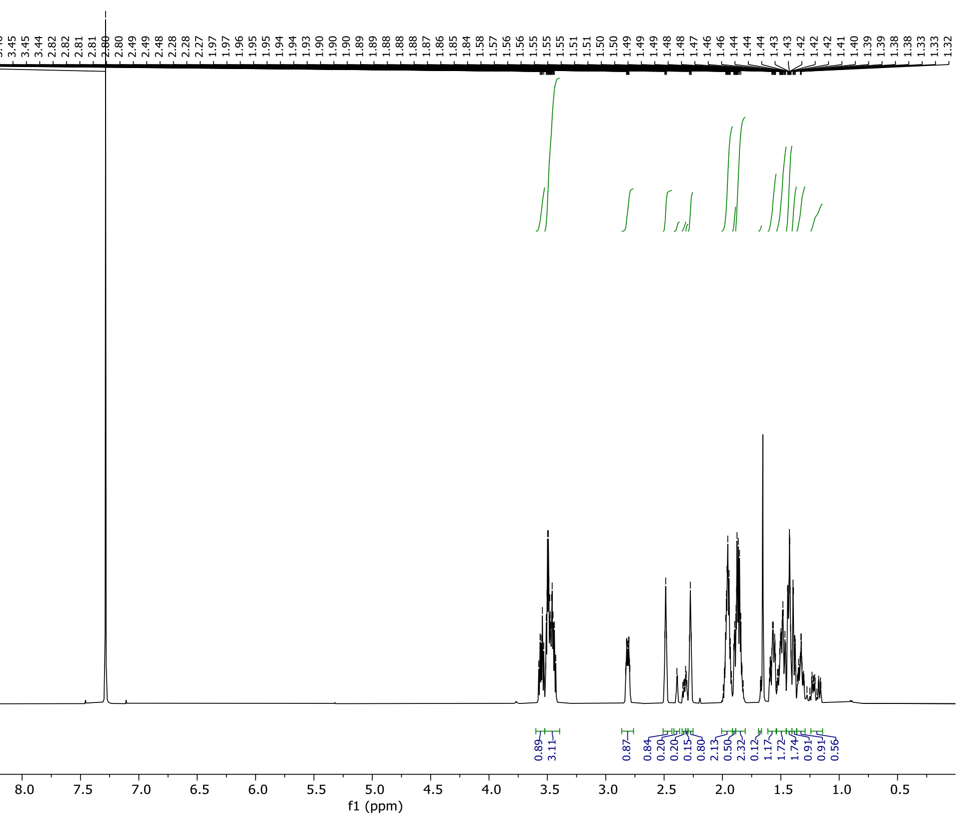
f1 (ppm)

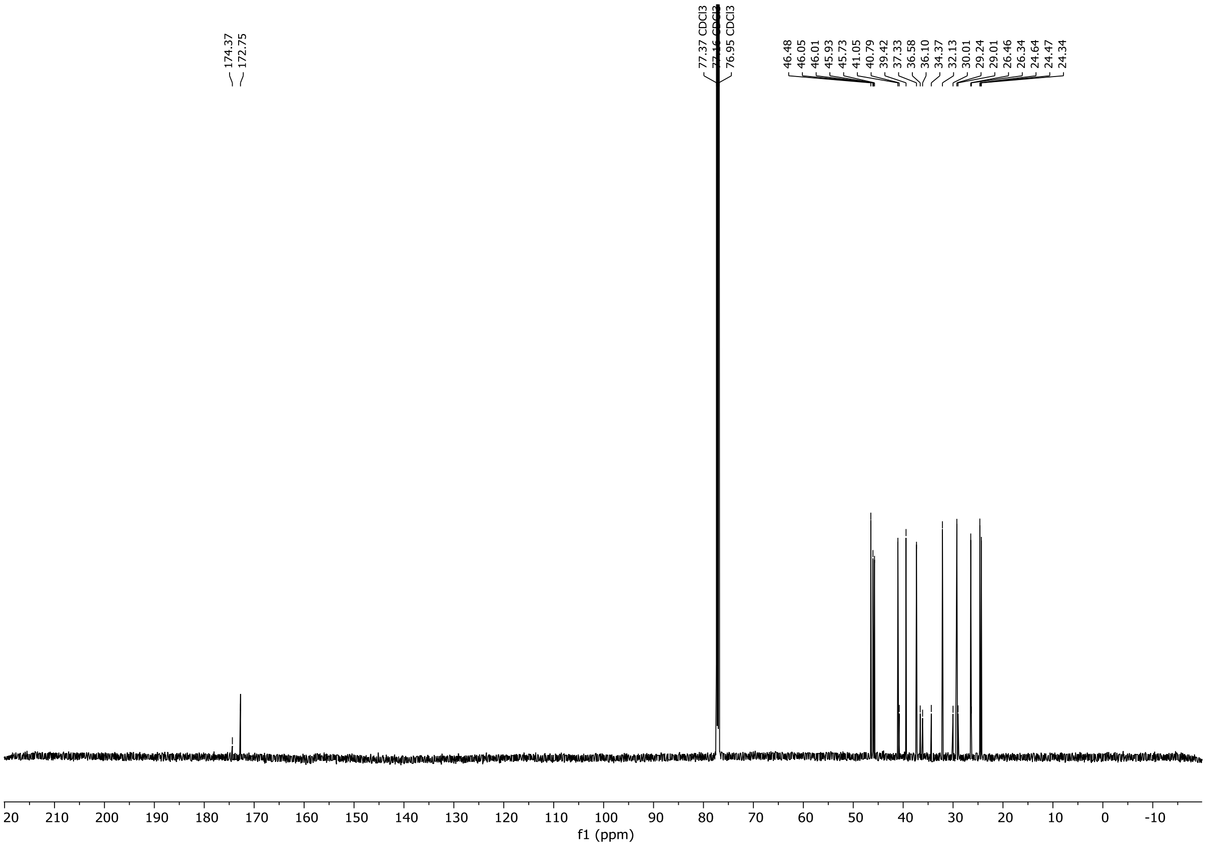
174.37
172.75
77.37 CDCl3
76.95 CDCl3
46.48
46.05
46.01
45.93
45.73
41.05
40.79
39.42
37.33
36.58
36.10
34.37
32.13
30.01
29.24
29.01
26.46
26.34
24.64
24.47
24.34
f1 (ppm)

35a

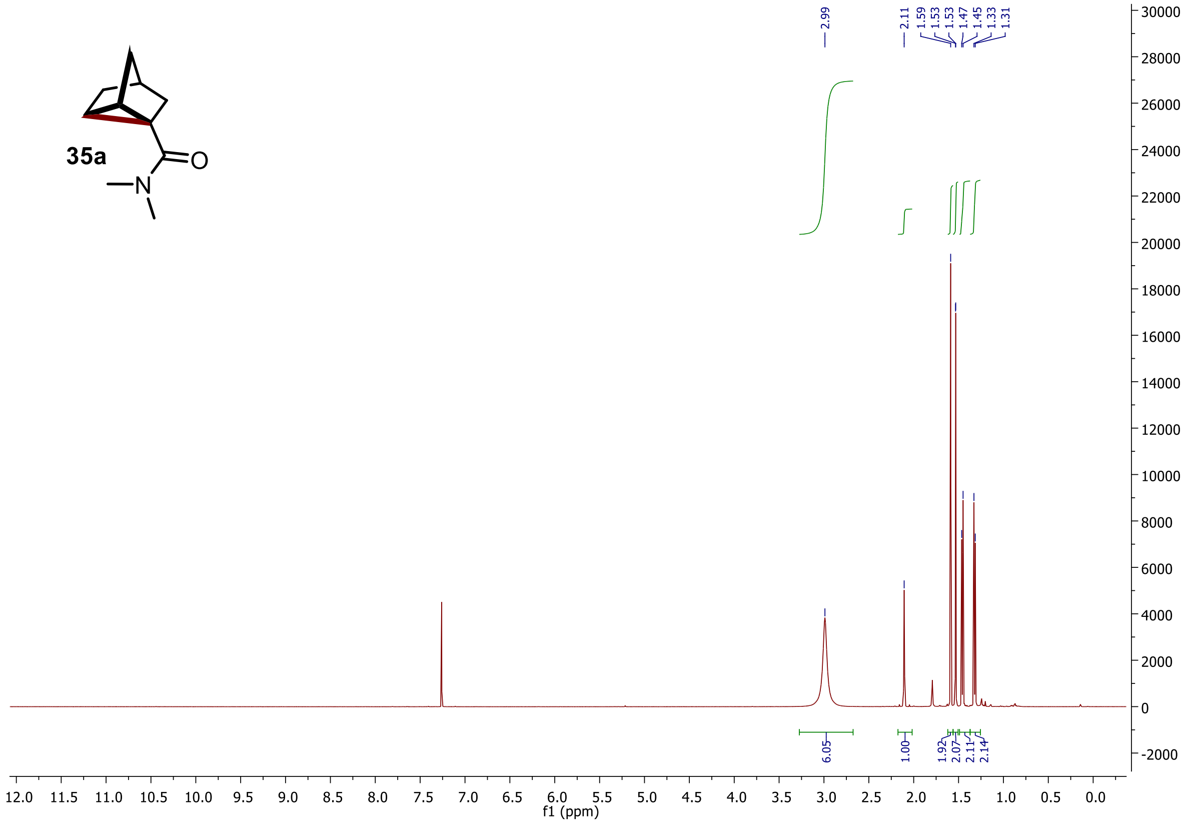
2.99
2.11
1.59
1.53
1.53
1.47
1.45
1.33
1.31
6.05
1.00
1.92
2.07
2.11
2.14
f1 (ppm)

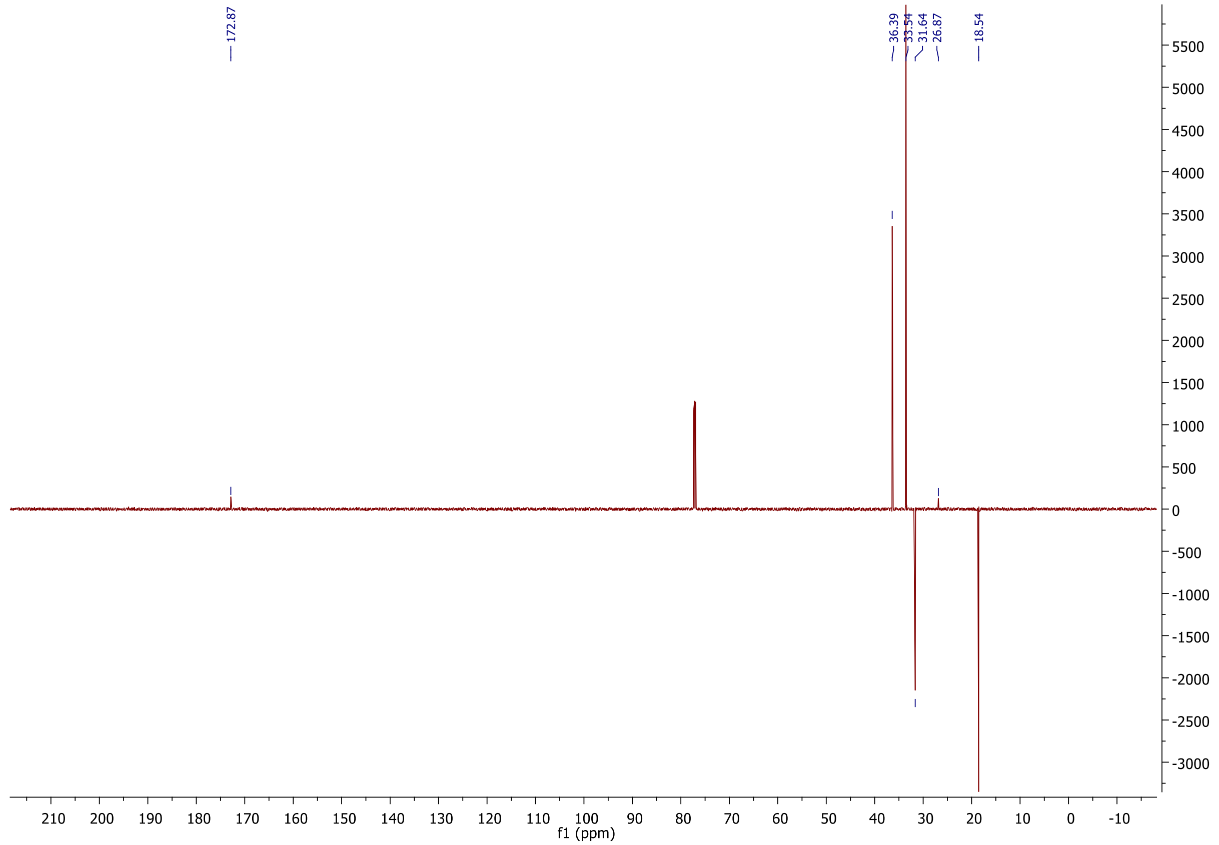
172.87
36.39
33.54
31.64
26.87
18.54
f1 (ppm)

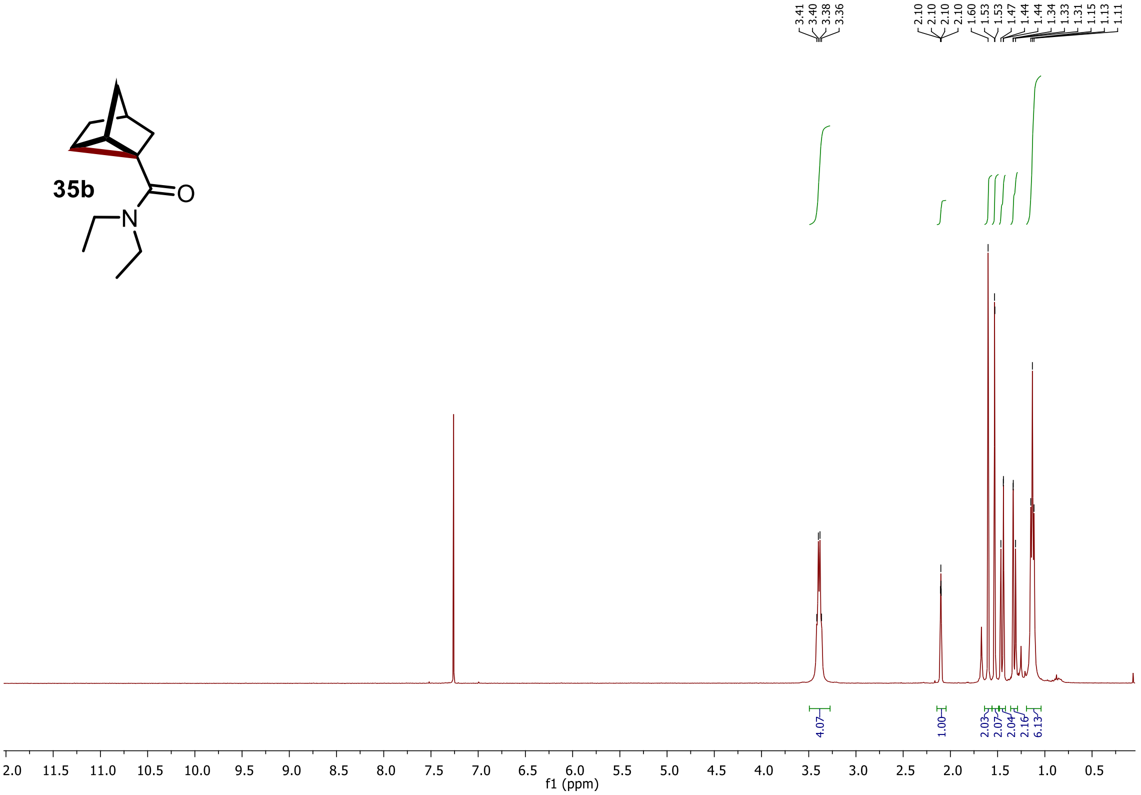
35b
3.41
3.40
3.38
3.36
2.10
2.10
2.10
2.10
1.60
1.53
1.53
1.47
1.44
1.34
1.33
1.31
1.15
1.13
1.11
4.07
1.00
2.03
2.07
2.04
2.16
6.13
f1 (ppm)

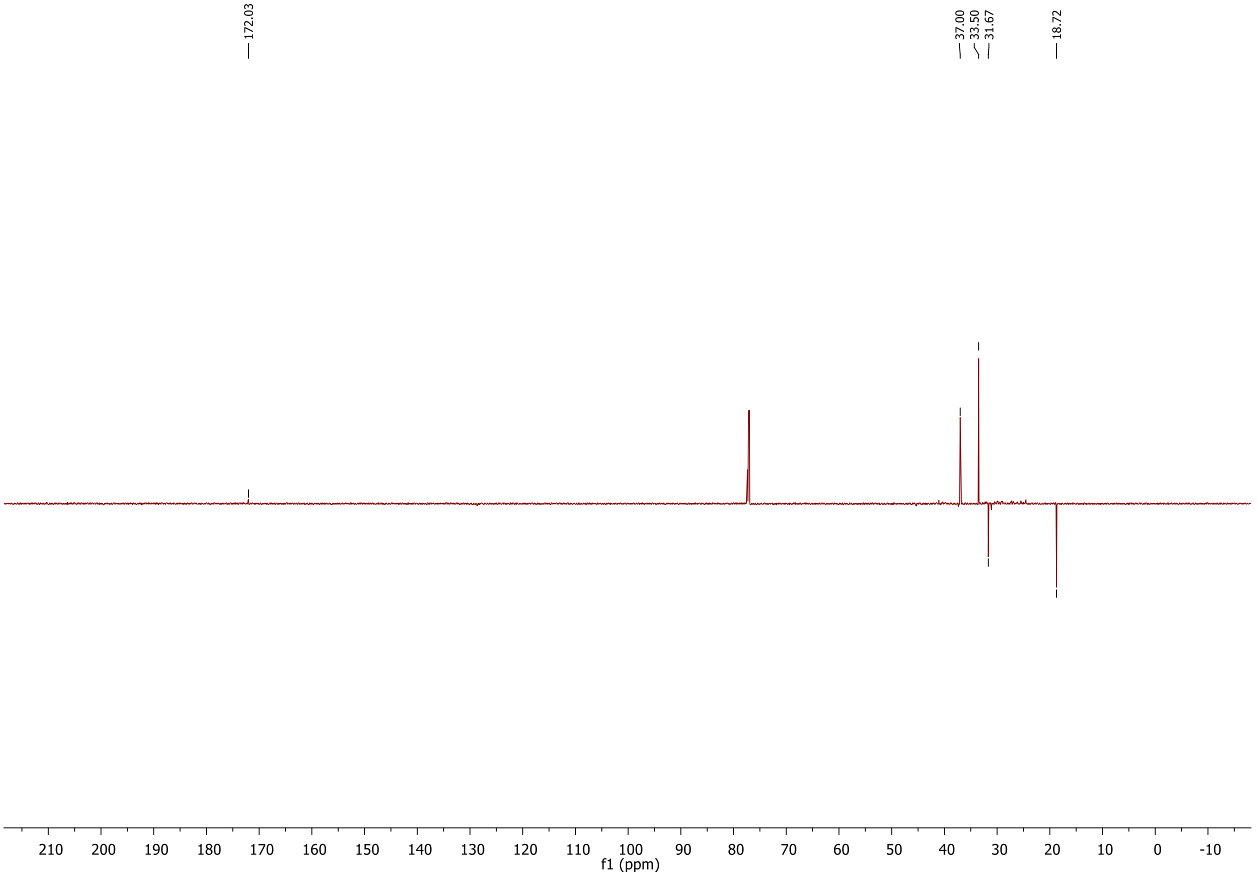
172.03
37.00
33.50
31.67
18.72
f1 (ppm)

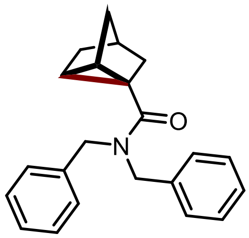
O
N

35c

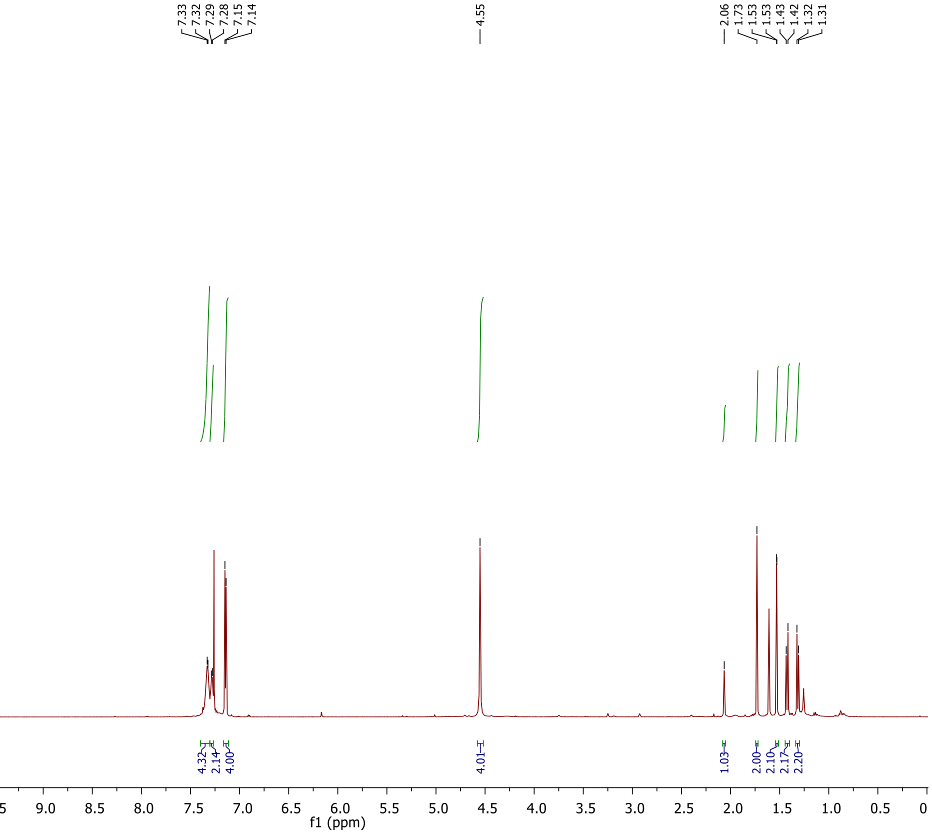
7.33
7.32
7.29
7.28
7.15
7.14
4.55
2.06
1.73
1.53
1.53
1.43
1.42
1.32
1.31
4.32
2.14
4.00
4.01
1.03
2.00
2.10
2.17
2.20
f1 (ppm)

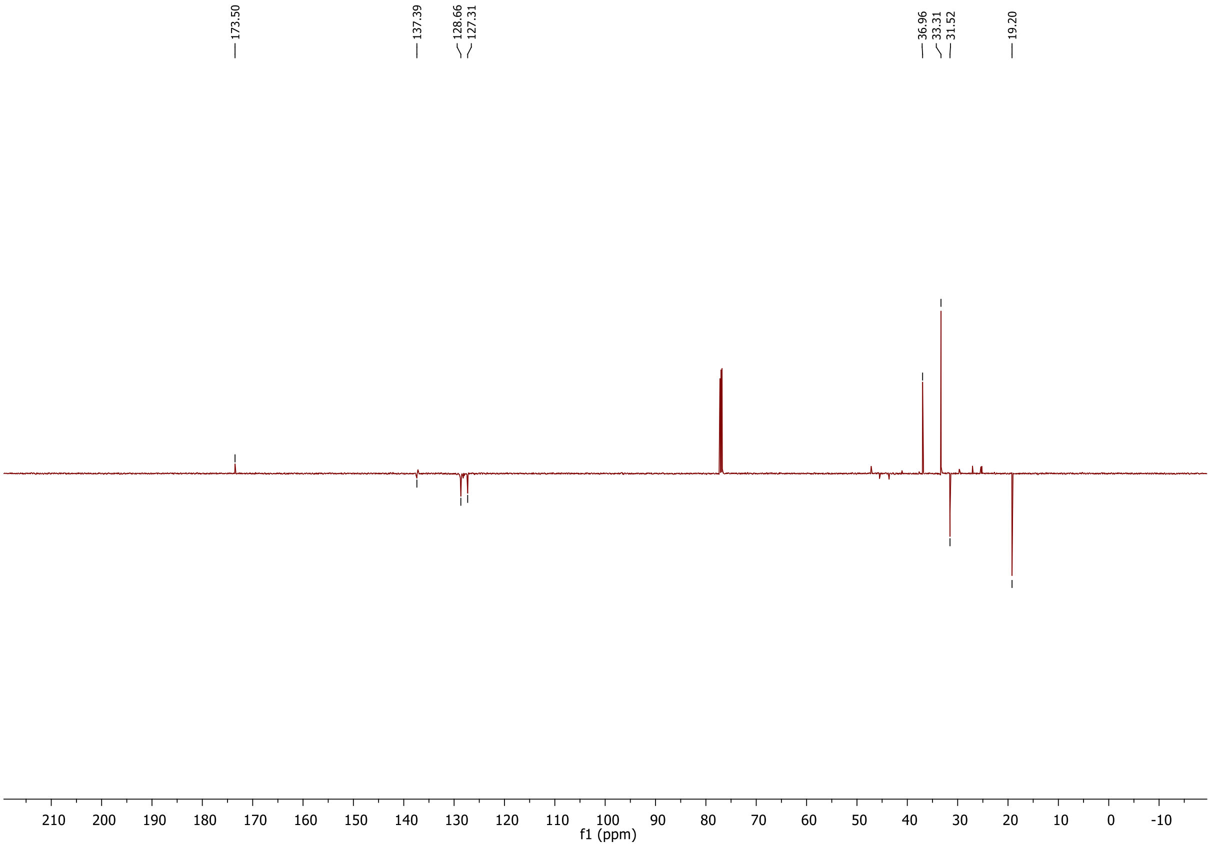
173.50
137.39
128.66
127.31
36.96
33.31
31.52
19.20
f1 (ppm)

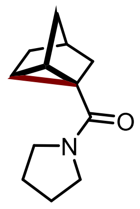

35d

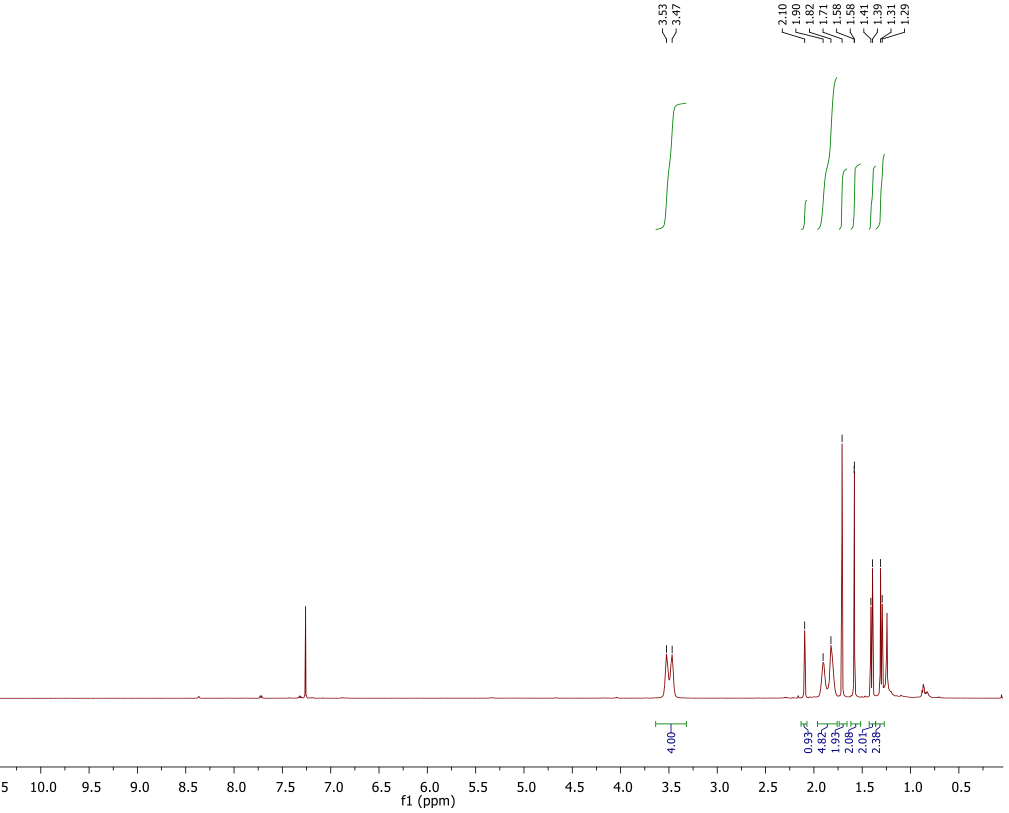

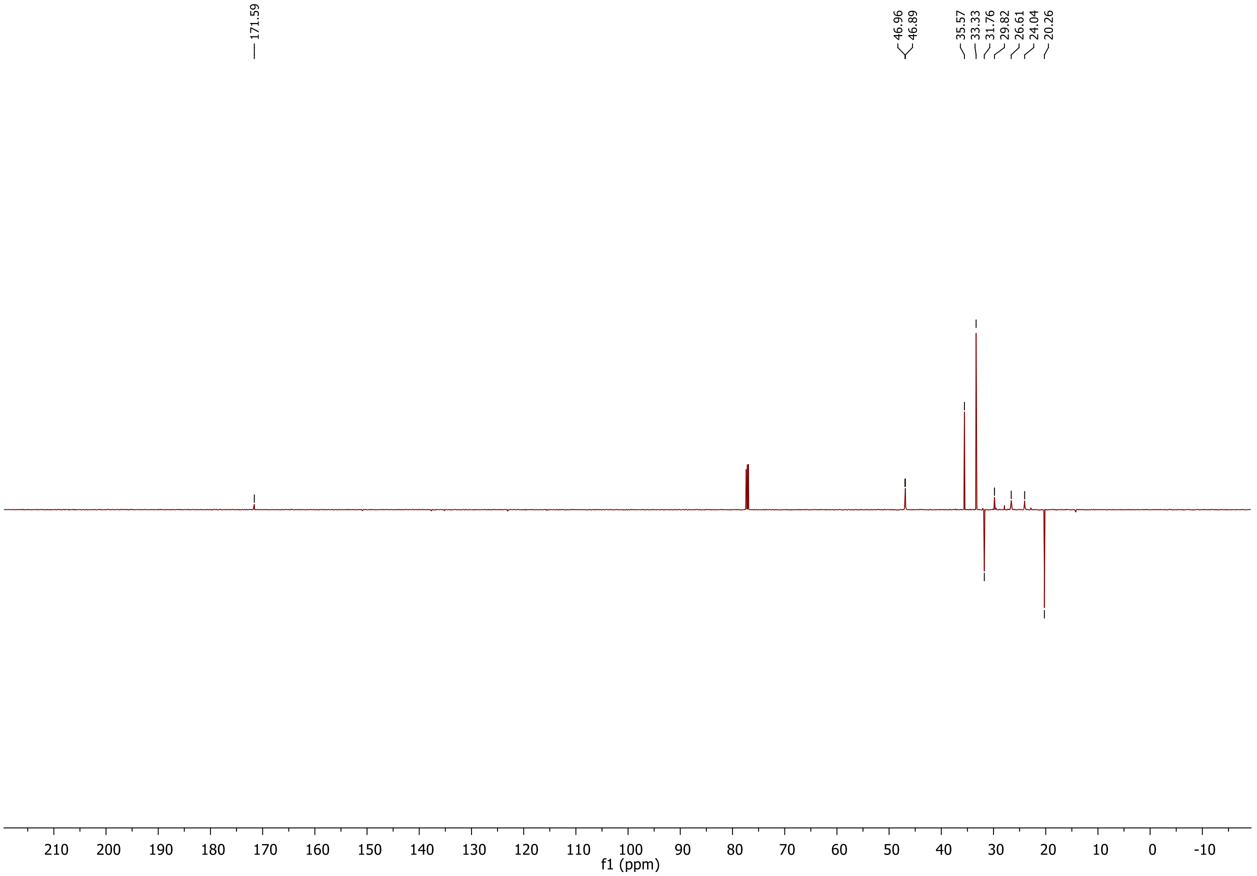

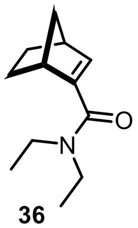

36

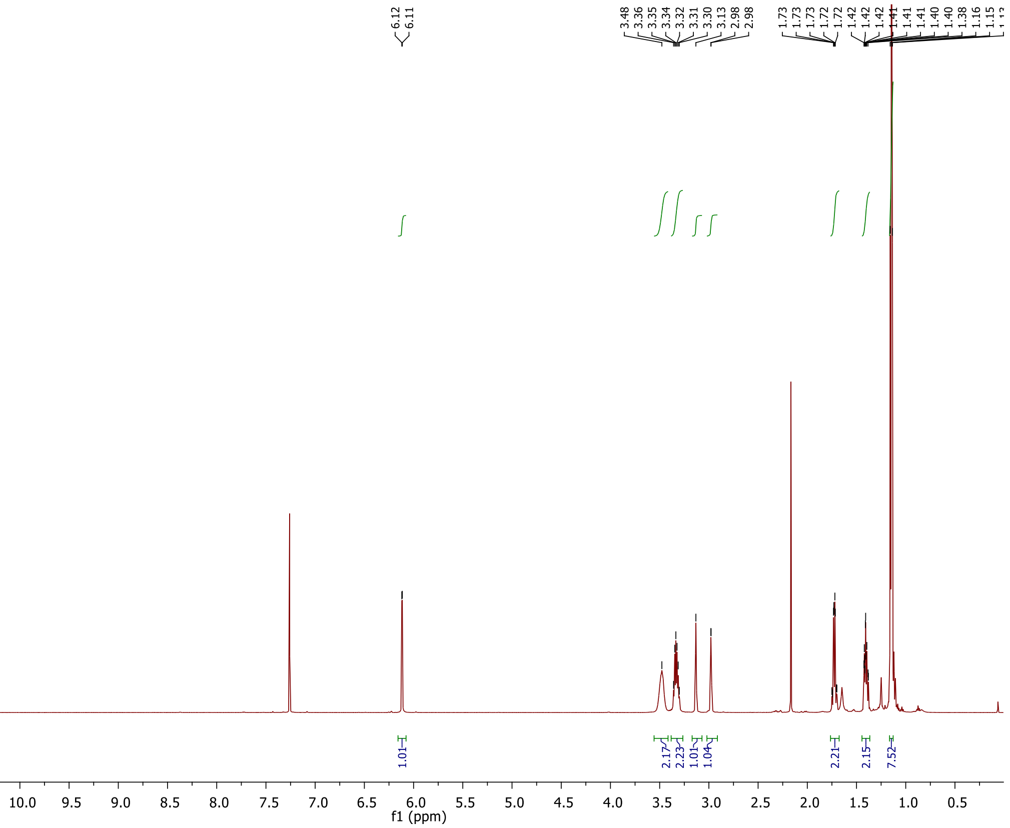

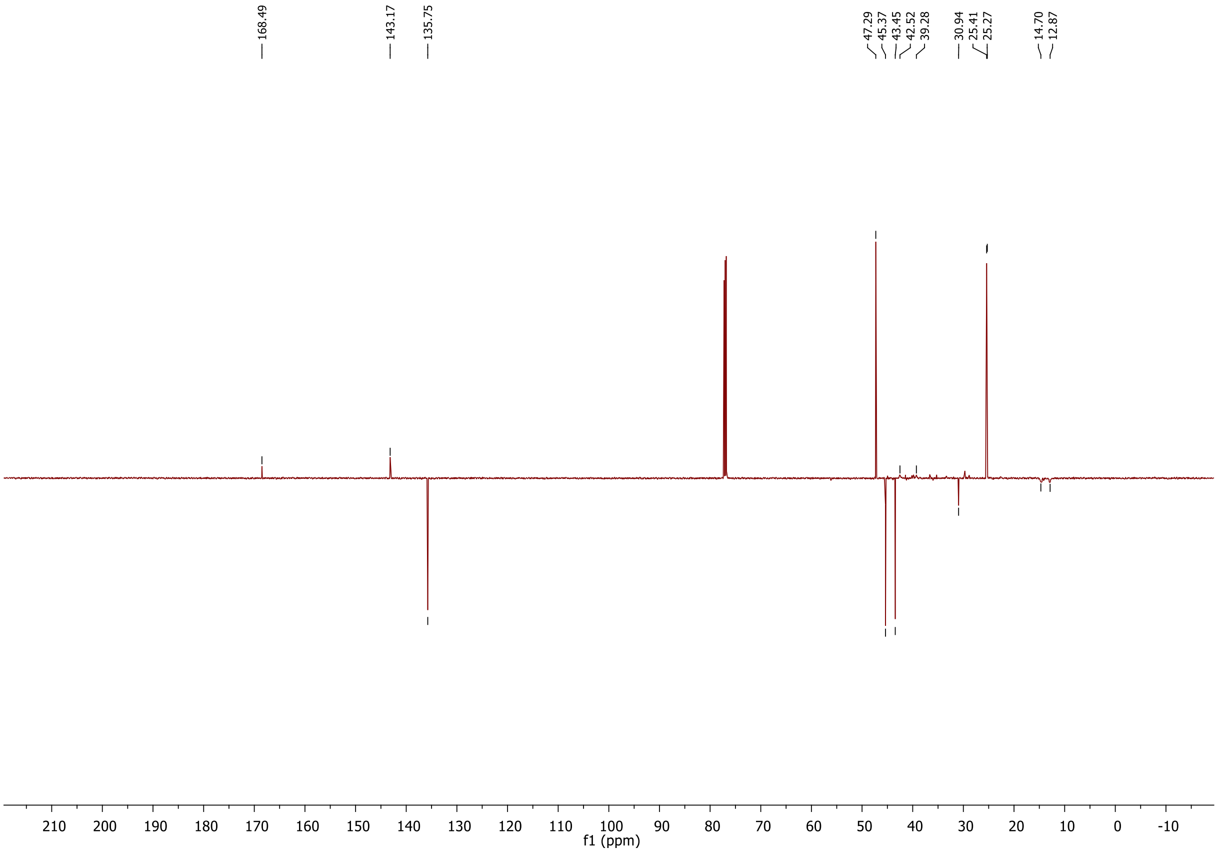

6) Bibliography

[1] H. Saltzman, J. G. Sharefkin, *Org. Synth.* **1963**, *43*, 60.

[2] Y. Wang, T. Yang, Q. Liu, Y. Ma, L. Yang, L. Zhou, Z. Xiang, Z. Cheng, S. Lu, L. A. Orband-Miller, et al., *Bioorg. Med. Chem.* **2015**, *23*, 5293–5302.

[3] C. Wiles, P. Watts, S. J. Haswell, E. Pombo-Villar, *Tetrahedron Lett.* **2002**, *43*, 2945–2948.

[4] R. A. Cox, M. McAllister, K. A. Roberts, P. J. Stang, T. T. Tidwell, *J. Org. Chem.* **1989**, *54*, 4899–4902.

[5] J. García-Fortanet, S. L. Buchwald, *Angew. Chemie Int. Ed.* **2008**, *47*, 8108–8111.

[6] D. B. Dess, J. C. Martin, *J. Am. Chem. Soc.* **1991**, *113*, 7277–7287.

[7] S. Menon, D. Sinha-Mahapatra, J. W. Herndon, *Tetrahedron* **2007**, *63*, 8788–8793.

[8] J. A. May, B. M. Stoltz, *J. Am. Chem. Soc.* **2002**, *124*, 12426–12427.

[9] H.-C. Zhang, B. D. Harris, M. J. Costanzo, E. C. Lawson, C. A. Maryanoff, B. E. Maryanoff, *J. Org. Chem.* **1998**, *63*, 7964–7981.

[10] W. Yu, Y. Mei, Y. Kang, Z. Hua, Z. Jin, *Org. Lett.* **2004**, *6*, 3217–3219.

[11] D. Christopher Braddock, J. Clarke, H. S. Rzepa, *Chem. Commun.* **2013**, *49*, 11176.

[12] P. Das, T. Mita, M. J. Lear, M. Hirama, *Chem. Commun.* **2002**, 2624–2625.

[13] T. Fujihara, K. Tatsumi, J. Terao, Y. Tsuji, *Org. Lett.* **2013**, *15*, 2286–2289.

[14] G. A. Olah, S. C. Narang, B. G. B. Gupta, R. Malhotra, *J. Org. Chem.* **1979**, *44*, 1247–1251.

[15] K. Nomura, T. Hirayama, S. Matsubara, *Chem. - An Asian J.* **2009**, *4*, 1298–1303.

[16] C. Hollmann, P. Eilbracht, *Tetrahedron* **2000**, *56*, 1685–1692.

[17] T. Fujihara, Y. Katafuchi, T. Iwai, J. Terao, Y. Tsuji, *J. Am. Chem. Soc.* **2010**, *132*, 2094–2098.

7) X-ray Analysis

The X-ray intensity data were measured on Bruker D8 Venture diffractometer equipped with multilayer monochromator, Mo K/α INCOATEC micro focus sealed tube and Oxford cooling system. The structure was solved by *direct methods* and refined by *full-matrix least-squares techniques*. Non-hydrogen atoms were refined with *anisotropic displacement parameters*. Hydrogen atoms were inserted at calculated positions and refined with riding model. The following software was used: *Bruker SAINT software package*[i] using a narrow-frame algorithm for frame integration, *SADABS*[ii] for absorption correction, *OLEX2*[iii] for structure solution, refinement, molecular diagrams and graphical user-interface, *Shelxle*[iv] for refinement and graphical user-interface *SHELXS-2015*[v] for structure solution, *SHELXL-2015*[vi] for refinement,

Platon[vii] for symmetry check. Experimental data and CCDC-Codes Experimental data and CCDC-Code (Available online: http://www.ccdc.cam.ac.uk/conts/retrieving.html) can be found in Table S1. Crystal data, data collection parameters, and structure refinement details are given in Tables S2 to S3. Crystal structure and packing view are visualized in Figure S3 to S4.

Table S1 Experimental parameter and CCDC-Code.

Sample	Machine	Source	Temp.	Detector Distance	Time/ Frame	#Frames	Frame width	CCDC
	Bruker		[K]	[mm]	[s]		[°]	
**11fa**	D8	Mo	100	30	1	600	0.300	198410 8

Rac-(1S,2S)-2-(4-bromobenzoyl)cyclopropyl)methyl methanesulfonate

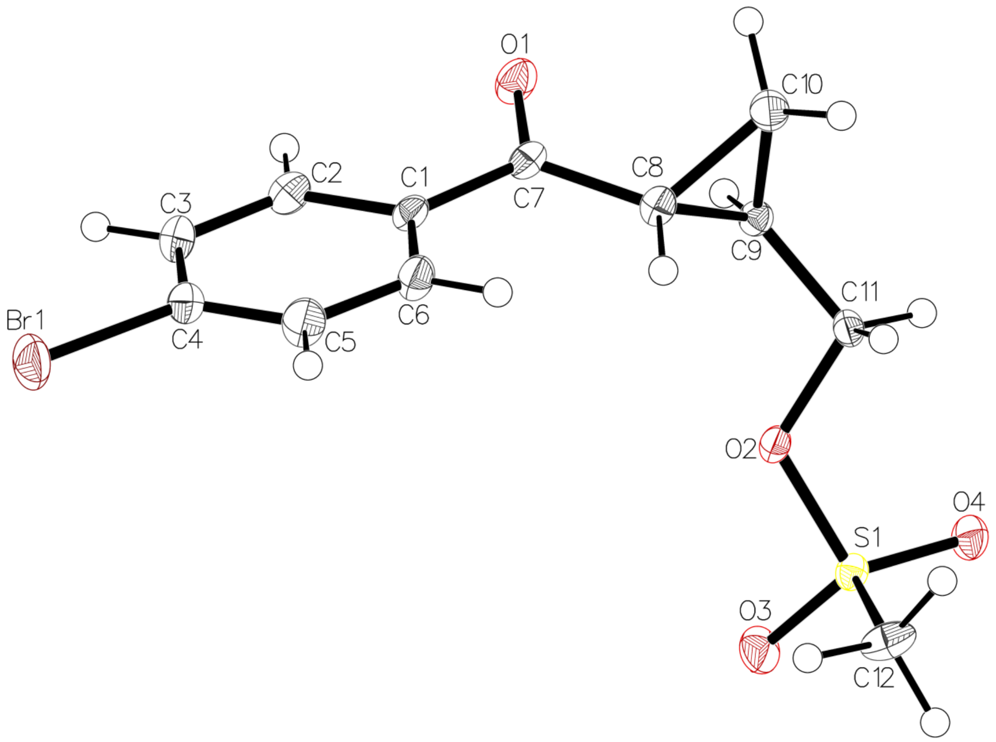

Figure S 3 - Crystal structure, drawn with 50% displacement ellipsoid. The bond precision for C-C single bonds is 0.0030Å. One short interaction could be detected Br1-O3= 2.957Å.

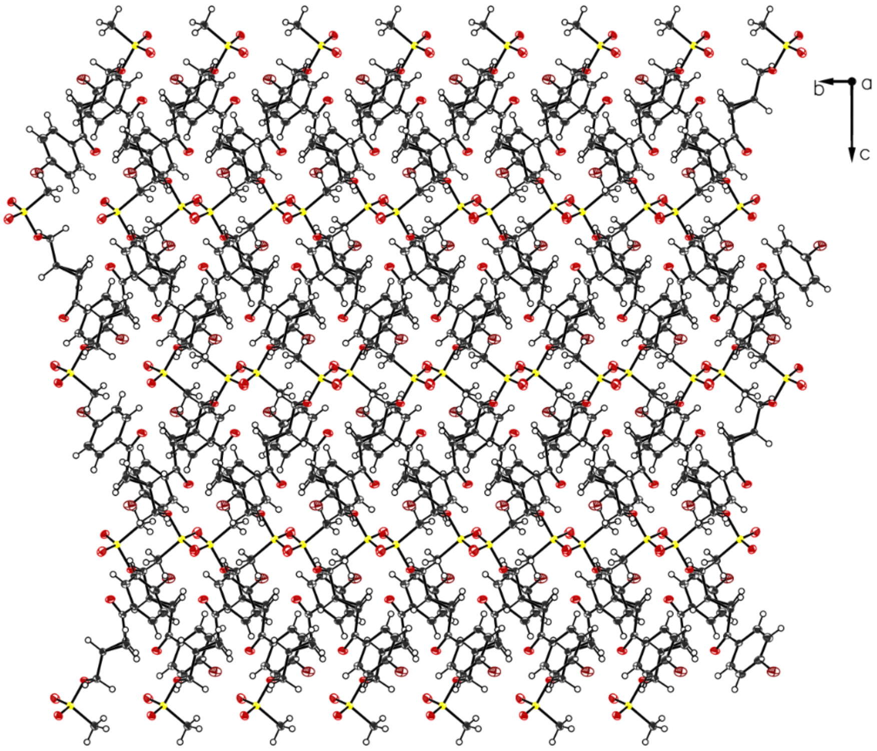

Figure S 4 - Packing view in plane bc.

Table S2 Sample and crystal data.

**Chemical formula**	C12H13BrO4S	**Crystal system**	monoclinic	
**Formula weight [g/mol]**	333.19	**Space group**	*P21/c*	
**Temperature [K]**	100	**Z**	4	
**Measurement method**	\f and \w scans	**Volume [Å^3]**	1336.28(7)	
**Radiation (Wavelength [Å])**	MoKα (λ = 0.71073)	**Unit cell dimensions [Å] and [°]**	15.4172(4)	90
**Crystal size / [mm^3]**	0.4 × 0.2 × 0.2		4.9257(2)	103.9620(11)
**Crystal habit**	clear colourless block		18.1321(5)	90
**Density (calculated) / [g/cm^3]**	1.656	**Absorption coefficient / [mm^{-1}]**	3.235	
**Abs. correction Tmin**	0.2781	**Abs. correction Tmax**	0.4932	
**Abs. correction type**	multiscan	**F(000) [e$^-$]**	672	

Table S3 Data collection and structure refinement.

<table>
<tr><td>Index ranges</td><td>-18 ≤ h ≤ 19, -6 ≤ k ≤ 6,
-22 ≤ l ≤ 21</td><td>Theta range for data collection [°]</td><td colspan="2">4.63 to 52.742</td></tr>
<tr><td>Reflections number</td><td>8837</td><td>Data / restraints / parameters</td><td colspan="2">2680/0/164</td></tr>
<tr><td>Refinement method</td><td>Least squares</td><td rowspan="2">Final R indices</td><td>all data</td><td>R1 = 0.0352, wR2 = 0.0743</td></tr>
<tr><td>Function minimized</td><td>Σ w(F_o^2 - F_c^2)^2</td><td>I>2σ(I)</td><td>R1 = 0.0275, wR2 = 0.0697</td></tr>
<tr><td>Goodness-of-fit on F^2</td><td>1.044</td><td rowspan="2">Weighting scheme</td><td colspan="2">w=1/[σ2(Fo2)+(0.0356P)2 +0.8893P]</td></tr>
<tr><td>Largest diff. peak and hole [e Å^{-3}]</td><td>0.59/-0.61</td><td colspan="2">where P=(F_o^2+2F_c^2)/3</td></tr>
</table>

---

[i] Bruker SAINT v8.38B Copyright © 2005-2019 Bruker AXS
[ii] L. Krause, R. Herbst-Irmer, G. M. Sheldrick and D. Stalke. J. Appl. Cryst. (2015). 48, 3-10.
[iii] Dolomanov, O.V., Bourhis, L.J., Gildea, R.J, Howard, J.A.K. & Puschmann, H. , OLEX2, (2009), J. Appl. Cryst. 42, 339-341
[iv] C. B. Huebschle, G. M. Sheldrick and B. Dittrich, ShelXle: a Qt graphical user interface for SHELXL, J. Appl. Cryst., 44, (2011) 1281-1284
[v] Sheldrick, G. M.. Acta Cryst (2008). A64, 112-122
[vi] Sheldrick, G. M.. Acta Cryst. (2015). C71, 3-8
[vii] A. L. Spek, Acta Cryst. 2009, D65, 148-155.
